# Heavyweight Champion: Caesium Diorganophosphides Outperform Lighter Congeners in the Catalytic Hydrophosphination of Alkenes and Alkynes

**DOI:** 10.1002/anie.202516376

**Published:** 2025-09-10

**Authors:** Felix Krämer, Michelle H. Crabbe, Israel Fernández, Robert E. Mulvey

**Affiliations:** ^1^ Department of Pure and Applied Chemistry University of Strathclyde Glasgow G1 1XL UK; ^2^ Departamento de Química Orgánica I Facultad de Ciencias Químicas and Centro de Innovación en Química Avanzada (ORFEO‐CINQA) Universidad Complutense de Madrid Madrid 28040 Spain

**Keywords:** Alkali metals, Alkenes, Alkynes, Catalysis, Hydrophosphination

## Abstract

We present a study of the ability of our recently reported well‐defined crown ether‐coordinated alkali metal phosphides **1^AM^** to catalyze hydrophosphination (HP) of alkynes and alkenes. In a comparative study including reaction monitoring by ^1^H NMR spectroscopy, we show that the activity of caesium compound **1^Cs^** greatly exceeds that of its lighter congeners, enabling us to solve some reported challenges of catalytic hydrophosphination. Through the rarely used application of dialkyl phosphines, we were able to produce trialkyl phosphines from HP of styrene derivatives and activated as well as non‐activated alkynes by catalytic HP with *^n^* Bu_2_PH and *^t^* Bu_2_PH. Using *^t^* BuPhPH, P‐chiral products were obtained, still in racemic mixtures showcasing this system's potential. We also proved the method is suited for preparing unsymmetrical ethylene‐bridged bisphosphines by HP of Ph_2_P(vinyl) isolable in high yields. These advances hint that well‐defined organocaesium compounds could make a long‐term impact in chemistry.

## Graphical abstract

**The heavier, the better**! We report that the caesium congener is the most active and versatile catalyst amongst a full group set of well‐defined crown ether supported alkali metal diphenyl phosphides for catalytic hydrophosphination of various substrates and phosphines including aryl and alkyl phosphines. Furthermore, methods to generate P‐chiral products, unsymmetrical ethylene‐bridged bisphosphines, and alkene isomerization are presented.

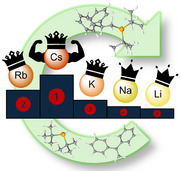

## Introduction

**Scheme 1:**
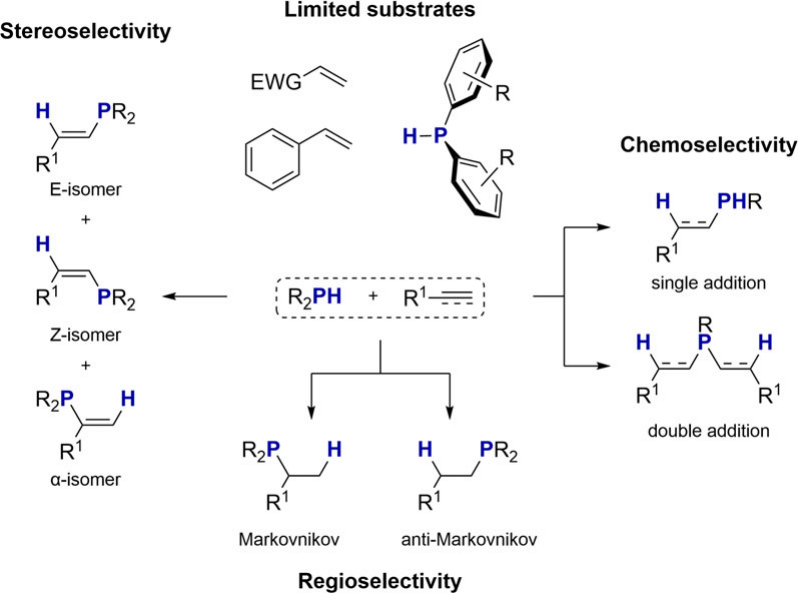
Key challenges remaining in catalytic hydrophosphination chemistry. Reproduced from reference.^[^ ^73^ ^]^

Due to their numerous applications in synthesis, *s*‐block complexes of lithium, and to a much lesser extent of sodium and potassium, have been the subject of several review articles emphasising the indispensability of these polar organometallic compounds.^[^ ^1^, ^2^, ^3^, ^4^, ^5^, ^6^, ^7^, ^8^ ^]^ Until recently, however, the use of alkali metal compounds was mainly limited to stoichiometric reactions. This limitation is starting to be overcome, as several examples are known in which organo‐alkali metal compounds were successfully applied catalytically in reactions such as Brønsted base catalysed C─C bond couplings,^[^ ^9^, ^10^, ^11^, ^12^, ^13^, ^14^, ^15^, ^16^, ^17^, ^18^, ^19^, ^20^, ^21^, ^22^, ^23^, ^24^ ^]^ C─N bond couplings,^[^ ^25^ ^]^ dehydrogenations and dehydrocouplings,^[^ ^26^, ^27^, ^28^, ^29^, ^30^, ^31^, ^32^, ^33^, ^34^, ^35^ ^]^ hydroboration,^[^ ^36^, ^37^, ^38^, ^39^, ^40^, ^41^, ^42^, ^43^, ^44^ ^]^ hydrogenations and transfer hydrogenations,^[^ ^45^, ^46^, ^47^, ^48^, ^49^, ^50^, ^51^, ^52^ ^]^ hydrosilylations,^[^ ^53^, ^54^, ^55^, ^56^, ^57^, ^58^ ^]^ isomerization of alkenes,^[^ ^59^ ^]^ hydrophosphorylation,^[^ ^60^, ^61^ ^]^ and hydrophosphination.^[^ ^62^, ^63^, ^64^, ^65^, ^66^, ^67^, ^68^, ^69^ ^]^ The first catalytic hydrophosphination (HP), reported in 1990, used a platinum catalyst^[^ ^70^ ^]^ and since then the field has been dominated by transition metal complexes.^[^ ^71^, ^72^, ^73^, ^74^, ^75^ ^]^ Now, main group catalytic systems are beginning to make an impact in HP chemistry. Besides some calcium‐based systems,^[^ ^76^, ^77^, ^78^, ^79^ ^]^ several bimetallic main group systems have been reported.^[^ ^80^, ^81^ ^]^ Although a few approaches have used alkali metal systems, mostly of potassium, these are underrepresented in the HP field, although such basic compounds are predestined for this important application.^[^ ^74^ ^]^ As recently discussed in an excellent review by Webster, there are difficult hurdles to overcome in the field of catalytic HP chemistry (Scheme 1).^[^ ^73^ ^]^

First, substrate scope is limited. Usually only activated multiple bonds can react with comparatively less basic diaryl phosphines such as Ph_2_PH. Dialkyl phosphines are usually not included in studies or dismissed due to little or no activity. The second challenge concerns regioselectivity. Although most reported systems yield anti‐Markovnikov products, it is exceedingly more difficult to selectively obtain Markovnikov products. Depending on the activity of the catalyst, problems are seen with the chemoselectivity, whereby the double addition product is often made, and the capture of the single addition products appears problematic. If compounds with triple bonds are used as substrates, product mixtures of E‐, Z‐, and *α*‐isomers are obtained, which are difficult or impossible to separate. To overcome some or all these problems is important, since HP offers a simple, atom‐economical, and sustainable approach to the synthesis of valuable phosphines, especially if the catalysts are main group based and thus more sustainable than precious transition metal catalysts.

**Scheme 2:**
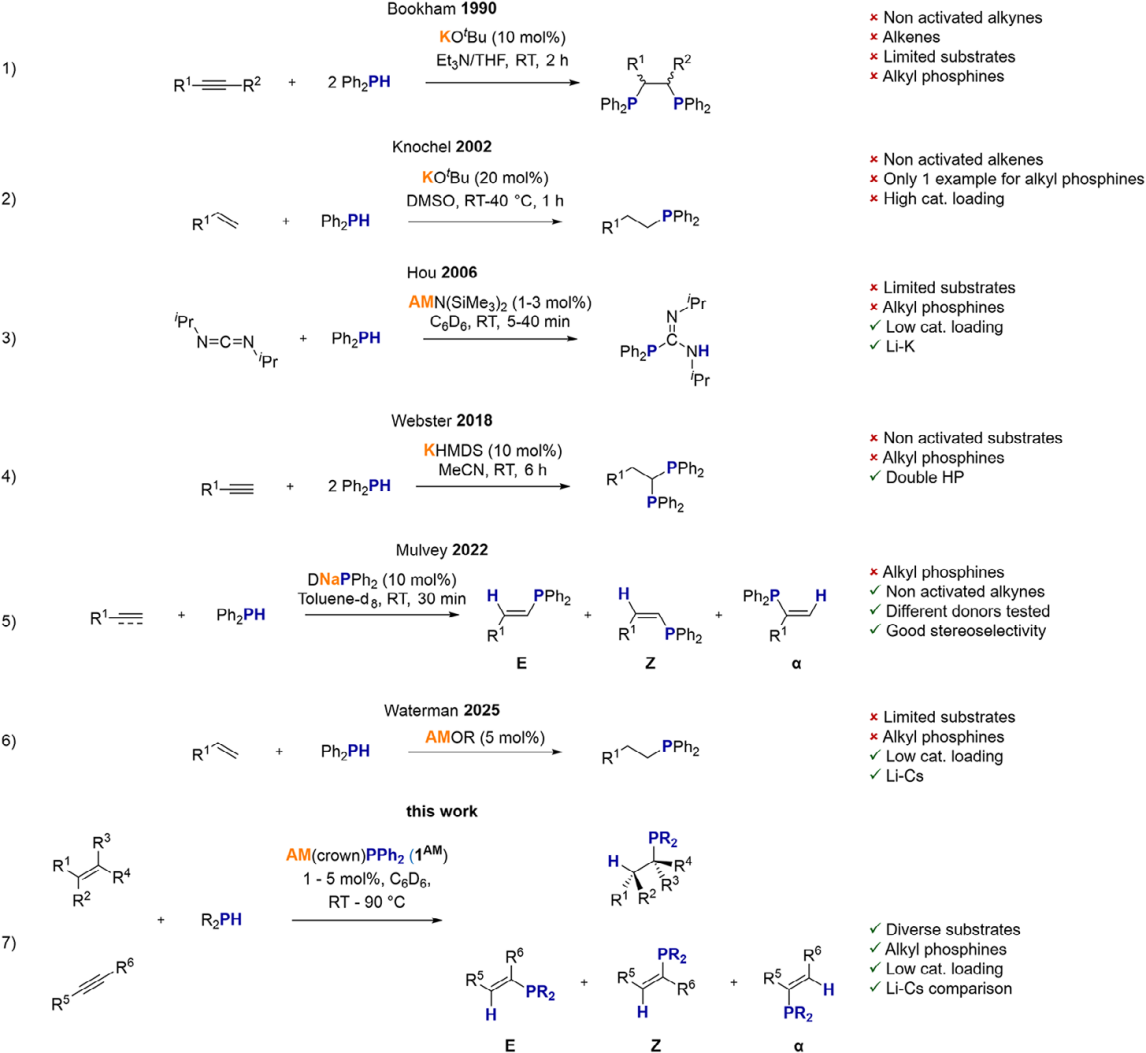
Alkali metal catalyzed hydrophosphination reactions.

Here we report a ground‐breaking study on well‐defined crown ether coordinated alkali metal diphenyl phosphides of formula AM(crown)PPh_2_ [AM = Li (**1^Li^**), Na (**1^Na^**), K (**1^K^**), Rb (**1^Rb^**), Cs (**1^Cs^**), crown = 15‐crown‐5 for Li and Na; 18‐crown‐6 for K‐Cs] in the catalytic hydrophosphination of C─C double and triple bonds conquering some of the previously mentioned challenges (Scheme 2).

## Results and Discussion

**Scheme 3:**
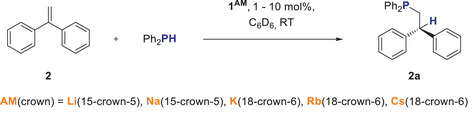
Benchmark HP reaction and conditions.

Based on our promising results on the **1^Na^** catalyzed HP of alkynes, we pondered whether heavier Rb and Cs congeners, could exhibit higher activity in HP reactions.^[^ ^66^, ^82^ ^]^ Waterman recently reported the first usage of Group one salt alkoxides as pre‐catalysts for HP of styrenes with Ph_2_PH finding increased activity on descending the group,^[^ ^69^ ^]^ a trend we had reported earlier for Li to Cs in transfer hydrogenation catalysis.^[^ ^83^ ^]^ Here, we focus on NMR studies of HP reactions starting from well‐defined crown ether supported alkali metal phosphides which we postulate to be the actual catalysts. For this purpose, we chose HP of 1,1‐diphenylethylene (**2**) with Ph_2_PH (**a**) as our benchmark (Scheme 3). Styrene, used often in the literature, is rapidly polymerized by **1^Cs^** under catalytic conditions.

Table 1 outlines our catalytic results. **1^Li^** and **1^Na^** with a catalyst loading of 10 mol% and concentration of 1 M (**1^Li^**) and 0.5 M (**1^Na^**) show similar activity at room temperature with a reaction time of 30 min to reach full conversion. Decreasing the catalyst loading for **1^Na^** to 1 mol% (Table 1, entry 6) results in a conversion of only 30% after 14 h. **1^Li^** shows no conversion after 24 h. When **1^Cs^** is used under the same conditions (10 mol%, 1 M), full conversion takes only 15 min. If the catalyst loading and concentration are successively reduced (Table 1, entries 4, 5, and 9), then even with only 1 mol% and a concentration of 0.01 M full conversion occurs after 12 min. Pleasingly, further reducing the concentration (1 mol%, 0.005 M) does not slow down the reaction. Interestingly, with 0.1 mol% and 0.001 M the reaction subsides, and no conversion is seen in NMR spectra. For comparison, the conditions were set at 1 mol% catalyst loading and a concentration of 0.01 M. To probe whether the metal has an influence on reactivity, and not just the weaker bond between the AM(crown) moiety and the PPh_2_ fragment leading to increased reactivity when descending the group, the metal‐free ammonium counterpart [*^n^* Bu_4_N][PPh_2_] was made and applied in the HP of **2** with Ph_2_PH (5 mol%, 0.05 M) (Table 1, entry 11). The conversion was less than 1% after 20 h, demonstrating the importance of the alkali metal in the reaction. The abstraction of the PPh_2_ fragment also inhibits activity (Table 1, entry 12). The dramatic increase in activity in C_6_D_6_ on descending Group 1 is comparable to the trend seen in the aforementioned saline study by Waterman but, by using well‐defined organometallic compounds, the catalyst loading can be reduced to 1 mol% compared to 5 mol% which suggests these compounds generated in situ in earlier studies by adding an alkali metal base to the substrate/phosphine mixture are the actual catalysts. Changing the solvent from C_6_D_6_ to pyridine‐D_5_ leads to a dramatic increase in the catalytic activity of **1^Na^** (full conversion after 5 h cf., 30% after 14 h in C_6_D_6_). This trend was even more drastic when MeCN‐D_3_ was applied where **1^Na^** reached 97% conversion in only 21 min (see Supporting Information Table S2). We probed the performance of all alkali metals in MeCN‐D_3_ and observed that the activity boost decreases on descending Group 1. We attribute the increased reactivity to the increased nucleophilicity of the Ph_2_P fragment in polar solvents. The difference in the improvement of the activity can be explained with a maximum nucleophilicity of a certain nucleophile. Attaching a heavier alkali metal to Ph_2_P^−^ increases its nucleophilicity by lowering the bond strength between cation and anion;^[^ ^82^ ^]^ the dissociation of AM(crown)^+^ and Ph_2_P^−^ can be facilitated in polar solvents which in turn leads to an increase in nucleophilicity. Also, sequestration of the cation from the nucleophile increases the activity of the alkali metal species in the catalytic HP which was shown by us and Waterman.^[^ ^66^, ^69^ ^]^ Thus, with crown‐ether supported caesium the maximum nucleophilicity of Ph_2_P^−^ can be reached in nonpolar solvents such as benzene where the lighter crown ether supported alkali metals require polar solvents to reach the maximum (K and Rb) or very high nucleophilicity (Na and Li) in polar solvents such as MeCN‐D_3_.

**Table 1:** Conditions of catalytic hydrophosphination of **2** with Ph_2_PH using **1^AM^** as catalyst

Entry	Catalyst	Cat. / mol%	c / mol/L	Time	Conversion (%)	TOF / h^−1^
1	**1^Li^**	10	1	20 min	>99	30
2	**1^Na^**	10	0.5^a)^	20 min	>99	30
3	**1^Cs^**	10	1	15 min	>99	–
4	**1^Cs^**	5	1	10 min	>99	–
5	**1^Cs^**	2	0.025	22 min	>99	–
6	**1^Na^**	1	0.01	**14 h**	30	2
7	**1^K^**	1	0.01	63 min	>99	94
8	**1^Rb^**	1	0.01	28 min	>99	211
9	**1^Cs^**	1	0.01	12 min	>99	493
10	**1^Cs^**	1	0.005	12 min	>99	493
11	**[*^n^* Bu_4_N][PPh_2_]**	5	0.05	**20 h**	<1	–
12	**[Cs][B(C_6_F_5_)_4_]**	5	0.03	**3 h**	0	–

Conversions determined by NMR integration relative to C_6_Me_6_ or adamantane as an internal standard based on the decrease in the substrate signal. TOFs are calculated from the conversion divided by the time required for conversion. ^a)^Lower concentration due to limits in solubility. Reactions run at room temperature.

**Figure 1:**
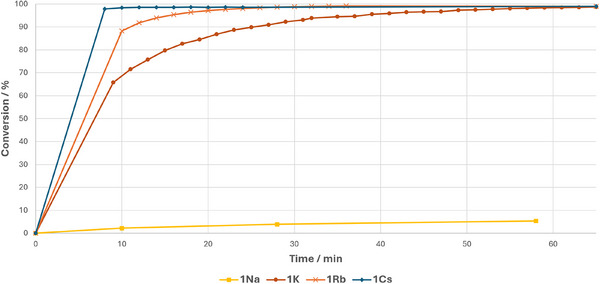
Conversion plotted against time for catalytic hydrophosphination of **2** with Ph_2_PH using 1^AM^ as catalyst. Conversions determined against C_6_Me_6_ or adamantane as internal standard.

Both Table 1 (entry 6–9) and Figure 1 show the dramatic increase in catalytic activity in the benchmark reaction on descending the group. While **1^Na^** reaches only 30% conversion after 14 h, **1^K^** is more active, with the reaction completed after 1 h. A smaller step is seen from potassium to rubidium, but nevertheless reaction time is halved when **1^Rb^** is used as a catalyst. **1^Cs^** shows by far the highest activity, reflected in a remarkable turnover frequency (TOF) of 493 h^−1^, compared to 211 h^−1^ (**1^Rb^**), 94 h^−1^ (**1^K^**), and 2 h^−1^ (**1^Na^**).

**Scheme 4:**
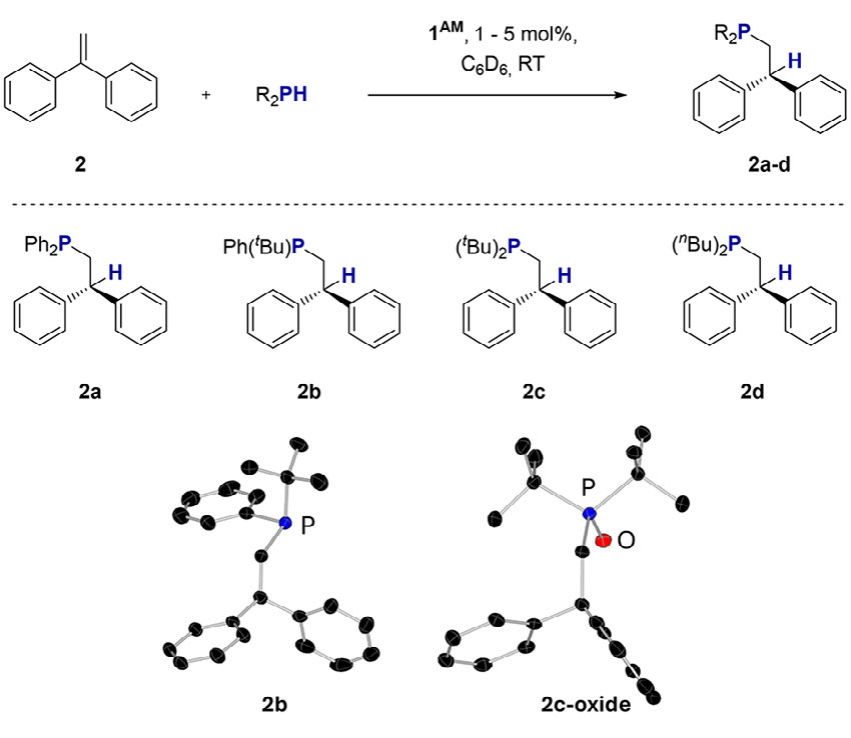
Benchmark HP reaction with different phosphines and molecular structures of isolated reaction products 2b and 2c‐oxide. Hydrogen atoms are omitted for clarity, ellipsoids at 30% probability.

Following these promising results, next we challenged our catalysts to more demanding substrates by successively replacing phenyl groups on the phosphine by alkyl groups (Scheme 4).

**Figure 2:**
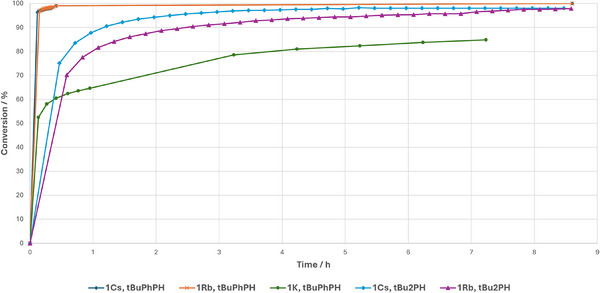
Conversion plotted against time for the catalytic hydrophosphination of **2** with *^t^* BuPhPH **b**), *^t^* Bu_2_PH **c**) and *^n^* Bu_2_PH **d**) with **1** ^**AM**^ as catalyst. Conversions are determined against C_6_Me_6_ or adamantane as internal standard.

We screened the catalytic activity of **1^K^**, **1^Rb^**, and **1^Cs^** with the alkyl/aryl phosphine *^t^* BuPhPH (**b**). A decrease in activity was seen for **1^K^**, whereas **1^Rb^** and **1^Cs^** show comparable reactivity to that with Ph_2_PH (Table 2, Figure 2). With the bulky dialkyl phosphine *^t^* Bu_2_PH (**c**), a difference between **1^Rb^** and **1^Cs^** is now seen. First, the conditions must be adjusted and the catalyst loading and concentration (5 mol%, 0.05 M) must be increased to observe a rapid reaction. Second, reaction of **2** with **c** shows that **1^Cs^** is most active reaching 97% conversion in half the time compared to **1^Rb^**. Interestingly, using the less sterically demanding dialkyl phosphine *^n^* Bu_2_PH (**d**), a longer reaction time is seen compared to that of *^t^* Bu_2_PH (**c**) under the same conditions, which represents an exception for substrate **2**. In addition, we were able to elucidate the molecular structures of **2b** and the oxide of **2c** in the crystal using SC‐XRD methods (Scheme 4).

**Table 2:** Conditions of catalytic hydrophosphination of **2** with *^t^* BuPhPH (**b**), *^t^* Bu_2_PH (**c**) and *^n^* Bu_2_PH (**d**) with **1^AM^** as catalyst.

Entry	Catalyst	Cat./mol%	c / mol/L	Phosphine	Time	Conversion (%)	TOF / h^−1^
1	**1^K^**	1	0.01	*^t^* BuPhPH	**15 h**	90	5
2	**1^Rb^**	1	0.01	*^t^* BuPhPH	9 min	97	645
3	**1^Cs^**	1	0.01	*^t^* BuPhPH	7 min	97	726
4	**1^Rb^**	5	0.05	*^t^* Bu_2_PH	**7.1 h**	97	3
5	**1^Cs^**	5	0.05	*^t^* Bu_2_PH	**3.2 h**	97	6
6	**1^Cs^**	5 (75 °C)	0.05	*^t^* Bu_2_PH	**1 h**	96	19
7	**1^Cs^**	5 (75 °C)	0.05	*^n^* Bu_2_PH	**2.5 h**	86	7

Conversions determined by integration relative to C_6_Me_6_ or adamantane as an internal standard based on the decrease in the substrate signal. TOFs calculated from the conversion divided by the time required. Reactions run at room temperature.

## 1^Cs^ Catalysed Hydrophosphination of Alkenes

**Scheme 5:**
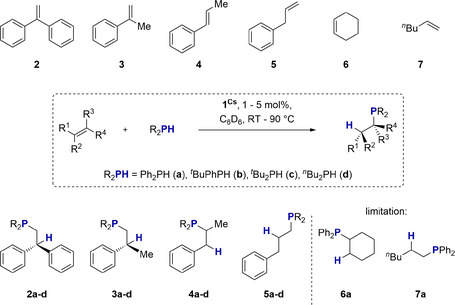
**1** ^**Cs**^ catalyzed HP of alkenes.

Next, we studied the styrene derivatives **3** and **4** as well as allylbenzene **5** and nonactivated alkenes **6** and **7** as possible substrates (Scheme 5). Unfortunately, **1^Cs^** shows no activity in the HP of **6** and **7**, even at elevated temperature and long reaction times (cf. Table 3, entries 5 and 6).

**Table 3:** **1^Cs^** catalyzed hydrophosphination of **2–7** with Ph_2_PH **a**), *^t^* BuPhPH **b**), *^t^* Bu_2_PH **c**), and *^n^* Bu_2_PH **d**)

Entry	Substrates	Phosphines	Cat./mol%	Time/h (min)	T/°C	Product	Yield/%
1	**2**	Ph_2_PH (**a**)	1	(8)	RT	**2a**	>99
2	**3**	Ph_2_PH (**a**)	5	2	90	**3a**	98
3	**4**	Ph_2_PH (**a**)	5	2	90	**4a**	90
4	**5**	Ph_2_PH (**a**)	5	2	90	**4a**	50
5	**6**	Ph_2_PH (**a**)	5	24	90	**6a**	n.r.
6	**7**	Ph_2_PH (**a**)	5	16	90	**7a**	n.r.
7	**2**	*^t^* BuPhPH (**b**)	1	(15)	RT	**2b**	98
8	**3**	*^t^* BuPhPH (**b**)	5	6	90	**3b**	74
9	**4**	*^t^* BuPhPH (**b**)	5	2	90	**4b**	81
10	**5**	*^t^* BuPhPH (**b**)	5	20	90	**4b**	n.r.^a)^
11	**2**	*^t^* Bu_2_PH (**c**)	1	22	90	**2c**	85
12	**2**	*^t^* Bu_2_PH (**c**)	5	4.2	RT	**2c**	98
13	**2**	*^t^* Bu_2_PH (**c**)	5	1.5	75	**2c**	98
14	**3**	*^t^* Bu_2_PH (**c**)	5	22	90	**3c**	n.r.
15	**4**	*^t^* Bu_2_PH (**c**)	5	22	90	**4c**	n.r.
16	**5**	*^t^* Bu_2_PH (**c**)	5	22	90	**4c**	n.r.^a)^
17	**2**	*^n^* Bu_2_PH (**d**)	5	2.5	75	**2d**	86
18	**3**	*^n^* Bu_2_PH (**d**)	5	4	90	**3d**	88
19	**4**	*^n^* Bu_2_PH (**d**)	5	4	90	**4d**	92
20	**5**	*^n^* Bu_2_PH (**d**)	5	4	90	**4d**	96

Yields determined against C_6_Me_6_ or adamantane as internal standard. ^a)^Only isomerization from **5** to **4** was seen.

For less activated styrene derivatives compared to **2**, we had to increase the catalyst loading to 5 mol% and raise the temperature to achieve short reaction times of a few hours. Although substrates **3** and **4** gave high yields (74%–98%) with phosphines **a**, **b**, and **d**, very bulky phosphine **c** appeared unreactive except with substrate **2**. Interestingly, respective products **4a** and **4d** were obtained in the HP of substrate **5** with Ph_2_PH and *^n^* Bu_2_PH. This can be explained by isomerization of **5** to its methyl isomer **4** and subsequent HP. For phosphines **b** and **c**, only isomerization was observed. This finding was surprising, as superbasic NaTMP (TMP = 2,2,6,6‐tetramethylpiperidide) was recently used for such isomerization reactions by Hevia.^[^ ^59^ ^]^ In general, the following trends can be derived from the data in Table 3: i) the activity of **1^Cs^** decreases on increasing the number of alkyl groups on the phosphine; ii) the sterically very demanding phosphine *^t^* Bu_2_PH can only be reacted with strongly activated substrates such as **2**; iii) the formal Markovnikov product **4a** can be made by “double catalysis” with allylbenzene **5**.

**Scheme 6:**
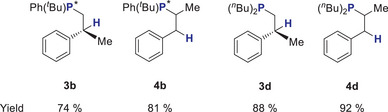
P‐chiral reaction products **3b** and **4b** and trialkyl phosphine products **3d** and **4d**.

Products **3b** and **4b** (Scheme 6), which represent P‐chiral phosphines whose synthesis represents one of the flagged hurdles in HP chemistry, deserve special attention. Reaction products **3d** and **4d** are also highlighted, as trialkyl phosphines were produced in impressive 88% and 92% yields, respectively, by using dialkyl phosphines as substrates, which has also been flagged as a challenge.

## 1^Cs^ Catalysed Hydrophosphination of Alkynes

**Scheme 7:**
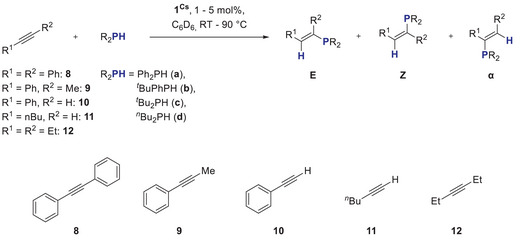
**1** ^**Cs**^ catalyzed HP of alkynes.

To further explore extending substrate scope, we also tested the use of activated (**8**–**10**) and nonactivated (**11** and **12**) alkynes as substrates (Scheme 7).

For HP of the activated alkynes **8**–**10** with Ph_2_PH, the reactions with 1 mol% **1^Cs^** were completed within a maximum of 4 h (Table 4, entries 1–3). For nonactivated alkynes **11** and **12**, the catalyst loading had to be increased to 5 mol% and reaction temperature raised to 90 °C to get conversion. Although the terminal alkyne 1‐hexyne (**11**) shows a yield of 73% after 4 h at 90 °C, the reaction with internal alkyne 3‐hexyne is significantly slower (25% after 22 h at 90 °C). The selectivity between *E*‐, *Z*‐, and *α‐*product is comparable to that found for **1^Na^**.^[^ ^66^ ^]^ When replacing phenyl groups on the phosphine with alkyl groups, 5 mol% catalyst must also be used and the reaction mixtures must be heated to 90 °C for 2–23 h (Table 4, entries 6–19). The *E*, *Z*, and *α*‐ratios for phosphines **b**‐**c** could not be clearly determined from the reaction mixtures using NMR spectroscopy. The same trends that were found for alkenes in Table 3 emerged: i) the activity of **1^Cs^** decreases with the increasing number of alkyl groups on the phosphine; ii) the sterically very demanding phosphine *^t^* Bu_2_PH can only be reacted with strongly activated substrates such as **8**; iii) internal, nonactivated alkynes are the most demanding substrates but can be reacted with all phosphines except *^t^* Bu_2_PH with satisfactory yields.

**Table 4:** **1^Cs^** catalyzed hydrophosphination of **8–12** with Ph_2_PH **a**), *^t^* BuPhPH **b**), *^t^* Bu_2_PH **c**), and *^n^* Bu_2_PH **d**).

Entry	Substrates	Phosphines	Cat./mol%	Time/h	T/°C	Yield/% (E:Z:α)
1	**8**	Ph_2_PH (**a**)	1	2	RT	>99 (1:0:0)
2	**9**	Ph_2_PH (**a**)	1	4	RT	>99 (1:2:0)
3	**10**	Ph_2_PH (**a**)	1	0.75	RT	>99 (1:1.5:0)
4	**11**	Ph_2_PH (**a**)	5	4	90	73 (1:3.5:4.4)
5	**12**	Ph_2_PH (**a**)	5	22	90	25 (1:0:0)
6	**8**	*^t^* BuPhPH (**b**)	5	18	90	n.r.
7	**9**	*^t^* BuPhPH (**b**)	5	2	90	93^a)^
8	**10**	*^t^* BuPhPH (**b**)	5	2	90	96^a)^
9	**11**	*^t^* BuPhPH (**b**)	5	18	90	87^a)^
10	**12**	*^t^* BuPhPH (**b**)	5	18	90	80^a)^
11	**8**	*^t^* Bu_2_PH (**c**)	5	4	90	99^a)^
12	**9**	*^t^* Bu_2_PH (**c**)	5	19	90	n.r.
13	**10**	*^t^* Bu_2_PH (**c**)	5	19	90	n.r.
14	**11**	*^t^* Bu_2_PH (**c**)	5	19	90	n.r.
15	**8**	*^n^* Bu_2_PH (**d**)	5	4	90	99^a)^
16	**9**	*^n^* Bu_2_PH (**d**)	5	19	90	50^a)^
17	**10**	*^n^* Bu_2_PH (**d**)	5	19	90	96^a)^
18	**11**	*^n^* Bu_2_PH (**d**)	5	4	90	98^a)^
19	**12**	*^n^* Bu_2_PH (**d**)	5	23	90	85^a)^

Yields determined against C_6_Me_6_ or adamantane as internal standard. ^a)^Mixture of isomers.

## Catalytic Studies of Additional Substrates

**Scheme 8:**
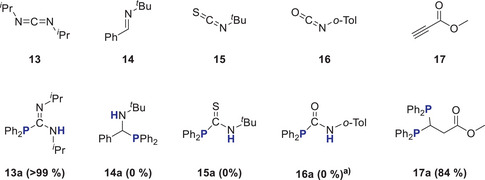
**1** ^**Cs**^ catalyzed HP of heterocumulenes **13**–**16** and double HP of methylpropiolate **17**. Yields determined against C_6_Me_6_ or adamantane as the internal standard. ^a)^Trimerization in a few minutes.

To explore a broader application for **1^Cs^** in HP chemistry and tolerance to functional groups, we tested additional substrates with 5 mol% catalyst (Scheme 8). Although the HP of *N*,*N*‐diisopropylcarbodiimide **13** proceeds rapidly within 30 min at RT and gives a yield of **13a** of 99%, imine **14** remains unchanged, even after heating the mixture to 75 °C for several hours. Isothiocyanate **15** shows no reaction, whereas isocyanate **16** reacts fully within a few minutes with precipitation of solid. The disappearance of all signals in the ^1^H NMR spectrum indicates a substrate cyclotrimerization. While Webster used 10 mol% KHMDS to convert **17** with 2 equivs Ph_2_PH to **17a** within 6 h,^[^ ^65^ ^]^ we noted a much increased reactivity of **1^Cs^** that yielded 84% of product **17a** upon slow addition of **17** to a mixture also containing **1^Cs^** and Ph_2_PH in C_6_D_6_ at RT. In addition to **17a**, several broad signals were seen in the ^1^H NMR spectrum, indicating partial substrate polymerization. When the substrate was added in a single addition, only 35% of product and mainly polymerization was seen. The single hydrophosphinated product was only detected in traces (4%) in the ^31^P‐NMR spectrum.

**Scheme 9:**
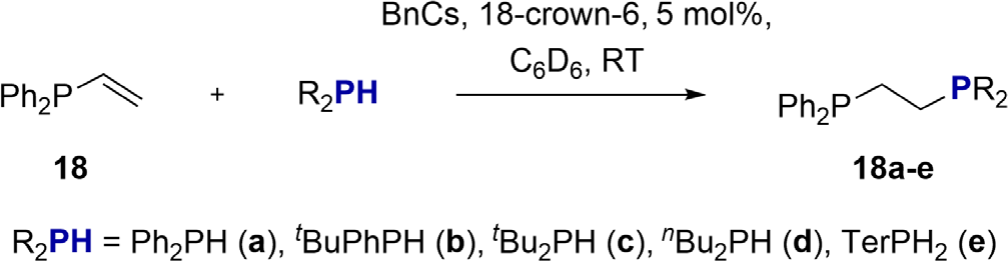
**1** ^**Cs**^ catalyzed HP of Ph_2_P(vinyl) **18**.

**Figure 3:**
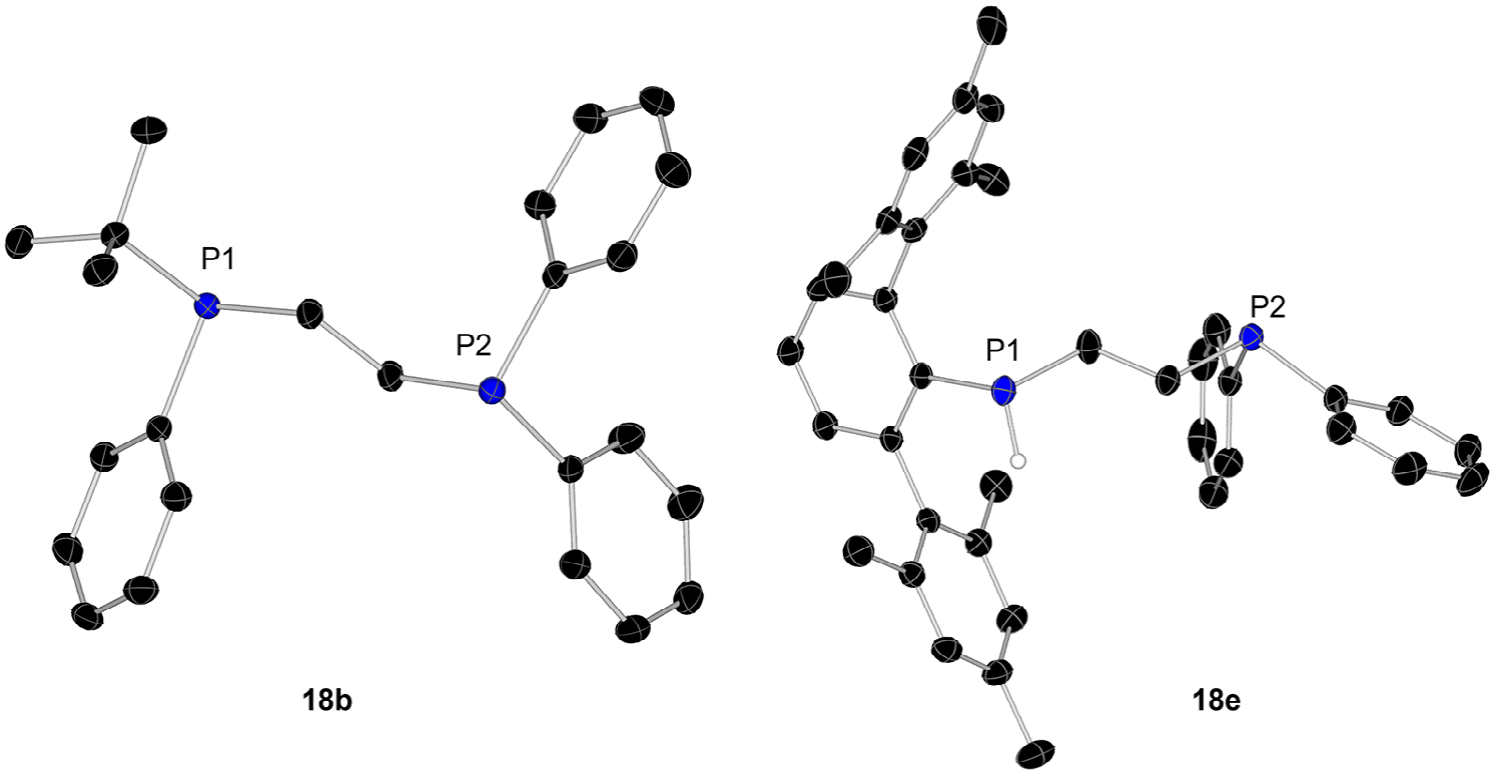
Molecular structures of products **18b** and **18e**. Hydrogen (except P‐H) atoms are omitted for clarity, and ellipsoids at 30% probability.

To probe synthetic utility further, we focused on the synthesis of unsymmetrical ethylene‐bridged bisphosphines of general formula Ph_2_PCH_2_CH_2_PR_2_. Starting from readily available Ph_2_P(vinyl) (**18**), various methods were described to synthesize unsymmetrical bisphosphines. Besides transition metal catalyzed HP,^[^ ^84^ ^]^ the radical approach using azobis(isobutyronitrile) (AIBN) is widely used.^[^ ^85^, ^86^, ^87^, ^88^, ^89^, ^90^, ^91^, ^92^ ^]^ Also, approaches using 20–30 mol% KO*^t^* Bu^[^ ^63^, ^93^, ^94^, ^95^, ^96^ ^]^ as well as catalytic amounts of LiO*^t^* Bu^[^ ^97^ ^]^ or *para*‐tolyllithium^[^ ^98^ ^]^ have been noted. We tested the reaction of **18** with the Ph_2_PH (**a**), *^t^* BuPhPH (**b**), *^t^* Bu_2_PH (**c**), and *^n^* Bu_2_PH (**d**) set in the presence of 5 mol% benzyl caesium (BnCs) and 18‐crown‐6 at RT (Scheme 9). The reaction of BnCs with each phosphine forms the catalyst in‐situ and prevents formation of the Ph_2_PCH_2_CH_2_PPh_2_ by‐product. The reactions achieved full conversion of starting materials in less than 30 min, except for *^t^* Bu_2_PH, which took 4 h. Reaction products were isolated in good yields of **18a** (78%, solid), **18b** (69%, solid), **18c** (47%, oil), and **18d** (65%, oil) with the molecular structure of **18b** determined using SC‐XRD methods (Figure 3).

In an even more testing challenge for **1^Cs^**, we used the super bulky primary phosphine TerPH_2_ (**e**), Ter = bis‐2,6‐(2,4,6‐trimethylphenyl)‐phenyl) in HP of **18**. After the catalyst loading was increased to 10 mol% and the reaction was heated to 80 °C for 3 days, a conversion of **18** of 75% and the clean formation of a new set of signals were detectable from NMR. Compound **18e** was isolated in a yield of 30% and its molecular structure determined by SC‐XRD methods (Figure 3).

## Conclusion

In conclusion, well‐defined crown ether‐coordinated alkali metal phosphides **1^AM^** have been studied in catalytic hydrophosphination (HP) of alkenes and alkynes. Progress has been made on several challenging fronts including reactions of common and uncommon phosphines with activated and nonactivated substrates, and methods to generate P‐chiral products, unsymmetrical ethylene‐bridged bisphosphines, and alkene isomerization. Results vis‐à‐vis published systems that use alkali metal bases such as alkoxides or amides as precatalysts suggest that the phosphides described here are the bona fide catalysts. The key to these breakthroughs is the remarkable activity of caesium compound **1^Cs^** that exceeds greatly that of its lighter congeners. How caesium achieves this exceptional activity is not yet clear so future work will use a mix of experimental and quantum chemical methods to elucidate possible mechanistic pathways which is a multidimensional challenge in which nucleophilicity and in turn the catalytic activity is increased by adding a heavier alkali metal to the Ph_2_P^−^ fragment, by changing the polarity of the solvent, or by adding a complexing agent to AMPPh_2_. Although, we see only one pixel of the picture now, we believe that acclaiming caesium as a champion among alkali metals in these HP reactions is not hyperbole as it has also shown the highest activities in other recently reported homogeneous catalytic reactions of important organic small molecules.^[^ ^52^, ^83^ ^]^

## Supporting Information

Experimental details including NMR spectra of the catalytic reactions and the isolated products, SC‐XRD and refinement data are provided in the supporting information. Additional references cited in the supporting information.^[^ ^99^, ^100^, ^101^, ^102^, ^103^, ^104^, ^105^, ^106^, ^107^, ^108^, ^109^ ^]^

## Conflict of Interests

The authors declare no conflict of interest.

## Supporting information



Supporting Information

## Data Availability

Deposition numbers 2474763–2474767 contain the supplementary crystallographic data for this paper. These data are provided free of charge by the joint Cambridge Crystallographic Data Centre and Fachinformationszentrum Karlsruhe Access Structures service. Data that support the findings of this study are openly available in Pureportal.strath.ac.uk at https://doi.org/10.15129/d389955d‐785a‐4663‐a0b7‐952b0994b44e, reference number 297245127.
