# Supplementary material for: Heavyweight Champion: Caesium Diorganophosphides Outperform Lighter Congeners in the Catalytic Hydrophosphination of Alkenes and Alkynes
Source: Angew Chem Int Ed Engl. 2025 Sep 10;64(44):e202516376. doi: 10.1002/anie.202516376 (PMC12559459; doi:10.1002/anie.202516376)
Supplement: Supplementary file 1 — Supporting Information [file ANIE-64-e202516376-s001.pdf]

Heavyweight Champion: Caesium Diorganophosphides Outperform Lighter Congeners in the Catalytic Hydrophosphination of Alkenes and Alkynes

Felix Krämer[a]*, Michelle H. Crabbe[a], Israel Fernández[b] and Robert E. Mulvey[a]*

[a] Dr F. Krämer, M. H. Crabbe and Prof. Dr R. E. Mulvey,
Department of Pure and Applied Chemistry, University of Strathclyde,
Glasgow G1 1XL, United Kingdom, E-mail: felix.kraemer@strath.ac.uk, r.e.mulvey@strath.ac.uk

[b] Prof. Dr I. Fernández
Departamento de Química Orgánica I, Facultad de Ciencias Químicas and Centro de Innovación en Química Avanzada (ORFEO-CINQA), Universidad Complutense de Madrid, 28040 Madrid, Spain.

Supporting Information

TOC

Section S1 – Experimental Details 3

Section S2 – NMR Spectra of the Isolated Compounds 9

Section S3 – NMR Spectra of the Catalytic Reactions 20

Section S3.1 – NMR Spectra of the catalytic hydrophosphination reactions of 1,1-diphenylethylene **2** 20

Ph_2PH 20

Different Solvents 28

^tBuPhPH 33

$^tBu_2PH$ 35

$^nBu_2PH$ 37

$Cs[B(C_6F_5)_4]$ as catalyst 38

Section S3.2 – NMR Spectra of the catalytic hydrophosphination reactions of alkenes **3**-**7** 40

Ph_2PH 40

^tBuPhPH 45

$^tBu_2PH$ 48

$^nBu_2PH$ 51

Section S3.3 – NMR Spectra of the catalytic hydrophosphination reactions of alkynes **8**-**12** 54

Ph_2PH 54

tBuPhPH 59

tBu_2PH 64

nBu_2PH 68

Section S3.4 – NMR Spectra of the catalytic hydrophosphination reactions of additional substrates **13**-**17**........ 73

Section S3.5 – NMR Spectra of the catalytic hydrophosphination reactions of Ph_2P(vinyl) **18**........ 79

Section S3.6 – Variable Temperature (VT) NMR Spectra of the reactions of $\mathbf{1}^{\mathbf{AM}}$ with **2**........ 84

Section S4 – Crystallographic details........ 89

References........ 90

Section S1 – Experimental Details

General Experimental Details: All synthetic procedures were carried out under a dry nitrogen atmosphere (N_2) using standard Schlenk techniques or in a glove box under an argon atmosphere (Ar). Before use, the glassware was pre-dried in an oven at 150 °C and then heated with a heat gun under vacuum. The solvents were dried, distilled and degassed using standard methods. C_6D_6 was dried over potassium, distilled, degassed and then stored in the glove box over activated molecular sieves (4 Å). n-Hexane, THF, Et_2O and toluene were dried in a Solvent Purification System (Innovative Technology, PS-Micro), degassed and stored under inert atmosphere over activated 4 Å molecular sieves. **1**^{AM} and ^{t}BuPhPH were synthesised as reported.[1] The phosphines Ph_2PH,[2] $^tBu_2PH$[3] and $TerPH_2$[4] were synthesised according to literature procedures. Ph_2PH was further purified by stirring over $LiAlH_4$ and a second distillation. $^nBu_2PNEt_2$ and $^nBu_2PH$ were synthesised according to modified procedures which are described in detail in the supporting information.[5-6] Benzyl caesium (BnCs)[7] and $[Ph_3C][B(C_6F_5)_4]$[8] were prepared according to literature procedures. All other substrates were obtained from commercial sources and were condensed, degassed and stored over activated 4 Å molecular sieves under argon in a glove box. Et_2NH, PCl_3, ^{n}BuLi (1.6 M, hexane), NaCl and H_2SO_4 were used as obtained from commercial sources. ^1H, $^{13}C\{^1H\}$ and $^{31}P\{^1H\}$ NMR spectra were recorded using an AV300, AV400 or AV500 MHz spectrometer. The chemical shifts (δ in ppm) in the ^1H and ^{13}C NMR spectra were referenced to the residual signals of the deuterated solvents. ^1H and $^{13}C\{^1H\}$ chemical shifts were reported against Me_4Si and $^{31}P\{^1H\}$ against H_3PO_4. Common abbreviations were used to describe the signal multiplications: s (singlet), d (doublet), t (triplet), q (quartet), dd (doublet of a doublet), m (multiplet) and b (broad).

General procedure for catalytic reactions in C_6D_6: For the experiments with 1 mol% catalyst stock solutions with a concentration of 0.05 M of **1**^{AM} were prepared with 2 mL C_6D_6. Amounts are listed in Table S2 in the supporting information. Separately a standard solution with a concentration of 0.125 M was prepared with adamantane or C_6Me_6. In a Young-NMR-Tube standard solution (0.4 mL, 0.05 mmol 10 eq.) was combined with phosphine (0.5 mmol, 100 eq.) and substrate (0.5 mmol, 100 eq.) and a ^1H NMR spectrum was recorded. Then catalyst solution (0.1 mL, 0.005 mmol 1 eq.) was added, and NMR spectra were recorded in given time intervals to monitor the conversions. For cat loadings higher than 1 mol% the catalyst was added as a solid (amounts in Table S1 in the supporting information) with additional C_6D_6 (0.1 mL). When the reactions were heated, the temperatures and times are given in Tables 2-4 for each substrate.

Table S1. Amounts of **1**^{AM} and phosphines **a**-**d** used for stock solutions and catalytic reactions.

	1 mol% (2 mL stock solution)	2 mol% (solid)	5 mol% (solid)	10 mol (solid)	Phosphines	0.5 mmol
**1**^{Li}	-	-	-	21 mg, 50 μmol	Ph_2PH (**a**)	87.0 mL
**1**^{Na}	44 mg, 0.1 mmol	-	-	11 mg, 25 μmol	^{t}BuPhPH (**b**)	89.8 mL
**1**^{K}	49 mg, 0.1 mmol	-	-	-	$^tBu_2PH$ (**c**)	94.8 mL
**1**^{Rb}	53 mg, 0.1 mmol	-	13 mg, 25 μmol	-	$^nBu_2PH$ (**d**)	92.8 mL
**1**^{Cs}	58 mg, 0.1 mmol	2.8 mg, 12 μmol	15 mg, 25 μmol	-		

General procedure for catalytic reactions in polar solvents: For the experiments in polar solvents Ph_2PH (0.5 mmol, 100 eq.), 1,1-DPE (0.5 mmol, 100 eq.) and C_6Me_6 (8.1 mg, 0.05 mmol, 10 eq.) or toluene (10.6 μL, 100 μmol, 20 eq.) for MeCN-D_3 were added into a Young-NMR-Tube with 0.5 mol of the solvent and ^1H NMR spectra were recorded. Then catalyst **1**^{AM} (2.1 mg (**1**^{Li}); 2.2 mg (**1**^{Na}); 2.4 mg (**1**^{K}); 2.7 mg (**1**^{Rb}); 2.9 mg (**1**^{Cs}); 0.005 mmol 1 eq.) was added as a solid, and NMR spectra were recorded

in given time intervals to monitor the conversions. The times and reached conversions are summarized in Table S2.

Table S2. Times, conversions and TOF (calculated at given conversion conversion) of the $\mathbf{1^{AM}}$ catalysed HP of 1,1-DPE with Ph_2PH in different solvents. Conversions determined by NMR integration relative to C_6Me_6 or toluene as an internal standard based on the decrease in the substrate signal.

		**C_6D_6**			**Pyridine-D_5**			**MeCN-D_3**		
	Cat. / Mol%	Time / min	Conv. / %	TOF / h^{-1}	Time / min	Conv. / %	TOF / h^{-1}	Time / min	Conv./ %	TOF / h^{-1}
$\mathbf{1^{Li}}$	1	**24 h**	<1	0	**24 h**	<1	0	**24 h**	9	0.38
$\mathbf{1^{Li}}$	5	22	81	44	11	99	108	17	98	69
$\mathbf{1^{Na}}$	1	**14 h**	30	2	**5 h**	90	18	21	97	262
$\mathbf{1^{K}}$	1	45	97	129	-	-	-	5	97	1174
$\mathbf{1^{Rb}}$	1	20	97	291	-	-	-	12	97	483
$\mathbf{1^{Cs}}$	1	8	98	734	-	-	-	7	97	835

HP of 2 with [Cs][B(C_6F_5)$_4$]: In a Young-NMR-Tube $\mathbf{1^{Cs}}$ (10 mg, 17 μmol, 1 eq.) was dissolved in THF (0.4 mL) and C_6D_6 (0.1 mL). Subsequently, [Ph_3C][B(C_6F_5)$_4$] (16 mg, 17 μmol, 1 eq.) was added resulting in a colour change from orange to colourless. To this solution Ph_2PH (59.8 μL, 343 μmol, 20 eq.) and $Ph_2C{=}CH_2$ (**2**, 60.7 μL, 343 μmol, 20 eq.) were added and NMR spectra were recorded after 20 minutes and 3 hours showing no conversion of the substrates.

HP of 2 with [^{n}Bu$_4$N][PPh$_2$]: In a Young-NMR-Tube **[^{n}Bu$_4$N][PPh$_2$]** (10 mg, 23 μmol, 1 eq.) was dissolved in C_6D_6 (0.5 mL). Subsequently Ph_2PH (81.4 μL, 468 μmol, 20 eq.) and $Ph_2C{=}CH_2$ (**2**, 82.6 μL, 468 μmol, 20 eq.) were added and NMR spectra were recorded after 20 minutes, 1 hour and 20 hours showing less than 1 % conversion of the substrates.

Synthesis of [^{n}Bu$_4$N][PPh$_2$]: To a dark red solution of $NaPPh_2$ (250 mg, 1.20 mmol, 1 eq.) in THF (10 mL) ^{n}Bu$_4$NCl (334 mg, 1.2 mmol, 1 eq.) was added as a solid. The resulting mixture was stirred overnight whereupon the colour brightens. After the mixture was syringe filtered and layered with hexane (10 mL) colourless crystals began to form which were identified as ^{n}Bu$_4$NCl. Removing the solvent from the mother liquor under reduced pressure yielded [^{n}Bu$_4$N][PPh$_2$] (200 mg, 0.47 mmol, 39 %) as a highly viscous off-white oil. ^{1}H NMR (400 MHz, 300 K, C_6D_6, ppm): δ = 7.44 - 7.37 (m, H_{Ar} 4H), 7.13 - 7.03 (m, H_{Ar}, 6H), 2.37 (m, CH_2, 6H), 1.93 (m, CH_2, 2H), 1.38 (m, CH_2, 16H), 0.92 (t, $^1J_{HH}$ = 7.2 Hz, CH_3, 8H), 0.77 (t, $^1J_{HH}$ = 7.2 Hz, CH_3, 4H). ^{31}P NMR (121 MHz, 300 K, C_6D_6, ppm): δ = -16.3 (s).

Synthesis of ^{n}Bu$_2$PNEt$_2$: Under a nitrogen atmosphere PCl_3 (8.75 mL, 100 mmol, 1 eq.) was dissolved in Et_2O (200 mL) and cooled to 0 °C with an ice bath. Subsequently Et_2NH (20.70 mL, 200 mmol, 2 eq.) was added dropwise via syringe whereupon a colourless solid formed. After the reaction mixture was refluxed overnight the colourless suspension was filtered via cannula and the remaining solids were washed twice with together 90 mL Et_2O resulting in a colourless solution of Et_2NPCl_2 which was used in the next step as obtained. To this ^{n}BuLi (1.6 M, 100 mL, 160 mmol, 1.6 eq.) was added at -78 °C via a thin cannula over the course of 70 min resulting in a colourless suspension. The progress of the reaction was monitored by ^{31}P NMR spectroscopy. After the reaction mixture was warmed to room temperature it was cannula filtered, and the volume was reduced to approx. 60 mL under reduced pressure. The yellowish solution was transferred into a smaller Schlenk flask, was dried in vacuo and distilled in dynamic vacuum at 80 °C yielding $Et_2NP^nBu_2$ as a colourless oil (17.21 g, 79 mmol, 79 %). ^{1}H NMR (400 MHz, 300 K, C_6D_6, ppm): δ = 2.87 (m, NCH_2CH_3, 4H), 1.49 (m, $P(CH_2)_3CH_3$, 12H), 0.98 (t, $^3J_{HH}$ = 7.1

Hz, NCH_2CH_3, 6H), 0.93 (t, $^3J_{HH}$ = 7.2 Hz, $P(CH_2)_3CH_3$, 6H). $^{13}C\{^1H\}$ NMR (101 MHz, C_6D_6) δ = 43.28 (d, J = 14.3 Hz), 30.36 (d, J = 14.9 Hz), 28.48 (d, J = 16.1 Hz), 24.81 (d, J = 12.3 Hz), 15.75 (d, J = 3.1 Hz), 14.24. ^{31}P NMR (121 MHz, 300 K, C_6D_6, ppm): δ = 53.3 (s).

Synthesis of ^{n}Bu$_2$PH: The apparatus shown in Figure S1 consists of a dropping funnel on an Erlenmeyer flask containing NaCl (58.44 g, 1000 mmol, 13 eq.) with a gas outlet connected to a $CaCl_2$ drying tube, which led to a gas inlet in a three-necked Schlenk flask, which in turn was connected to two gas washing bottles containing an aqueous KOH solution. The whole apparatus was purged with nitrogen for 45 minutes. The three neck round bottom flask wash charged with Et_2O (500 mL) and $Et_2NP^nBu_2$ (17.21 g, 79 mmol, 1 eq.) and cooled to 0 °C. Then H_2SO_4 (96 %, 77.48 g, 42 mL, 790 mmol, 10 eq.) was degassed by bubbling through N_2 and added to the dropping funnel. While the solution in the Schlenk flask was vigorously stirred H_2SO_4 was slowly added to the NaCl generating gaseous HCl which was bubbled through the reaction mixture with a gentle N_2 flow applied on top of the dropping funnel. The reaction mixture becomes cloudy resulting in a thick colourless suspension. After complete HCl addition the reaction was stirred at 0 °C for additional 30 min. The completeness of the reaction was verified by NMR spectroscopy. While the reaction mixture was allowed to reach room temperature N_2 was bubbled through to degas the excess HCl. The reaction mixture was cannula filtered and the remaining solids were washed with Et_2O (2 x 100 mL) and the combined filtrates were degassed again by 10 alternating cycles of vacuum and N_2. This colourless solution was cooled to 0 °C and $LiAlH_4$ (900 mg, 23 mmol, 0.3 eq.) dissolved in Et_2O (20 mL) and carefully added dropwise resulting in a vigorous reaction. After the reaction was stirred overnight at room temperature a NMR spectrum was measured and a conversion of 90 % of the ^{n}Bu$_2$PCl observed. After the addition of further $LiAlH_4$ (90 mg, 2.38 mmol, 0.03 eq.) in Et_2O (5 mL) at room temperature and stirring for 1 hour another NMR proofed full consumption of the starting materials. The volume of the reaction mixture was reduced to approx. 80 mL by distillation and it was transferred into a smaller Schlenk tube. Further fractionate distillation at ambient pressure yielded ^{n}Bu$_2$PH (5.34 g, 29 mmol 37 %) as a colourless liquid. More products can be obtained by further vacuum distillation of the residues. The obtained analytical data of the isolated product matches the previously reported data.[1]

[1] G. Baccolini, C. Boga, M. Mazzacurati, F. Sangirardi, *High Atom-Economical One-Pot Synthesis of Secondary Phosphines and Their Borane Complexes Using Recycling Phosphorus Donor Reagent, Org. Lett.* **2006**, *8*, 1677-1680.

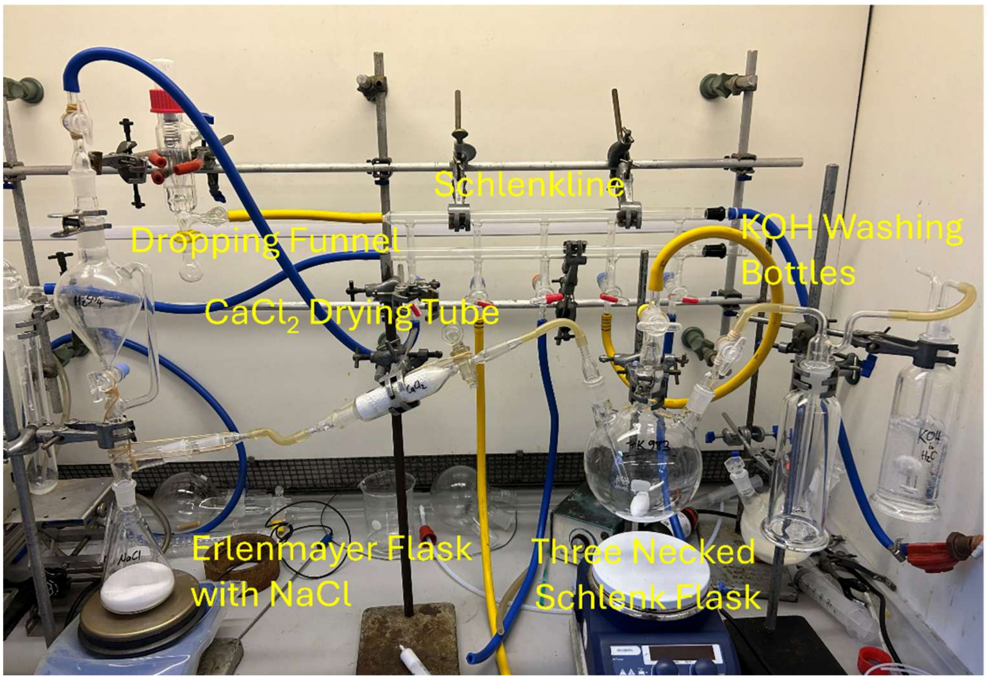

Figure S1: Apparatus for the synthesis of $^nBu_2PCl$.

Isolation of phosphine 2b: Three of the NMR scale HP reactions of ^{t}BuPhPH with **2** in C_6D_6 were combined and layered with hexane (6 mL) resulting in the formation of a greenish solution with brown solids. After syringe filtering the solvent was removed under reduced pressure and the resulting oil was dried in vacuo with a warm water bath. Upon storing the oily residue at room temperature, it slowly solidifies forming crystals suitable for SC-XRD analysis. After the addition of hexane (5 mL), syringe filter, storing at -30 °C, removing the supernatant and drying in vacuo the product was isolated as a colourless solid (312 mg, 0.90 mmol, 60 %). ^{1}H NMR (400 MHz, 300 K, C_6D_6, ppm): δ = 7.49 - 7.43 (m, H_{Ar} 2H), 7.22 - 7.17 (m, H_{Ar}, 4H), 7.15 - 7.06 (m, H_{Ar}, overlap with C_6D_6, 7H), 7.04 – 6.96 (m, H_{Ar}, 2H), 4.07 (m, $Ph_2C\mathit{H}$-CH_2-P^{t}BuPh, 1H), 2.27 (m, Ph_2CH-$C\mathit{H_2}$-P^{t}BuPh, 1H), 2.44 (m, Ph_2CH-$C\mathit{H_2}$-P^{t}BuPh, 1H), 0.92 (d, $^3J_{PH}$ = 11.7 Hz, H_{tBu}, 9H). ^{31}P NMR (121 MHz, 300 K, C_6D_6, ppm): δ = -1.2 (s).

Isolation of phosphine 2c: Two of the NMR scale HP reactions of ^{t}Bu$_2$PH with **2** in C_6D_6 were combined and layered with hexane (6 mL) resulting in the formation of a greenish solution with brown solids. After syringe filtering the solvent was removed under reduced pressure and the resulting dark red oil was dried in vacuo with a warm water bath. The oil was extracted with pentane (5 mL), syringe filtered and the solvent was again evaporated resulting in a yellowish oil. A second extraction with hot hexane (5 mL) and storing the resulting solution at -30 °C leads to the formation of colourless crystals suitable for SC-XRD which were identified as the oxide **2c-O**. The colourless solid was isolated yielding **2c-O** (85 mg, 0.25 mmol, 25 %) contaminated with **2c**. The solvent of the mother liquor was removed in vacuo yielding **2c** (140 mg, 0.43 mmol, 43 %) as a colourless oil contaminated with **2c-O**. **2c**: ^{1}H NMR (400 MHz, 300 K, C_6D_6, ppm): δ = 7.33 - 7.28 (m, H_{Ar} 4H), 7.15 - 7.11 (m, H_{Ar}, overlap with C_6D_6, 4H), 7.05 - 6.99 (m, H_{Ar}, 2H), 4.17 (m, $Ph_2C\mathit{H}$-CH_2-P^{t}Bu$_2$, 1H), 2.13 (m, overlap with C_6Me_6 Ph_2CH-$C\mathit{H_2}$-P^{t}Bu$_2$, 2H), 1.00 (d, $^3J_{PH}$ = 10.7 Hz, H_{tBu}, 18H). ^{31}P NMR (121 MHz, 300 K, C_6D_6, ppm): δ = 21.0 (s). **2c-O**: ^{1}H NMR (400 MHz, 300 K, C_6D_6, ppm): δ = 7.47 - 7.42 (m, H_{Ar} 4H), 7.15 - 7.10 (m, H_{Ar}, 4H), 7.04 - 6.97 (m, H_{Ar},

2H), 5.00 (m, Ph_2CH-CH_2-P(O)^tBu_2, 1H), 2.35 (m, Ph_2CH-CH_2-P(O)^tBu_2, 2H), 0.96 (d, $^3J_{PH}$ = 12.9 Hz, H_{tBu}, 18H). ^{31}P NMR (121 MHz, 300 K, C_6D_6, ppm): δ = 57.1 (s).

Isolation of phosphine 2d: Two of the NMR scale HP reactions of ^{n}Bu_2PH with **2** in C_6D_6 were combined and layered with hexane (6 mL) resulting in the formation of a greenish solution with yellowish-brown solids. After syringe filtering the solvent was removed under reduced pressure and the resulting oil was dried in vacuo with a warm water bath. Upon storing the oily residue at room temperature, it slowly solidifies forming crystals which were identified as C_6Me_6. After the addition of hexane (2 mL), syringe filter, removing the solvent and drying in vacuo the product was isolated as a colourless oil (275 mg, 0.84 mmol, 84 %) with traces of C_6Me_6 and **2**. ^{1}H NMR (400 MHz, 300 K, C_6D_6, ppm): δ = 7.28 - 7.23 (m, H_{Ar} 4H), 7.15 - 7.10 (m, H_{Ar}, overlap with C_6D_6, 4H), 7.06 - 7.00 (m, H_{Ar}, 2H), 4.20 (m, Ph_2CH-CH_2-P^nBu_2, 1H), 2.13 (m, Ph_2CH-CH_2-P^nBu_2, 2H), 1.28 (m, ^{n}Bu-CH_2, 12H) 0.84 (t, $^3J_{HH}$ = 7.1 Hz, ^{n}Bu-CH_3, 6H). ^{31}P NMR (121 MHz, 300 K, C_6D_6, ppm): δ = -32.4 (s).

Synthesis of bisphosphines 18a-d: In a Young-NMR-Tube BnCs (4 mg, 18 μmol, 1 eq.) was treated with C_6D_6 (0.5 mL) which contained 18-crown-6 (5.2 mg, 20 μmol, 1.1 eq.) resulting in a dark red solution. Subsequently the phosphine (393 μmol, 22 eq.; **a**: 68.4 μL, **b**: 70.6 μL, **c**: 74.5 μL, **d**: 80.3 μL) and Ph_2P(vinyl) (70.8 μL, 357 μmol, 20 eq.) were added and the mixture was shaken vigorously for 2 min resulting in a bright orange solution. NMR spectra were recorded after 30 min revealing full conversion for the phosphines **a**, **b** and **d**. With phosphine **c** full conversion was reached after 4 hours at room temperature. Toluene (2 mL) and Phenol was added in small portions until the mixture becomes colourless and a solid formed.

Isolation of 18a: The quenched mixture was syringe filtered and the volume was reduced to approx. 1 mL whereupon the formation of a crystalline material was observed. Heating the mixture until everything was dissolved again and storing at room temperature led to the formation of huge colourless block shaped crystals. To this pentane (1 mL) was added and after storing at -30 °C overnight, removing the mother liquor and drying the crystals in vacuo yielded **18a** (111 mg, 278 μmol, 78 %) in form of colourless crystals. The obtained analytical data of the isolated product matches published data of DPPE.

Isolation of 18b: The quenched mixture was syringe filtered and the volume was reduced to approx. 0.5 mL. Extraction with hot hexane (3 mL), syringe filter and evaporation of the solvent yielded a colourless oil which was recollected in hexane (0.5 mL) and cooled to -30 °C whereupon a colourless solid and crystalline material formed. The crystals where suitable for SC-XRD analysis and were identified as **18b**. removing the mother liquor and drying in vacuo yielded **18b** (93 mg, 246 μmol, 69 %) in form of a colourless solid. ^{1}H NMR (400 MHz, 300 K, C_6D_6, ppm): δ = 7.42 - 7.31 (m, H_{Ar}, 6H), 7.14 - 7.08 (m, H_{Ar}, 3H), 7.07 - 6.98 (m, H_{Ar}, 6H), 2.28 (m, Ph_2P-(CH_2)-P^{t}BuPh, 1H), 2.09 (m, Ph_2P-(CH_2)-P^{t}BuPh, 2H), 1.80 (m, Ph_2P-(CH_2)-P^{t}BuPh, 1H), 0.85 (d, $^3J_{PH}$ = 11.7 Hz, H_{tBu}, 9H). ^{31}P NMR (121 MHz, 300 K, C_6D_6, ppm): δ = 7.38 (d, $^1J_{PP}$ = 32.2 Hz, P^tBuPh), -12.1 (d, $^1J_{PP}$ = 32.2 Hz, PPh_2).

Isolation of 18c and d: The quenched mixtures were syringe filtered and the volume was reduced to approx. 0.5 mL. Extraction with hot hexane (3 mL), syringe filter and evaporation of the solvent yielded

18c (60 mg, 142 μmol,47 %) and **18d** (83 mg, 232 μmol, 65 %) as colourless oils. **18c**: ^{1}H NMR (400 MHz, 300 K, C_6D_6, ppm): δ = 7.49 - 7.43 (m, H_{Ar}, 4H), 7.11 - 7.03 (m, H_{Ar}, 6H), 2.37 (m, Ph_2P-(C*H2*)-P^{t}Bu$_2$, 2H), 1.52 (m, Ph_2P-(C*H2*)-P^{t}Bu$_2$, 2H), 0.99 (d, $^{3}J_{PH}$ = 10.7 Hz, H_{tBu}, 18H). ^{31}P NMR (121 MHz, 300 K, C_6D_6, ppm): δ = 34.8 (d, $^{1}J_{PP}$ = 31.9 Hz, *P*^{t}Bu$_2$), -12.8 (d, $^{1}J_{PP}$ = 31.9 Hz, *P*Ph$_2$). **18d**: ^{1}H NMR (400 MHz, 300 K, C_6D_6, ppm): δ = 7.47 - 7.40 (m, H_{Ar}, 4H), 7.12 - 7.02 (m, H_{Ar}, 6H), 2.19 (m, Ph_2P-(C*H2*)-P^{n}Bu$_2$, 2H), 1.52 (m, Ph_2P-(C*H2*)-P^{n}Bu$_2$, 2H), 1.27 (m, ^{n}Bu-C*H2*, 12H) 0.84 (t, $^{3}J_{HH}$ = 7.0 Hz, ^{n}Bu-C*H3*, 6H). ^{31}P NMR (121 MHz, 300 K, C_6D_6, ppm): δ = -12.8 (d, $^{1}J_{PP}$ = 25.4 Hz, *P*Ph$_2$), -26.7 (d, $^{1}J_{PP}$ = 25.4 Hz, *P*^{n}Bu$_2$).

Synthesis of bisphosphines 18e: In a Young-NMR-Tube BnCs (8 mg, 36 μmol, 1 eq.) was suspended in C_6D_6 (1 mL). Subsequently TerPH$_2$ (357 μmol, 124 mg, 10 eq.) and Ph$_2$P(vinyl) (70.8 μL, 357 μmol, 10 eq.) were added and the mixture was shaken vigorously for 2 min. After no conversion was detected in the NMR spectra the mixture was heated to 80 °C for three days and the progress of the reaction was monitored by NMR spectroscopy reaching a plateau at 75 % conversion of the starting materials. To the dark red solution two drops of MeOH were added leading to discolouration. After toluene (2 mL) was added the mixture was syringe filtered and hexane (9 mL) was added. Overnight a precipitate formed which was removed by another syringe filtration. Subsequently the solvent was removed in vacuo yielding a sticky yellowish solid. Recrystallization from methylcyclohexane (5 mL) by slow solvent evaporation yielded **18e** (60 mg, 107 μmol, 30 %) as off-white crystalline solid contaminated with TerPH$_2$ which could not be fully removed by crystallisation. ^{1}H NMR (400 MHz, 300 K, C_6D_6, ppm): δ = 7.17 - 7.09 (m, H_{Ar}, overlap with C_6D_6, 4H), 7.03 - 6.97 (m, H_{Ar}, 6H), 6.93 - 6.89 (m, H_{Ar}, 3H), 6.88 (bs, H_{Ar}, 2H), 6.83 (bs, H_{Ar}, 2H), 4.16 (m, Ph_2P-(C*H2*)-P(H)Ter, 2H), 3.61 (m, Ph_2P-(C*H2*)-P(H)Ter, 2H), 3.26 (d, $^{1}J_{PH}$ = 209.8 Hz, Ph_2P-(CH$_2$)-P(*H*)Ter, 1H), 2.20 (s, C*H3*, 6H), 2.16 (s, C*H3*, 6H), 2.04 (s, C*H3*, 6H). ^{31}P NMR (121 MHz, 300 K, C_6D_6, ppm): δ = -12.4 (d, $^{1}J_{PP}$ = 25.4 Hz, *P*Ph$_2$), -66.7 (d, $^{1}J_{PP}$ = 25.4 Hz, *P*^{n}Bu$_2$).

Section S2 – NMR Spectra of the Isolated Compounds

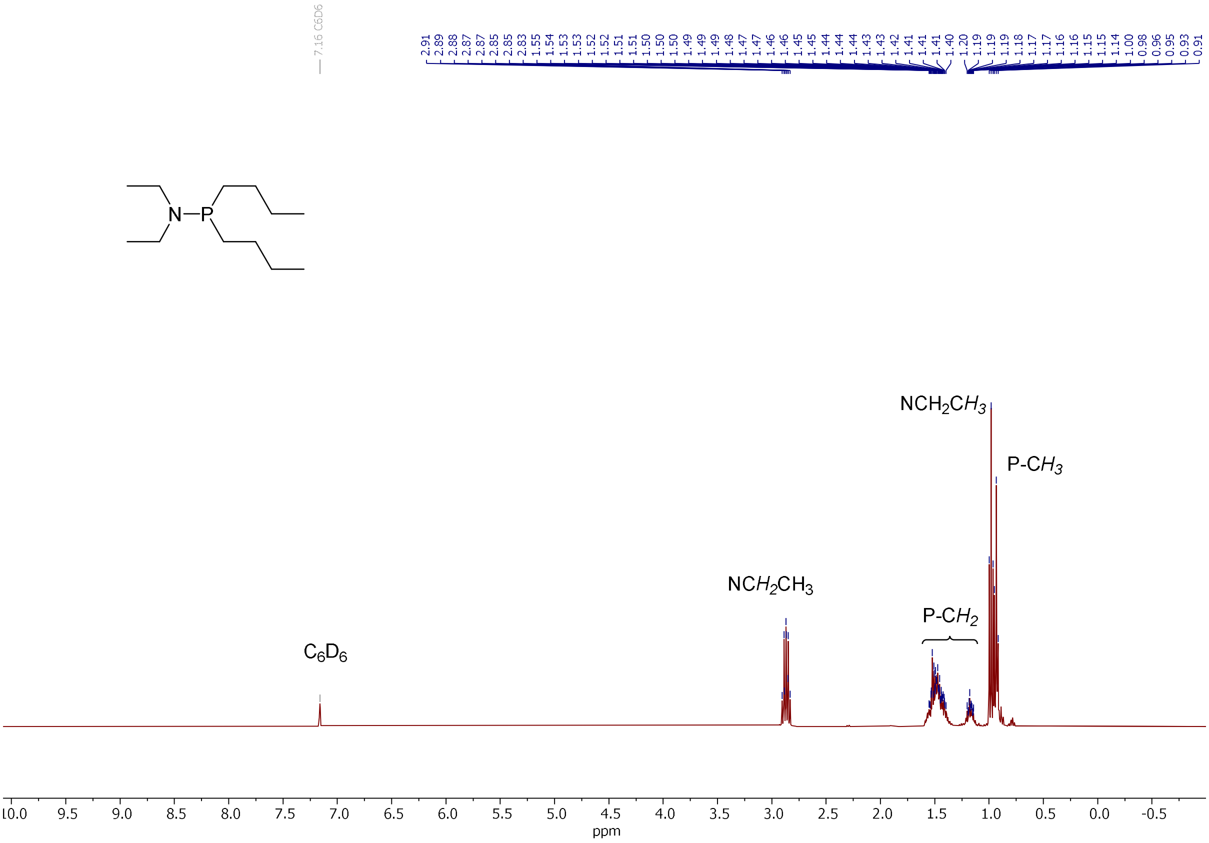

Figure S2. ¹H NMR spectrum of isolated $Et_2NP^nBu_2$ in benzene-d_6 at 300 K.

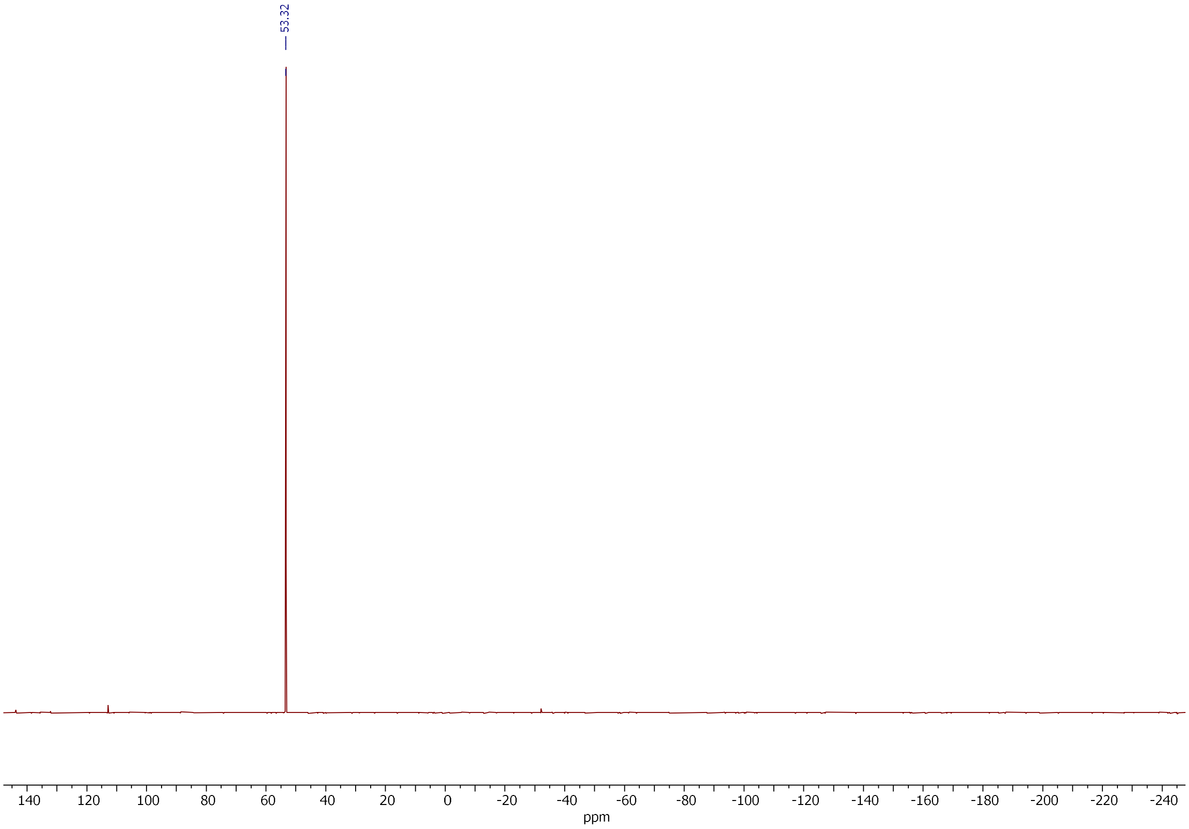

Figure S3. ³¹P{¹H} NMR spectrum of isolated $Et_2NP^nBu_2$ in benzene-d_6 at 300 K.

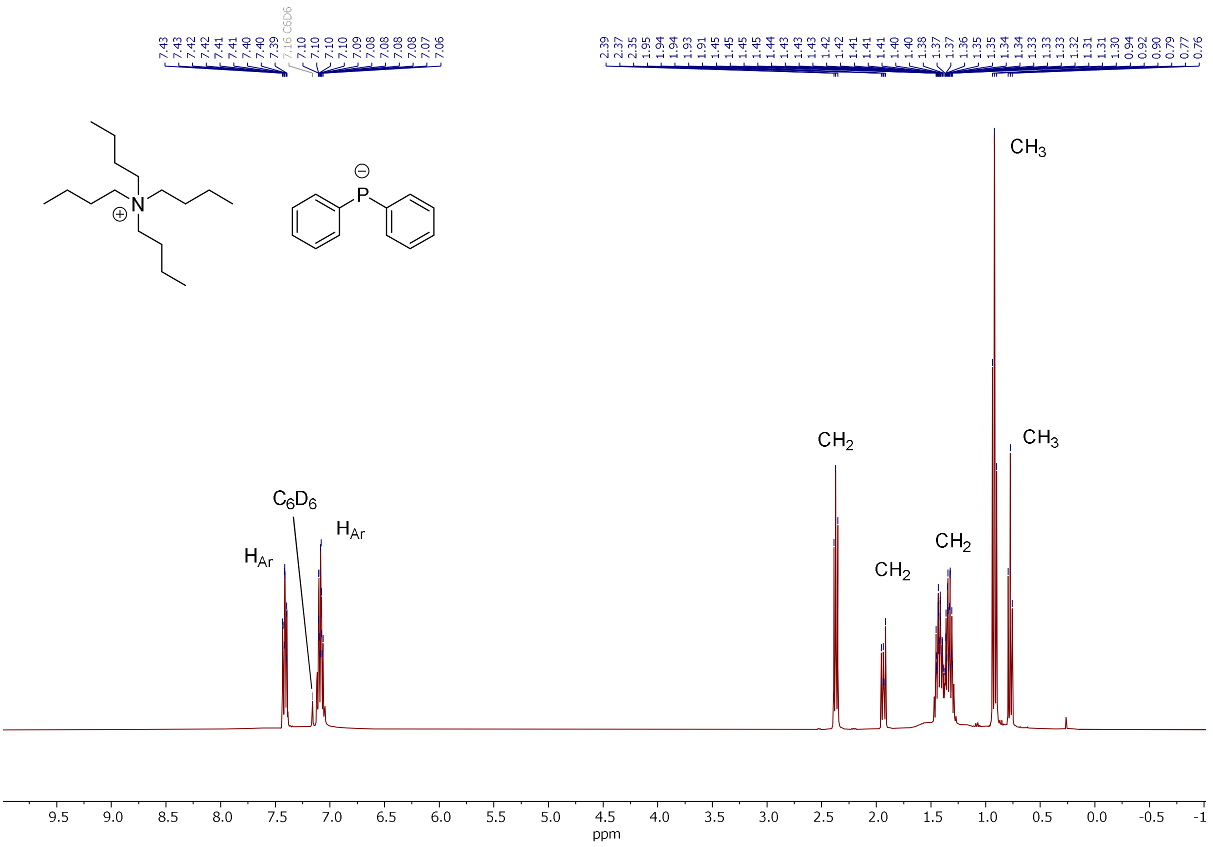

Figure S4. ^{1}H NMR spectrum of isolated [$^{n}Bu_4N$][PPh_2] in benzene-d_6 at 300 K.

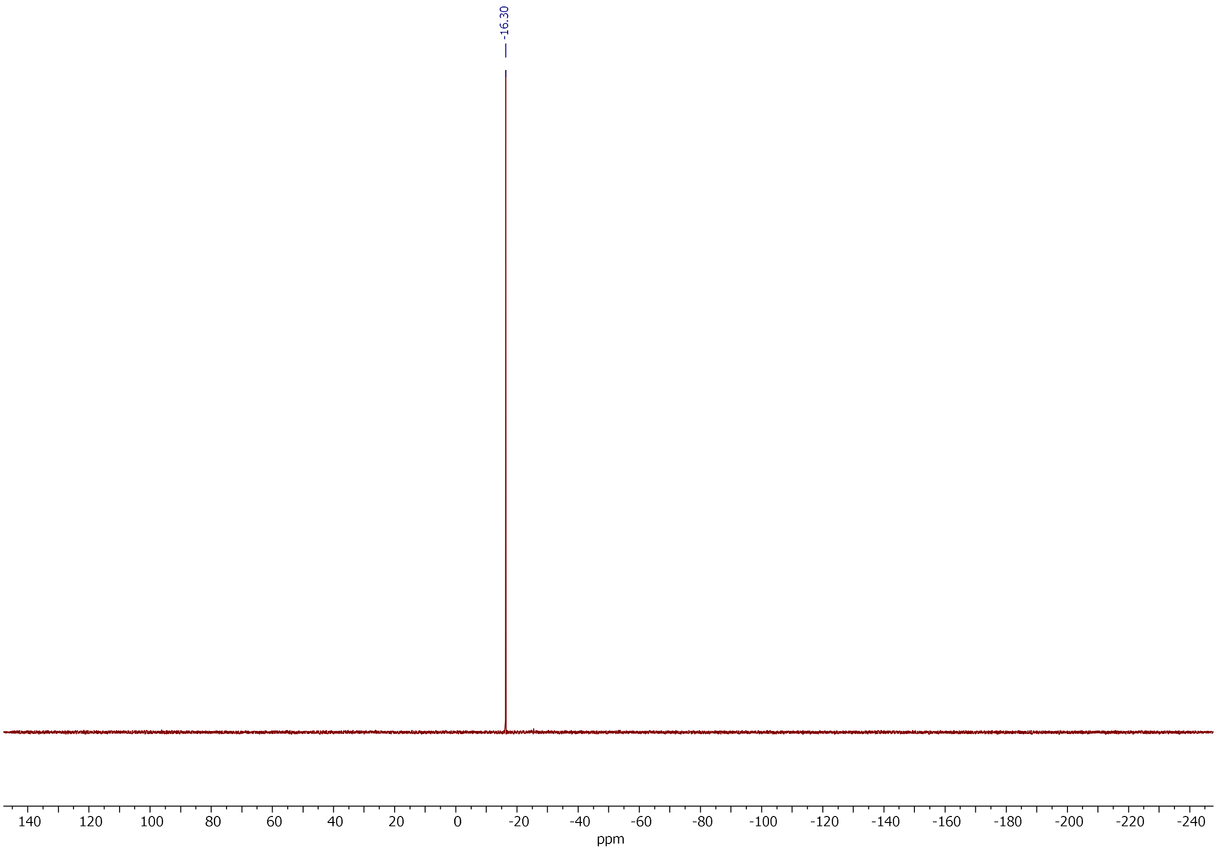

Figure S5. $^{31}P\{^{1}H\}$ NMR spectrum of isolated [$^{n}Bu_4N$][PPh_2] in benzene-d_6 at 300 K.

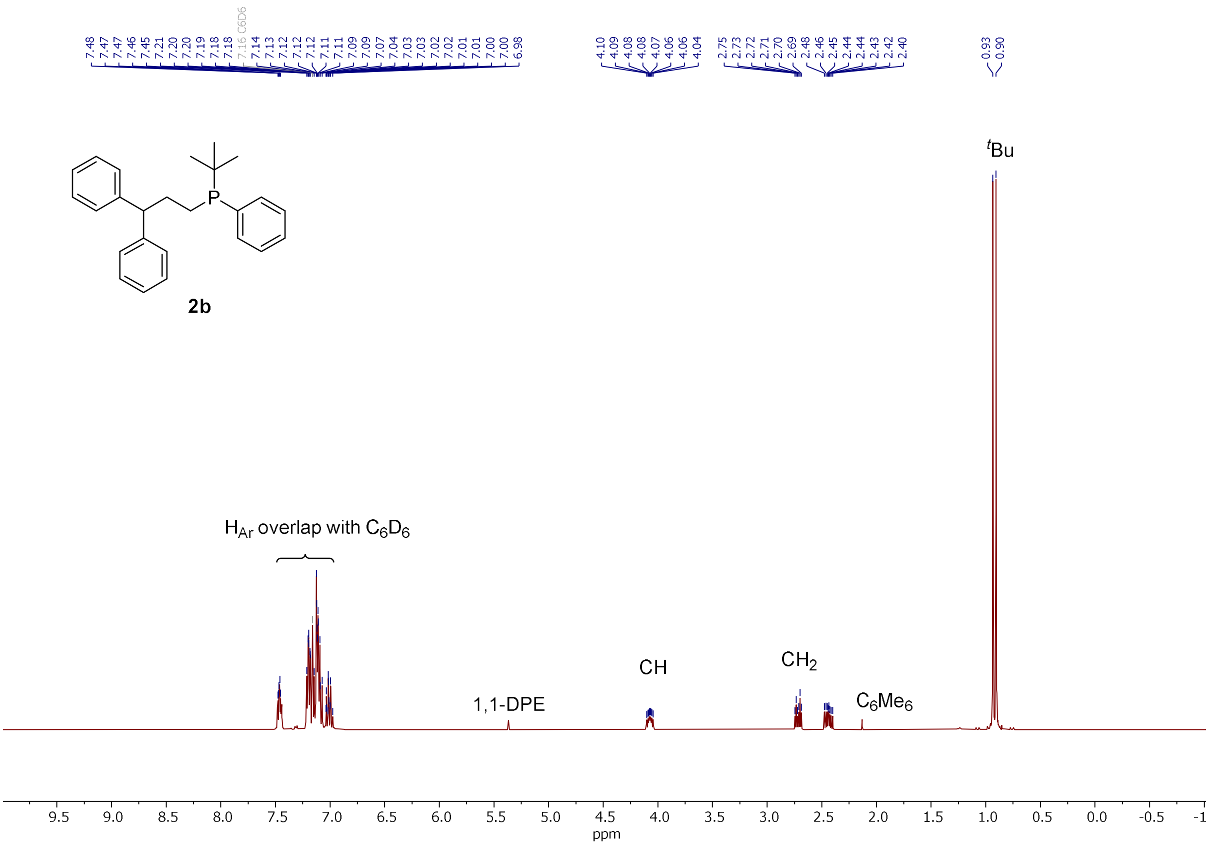

*Figure S6. ^{1}H NMR spectrum of isolated **2b** in benzene-d_6 at 300 K.*

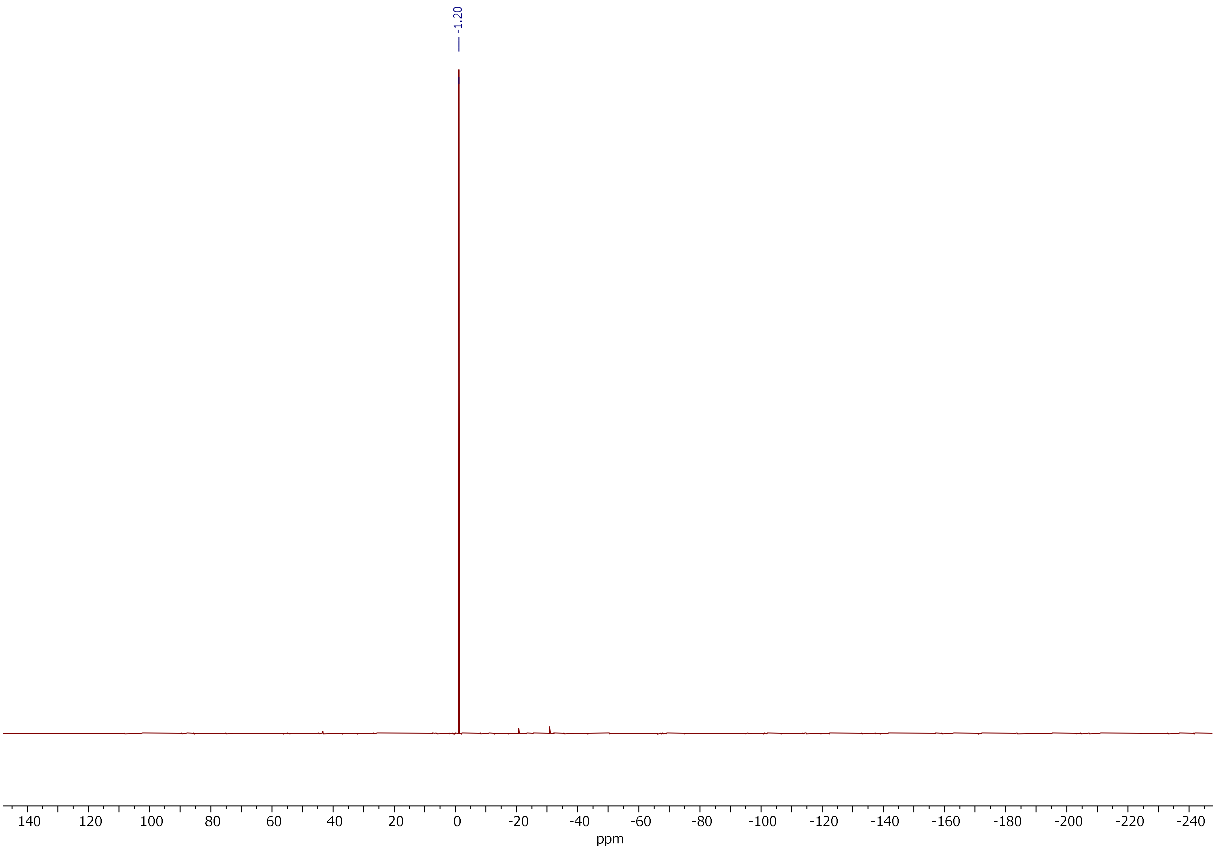

*Figure S7. $^{31}P\{^{1}H\}$ NMR spectrum of isolated **2b** in benzene-d_6 at 300 K.*

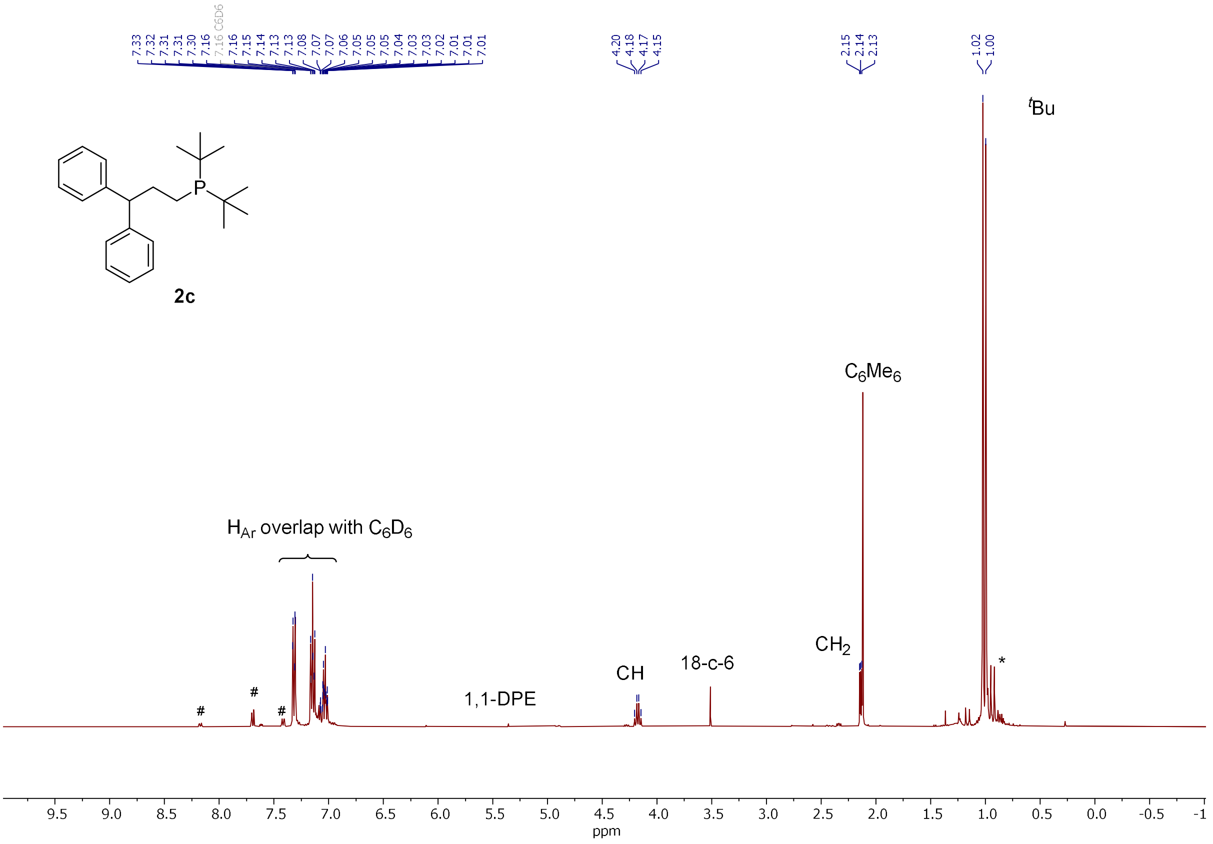

*Figure S8. ^{1}H NMR spectrum of isolated **2c** in benzene-d_6 at 300 K. * belongs to $^{t}Bu_2PH$, # belongs to unknown impurities.*

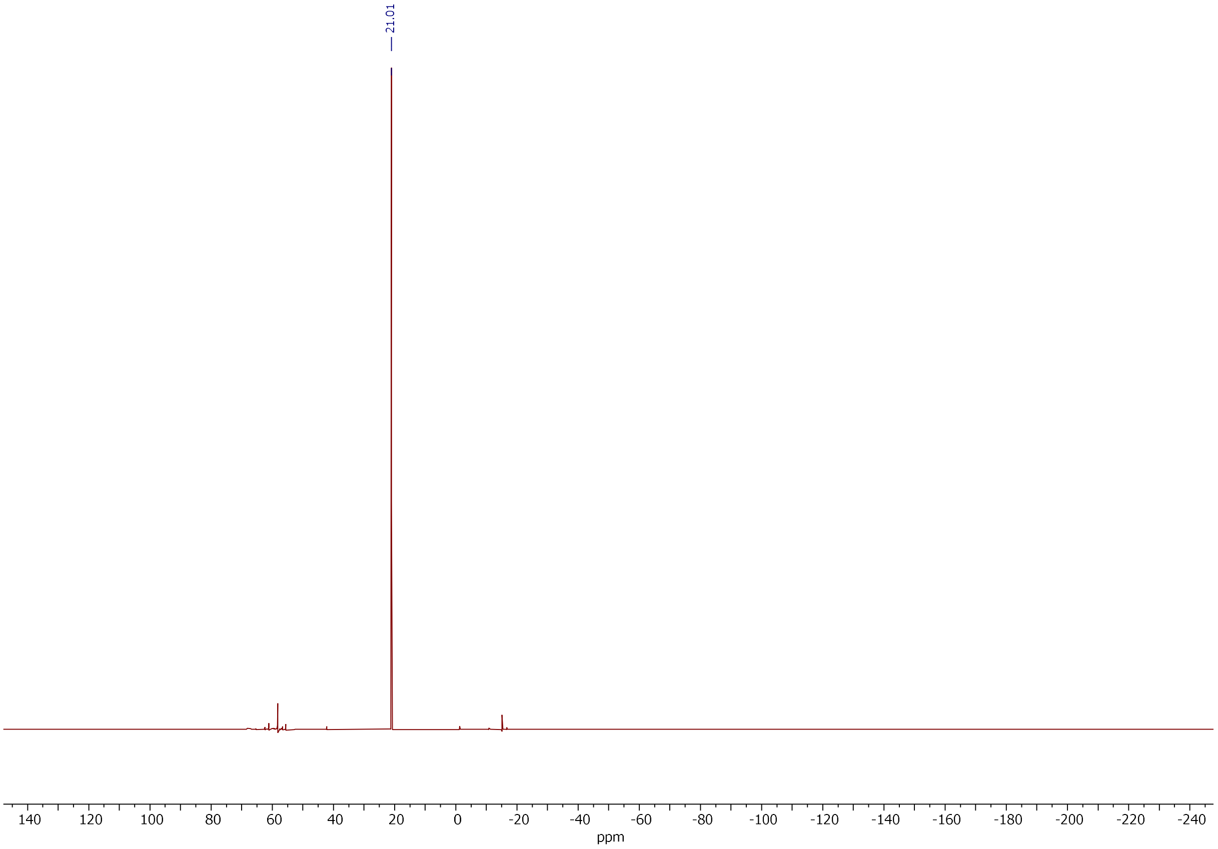

*Figure S9. $^{31}P\{^{1}H\}$ NMR spectrum of isolated **2c** in benzene-d_6 at 300 K. Signal at 59 ppm belongs to 2c-O. Signal at -19 ppm belongs to $^{t}Bu_2PH$.*

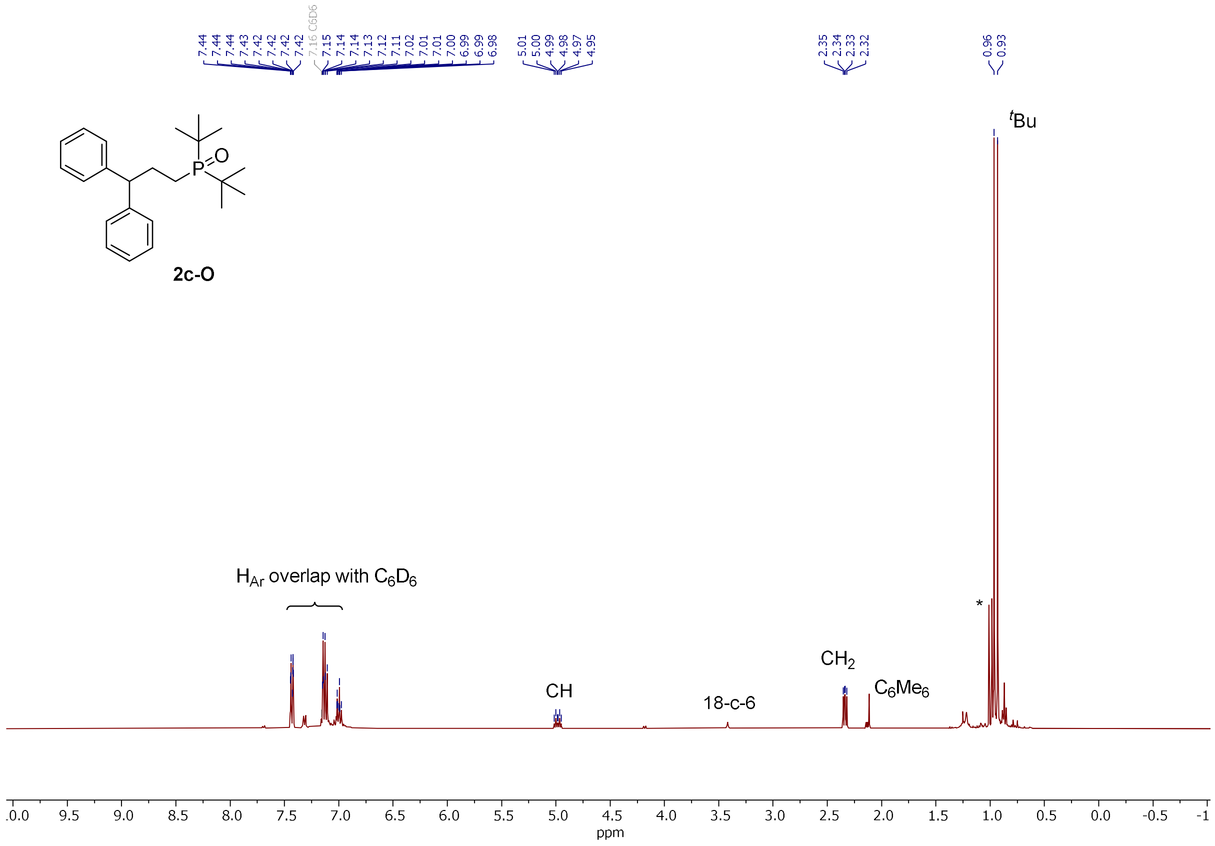

Figure S10. ^{1}H *NMR spectrum of isolated* ***2c-O*** *in benzene-*d_6 *at 300 K. * belongs to* ***2c****.*

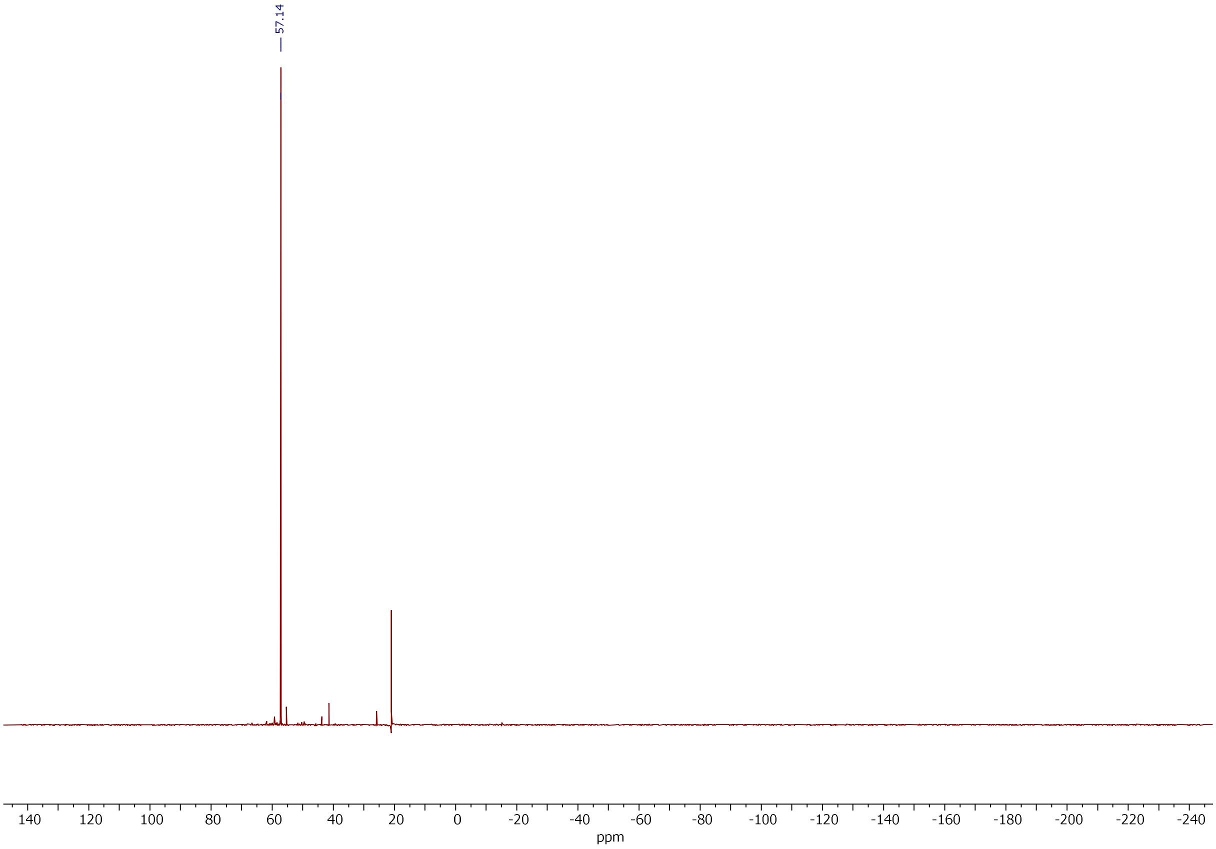

Figure S11. $^{31}P\{^{1}H\}$ *NMR spectrum of isolated* ***2c-O*** *in benzene-*d_6 *at 300 K. Signal at 21 ppm belongs to* ***2c****.*

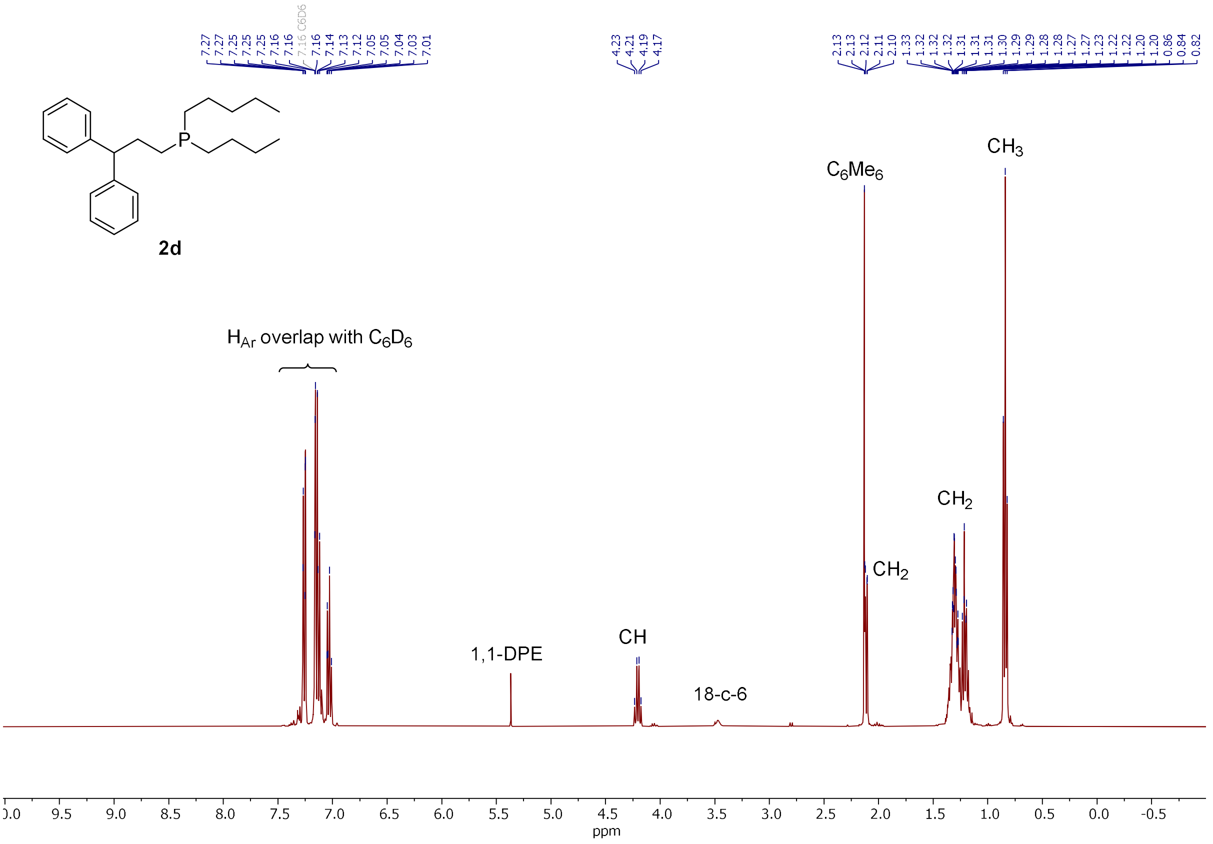

*Figure S12. ^{1}H NMR spectrum of isolated **2d** in benzene-d_6 at 300 K.*

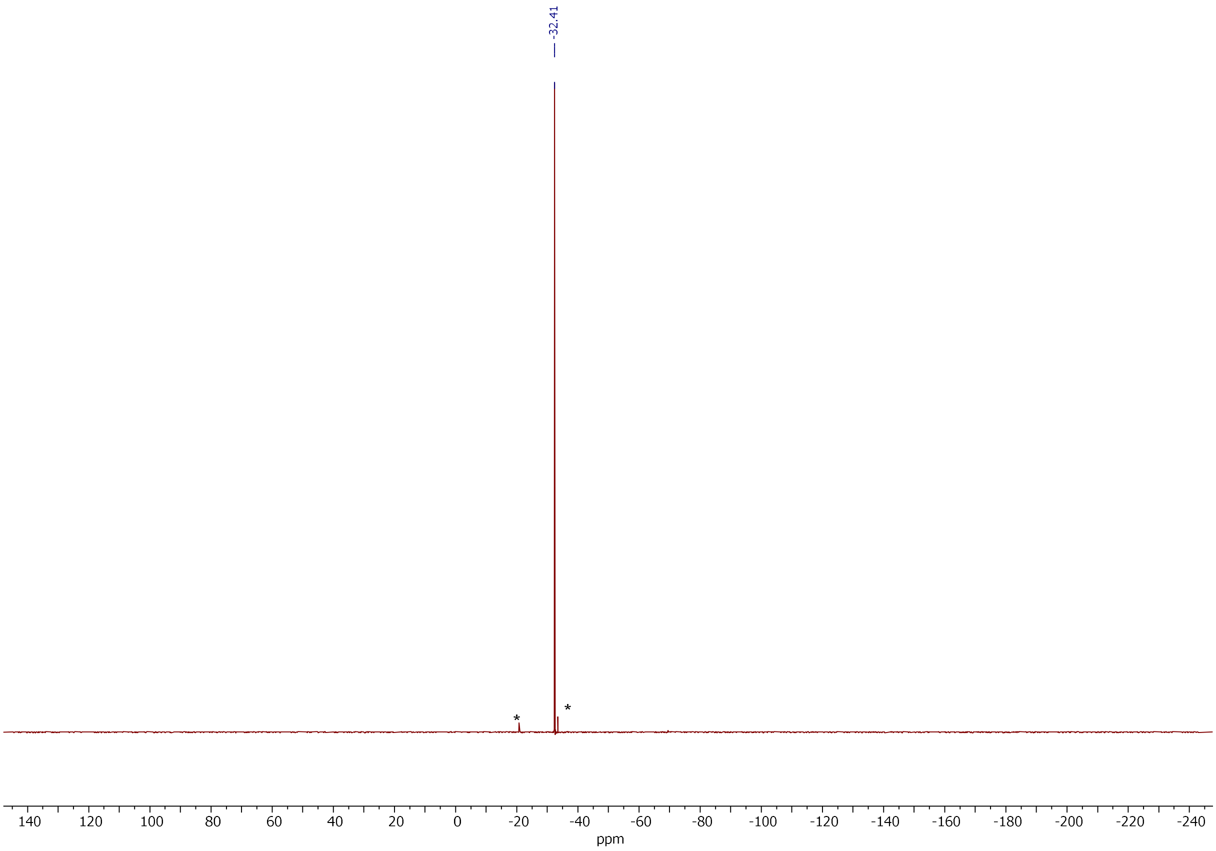

*Figure S13. $^{31}P\{^{1}H\}$ NMR spectrum of isolated **2d** in benzene-d_6 at 300 K. *minor unknown impurities.*

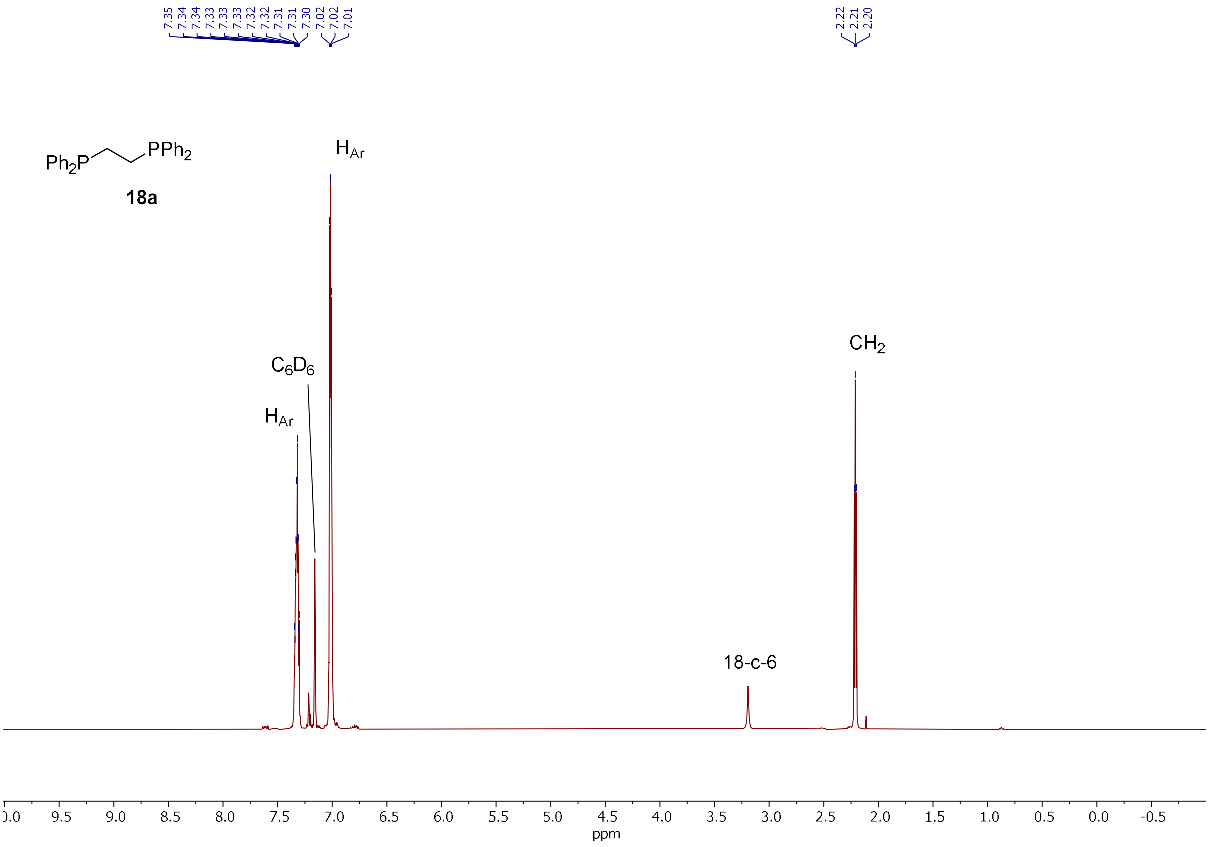

*Figure S14. ^{1}H NMR spectrum of isolated **18a** in benzene-d_6 at 300 K.*

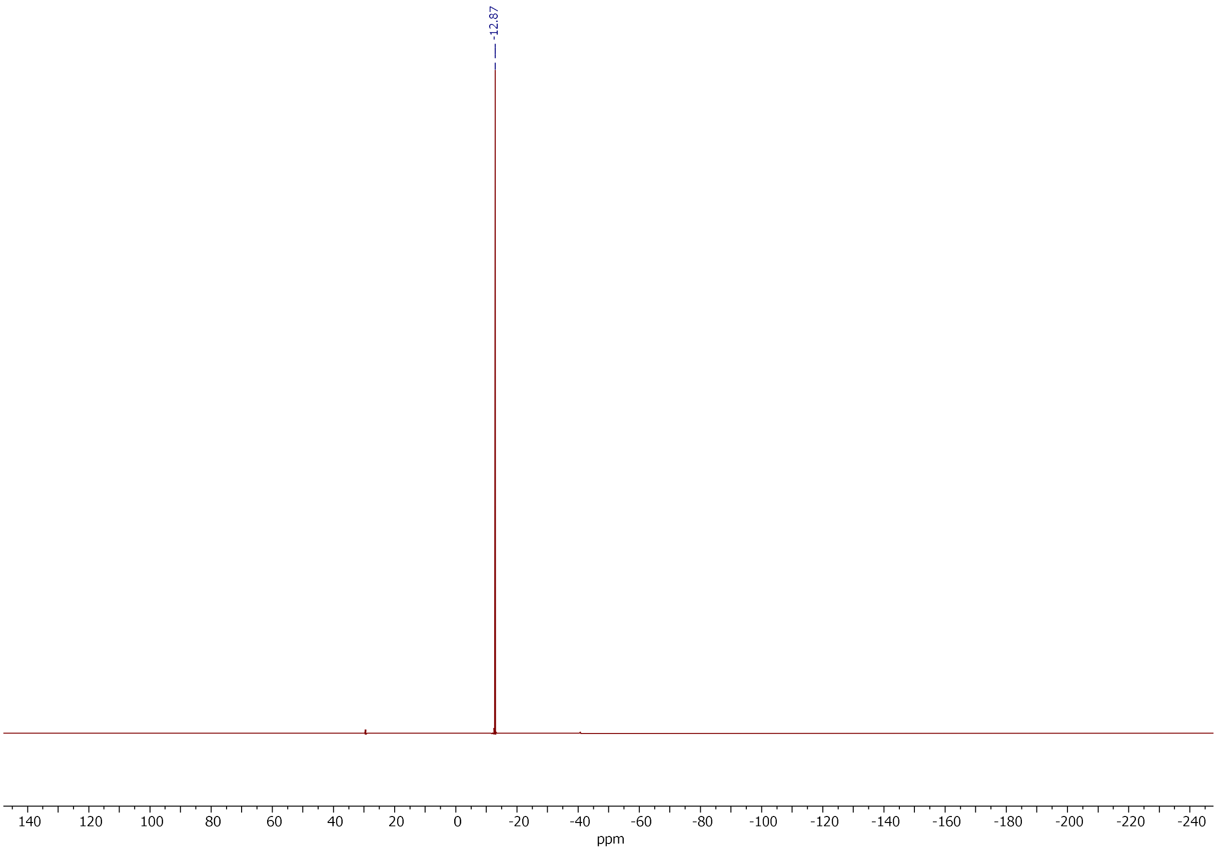

*Figure S15. ^{31}P{^{1}H} NMR spectrum of isolated **18a** in benzene-d_6 at 300 K.*

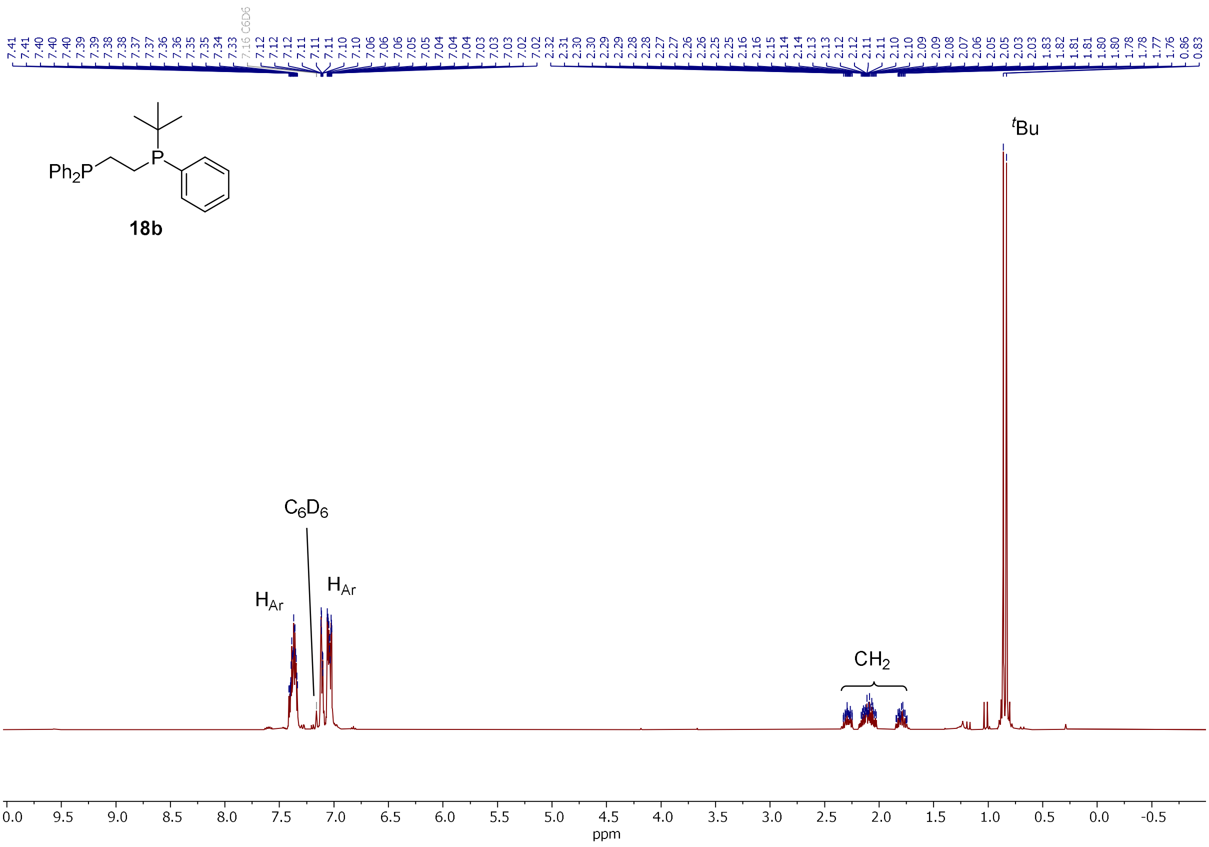

*Figure S16. ^{1}H NMR spectrum of isolated **18b** in benzene-d_6 at 300 K.*

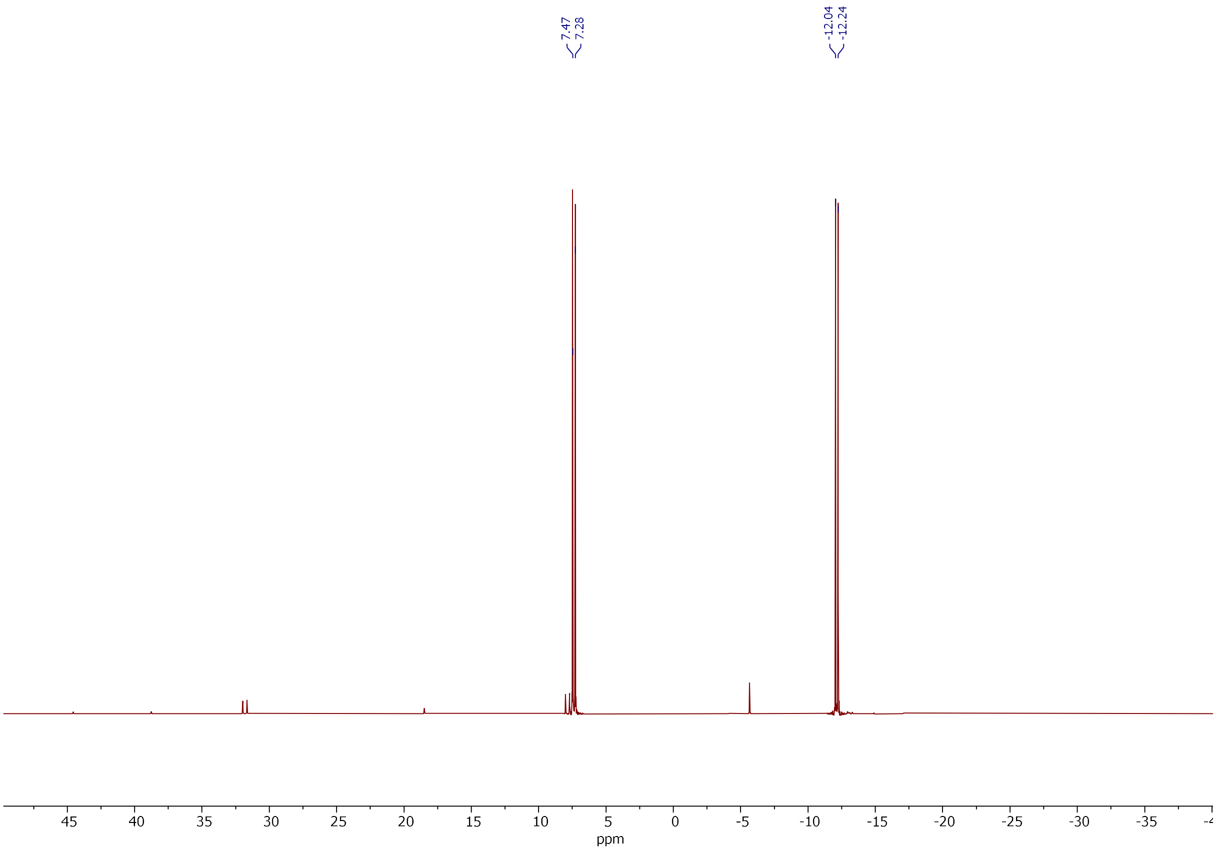

*Figure S17. $^{31}P\{^{1}H\}$ NMR spectrum of isolated **18b** in benzene-d_6 at 300 K. Minor unknown impurities at -6, 18 and 32 ppm.*

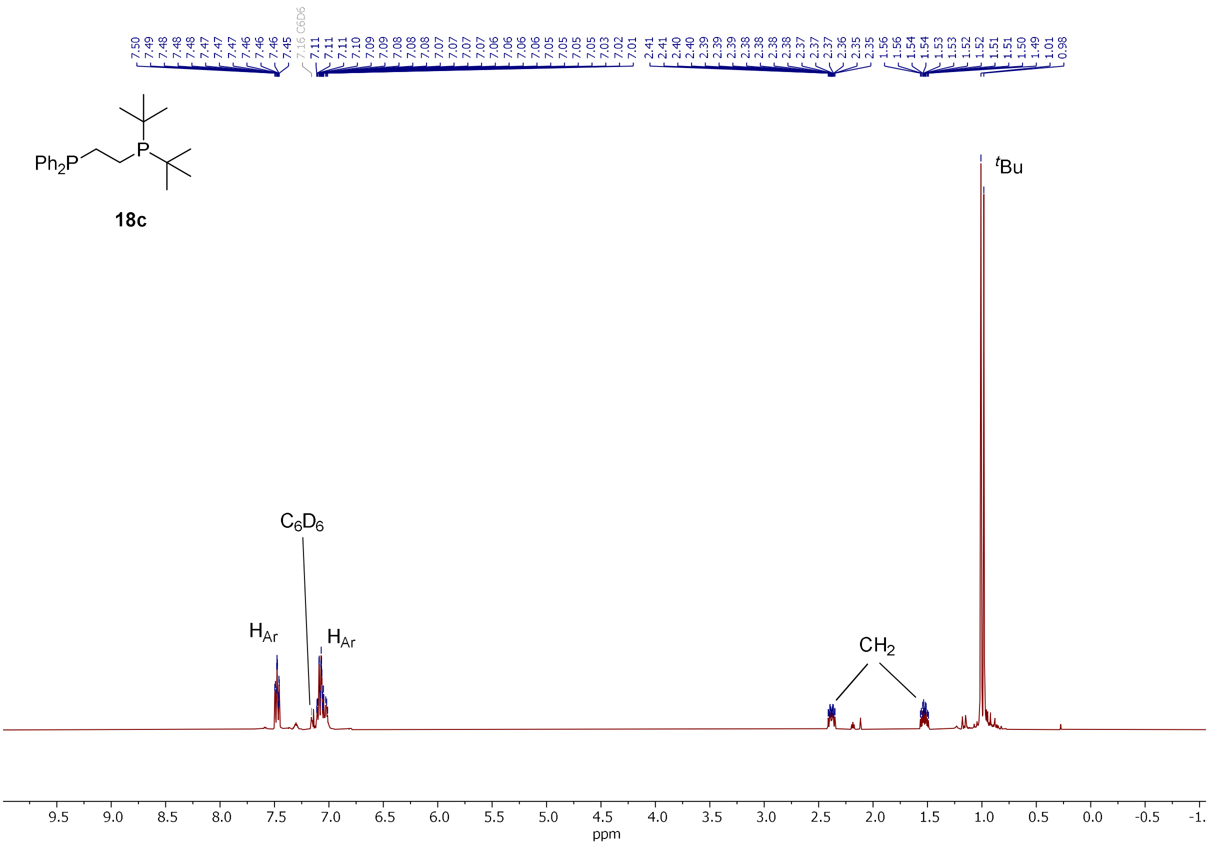

Figure S18. ^{1}H *NMR spectrum of isolated* ***18c*** *in benzene-d*$_6$ *at 300 K. With minor impurities of toluene.*

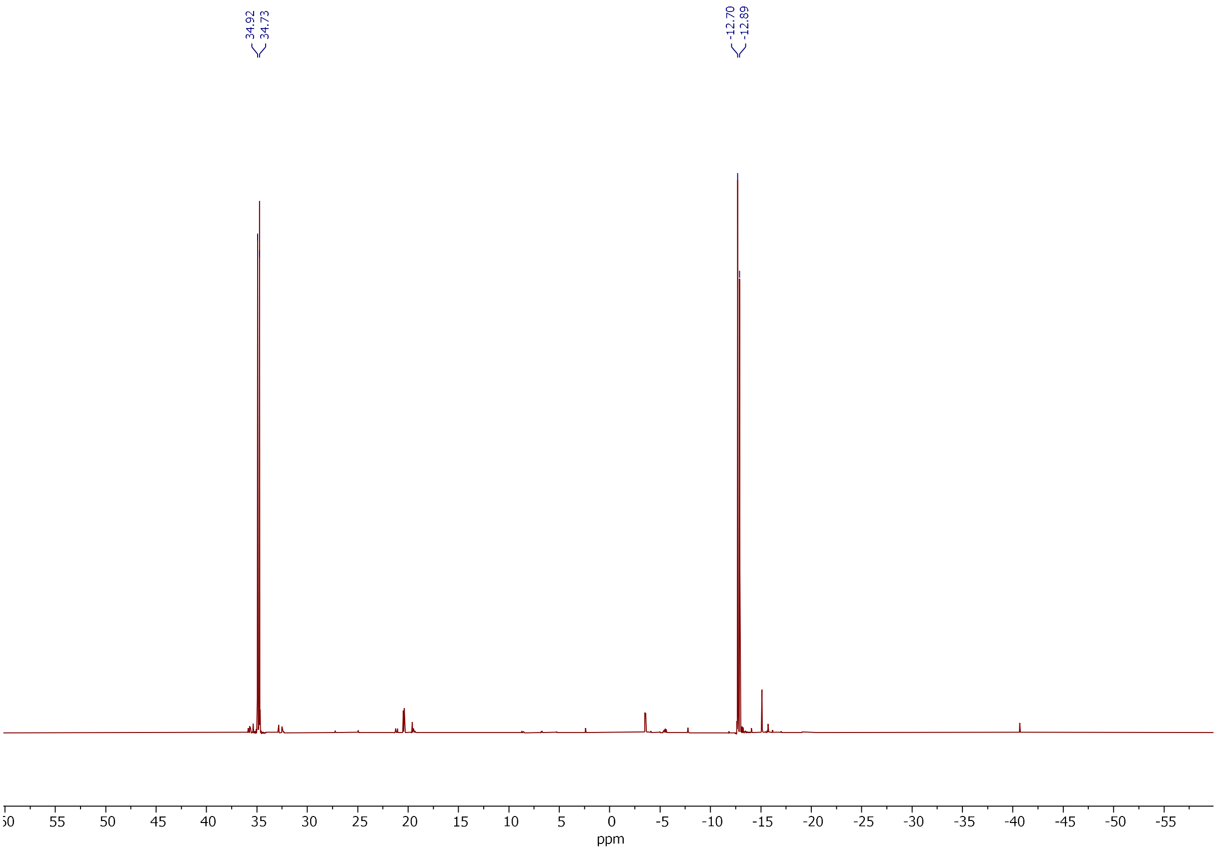

Figure S19. $^{31}P\{^{1}H\}$ *NMR spectrum of isolated* ***18c*** *in benzene-d*$_6$ *at 300 K. Minor unknown impurities.*

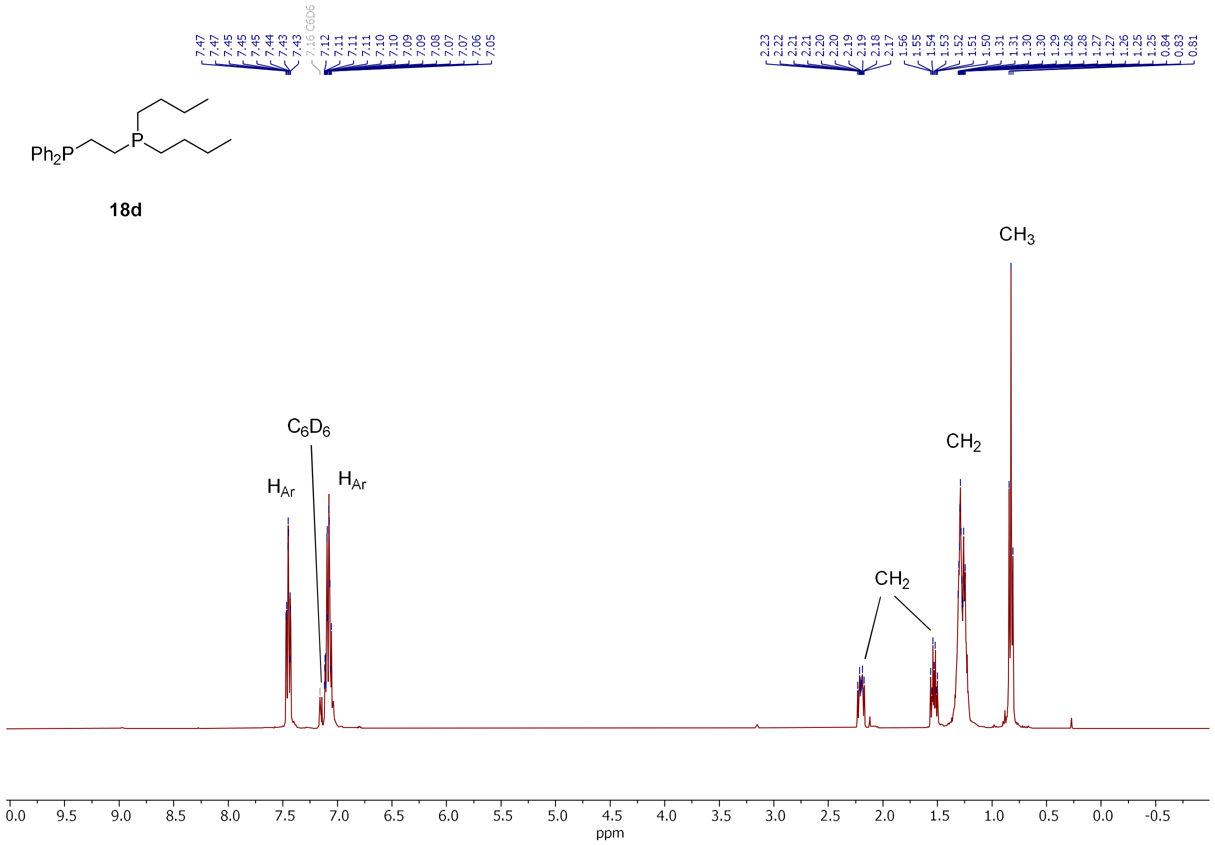

Figure S20. ^{1}*H NMR spectrum of isolated* ***18d*** *in benzene-d*$_6$ *at 300 K. With minor impurities of toluene.*

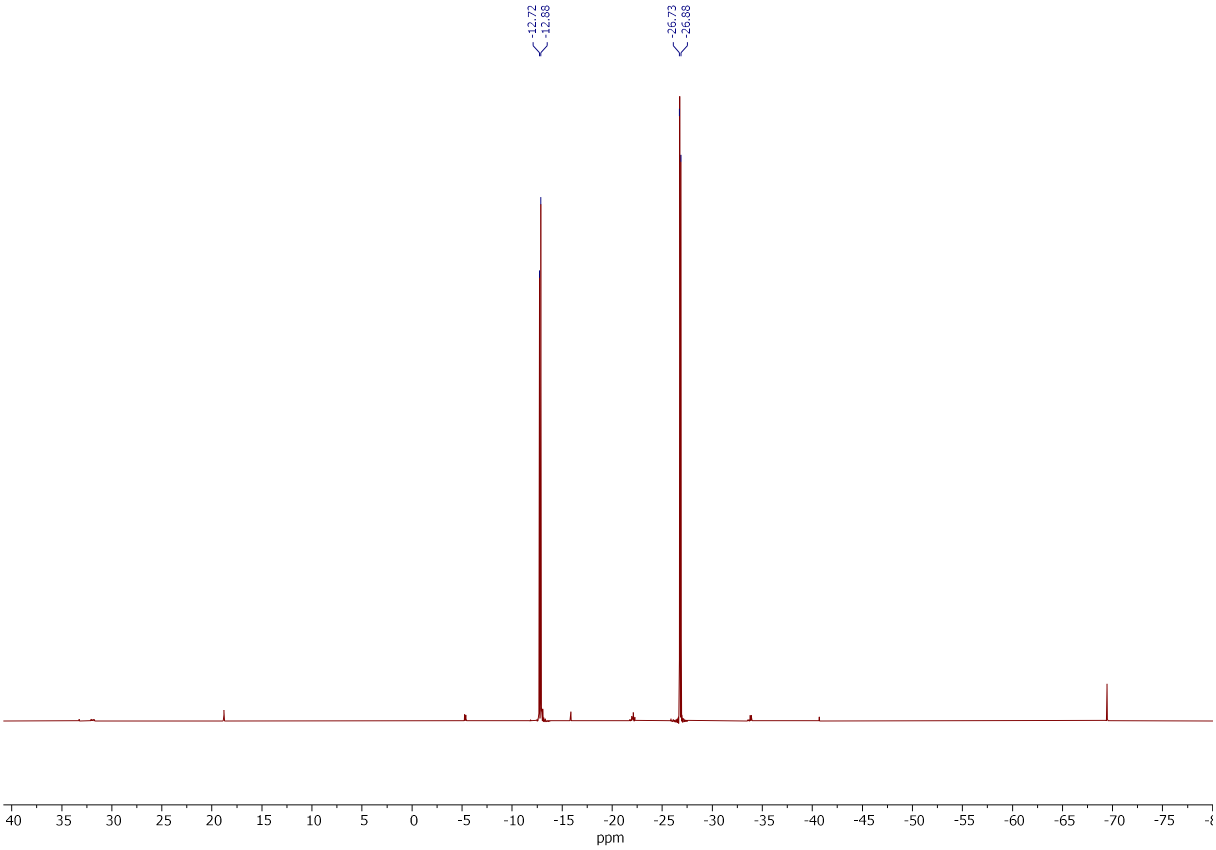

Figure S21. $^{31}P\{^{1}H\}$ *NMR spectrum of isolated* ***18d*** *in benzene-d*$_6$ *at 300 K. Minor unknown impurities.*

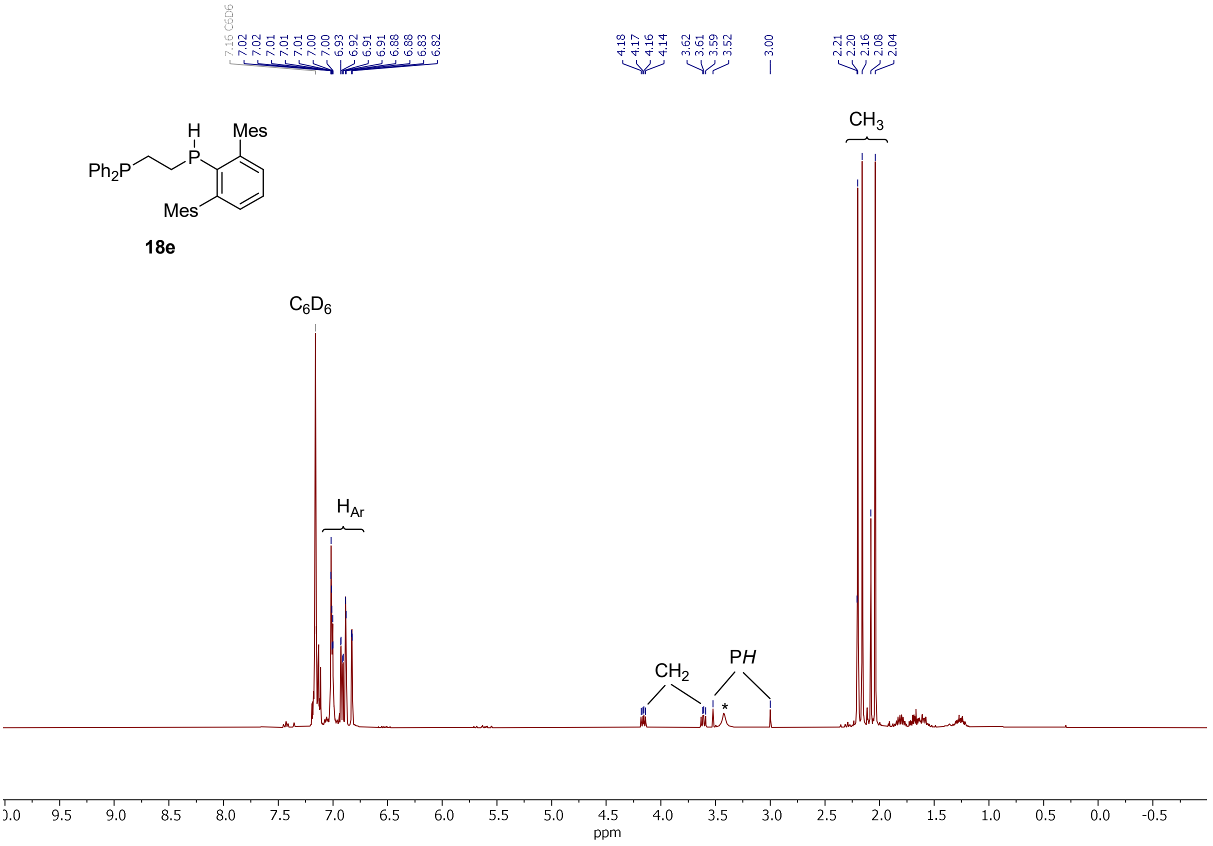

Figure S22. ^{1}H NMR spectrum of isolated ***18e*** *in benzene-d_6 at 300 K. *belongs to 18-c-6. Minor impurities of Methylcyclohexane between 1-2 ppm.*

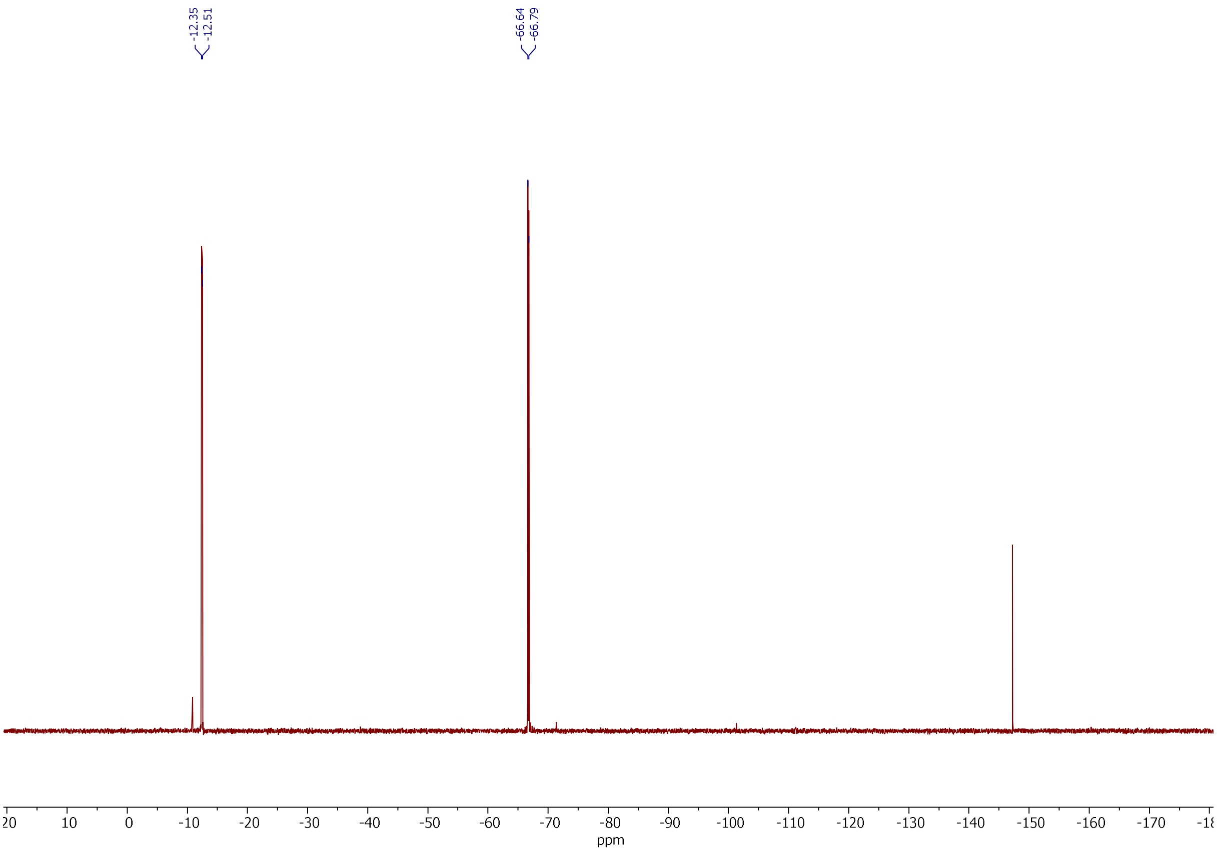

Figure S23. $^{31}P\{^{1}H\}$ NMR spectrum of isolated ***18e*** *in benzene-d_6 at 300 K. Minor impurities of $TerPH_2$ at -147 ppm.*

Section S3 – NMR Spectra of the Catalytic Reactions

Section S3.1 – NMR Spectra of the catalytic hydrophosphination reactions of 1,1-diphenylethylene 2

Ph_2PH

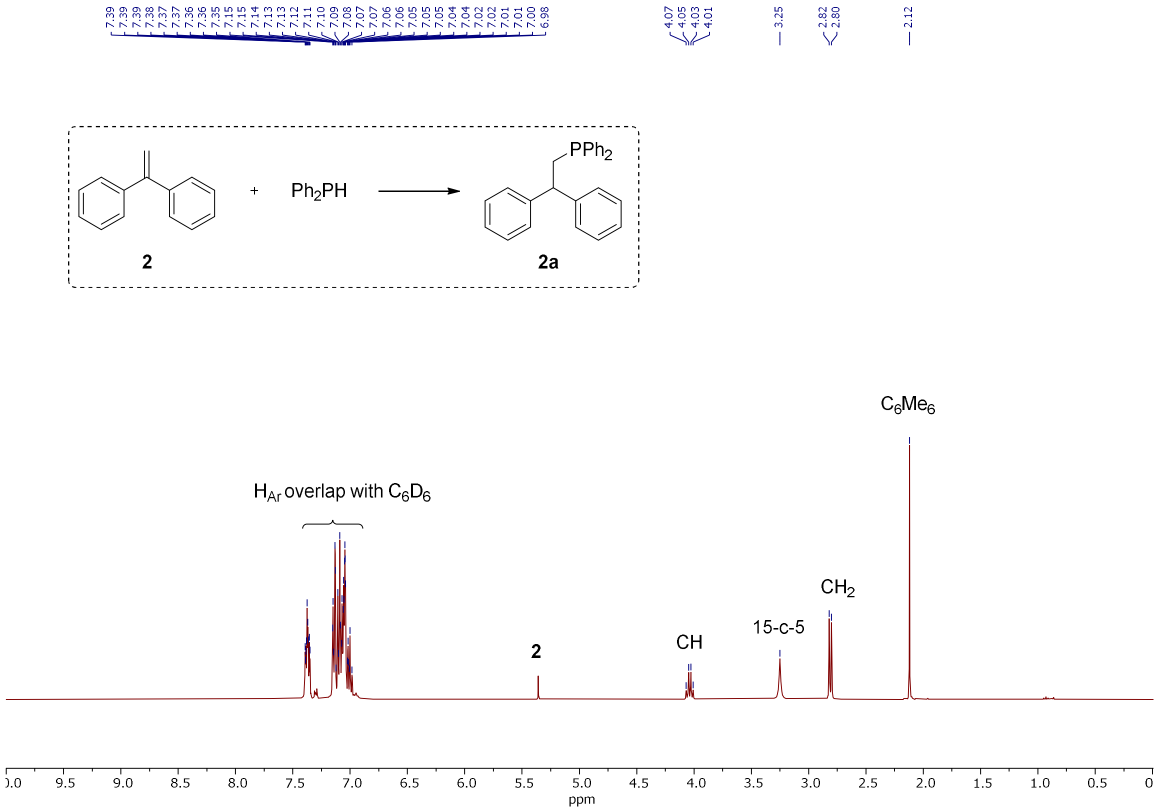

*Figure S24. ^{1}H NMR spectrum of the $\mathbf{1^{Li}}$ (10 mol%) catalysed HP reaction of **2** with Ph_2PH in benzene-d_6 at 300 K after 20 min at RT. C_6Me_6 as internal standard.*

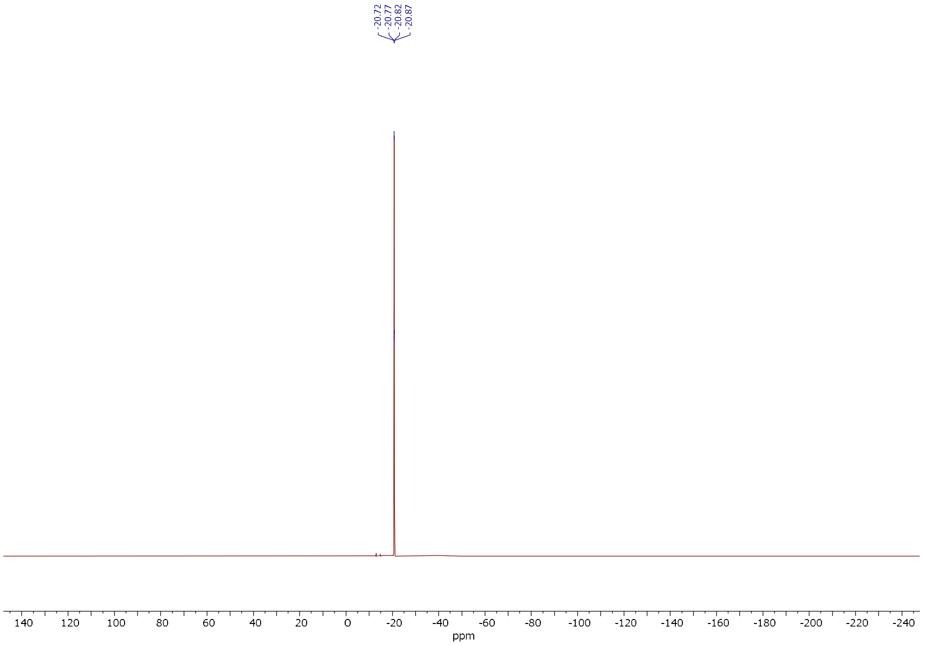

*Figure S25. ^{31}P NMR spectrum of the $\mathbf{1^{Li}}$ (10 mol%) catalysed HP reaction of **2** with Ph_2PH in benzene-d_6 at 300 K after 20 min at RT. Only product **2a** (-21 ppm) visible.*

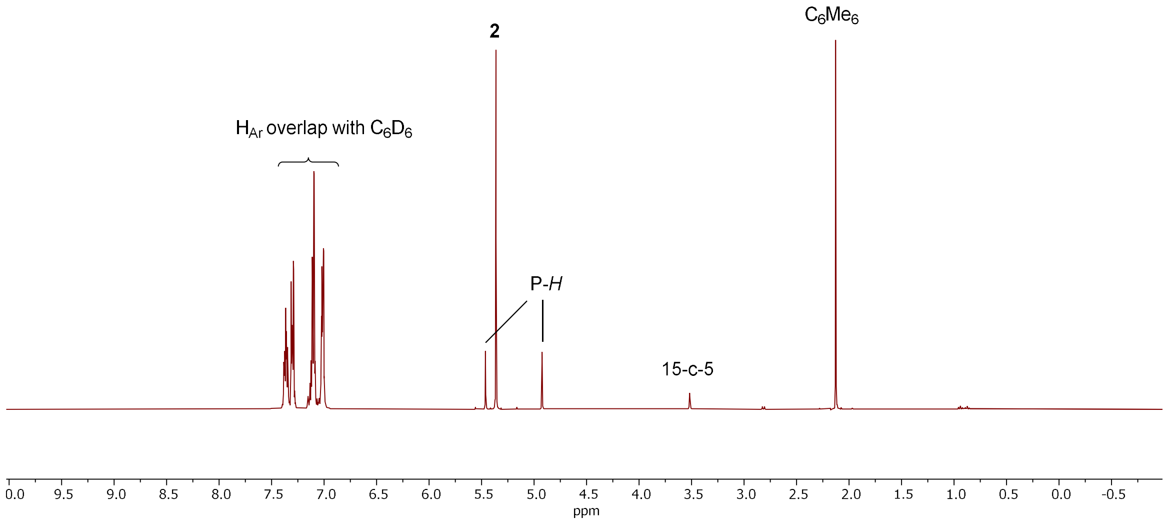

*Figure S26. ^{1}H NMR spectrum of the **1**^{Li} (1 mol%) catalysed HP reaction of **2** with Ph_2PH in benzene-d_6 at 300 K after 24 h at RT. C_6Me_6 as internal standard. No conversion.*

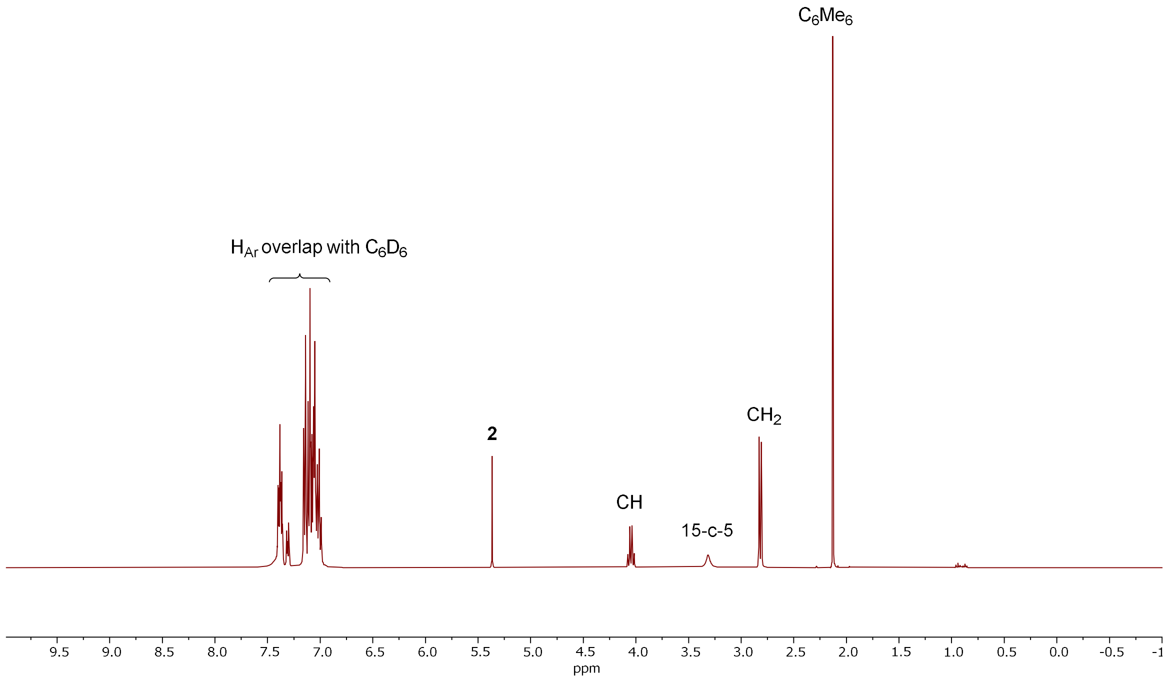

*Figure S27. ^{1}H NMR spectrum of the **1**^{Li} (5 mol%) catalysed HP reaction of **2** with Ph_2PH in benzene-d_6 at 300 K after 22 min at RT. C_6Me_6 as internal standard. 81% conversion.*

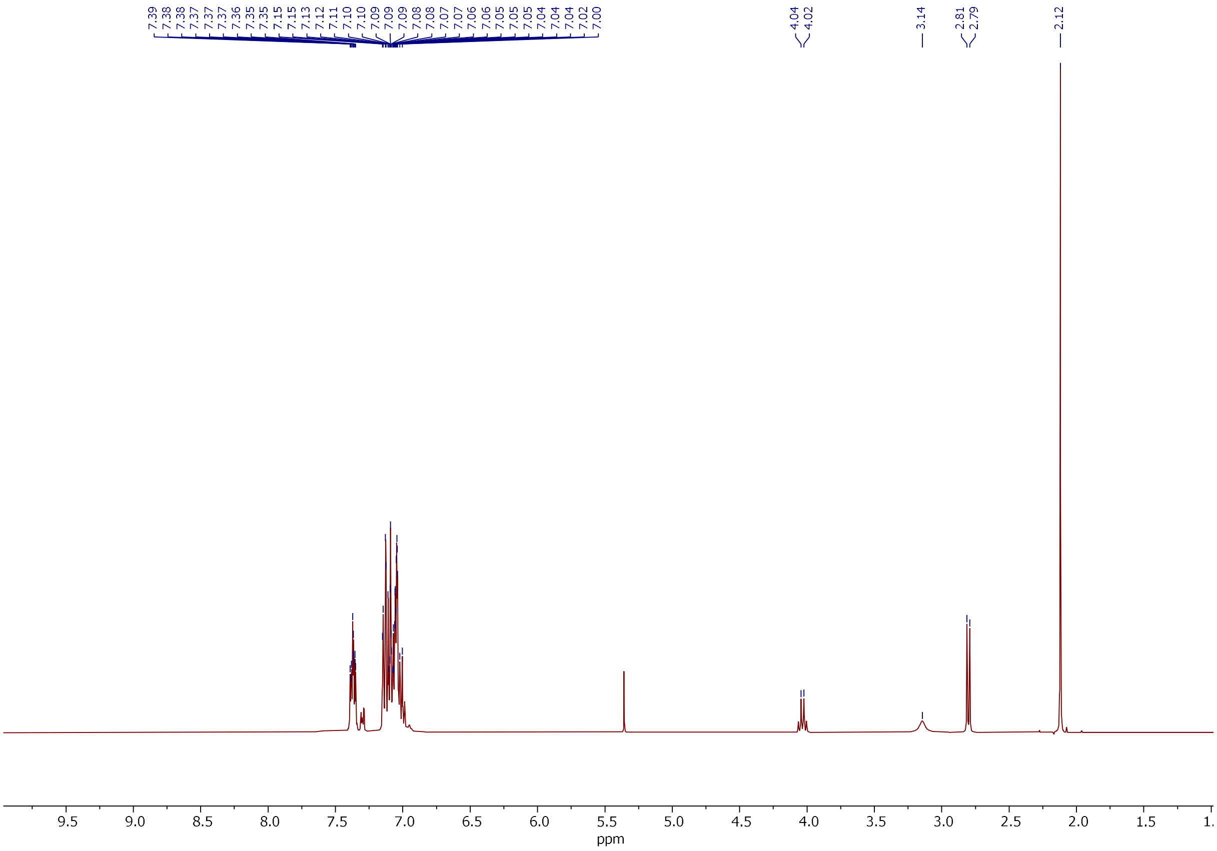

*Figure S28. ^{1}H NMR spectrum of the **1^{Na}** (10 mol%) catalysed HP reaction of **2** with Ph_2PH in benzene-d_6 at 300 K after 20 min at RT. C_6Me_6 as internal standard.*

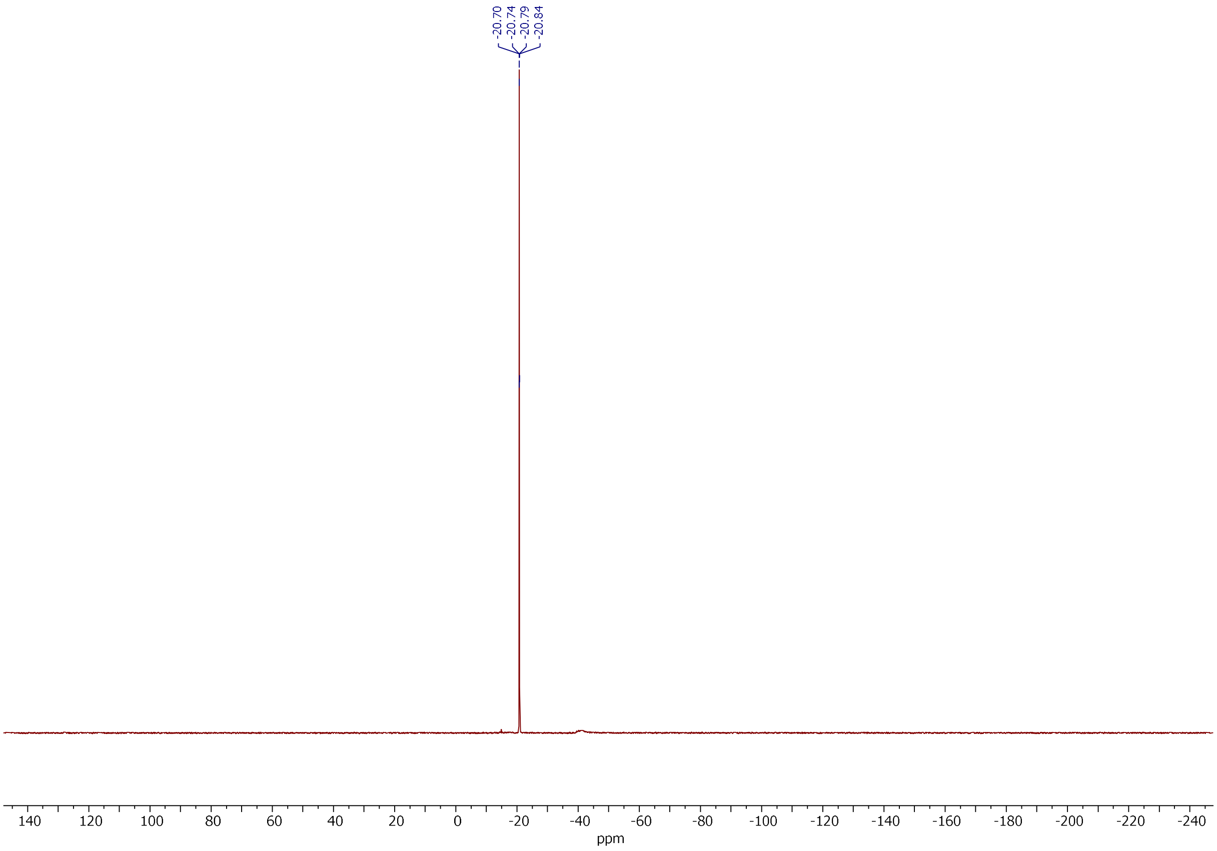

*Figure S29. ^{31}P NMR spectrum of the **1^{Na}** (10 mol%) catalysed HP reaction of **2** with Ph_2PH in benzene-d_6 at 300 K after 20 min at RT. Only product **2a** (-21 ppm) visible.*

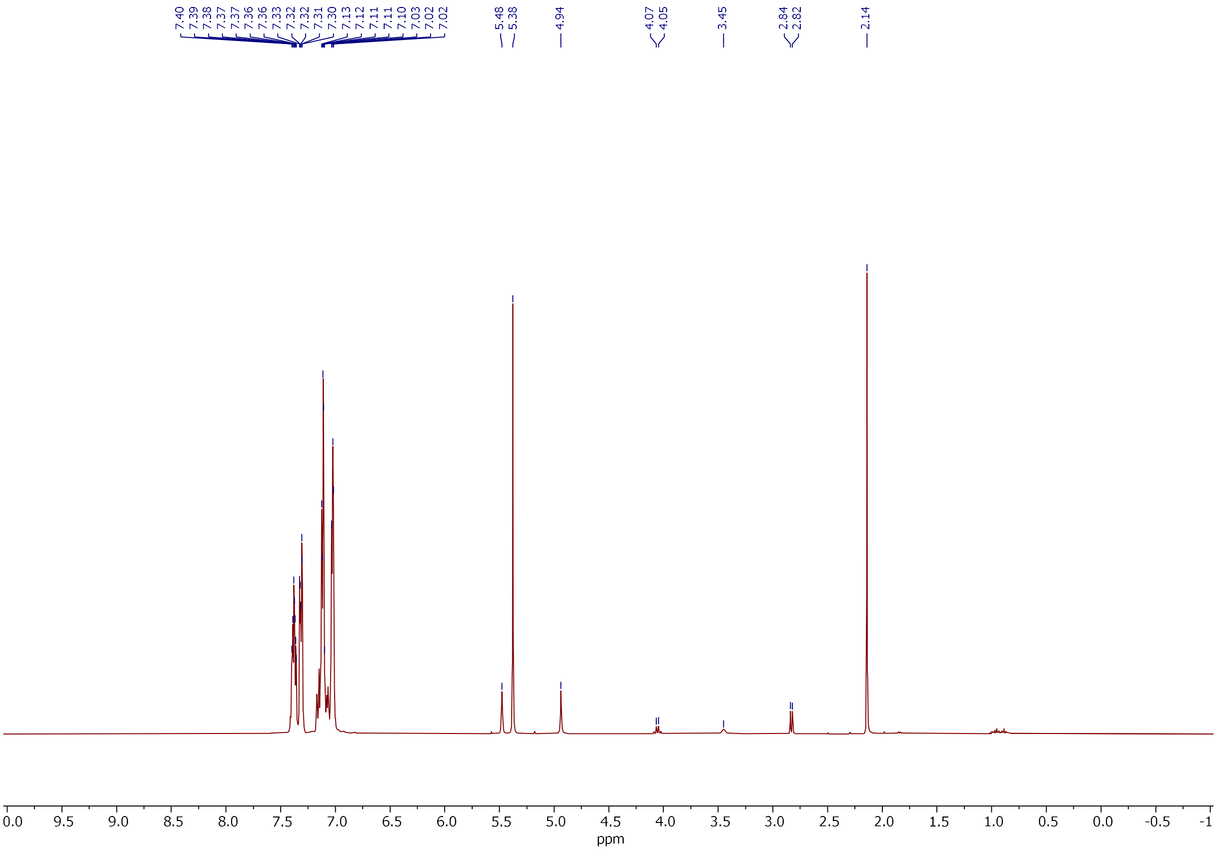

*Figure S30. ^{1}H NMR spectrum of the **1^{Na}** (1 mol%) catalysed HP reaction of **2** with Ph_2PH in benzene-d_6 at 300 K after 14 h at RT. C_6Me_6 as internal standard. Mixture of starting materials and product (30 % conversion).*

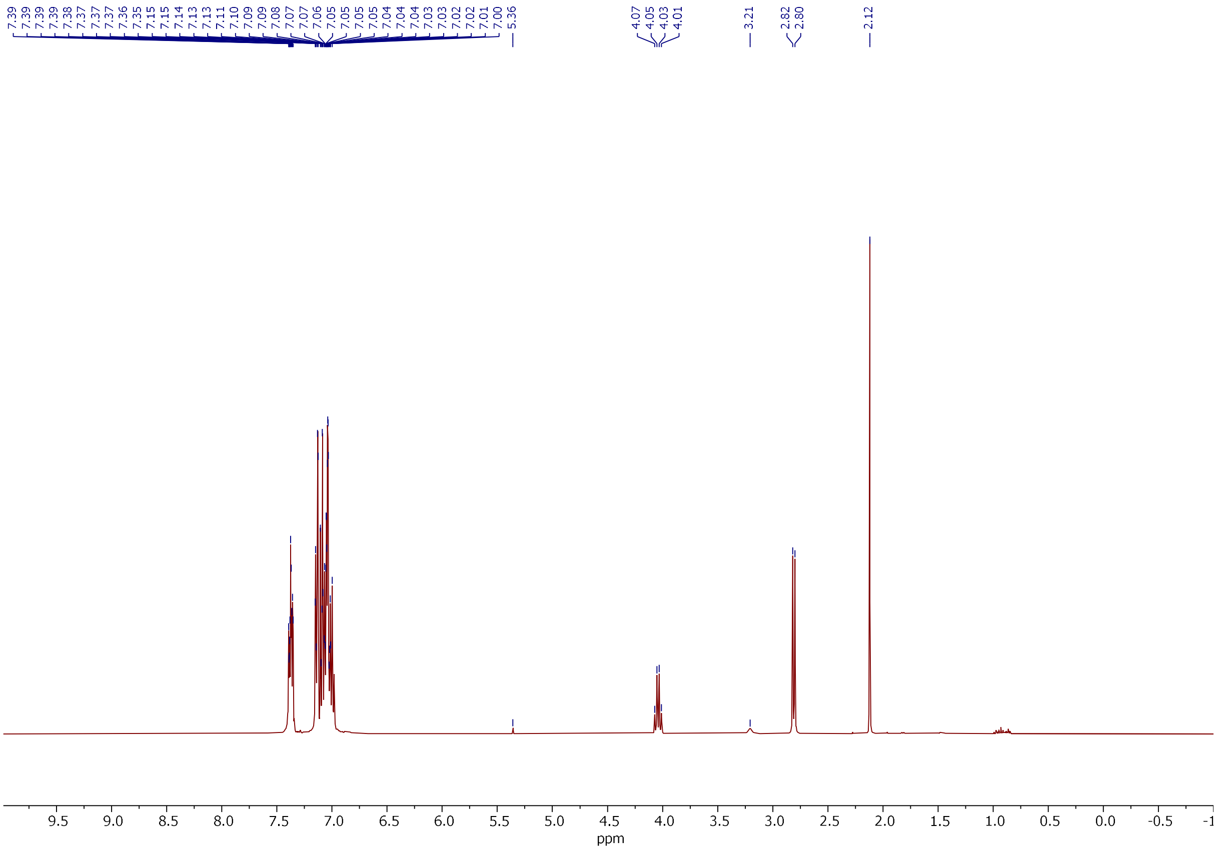

*Figure S31. ^{1}H NMR spectrum of the **1^{K}** (1 mol%) catalysed HP reaction of **2** with Ph_2PH in benzene-d_6 at 300 K after 1 h at RT. C_6Me_6 as internal standard. Full conversion.*

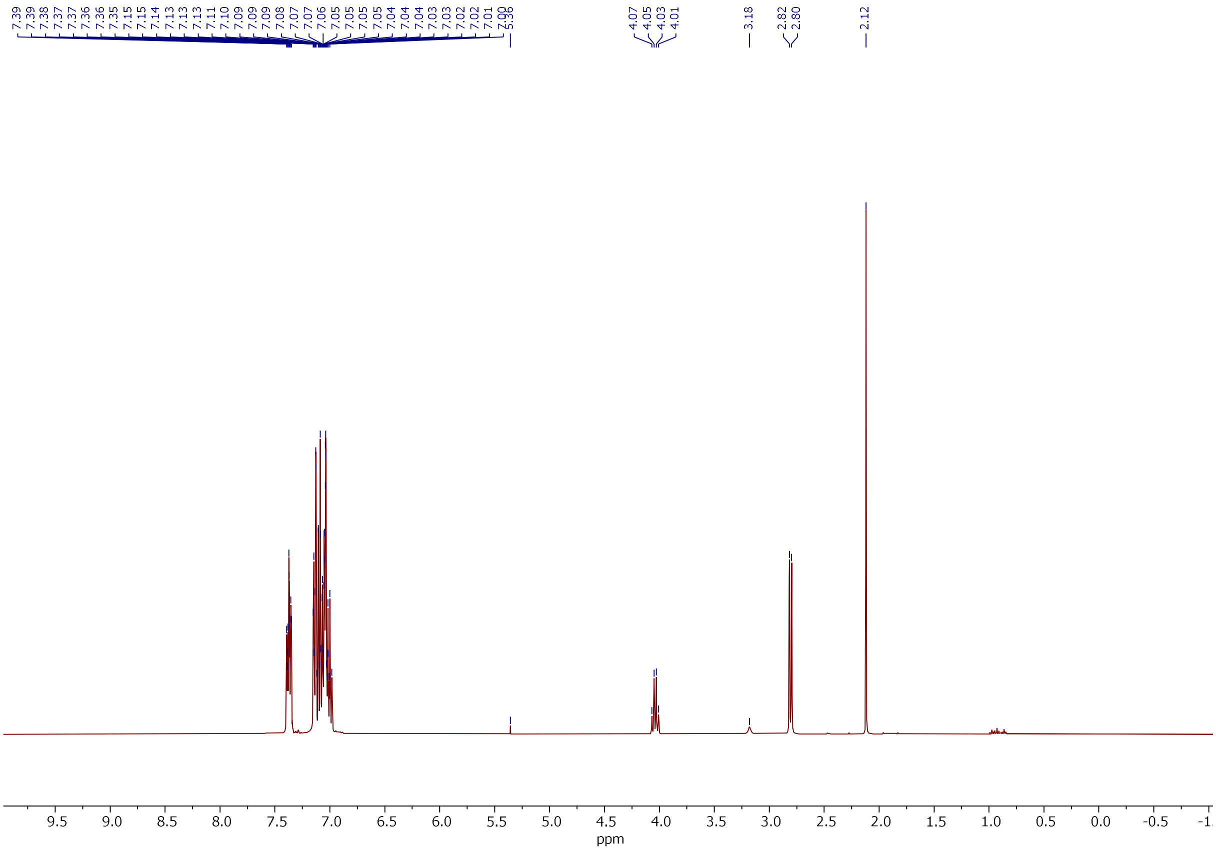

*Figure S32. ^{1}H NMR spectrum of the **1^{Rb}** (1 mol%) catalysed HP reaction of **2** with Ph_2PH in benzene-d_6 at 300 K after 28 min at RT. C_6Me_6 as internal standard. Full conversion.*

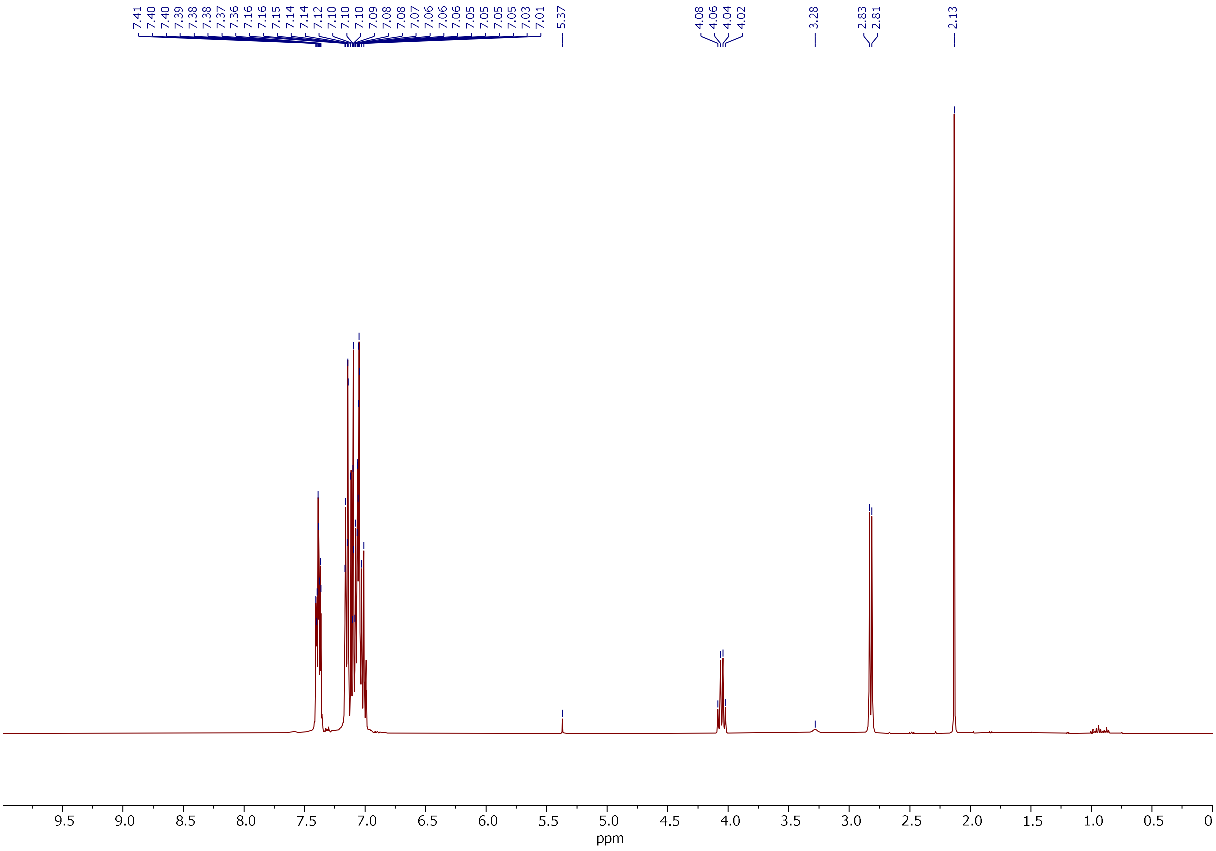

*Figure S33. ^{1}H NMR spectrum of the **1^{Cs}** (1 mol%) catalysed HP reaction of **2** with Ph_2PH in benzene-d_6 at 300 K after 12 min at RT. C_6Me_6 as internal standard. Full conversion.*

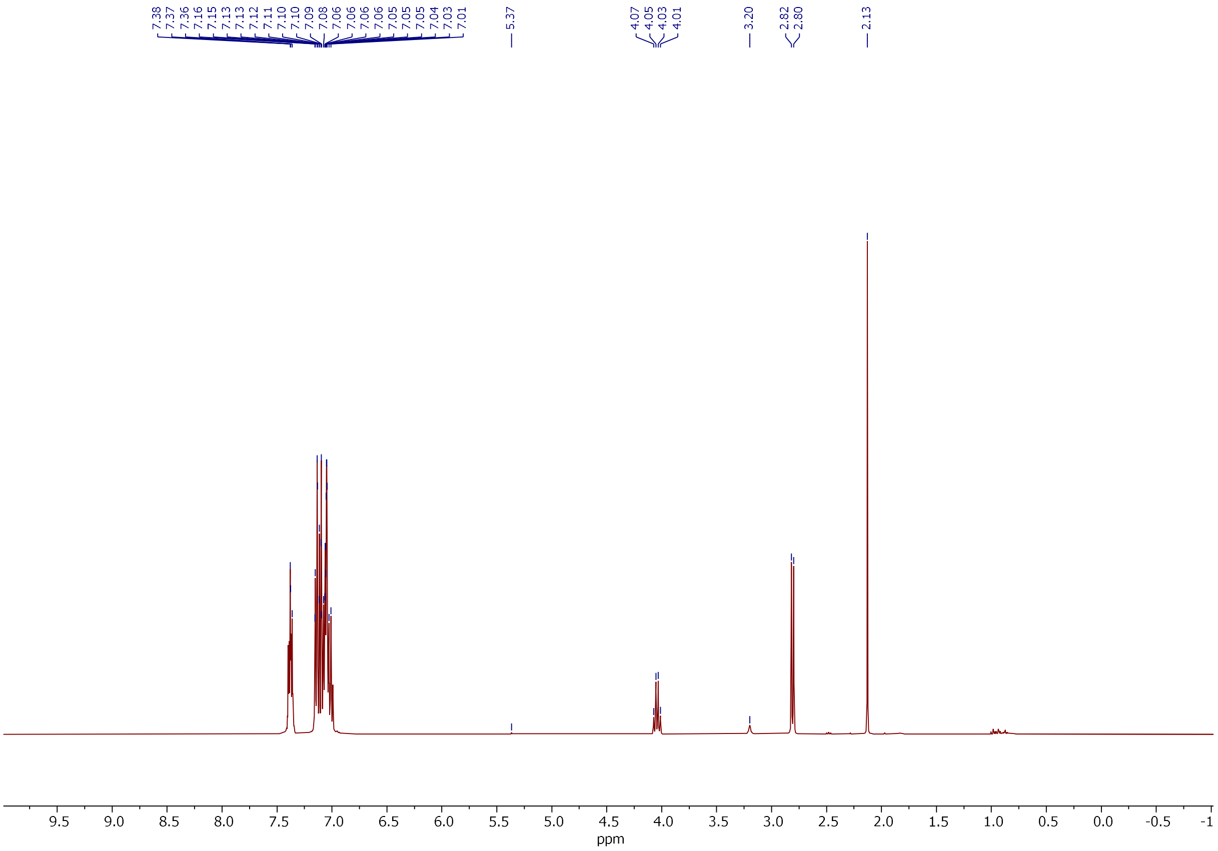

*Figure S34. ^{1}H NMR spectrum of the **1**^{Cs} (1 mol%, 0.005 M) catalysed HP reaction of **2** with Ph_2PH in benzene-d_6 at 300 K after 12 min at RT. C_6Me_6 as internal standard. Full conversion.*

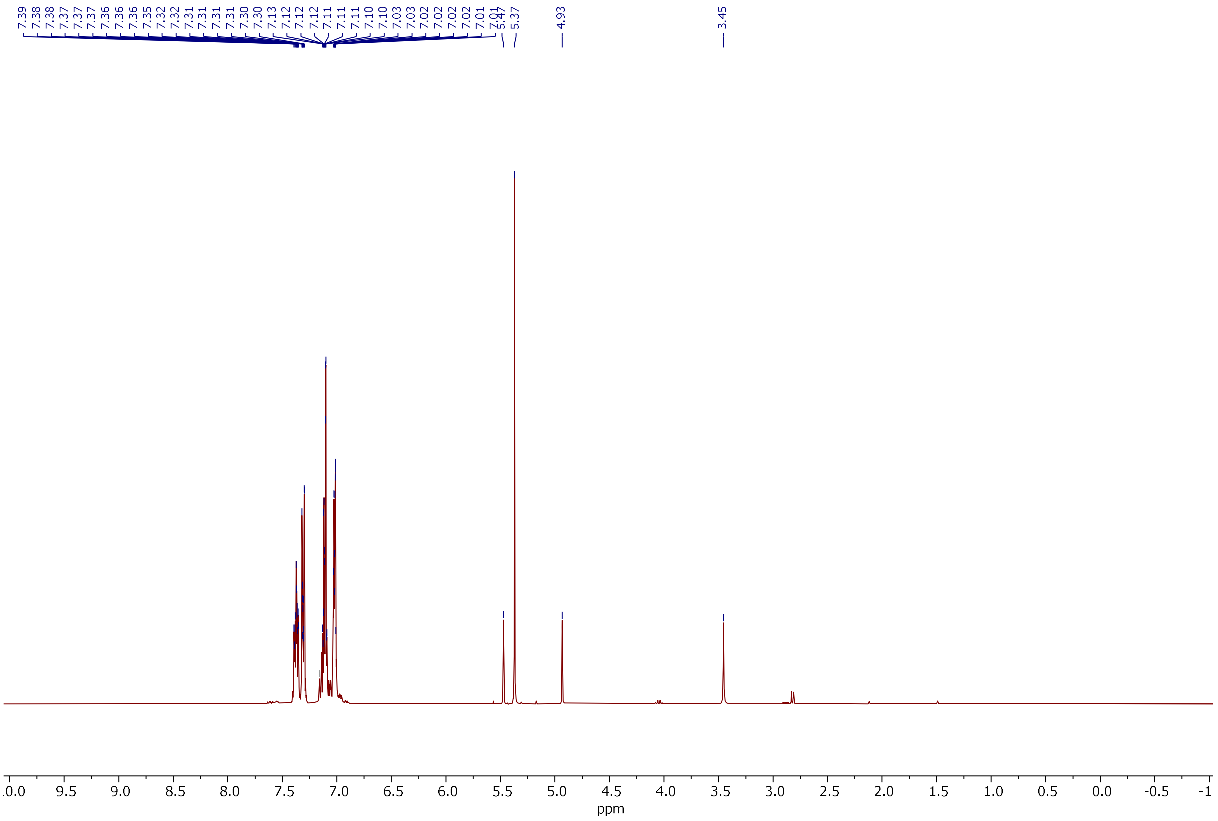

*Figure S35. ^{1}H NMR spectrum of the **1**^{Cs} (0.5 mol%, 0.005 M) catalysed HP reaction of **2** with Ph_2PH in benzene-d_6 at 300 K after 4 h at RT. no internal standard, no conversion.*

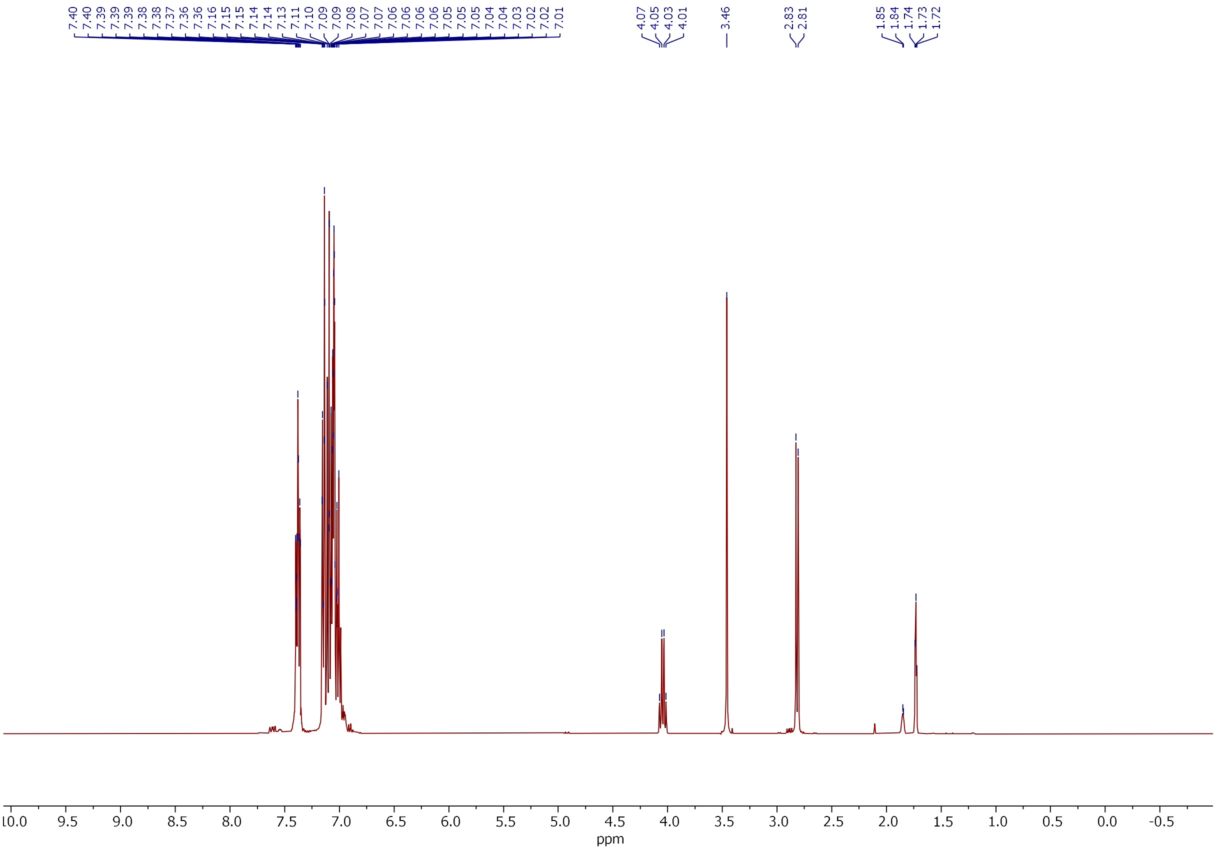

Figure S36. ^{1}H NMR spectrum of the ***1***^{Cs} *(2 mol%) catalysed HP reaction of* ***2*** *with Ph_2PH in benzene-d_6 at 300 K after 20 min at RT. Adamantane as internal standard. Full conversion.*

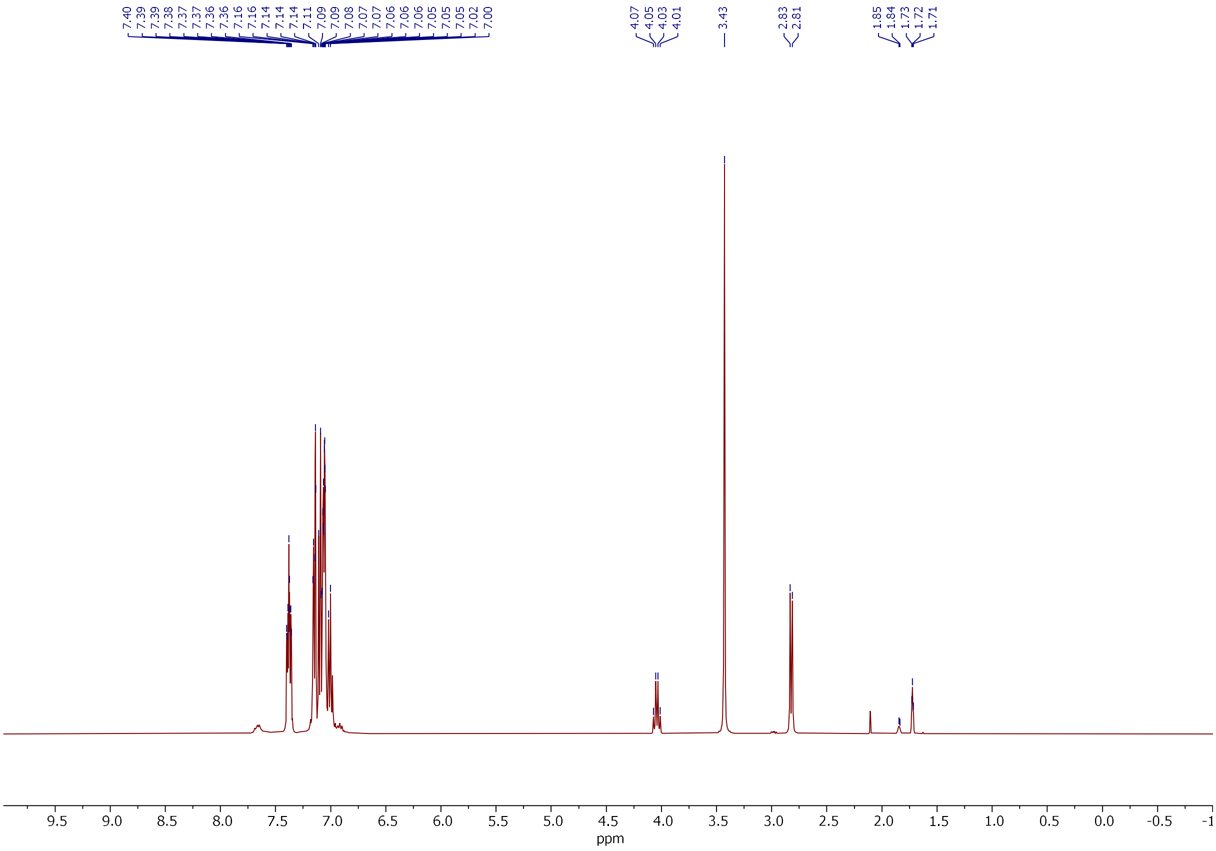

Figure S37. ^{1}H NMR spectrum of the ***1***^{Cs} *(5 mol%) catalysed HP reaction of* ***2*** *with Ph_2PH in benzene-d_6 at 300 K after 20 min at RT. Adamantane as internal standard. Full conversion.*

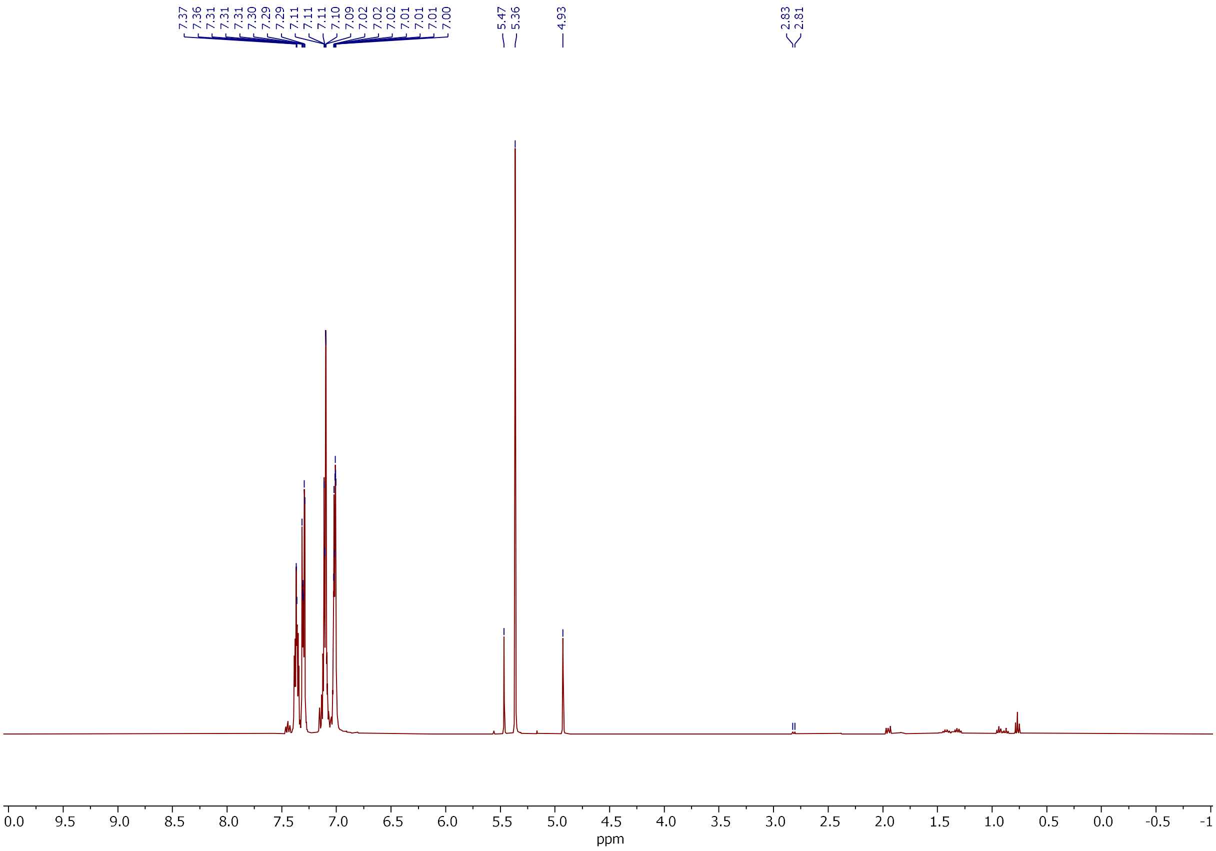

*Figure S38. ^{1}H NMR spectrum of the [$^{n}Bu_4N$][PPh_2] (5 mol%) catalysed HP reaction of **2** with Ph_2PH in benzene-d_6 at 300 K after 20 h at RT. No internal standard. <1 % conversion.*

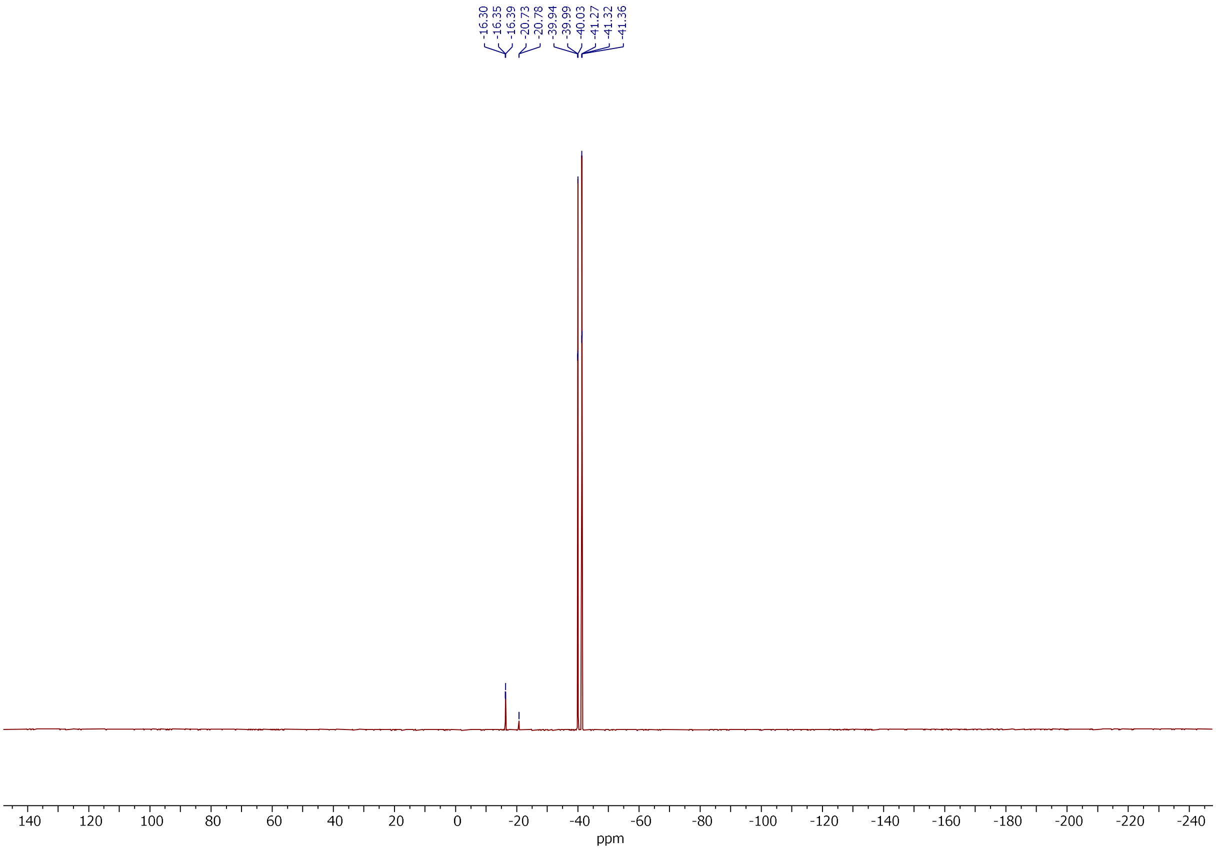

*Figure S39. ^{31}P NMR spectrum of the [$^{n}Bu_4N$][PPh_2] (5 mol%) catalysed HP reaction of **2** with Ph_2PH in benzene-d_6 at 300 K after 20 h at RT. <1 % conversion.*

Different Solvents

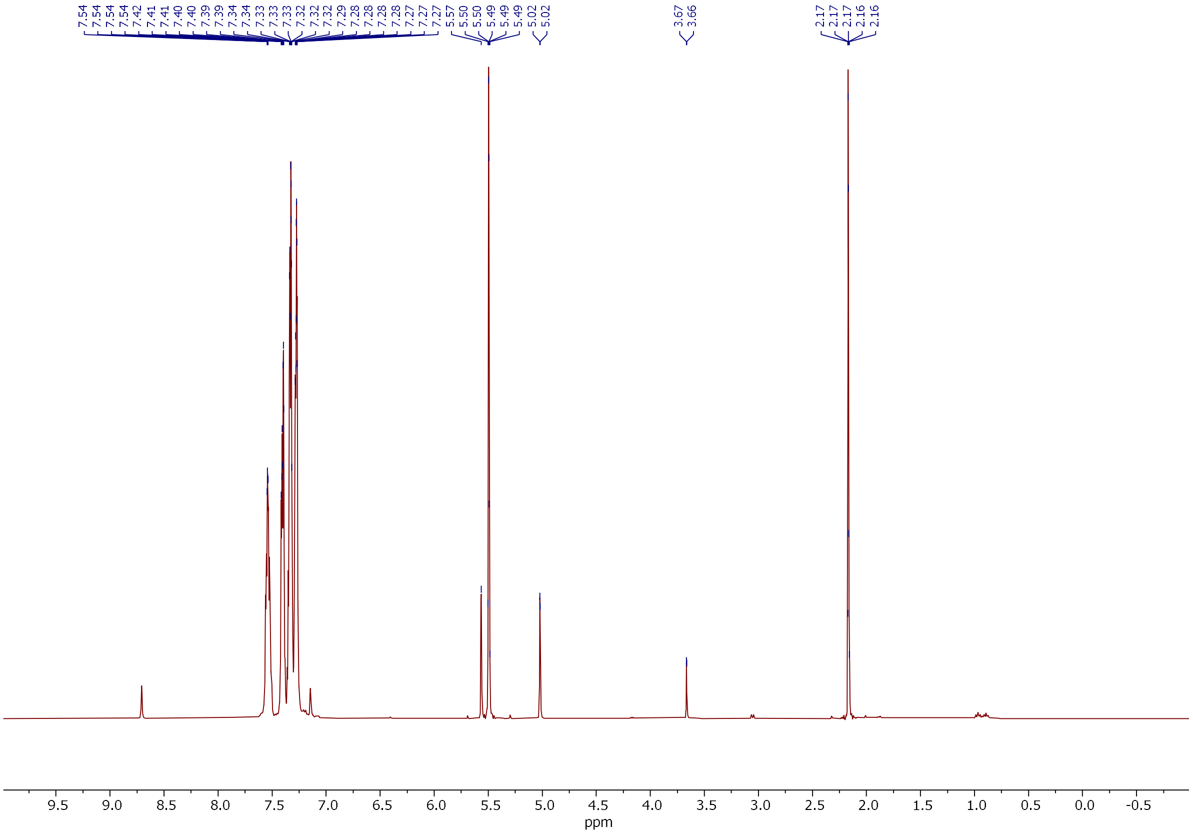

Figure S40. ¹H NMR spectrum of the ***1****^{Li} (1 mol%) catalysed HP reaction of* ***2*** *with Ph_2PH in pyridine-d_5 at 300 K after 24 h at RT. C_6Me_6 as internal standard. No conversion.*

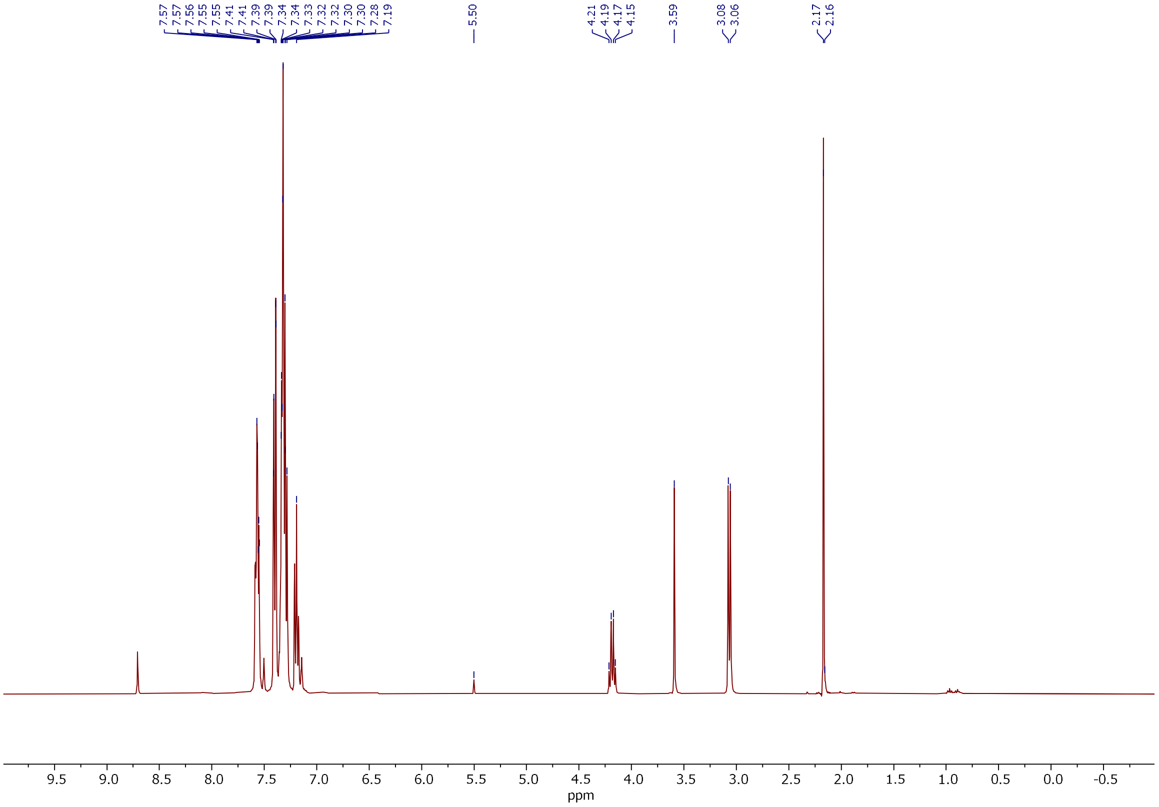

Figure S41. ¹H NMR spectrum of the ***1****^{Li} (5 mol%) catalysed HP reaction of* ***2*** *with Ph_2PH in pyridine-d_5 at 300 K after 11 min at RT. C_6Me_6 as internal standard. 99% conversion.*

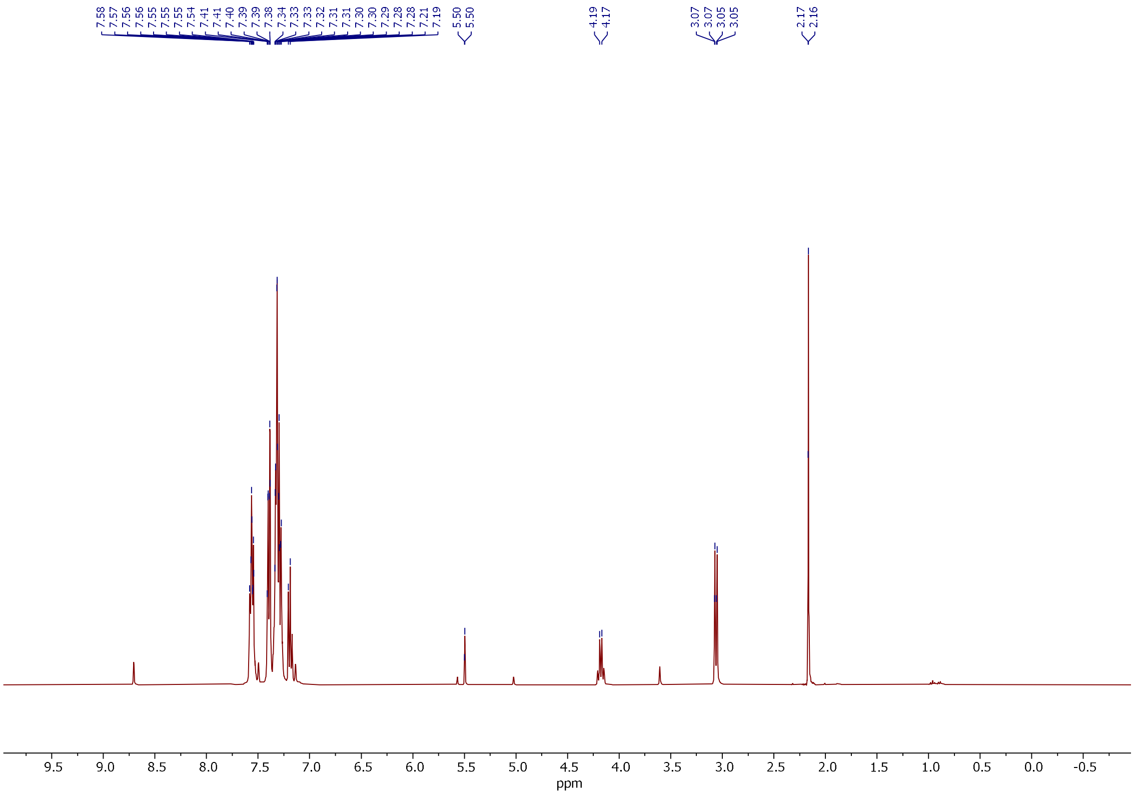

*Figure S42. ^{1}H NMR spectrum of the $\mathbf{1}^{\mathbf{Na}}$ (1 mol%) catalysed HP reaction of **2** with Ph_2PH in pyridine-d_5 at 300 K after 5 h at RT. C_6Me_6 as internal standard. 90 % conversion.*

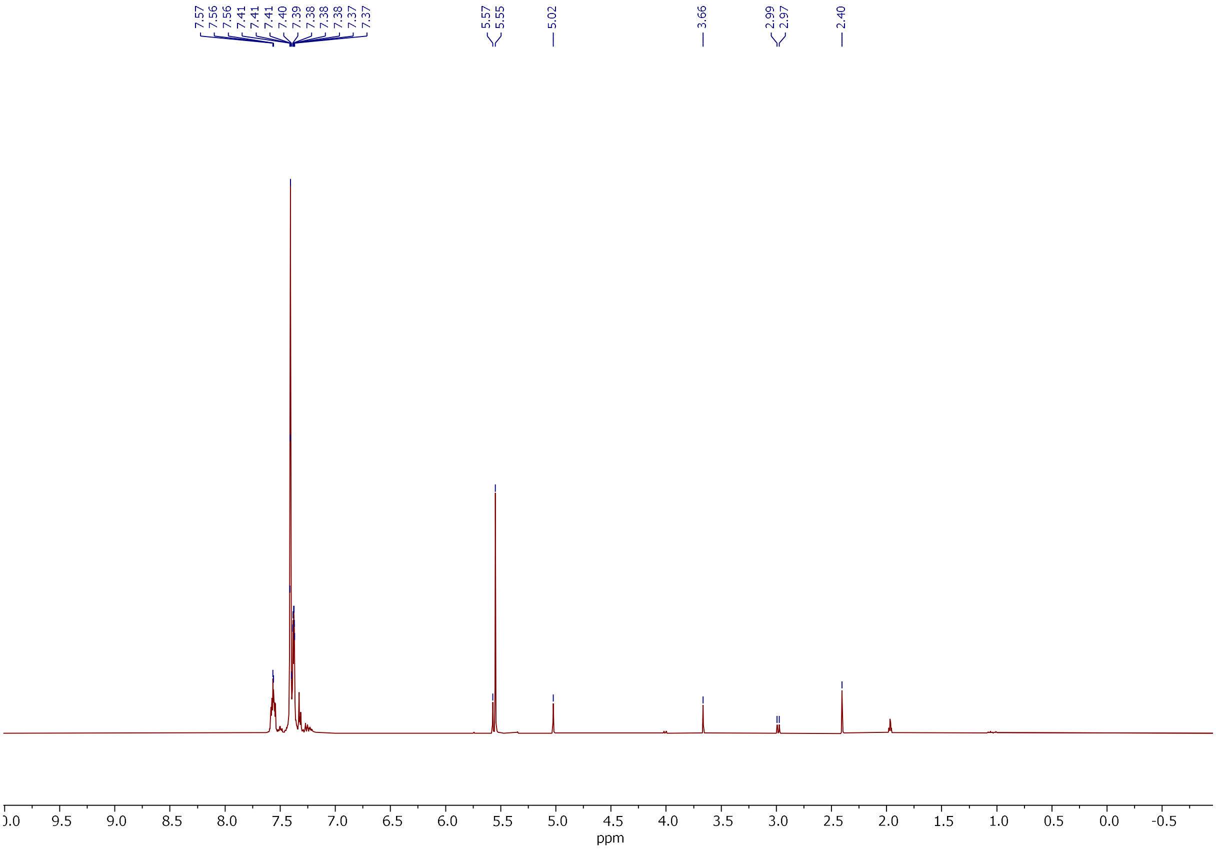

*Figure S43. ^{1}H NMR spectrum of the $\mathbf{1}^{\mathbf{Li}}$ (1 mol%) catalysed HP reaction of **2** with Ph_2PH in MeCN-d_3 at 300 K after 24 h at RT. Toluene as internal standard. 9 % conversion.*

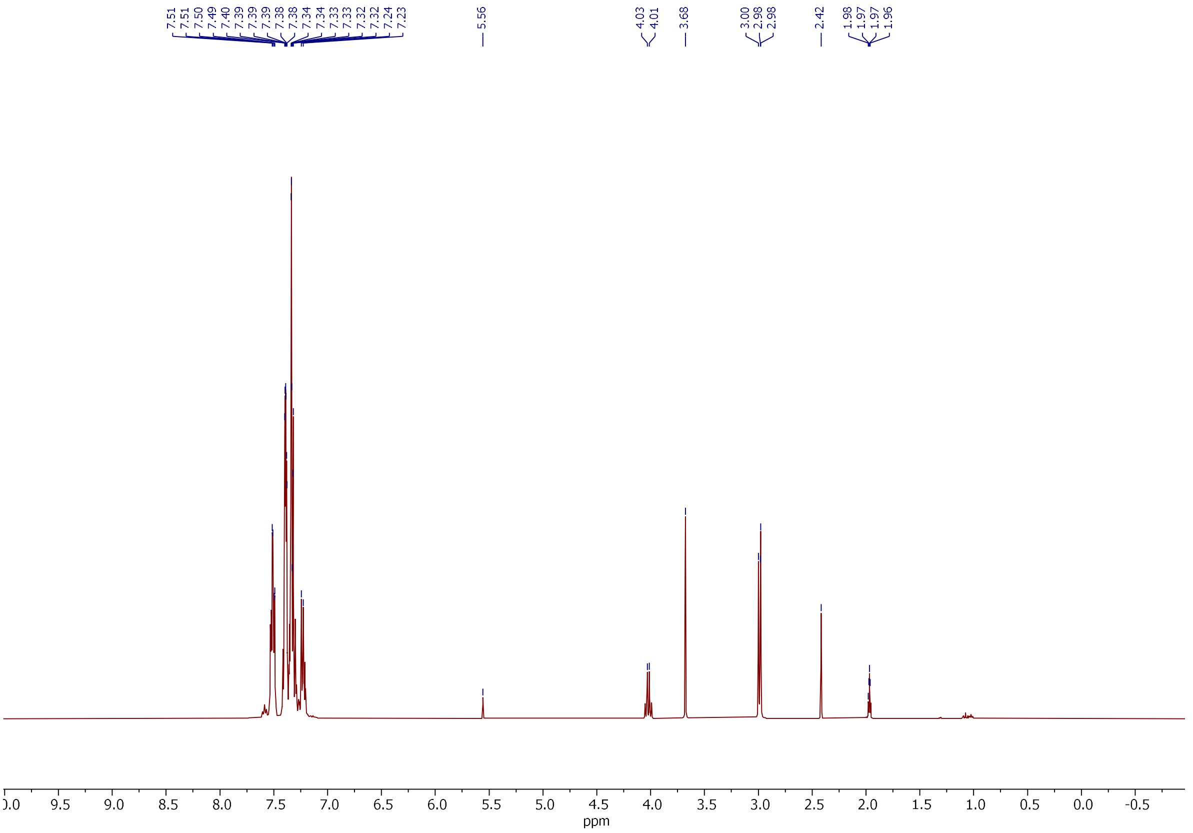

*Figure S44. ¹H NMR spectrum of the **1^{Li}** (5 mol%) catalysed HP reaction of **2** with Ph_2PH in MeCN-d_3 at 300 K after 17 min at RT. Toluene as internal standard. 98 % conversion.*

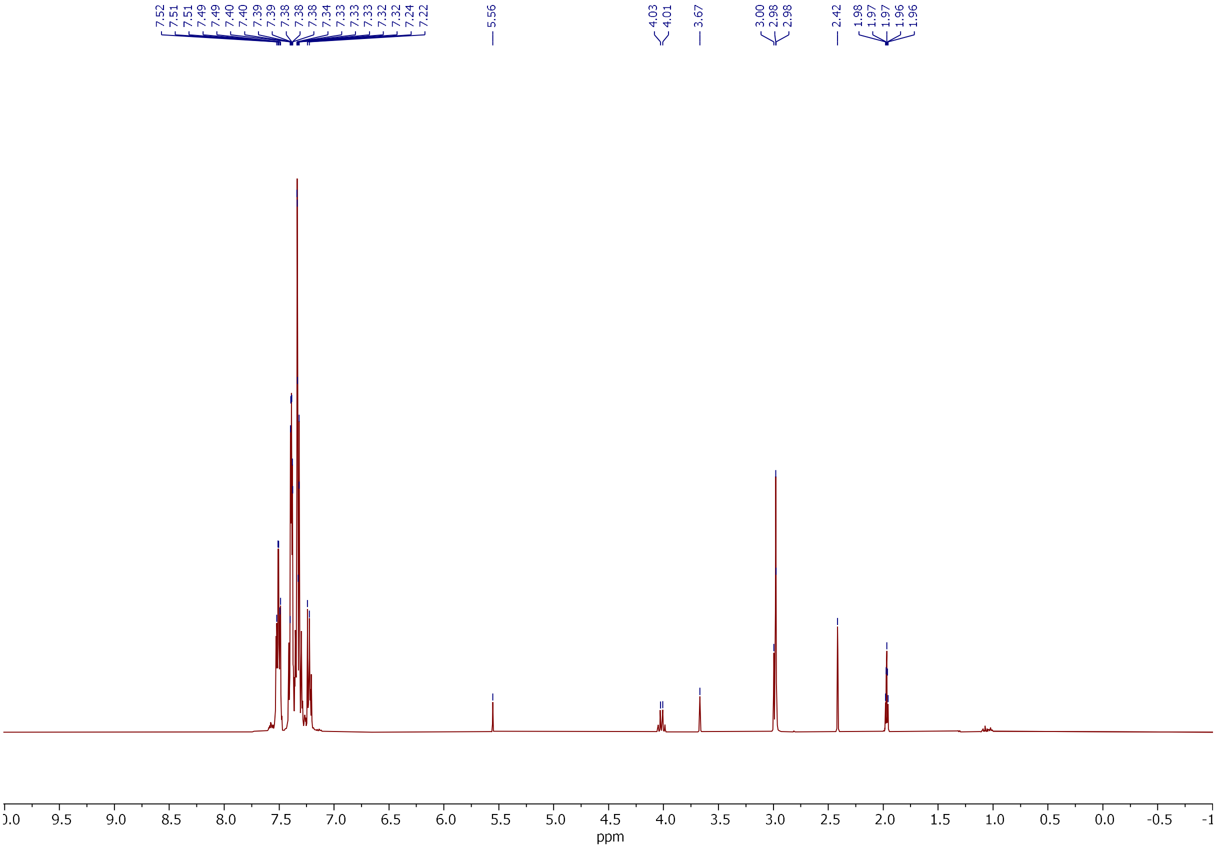

*Figure S45. ¹H NMR spectrum of the **1^{Na}** (1 mol%) catalysed HP reaction of **2** with Ph_2PH in MeCN-d_3 at 300 K after 21 min at RT. Toluene as internal standard. 97 % conversion.*

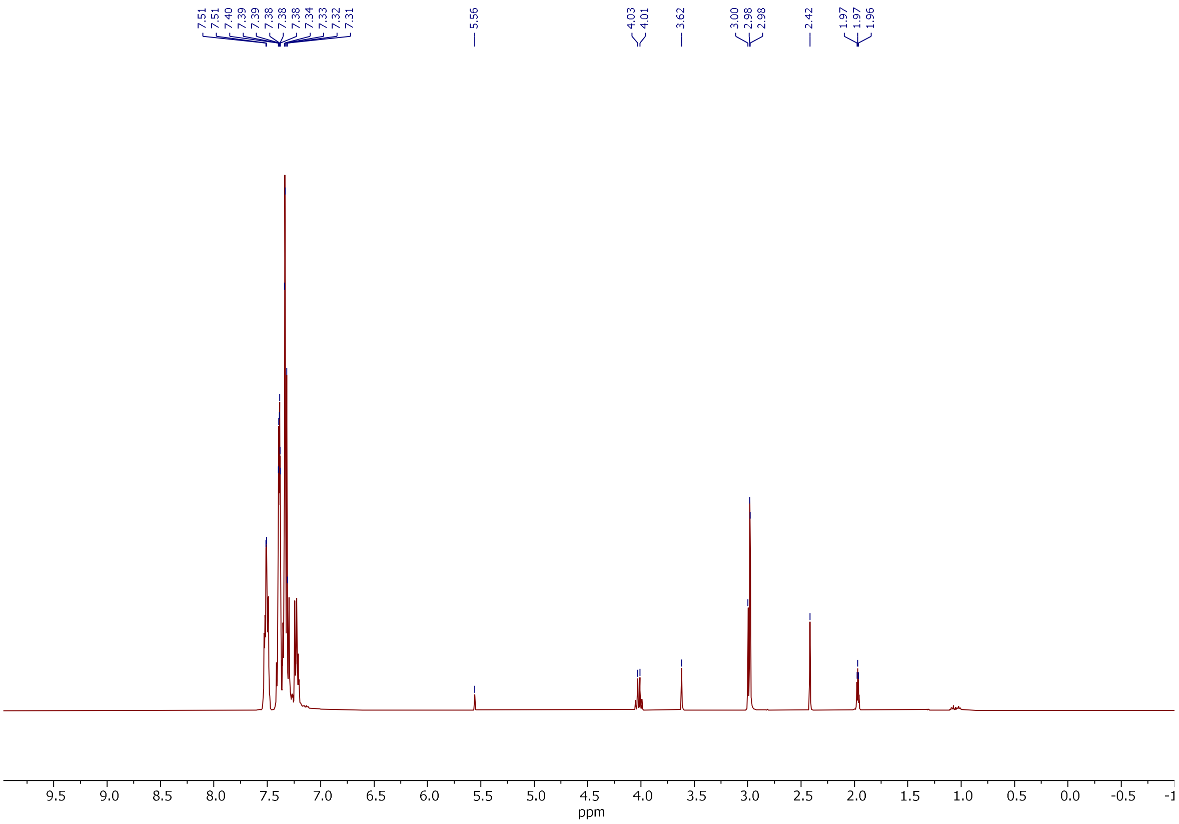

*Figure S46. ^{1}H NMR spectrum of the **1^{K}** (1 mol%) catalysed HP reaction of **2** with Ph_2PH in MeCN-d_3 at 300 K after 5 min at RT. Toluene as internal standard. 97 % conversion.*

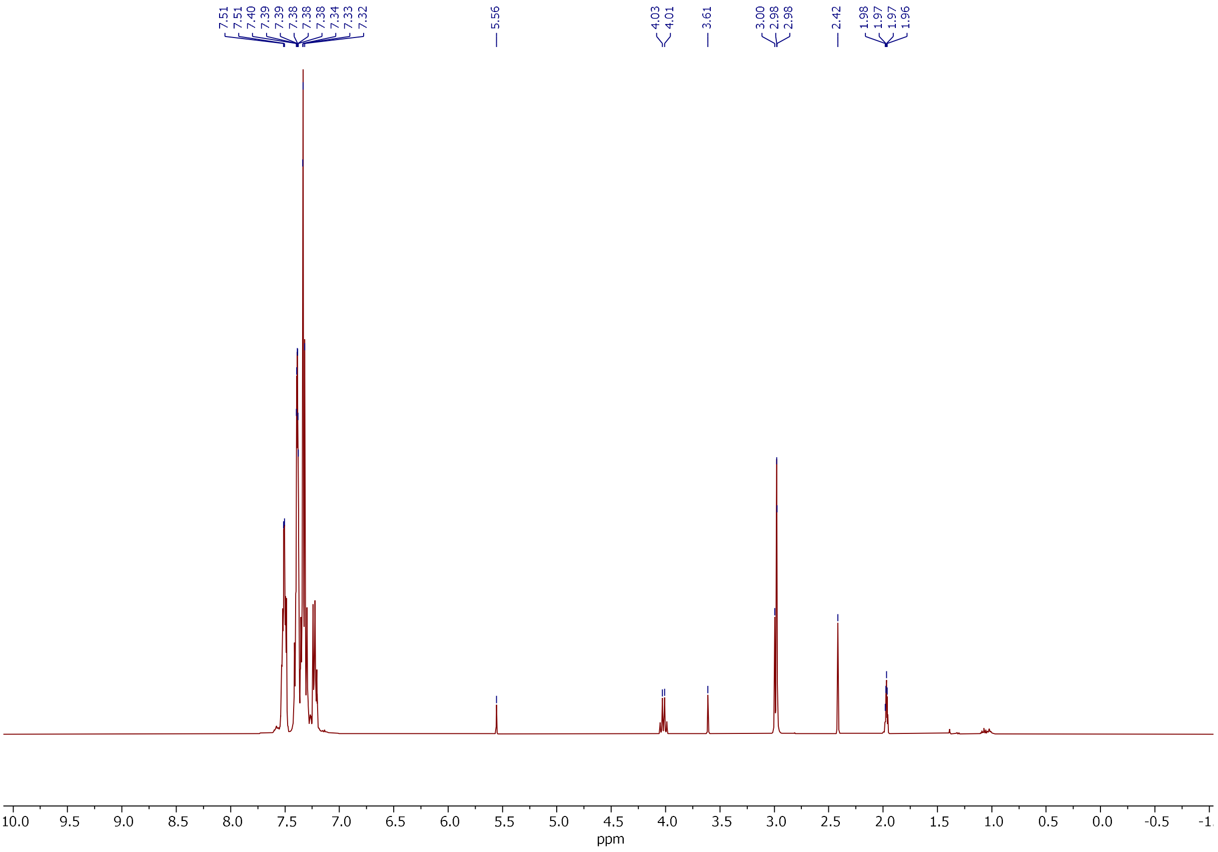

*Figure S47. ^{1}H NMR spectrum of the **1^{Rb}** (1 mol%) catalysed HP reaction of **2** with Ph_2PH in MeCN-d_3 at 300 K after 12 min at RT. Toluene as internal standard. 97 % conversion.*

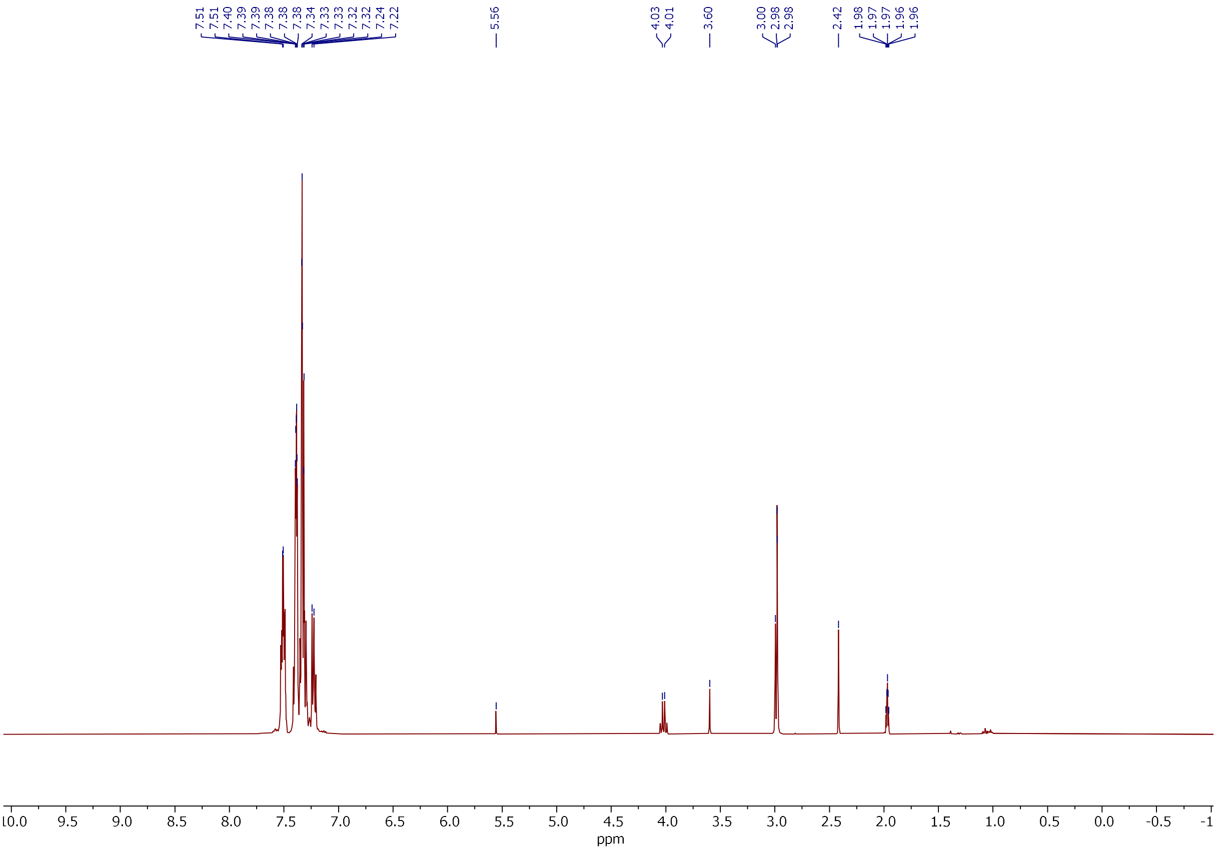

*Figure S48. ^{1}H NMR spectrum of the **1**^{Cs} (1 mol%) catalysed HP reaction of **2** with Ph_2PH in MeCN-d_3 at 300 K after 7 min at RT. Toluene as internal standard. 97 % conversion.*

^{t}BuPhPH

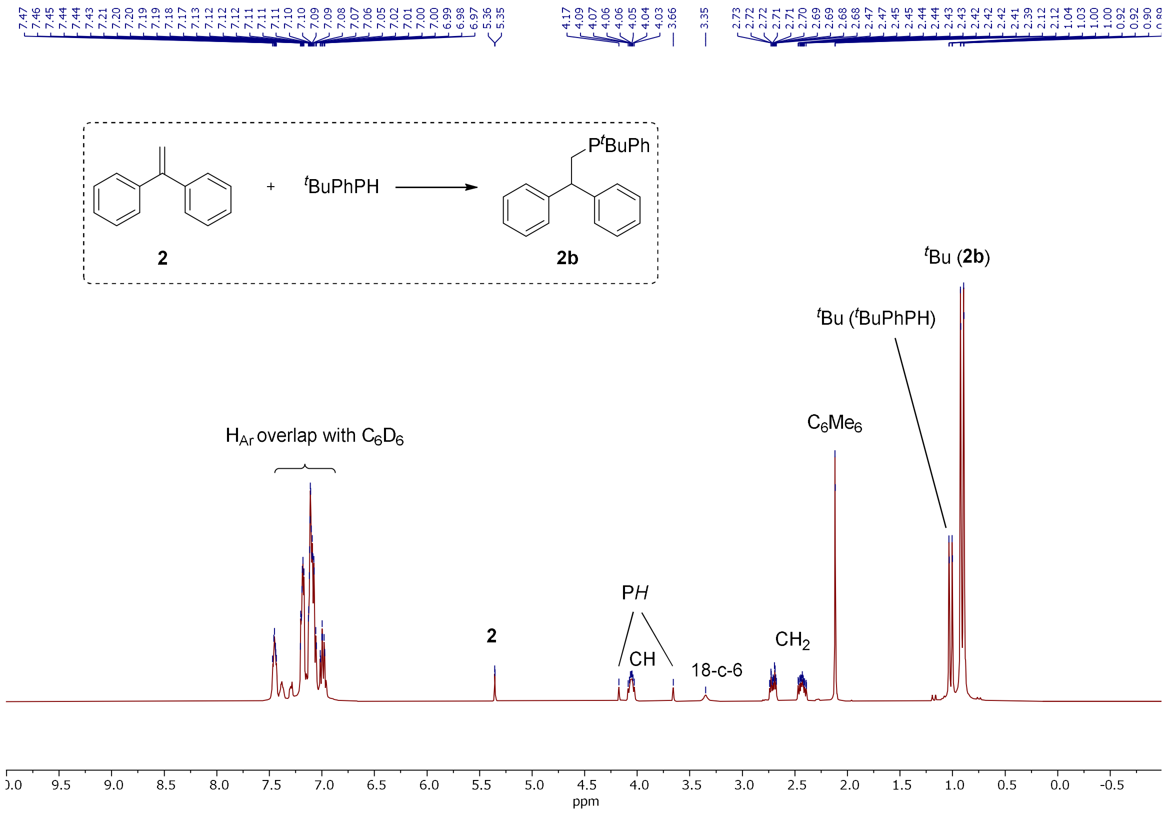

*Figure S49. ^{1}H NMR spectrum of the $\mathbf{1^K}$ (1 mol%) catalysed HP reaction of **2** with ^{t}BuPhPH in benzene-d$_6$ at 300 K after 15 h at RT. C_6Me_6 as internal standard. 97% conversion.*

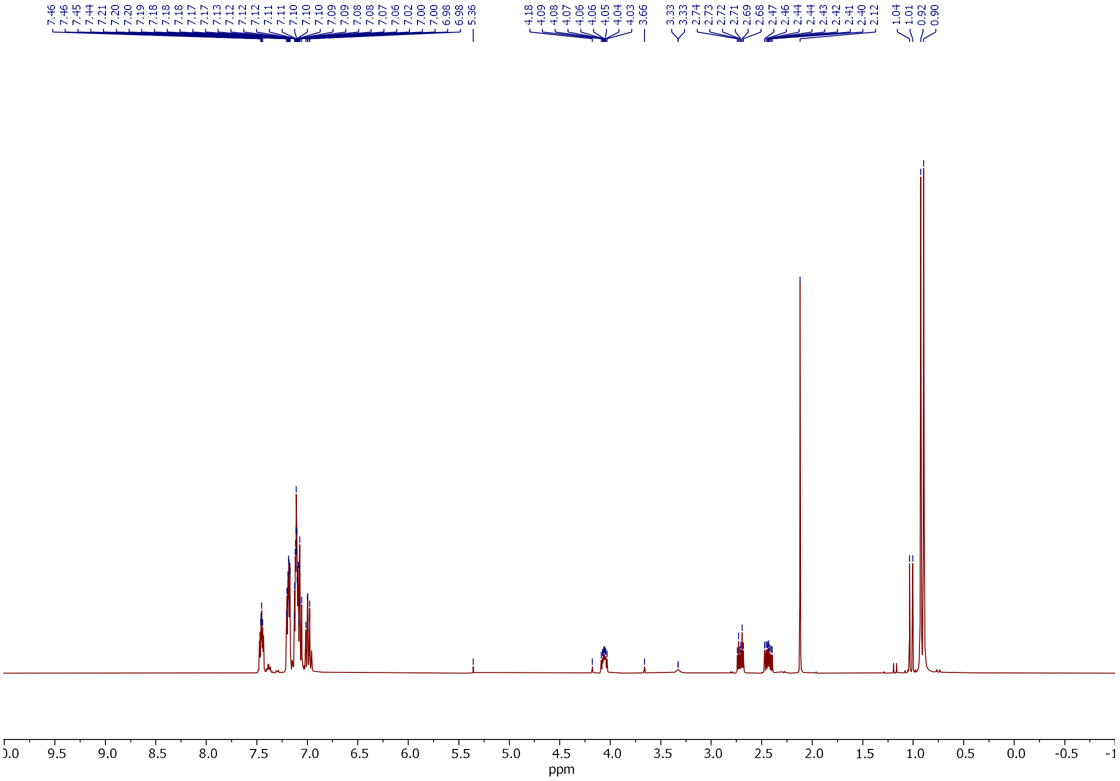

*Figure S50. ^{1}H NMR spectrum of the $\mathbf{1^{Rb}}$ (1 mol%) catalysed HP reaction of **2** with ^{t}BuPhPH in benzene-d$_6$ at 300 K after 9 min at RT. C_6Me_6 as internal standard. 97% conversion.*

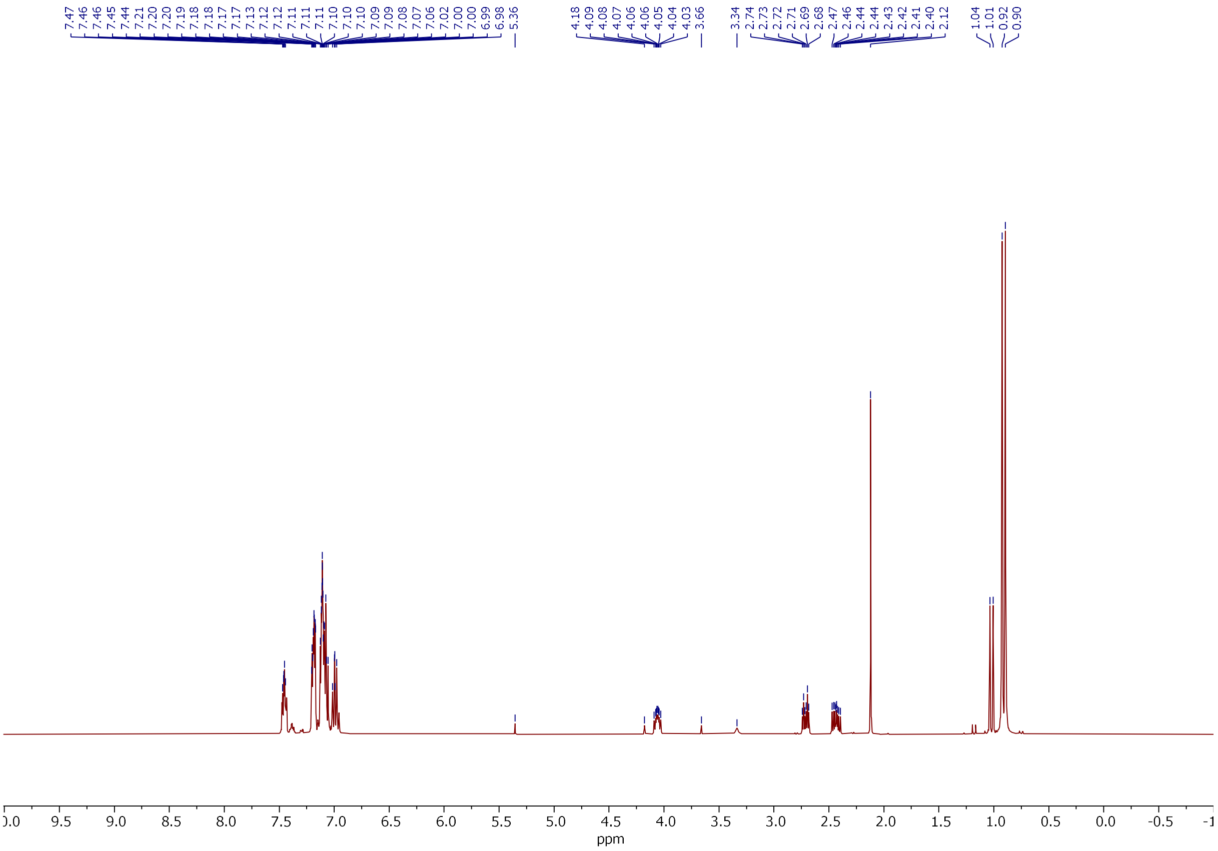

*Figure S51. ^{1}H NMR spectrum of the **1^{Cs}** (1 mol%) catalysed HP reaction of **2** with ^{t}BuPhPH in benzene-d_6 at 300 K after 7 min at RT. C_6Me_6 as internal standard. 97% conversion.*

$^tBu_2PH$

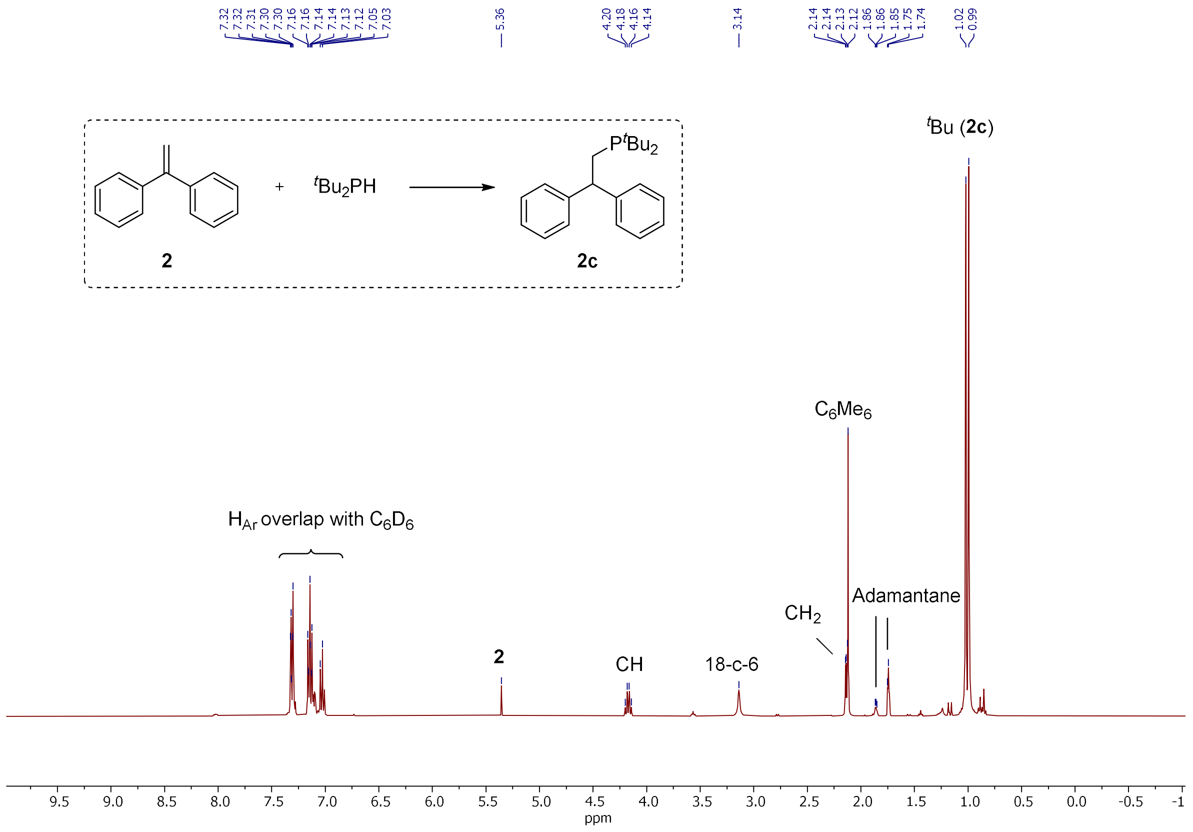

*Figure S52. ^1H NMR spectrum of the $\mathbf{1^{Rb}}$ (5 mol%) catalysed HP reaction of **2** with $^tBu_2PH$ in benzene-d_6 at 300 K after 7.1 h at RT. C_6Me_6 and Adamantane as internal standard. 97% conversion.*

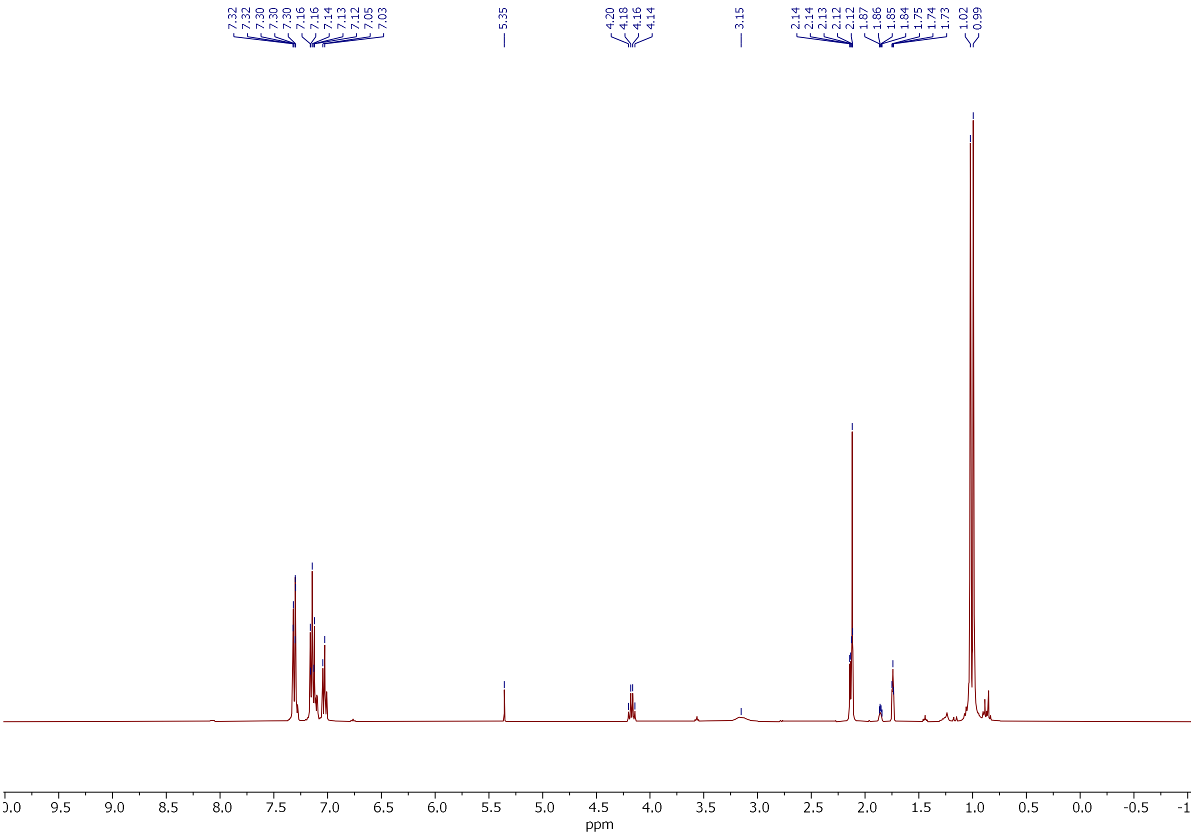

*Figure S53. ^1H NMR spectrum of the $\mathbf{1^{Cs}}$ (5 mol%) catalysed HP reaction of **2** with $^tBu_2PH$ in benzene-d_6 at 300 K after3.2 h at RT. C_6Me_6 and Adamantane as internal standard. 97% conversion.*

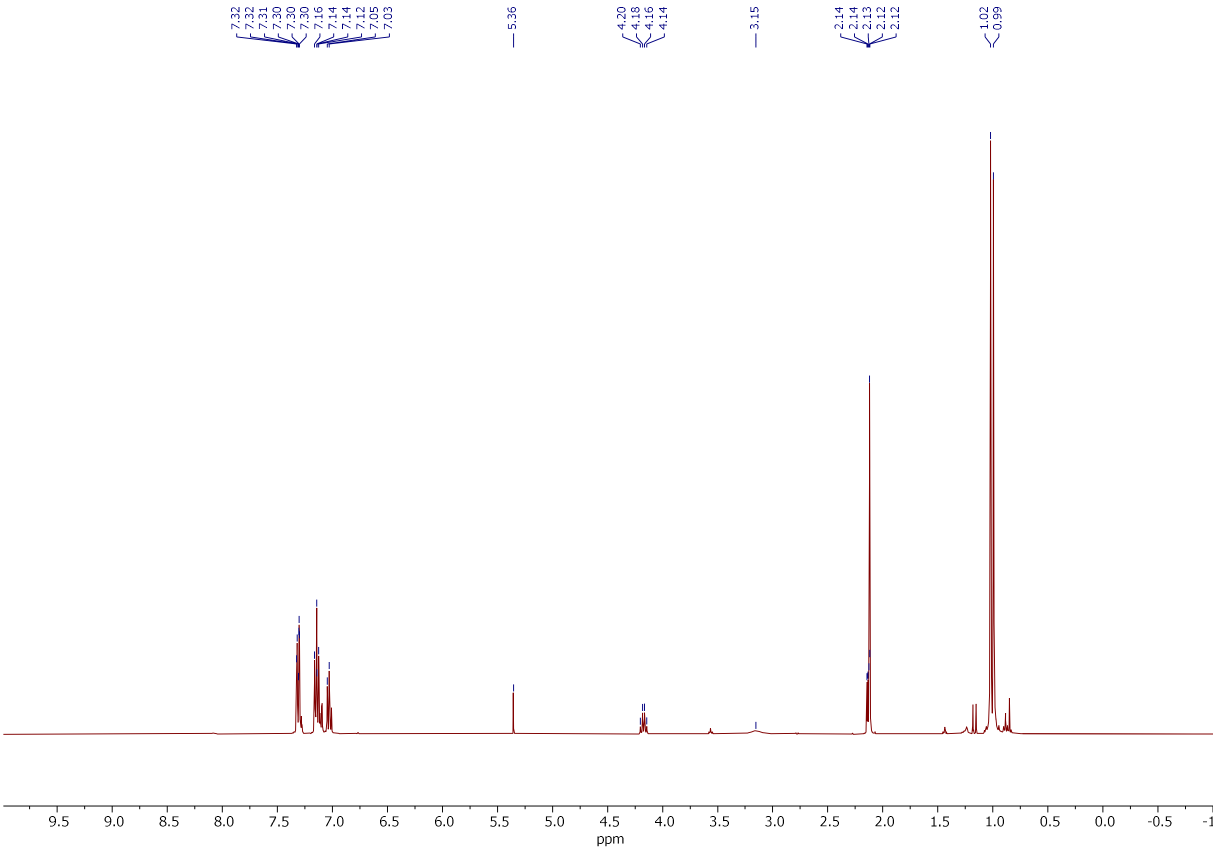

*Figure S54. ^{1}H NMR spectrum of the **1^{Cs}** (5 mol%) catalysed HP reaction of **2** with $^{t}Bu_2PH$ in benzene-d_6 at 300 K after 1 h at 75 °C. C_6Me_6 as internal standard. 96% conversion.*

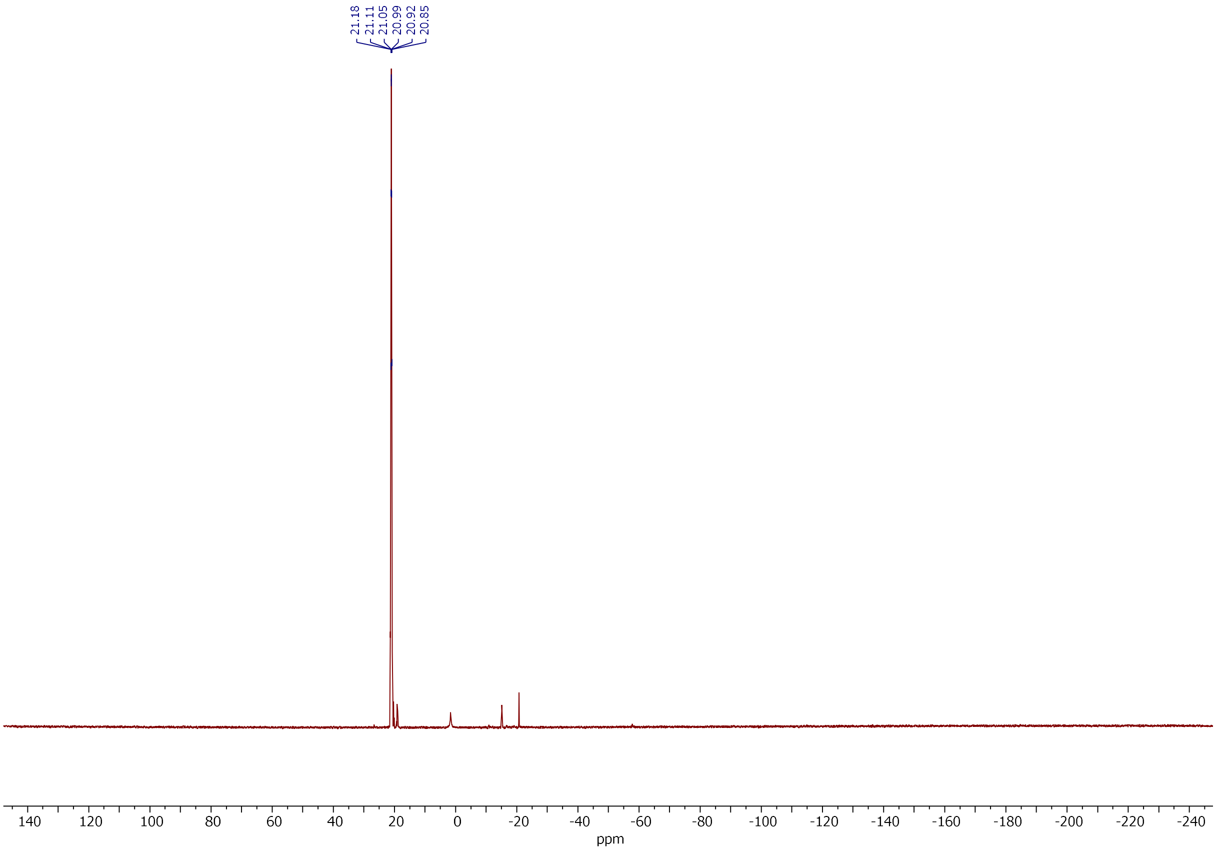

*Figure S55. ^{31}P NMR spectrum of the **1^{Cs}** (5 mol%) catalysed HP reaction of **2** with $^{t}Bu_2PH$ in benzene-d_6 at 300 K after 1 h at 75 °C. 96% conversion. Minor side products observed between -25-5 ppm.*

$^{n}Bu_2PH$

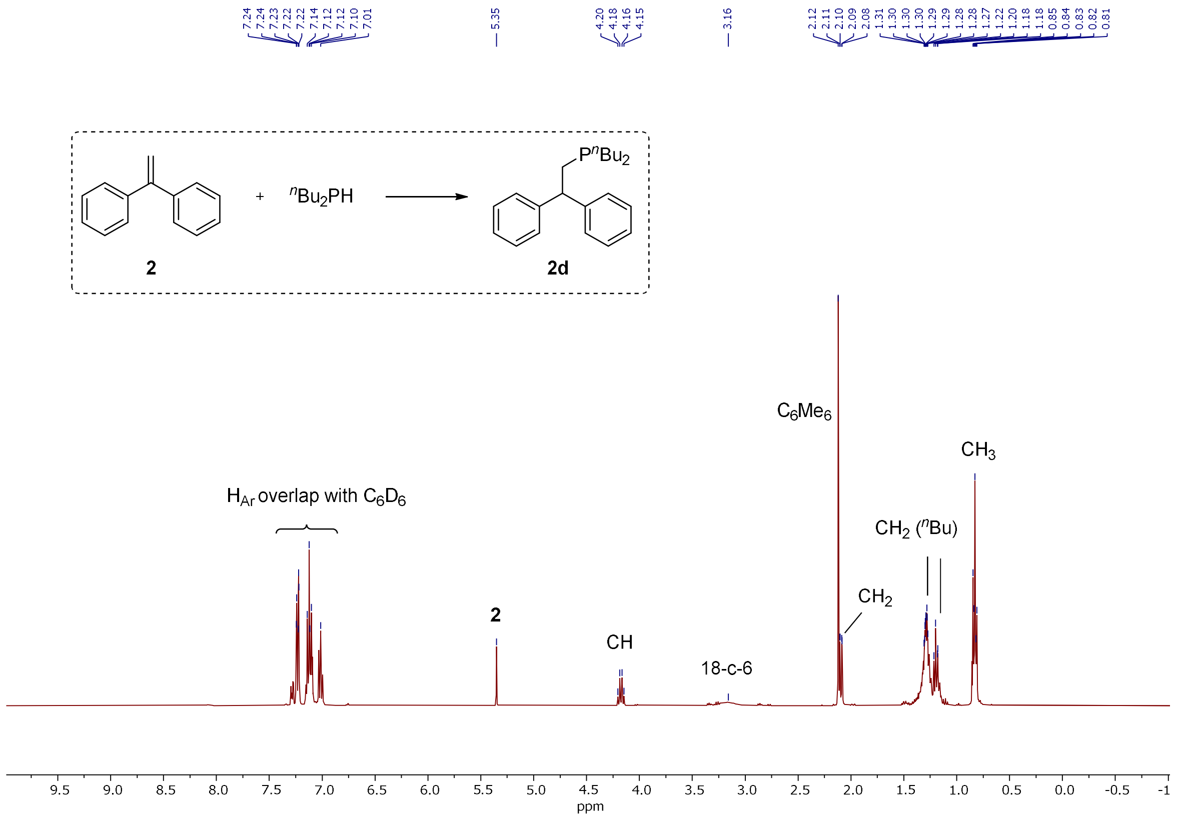

*Figure S56. ^{1}H NMR spectrum of the $\mathbf{1^{Cs}}$ (5 mol%) catalysed HP reaction of **2** with $^{n}Bu_2PH$ in benzene-d_6 at 300 K after 2.5 h at 75 °C. C_6Me_6 as internal standard. 86% conversion.*

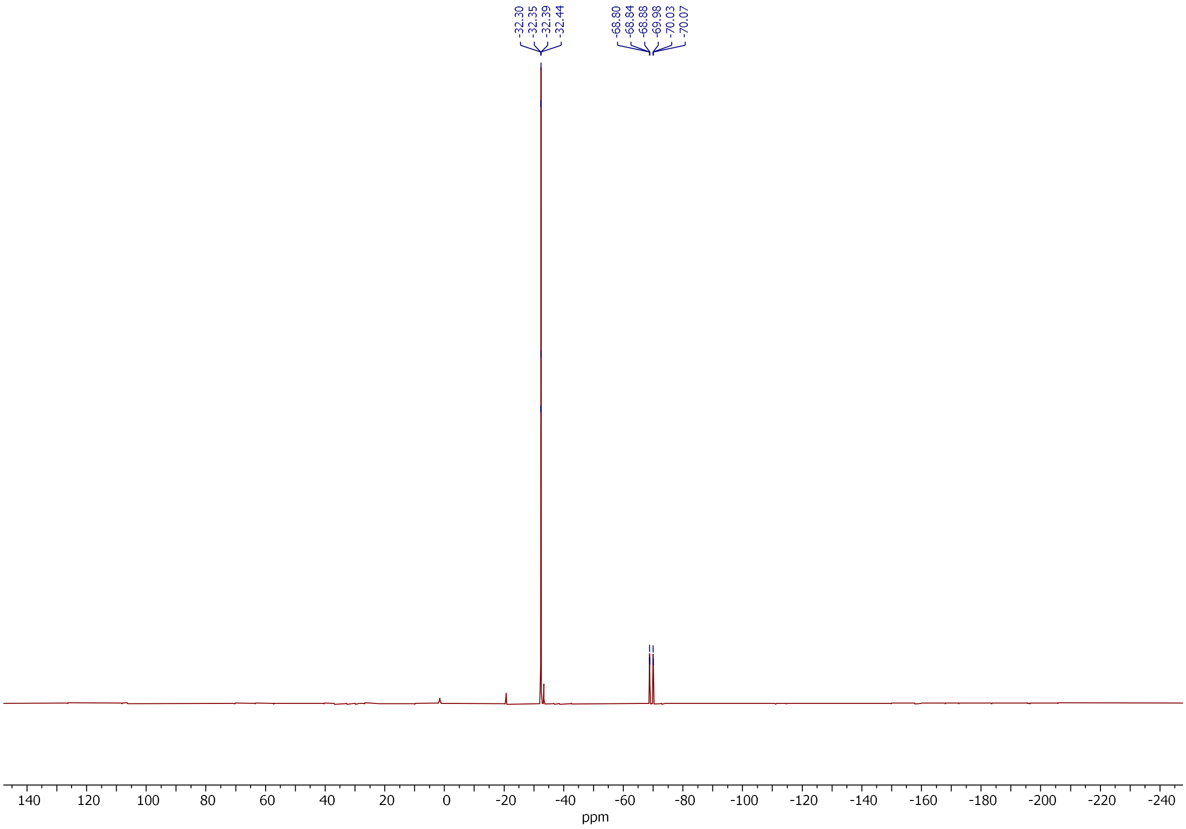

*Figure S57. ^{31}P NMR spectrum of the $\mathbf{1^{Cs}}$ (5 mol%) catalysed HP reaction of **2** with $^{n}Bu_2PH$ in benzene-d_6 at 300 K after 2.5 h at 75 °C. 86% conversion. nBu2PH (-70 ppm) and minor side products observed.*

$Cs[B(C_6F_5)_4]$ as catalyst

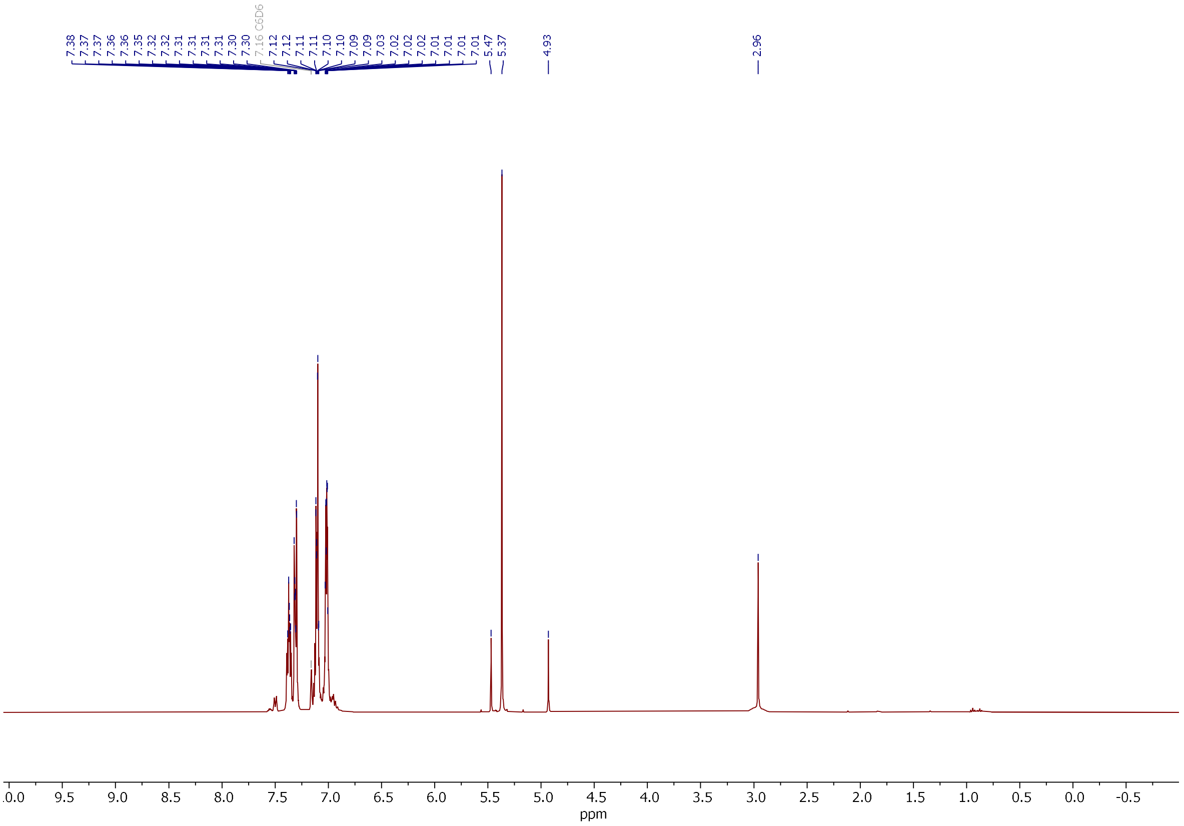

*Figure S58. ^{1}H NMR spectrum of the $Cs[B(C_6F_5)_4]$ (10 mol%) catalysed HP reaction of **2** with $^nBu_2PH$ in benzene-d_6 at 300 K after 3 h at RT. No conversion, mixture of **2** and Ph_2PH.*

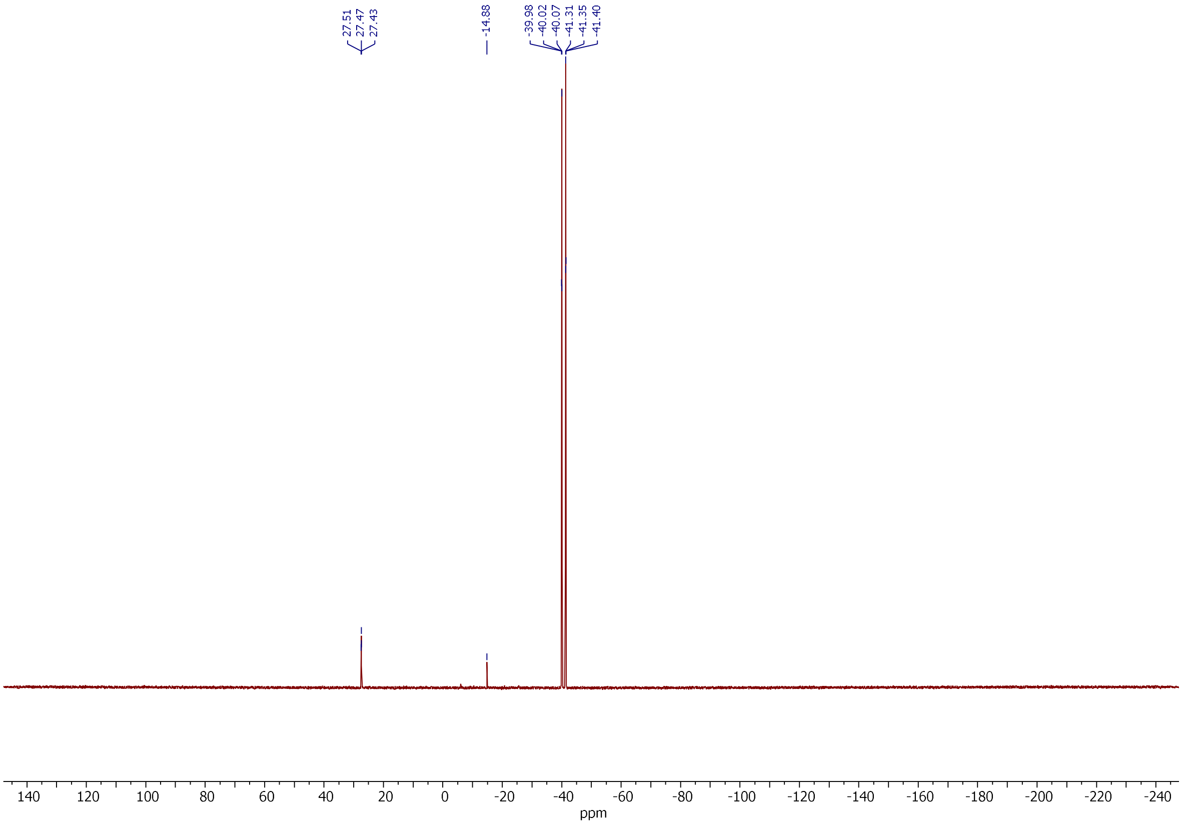

*Figure S59. ^{31}P NMR spectrum of the $Cs[B(C_6F_5)_4]$ (10 mol%) catalysed HP reaction of **2** with $^nBu_2PH$ in benzene-d_6 at 300 K after 3 h at RT. No conversion, mainly Ph_2PH.*

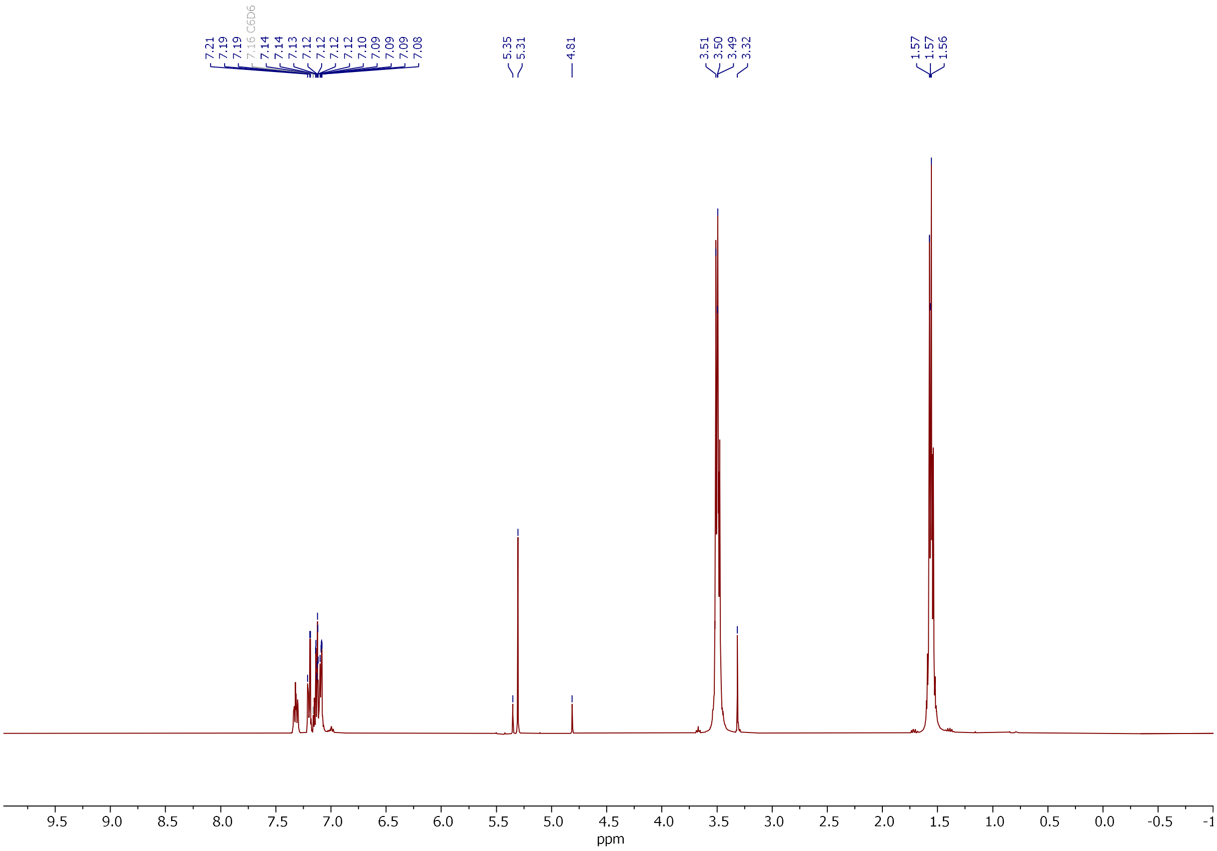

*Figure S60. ^{1}H NMR spectrum of the $Cs[B(C_6F_5)_4]$ (5 mol%) catalysed HP reaction of **2** with $^{n}Bu_2PH$ in THF:benzene-d_6 1:1 at 300 K after 20 h at RT. No conversion, mixture of **2** and Ph_2PH.*

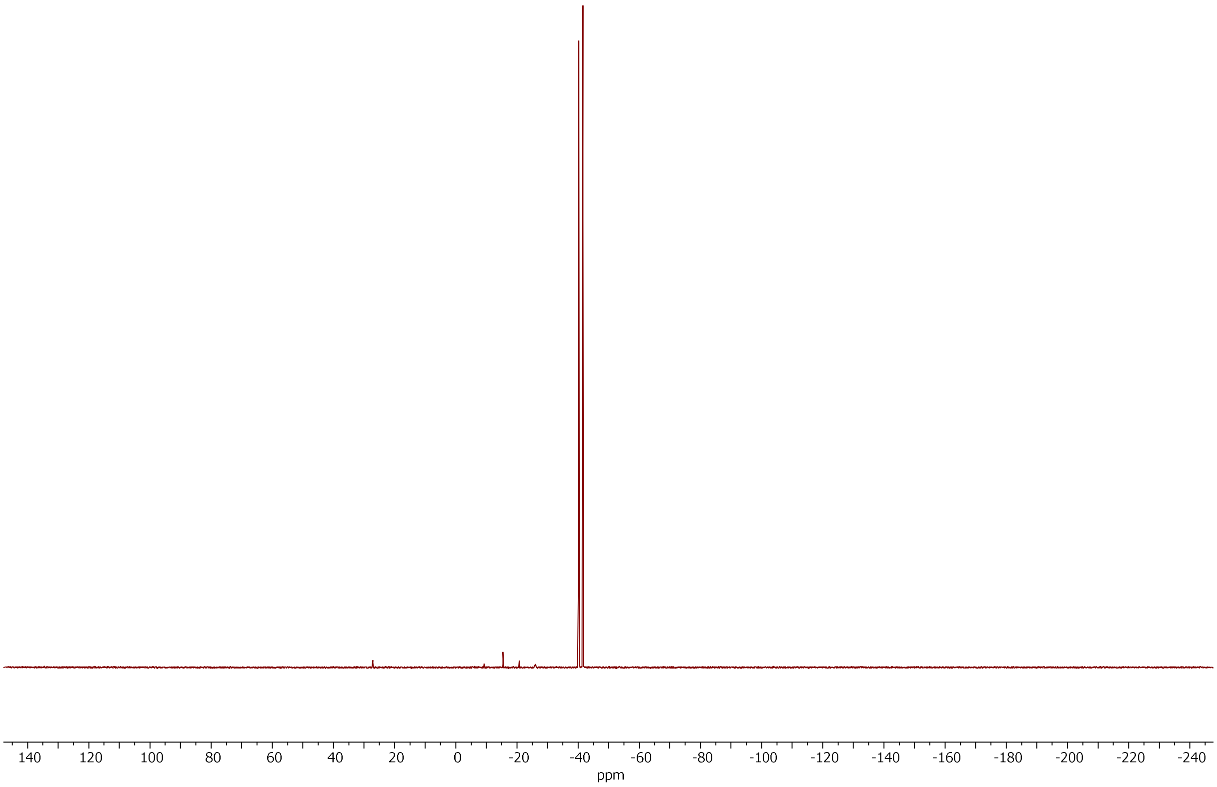

*Figure S61. ^{31}P NMR spectrum of the $Cs[B(C_6F_5)_4]$ (5 mol%) catalysed HP reaction of **2** with $^{n}Bu_2PH$ in THF:benzene-d_6 1:1 at 300 K after 20 h at RT. No conversion, almost pure Ph_2PH.*

Section S3.2 – NMR Spectra of the catalytic hydrophosphination reactions of alkenes 3-7

Ph_2PH

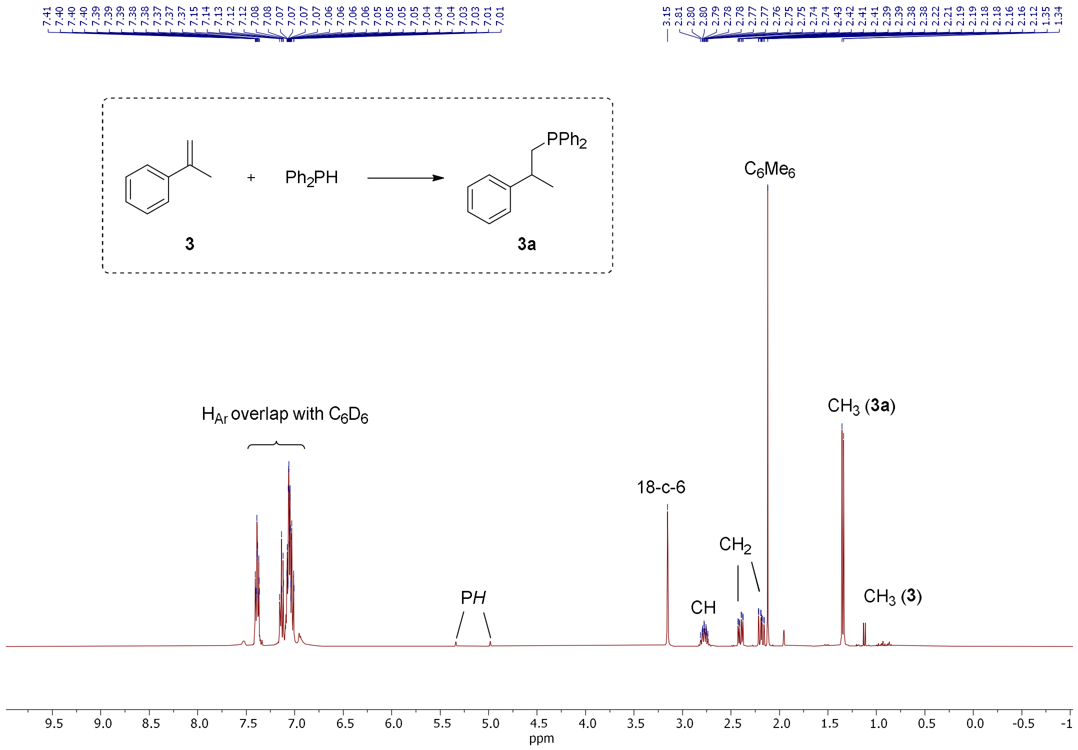

*Figure S62. ^1H NMR spectrum of the **1**^{Cs} (5 mol%) catalysed HP reaction of **3** with Ph_2PH in benzene-d_6 at 300 K after 2 h at 90 °C. C_6Me_6 as internal standard, 98 % conversion.*

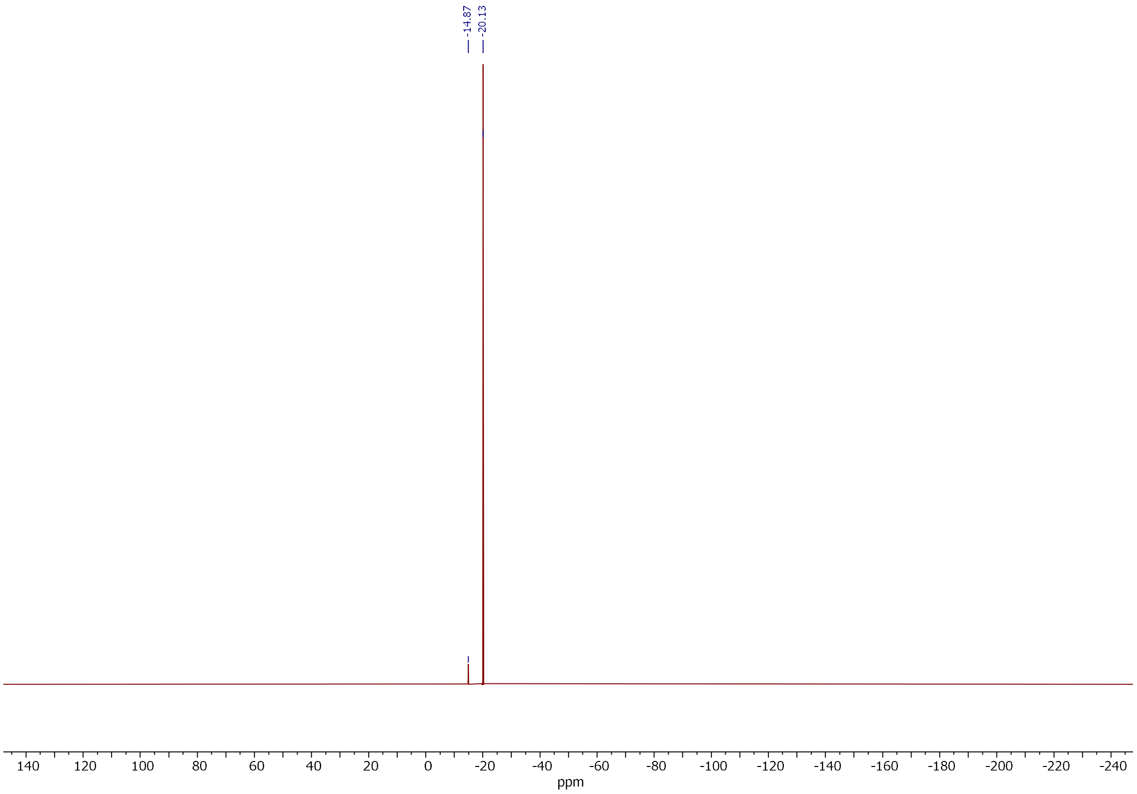

*Figure S63. ^{31}P NMR spectrum of the **1**^{Cs} (5 mol%) catalysed HP reaction of **3** with Ph_2PH in benzene-d_6 at 300 K after 2 h at 90 °C. 98 % conversion minor unknown side product visible at -15 ppm.*

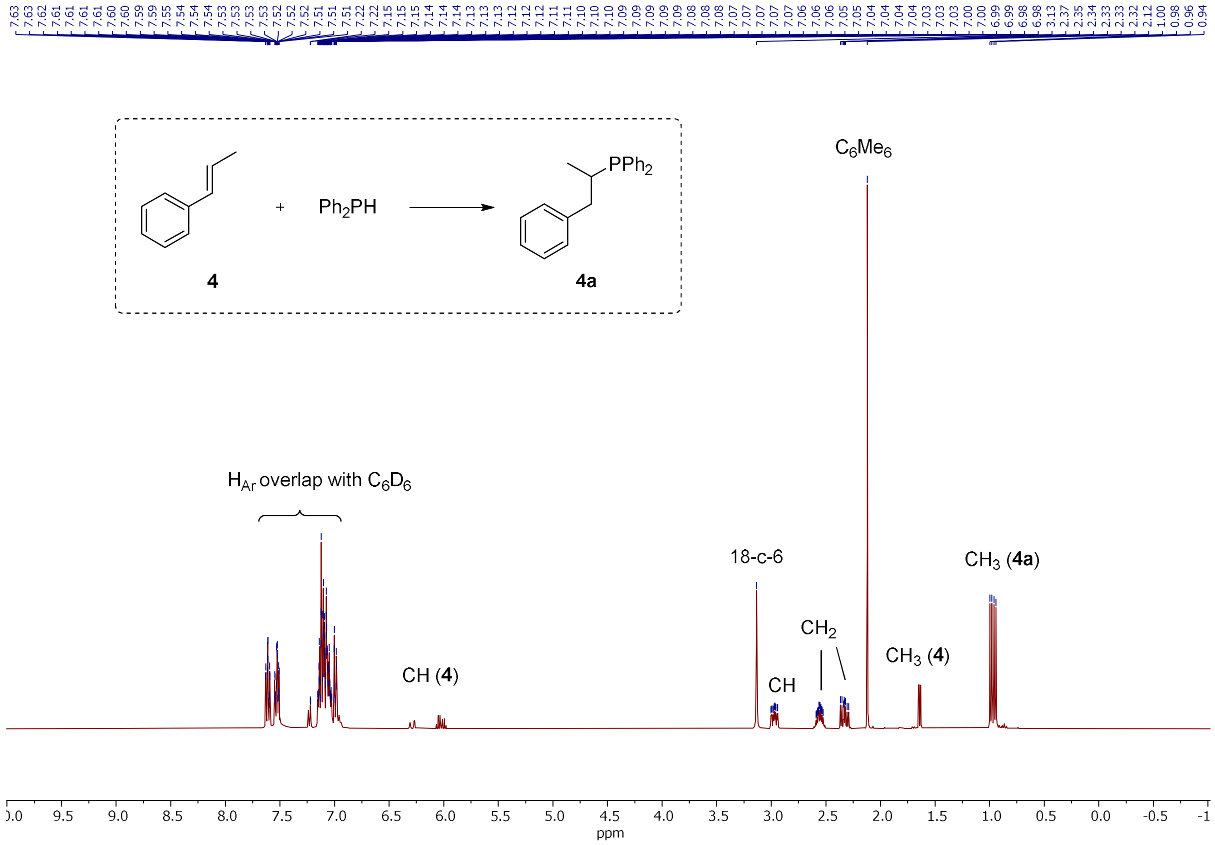

*Figure S64. ^{1}H NMR spectrum of the **1**^{Cs} (5 mol%) catalysed HP reaction of **4** with Ph_2PH in benzene-d_6 at 300 K after 2 h at 90 °C. C_6Me_6 as internal standard, 90 % conversion.*

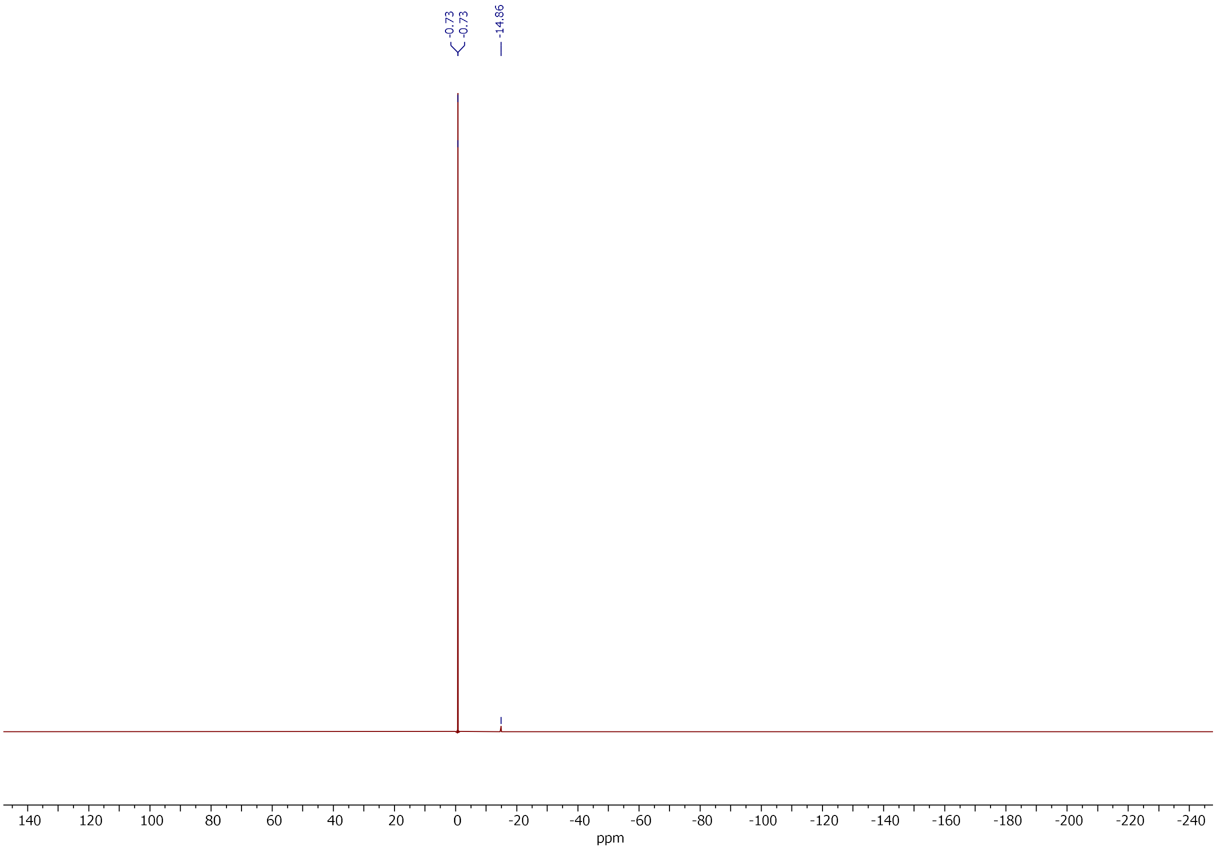

*Figure S65. ^{31}P NMR spectrum of the **1**^{Cs} (5 mol%) catalysed HP reaction of **4** with Ph_2PH in benzene-d_6 at 300 K after 2 h at 90 °C. Full conversion of phosphine, minor unknown side product visible at -15 ppm.*

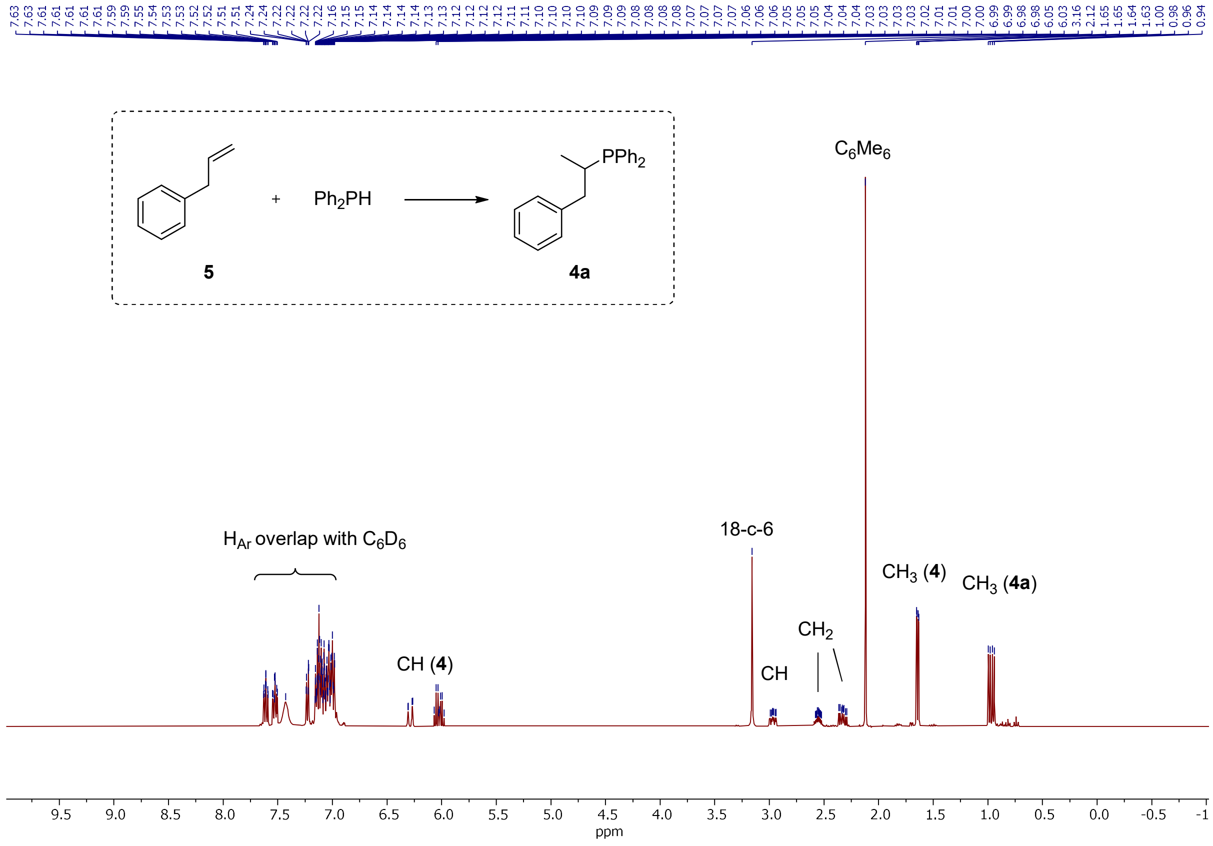

*Figure S66. ^{1}H NMR spectrum of the **1**[Cs] (5 mol%) catalysed HP reaction of **5** with Ph_2PH in benzene-d_6 at 300 K after 2 h at 90 °C. C_6Me_6 as internal standard, 50 % conversion to **4a** after initial isomerization of **5** to **4** and subsequent HP.*

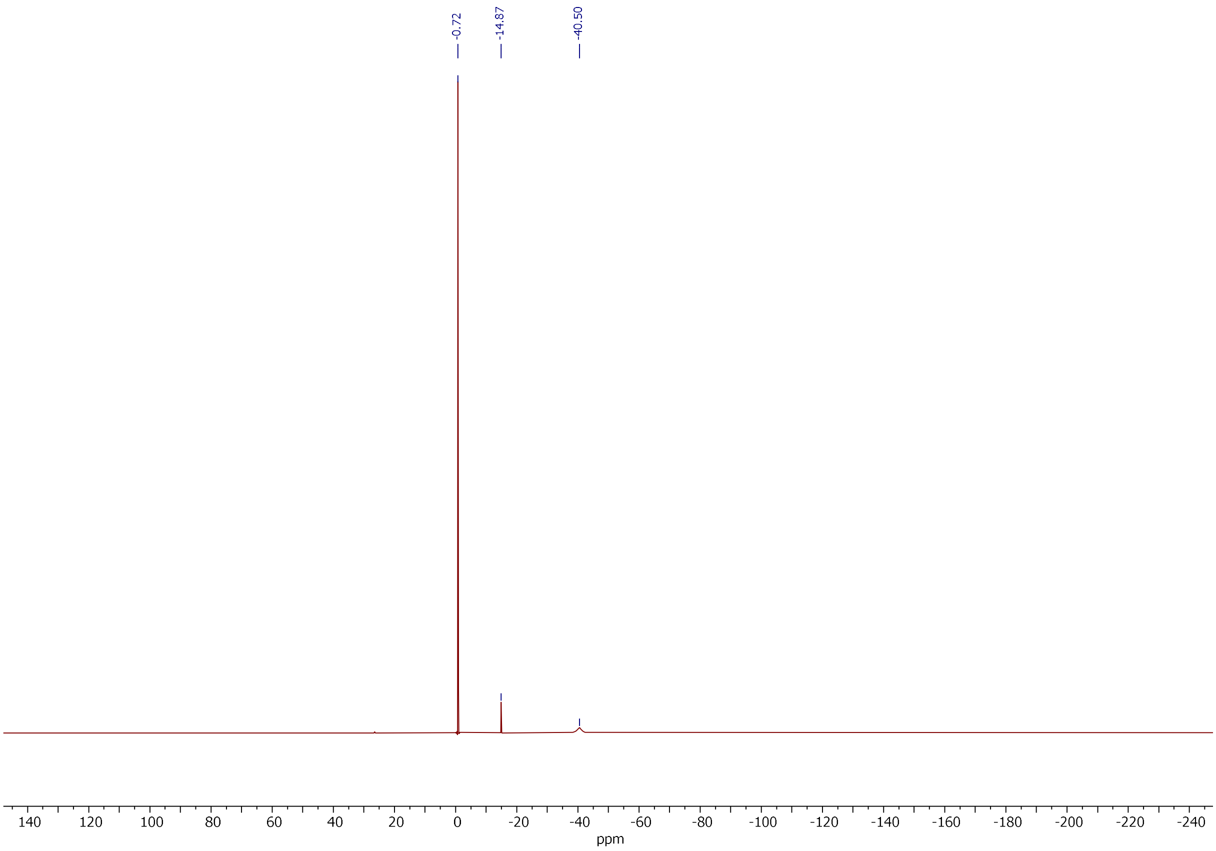

*Figure S67. ^{31}P NMR spectrum of the **1**[Cs] (5 mol%) catalysed HP reaction of **5** with Ph_2PH in benzene-d_6 at 300 K after 2 h at 90 °C. 50% conversion of phosphine (Ph_2PH at -40 ppm), minor unknown side product visible at -15 ppm.*

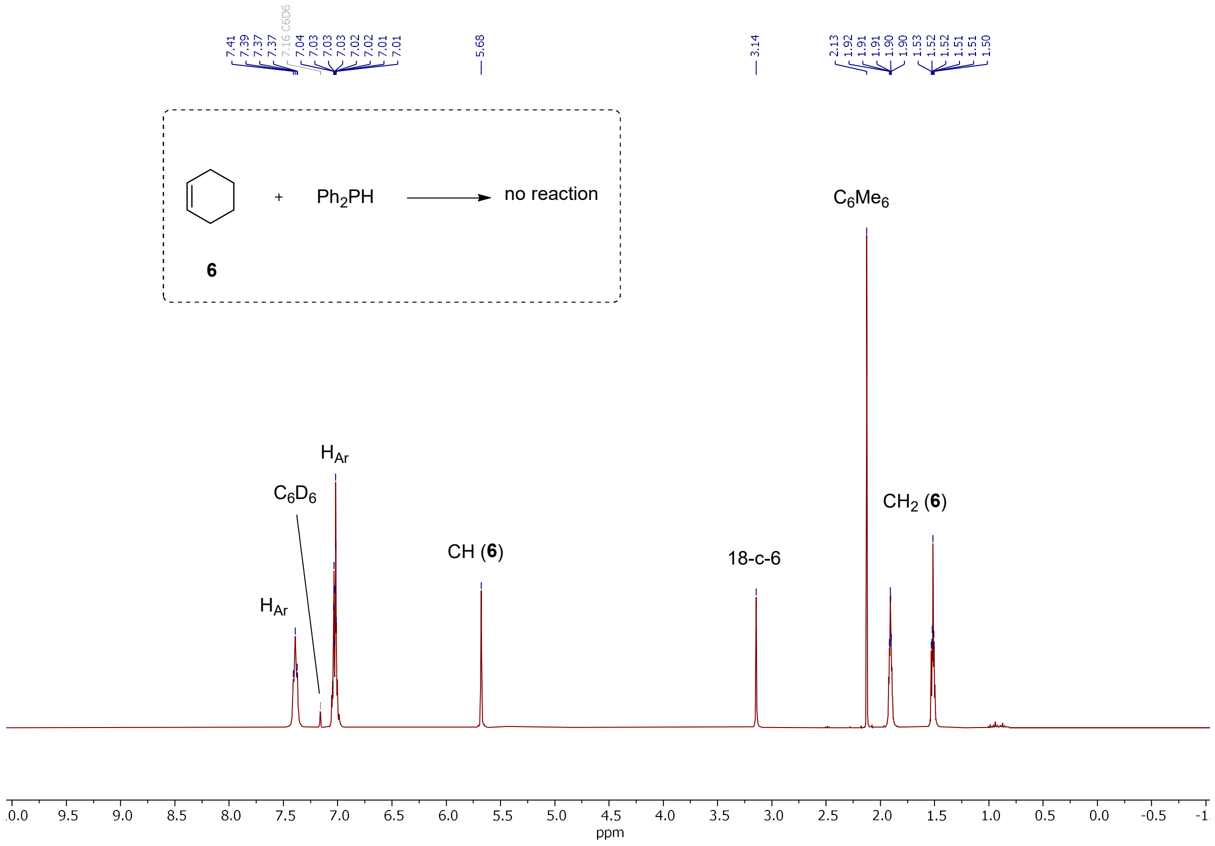

Figure S68. *^{1}H NMR spectrum of the* ***1***[Cs] *(5 mol%) catalysed HP reaction of* ***6*** *with Ph_2PH in benzene-d_6 at 300 K after 24 h at 90 °C. C_6Me_6 as internal standard, no conversion.*

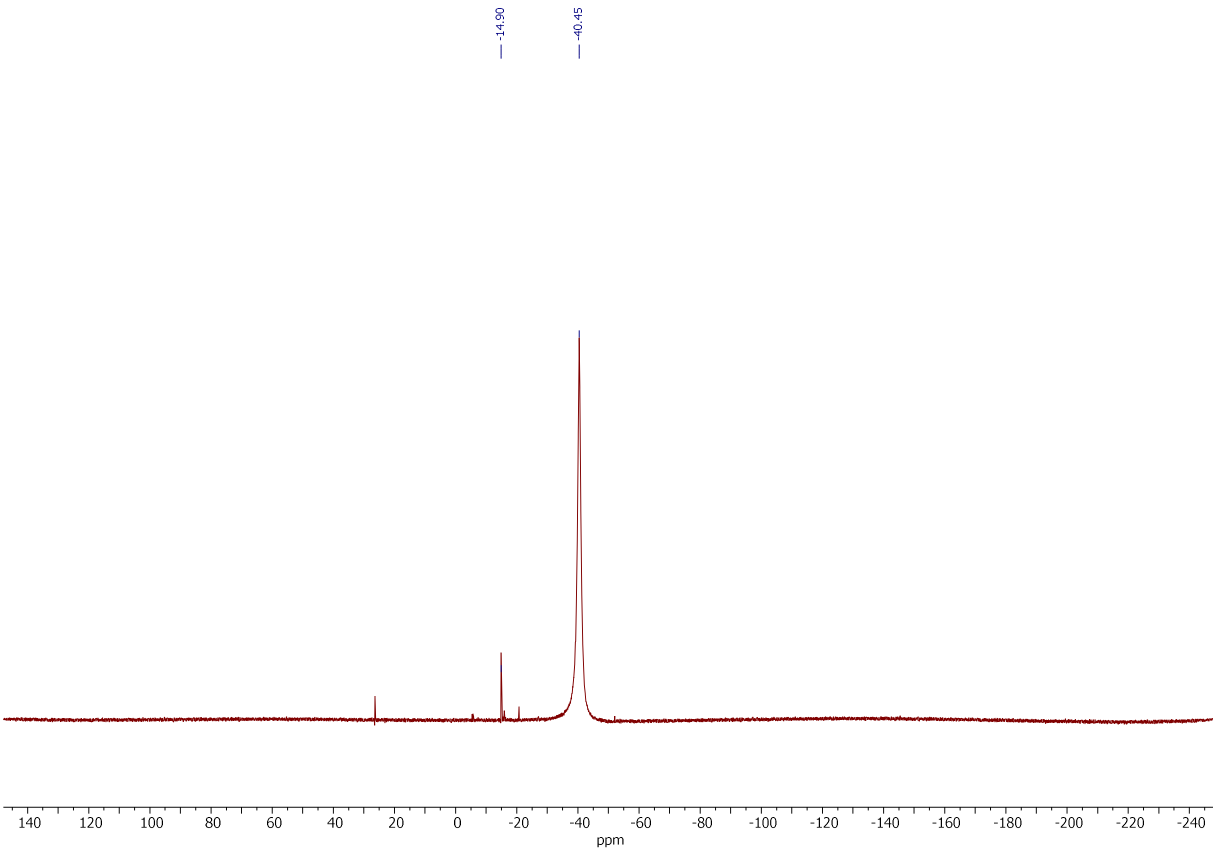

Figure S69. *^{31}P NMR spectrum of the* ***1***[Cs] *(5 mol%) catalysed HP reaction of* ***6*** *with Ph_2PH in benzene-d_6 at 300 K after 24 h at 90 °C, no conversion.*

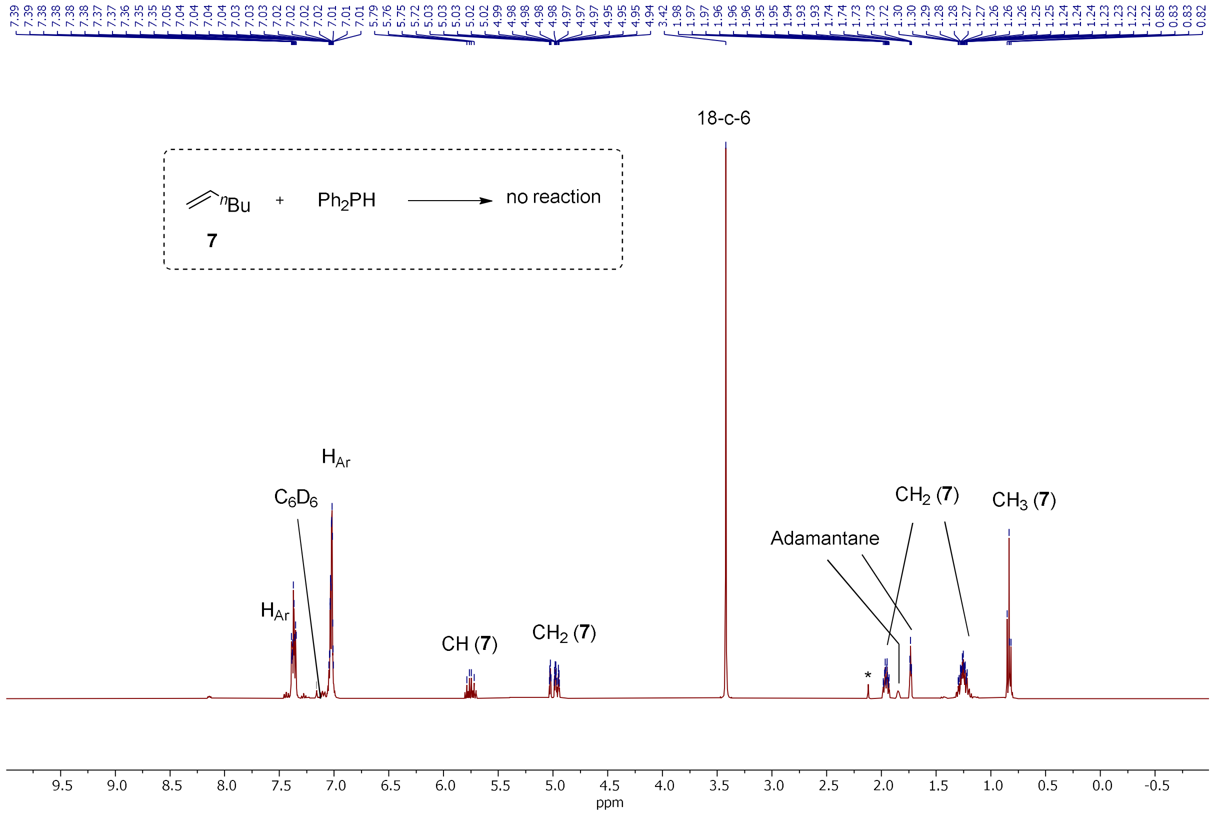

*Figure S70. ^1H NMR spectrum of the $\mathbf{1^{Cs}}$ (5 mol%) catalysed HP reaction of **7** with Ph_2PH in benzene-d_6 at 300 K after 20 h at 90 °C. Adamantane as internal standard, no conversion.*

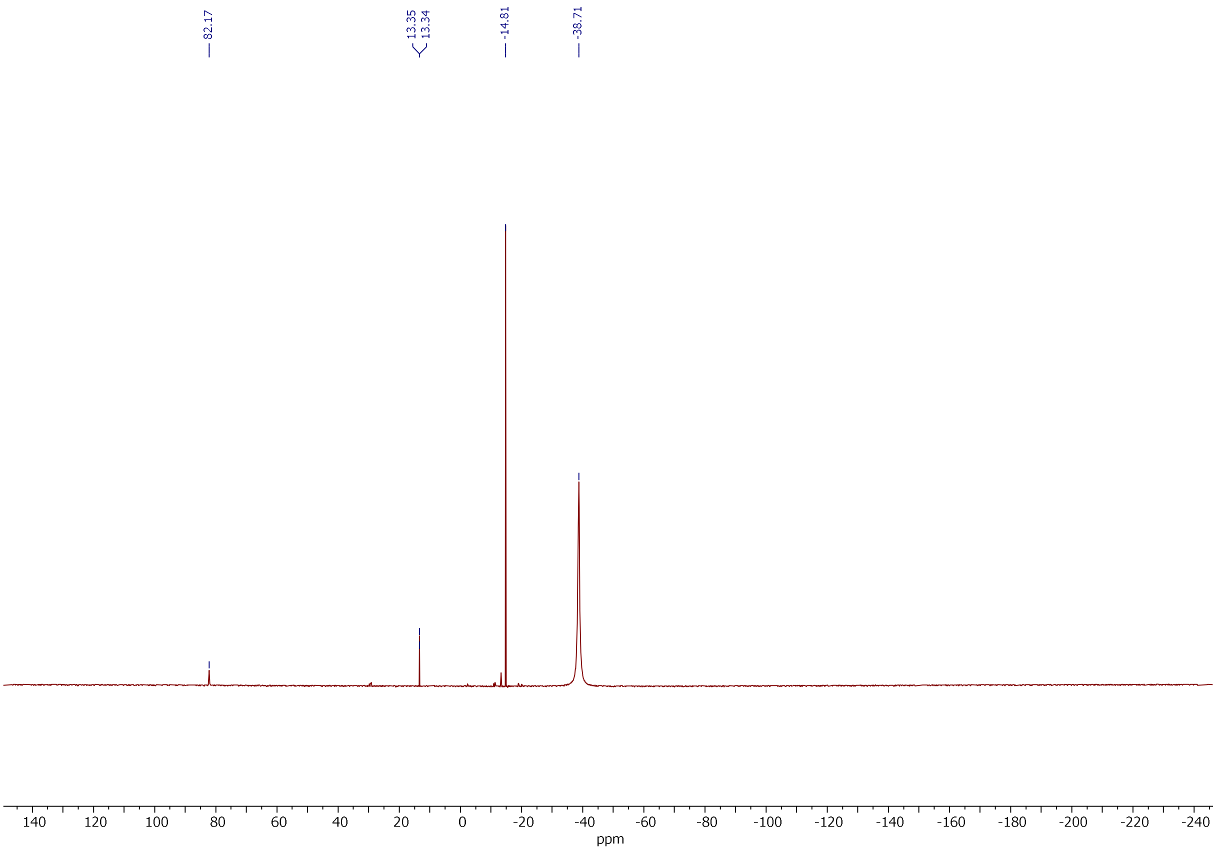

*Figure S71. ^{31}P NMR spectrum of the $\mathbf{1^{Cs}}$ (5 mol%) catalysed HP reaction of **7** with Ph_2PH in benzene-d_6 at 300 K after 20 h at 90 °C, no conversion.*

^{t}BuPhPH

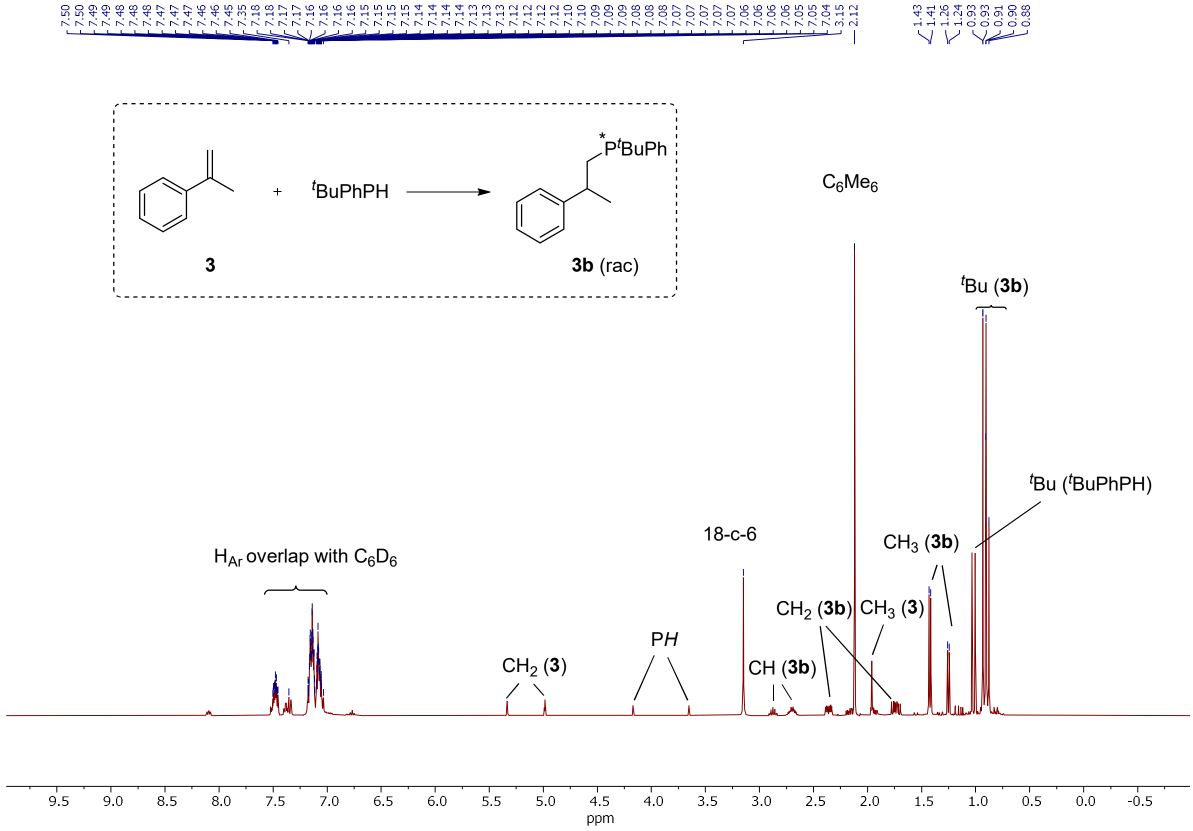

*Figure S72. ^{1}H NMR spectrum of the **1^{Cs}** (5 mol%) catalysed HP reaction of **3** with ^{t}BuPhPH in benzene-d_6 at 300 K after 6 h at 90 °C. C_6Me_6 as internal standard, 74 % conversion, mixture of isomers (**3b** rac).*

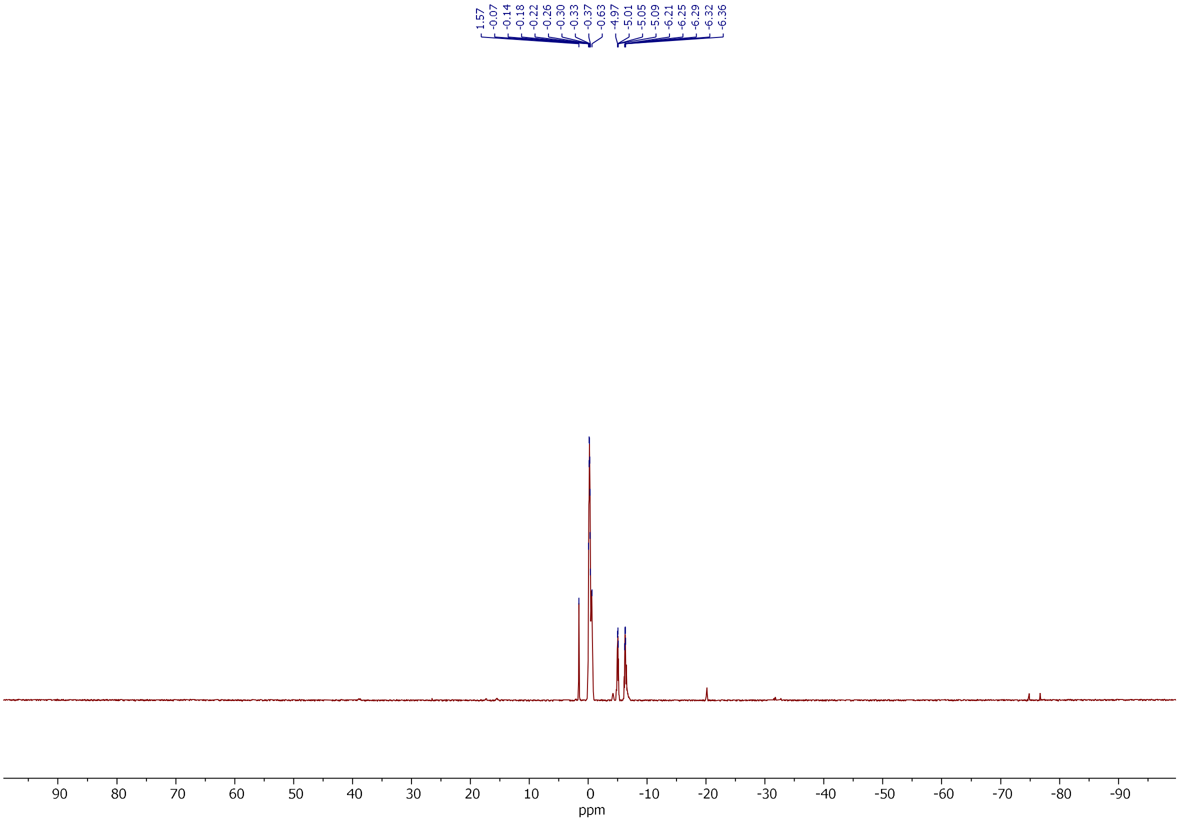

*Figure S73. ^{31}P NMR spectrum of the **1^{Cs}** (5 mol%) catalysed HP reaction of **3** with ^{t}BuPhPH in benzene-d_6 at 300 K after 6 h at 90 °C. 74 % conversion, mixture of isomers (**3b** rac 0 ppm), ^{t}BuPhPH (5.5 ppm) and **1^{Cs}** (1.6 ppm).*

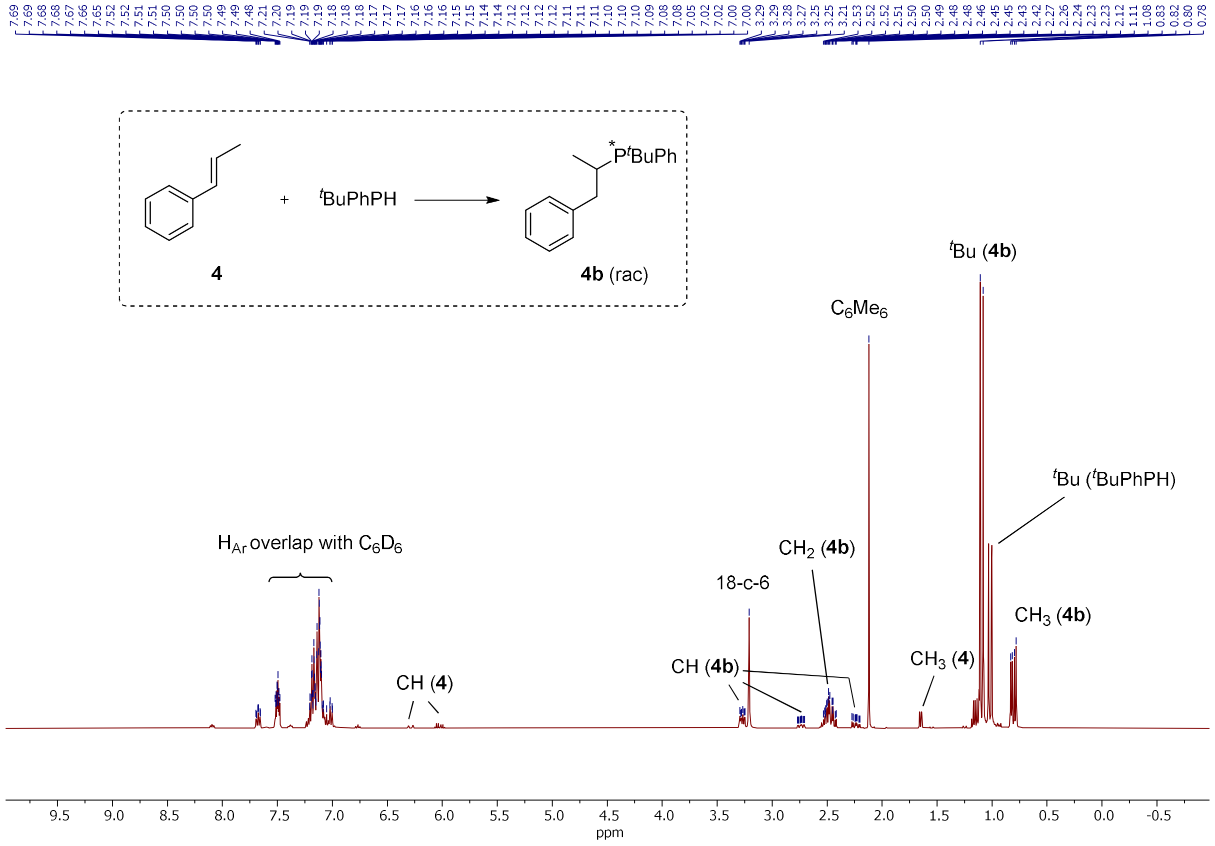

*Figure S74. ^{1}H NMR spectrum of the $\mathbf{1^{Cs}}$ (5 mol%) catalysed HP reaction of **4** with ^{t}BuPhPH in benzene-d_6 at 300 K after 2 h at 90 °C. C_6Me_6 as internal standard, 81 % conversion, mixture of isomers (**4b** rac).*

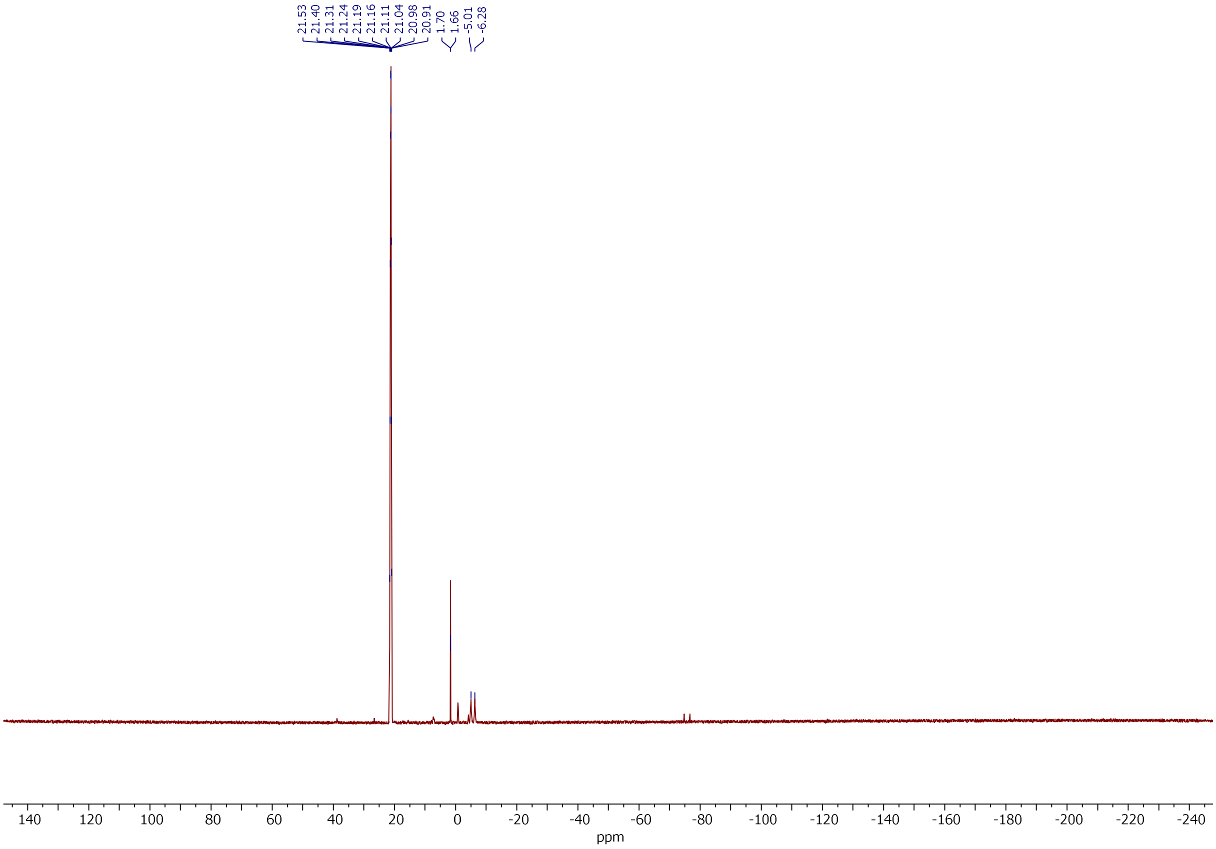

*Figure S75. ^{31}P NMR spectrum of the $\mathbf{1^{Cs}}$ (5 mol%) catalysed HP reaction of **4** with ^{t}BuPhPH in benzene-d_6 at 300 K after 2 h at 90 °C. 81 % conversion, mixture of isomers (**4b** rac 21 ppm), ^{t}BuPhPH (5.5 ppm) and $\mathbf{1^{Cs}}$ (1.7 ppm).*

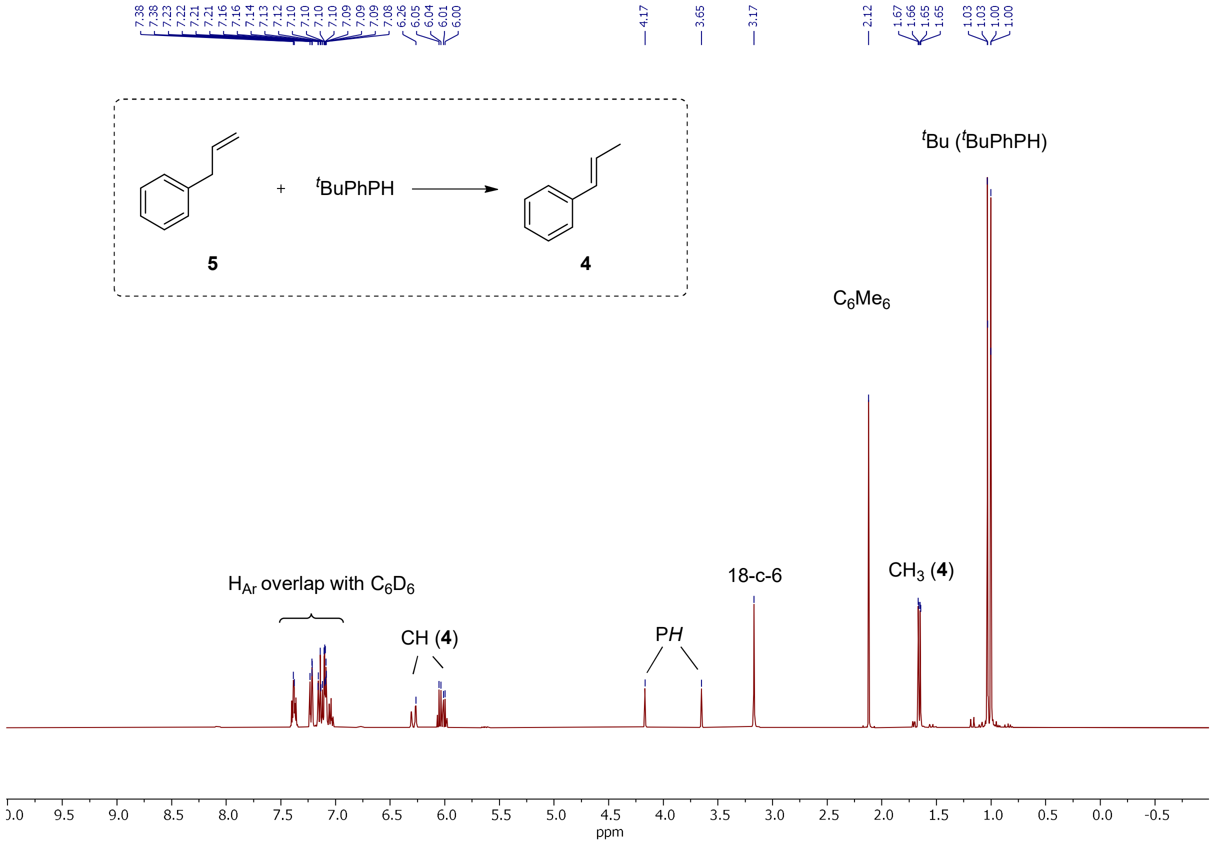

*Figure S76. ^1H NMR spectrum of the $\mathbf{1^{Cs}}$ (5 mol%) catalysed HP reaction of **5** with ^tBuPhPH in benzene-d_6 at 300 K after 20 h at 90 °C. C_6Me_6 as internal standard. Full conversion, but isomerisation from **5** into **4** instead of HP.*

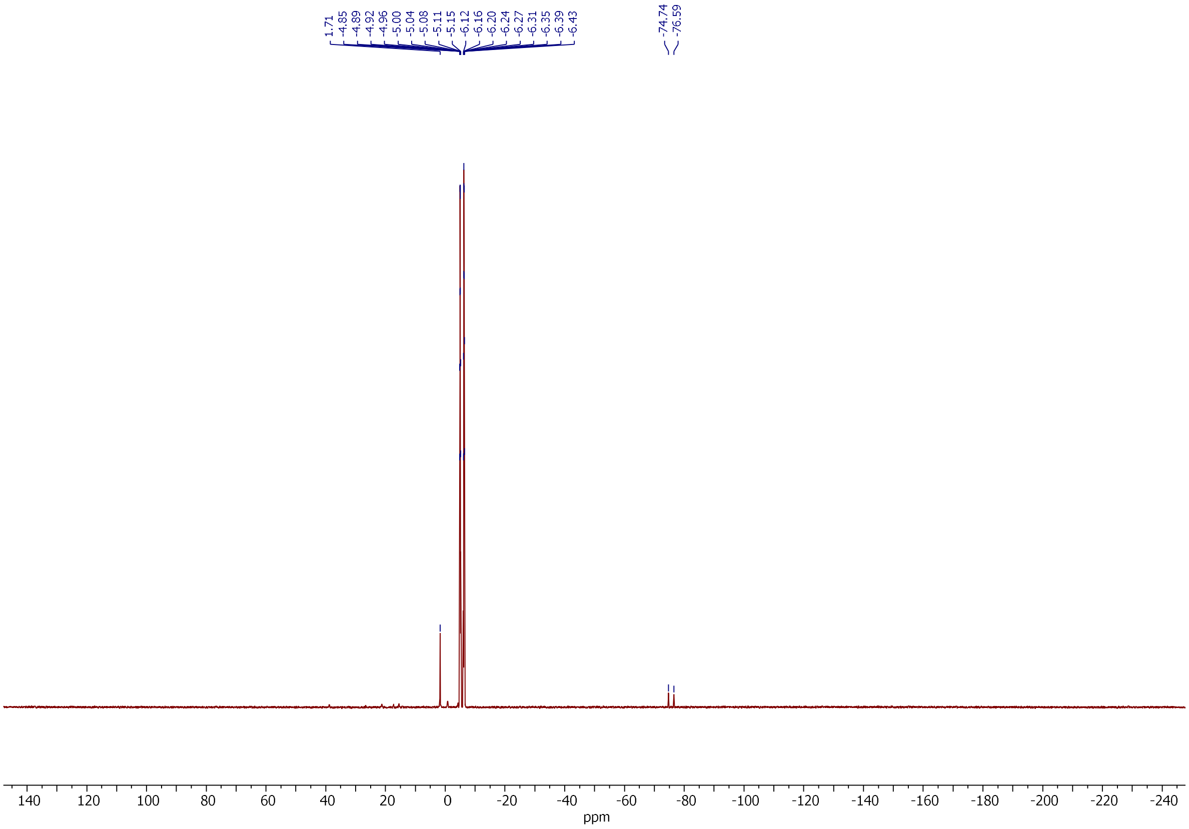

*Figure S77. ^1H NMR spectrum of the $\mathbf{1^{Cs}}$ (5 mol%) catalysed HP reaction of **5** with ^tBuPhPH in benzene-d_6 at 300 K after 20 h at 90 °C. C_6Me_6 as internal standard. Full conversion, but isomerisation from **5** into **4** instead of HP. Mixture of ^tBuPhPH (5.5 ppm), $\mathbf{1^{Cs}}$ (1.7 ppm) and unknown impurity (-75 ppm).*

$^{t}Bu_2PH$

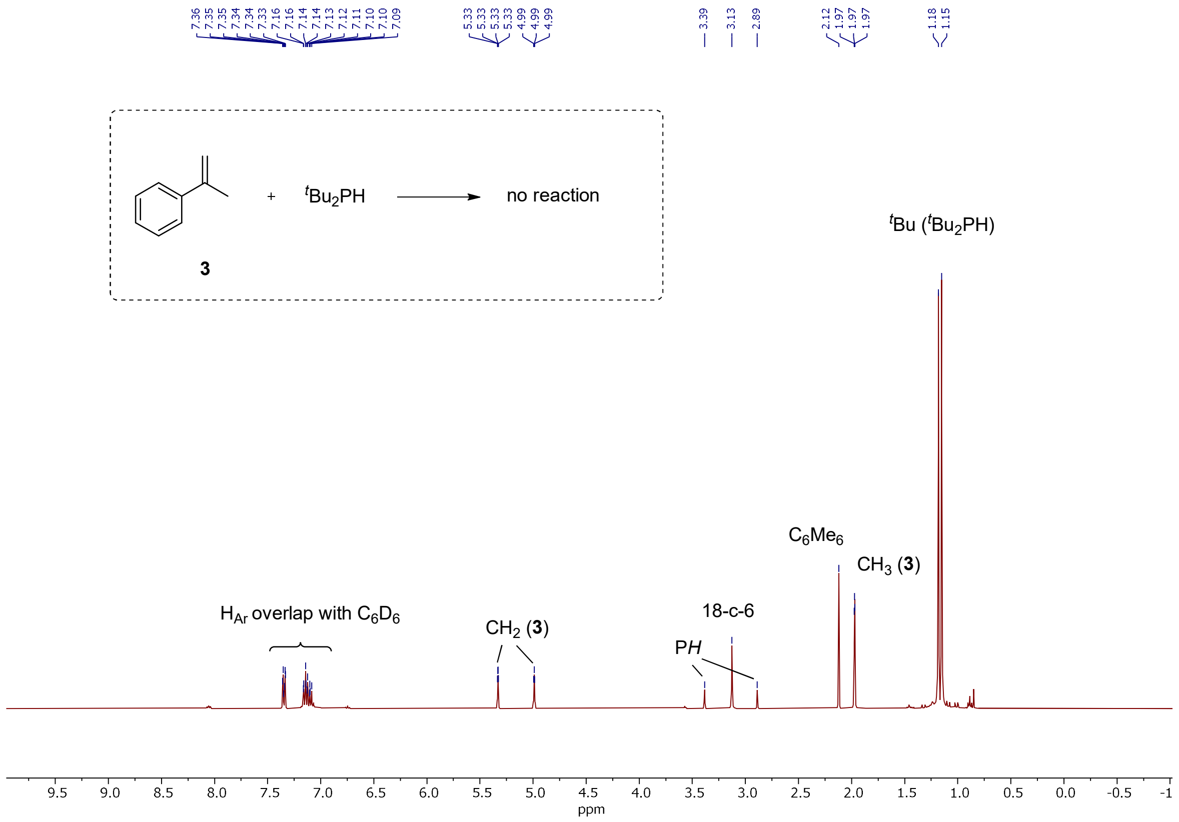

*Figure S78. ^{1}H NMR spectrum of the **1**^{Cs} (5 mol%) catalysed HP reaction of **3** with $^{t}Bu_2PH$ in benzene-d_6 at 300 K after 22 h at 90 °C. C_6Me_6 as internal standard. No conversion, mixture of substrates.*

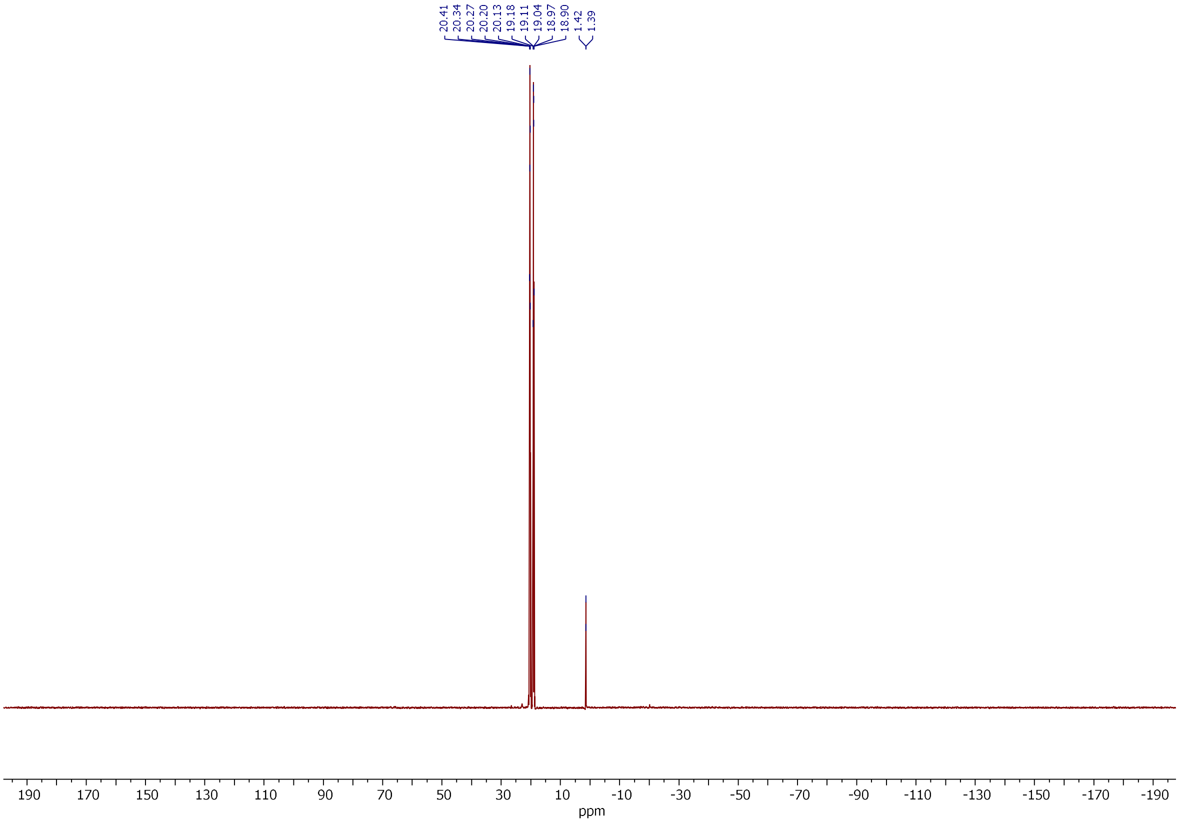

*Figure S79. ^{31}P NMR spectrum of the **1**^{Cs} (5 mol%) catalysed HP reaction of **3** with $^{t}Bu_2PH$ in benzene-d_6 at 300 K after 22 h at 90 °C. C_6Me_6 as internal standard. No conversion, mixture of $^{t}Bu_2PH$ (20 ppm) and **1**^{Cs} (1.4 ppm).*

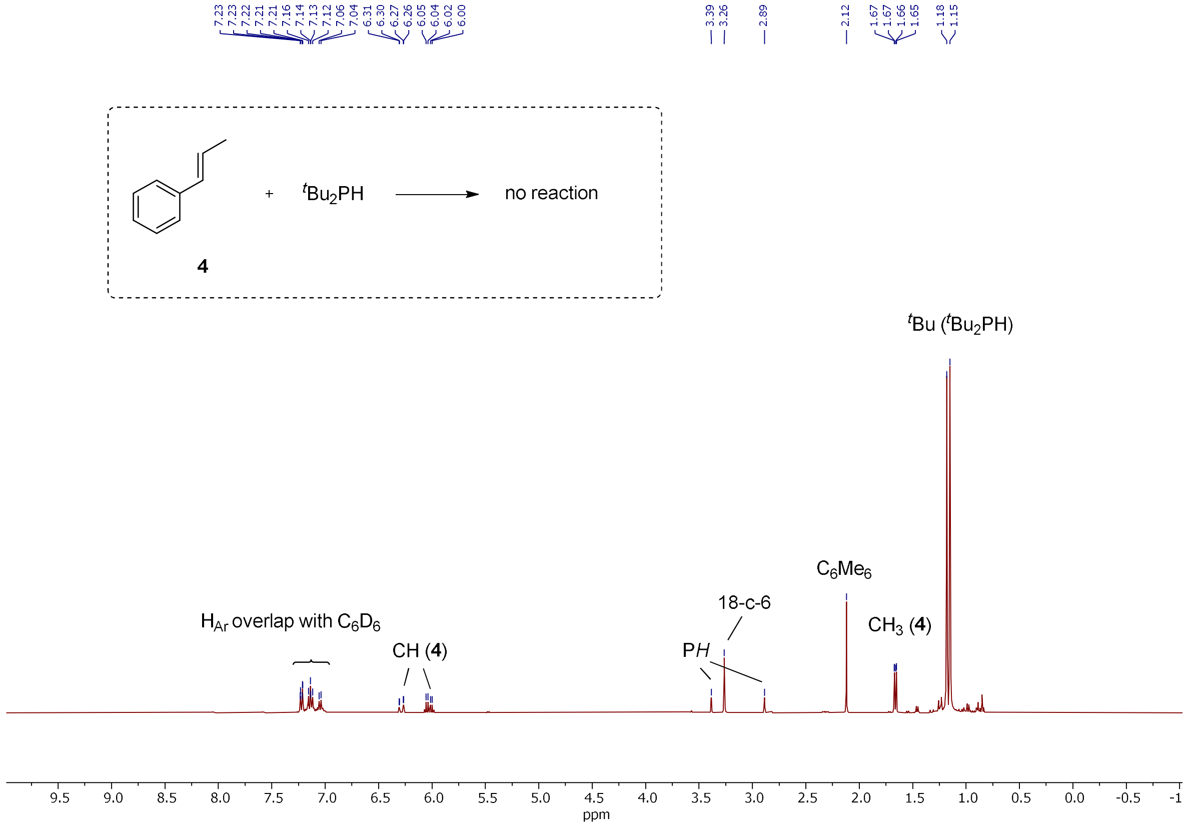

*Figure S80. ^{1}H NMR spectrum of the **1**^{Cs} (5 mol%) catalysed HP reaction of **4** with $^{t}Bu_2PH$ in benzene-d_6 at 300 K after 22 h at 90 °C. C_6Me_6 as internal standard. No conversion, mixture of substrates.*

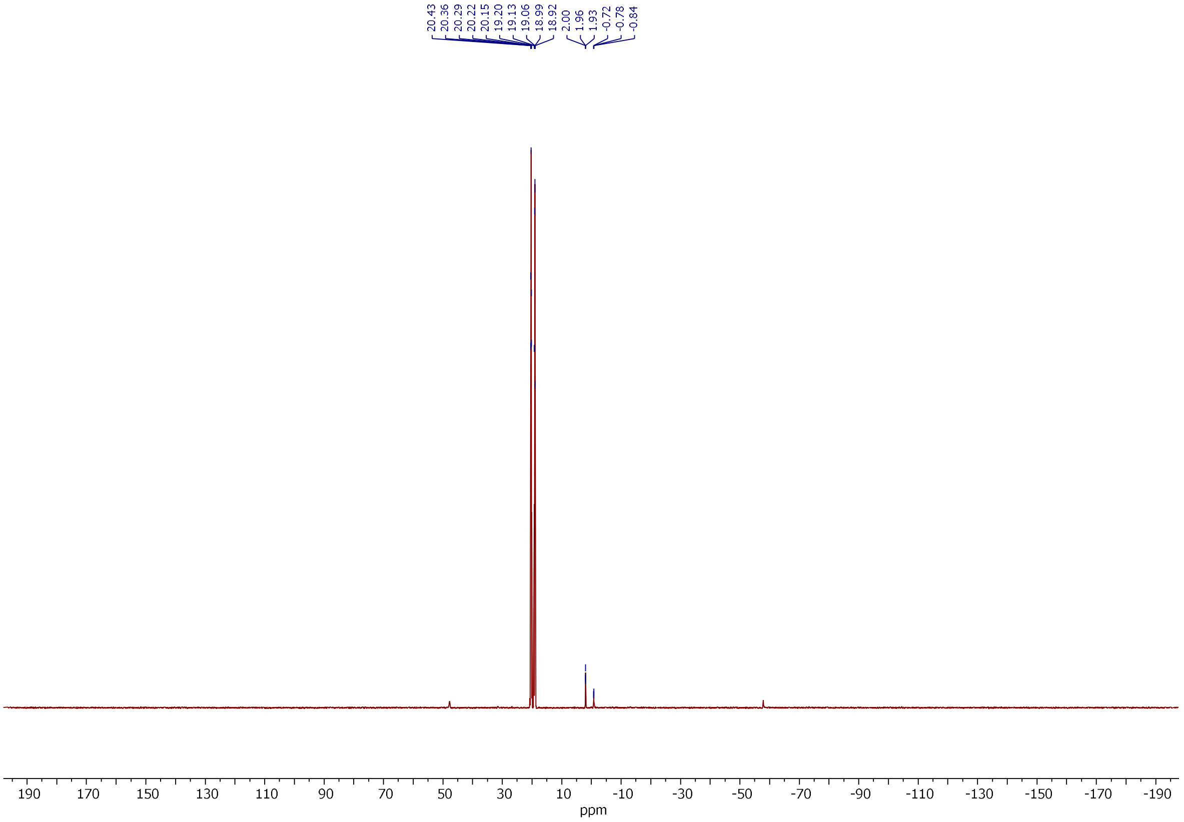

*Figure S81. ^{31}P NMR spectrum of the **1**^{Cs} (5 mol%) catalysed HP reaction of **4** with $^{t}Bu_2PH$ in benzene-d_6 at 300 K after 22 h at 90 °C. C_6Me_6 as internal standard. No conversion, mixture of $^{t}Bu_2PH$ (20 ppm),**1**^{Cs} (1.9 ppm) and a minor unknown impurity (-0.8).*

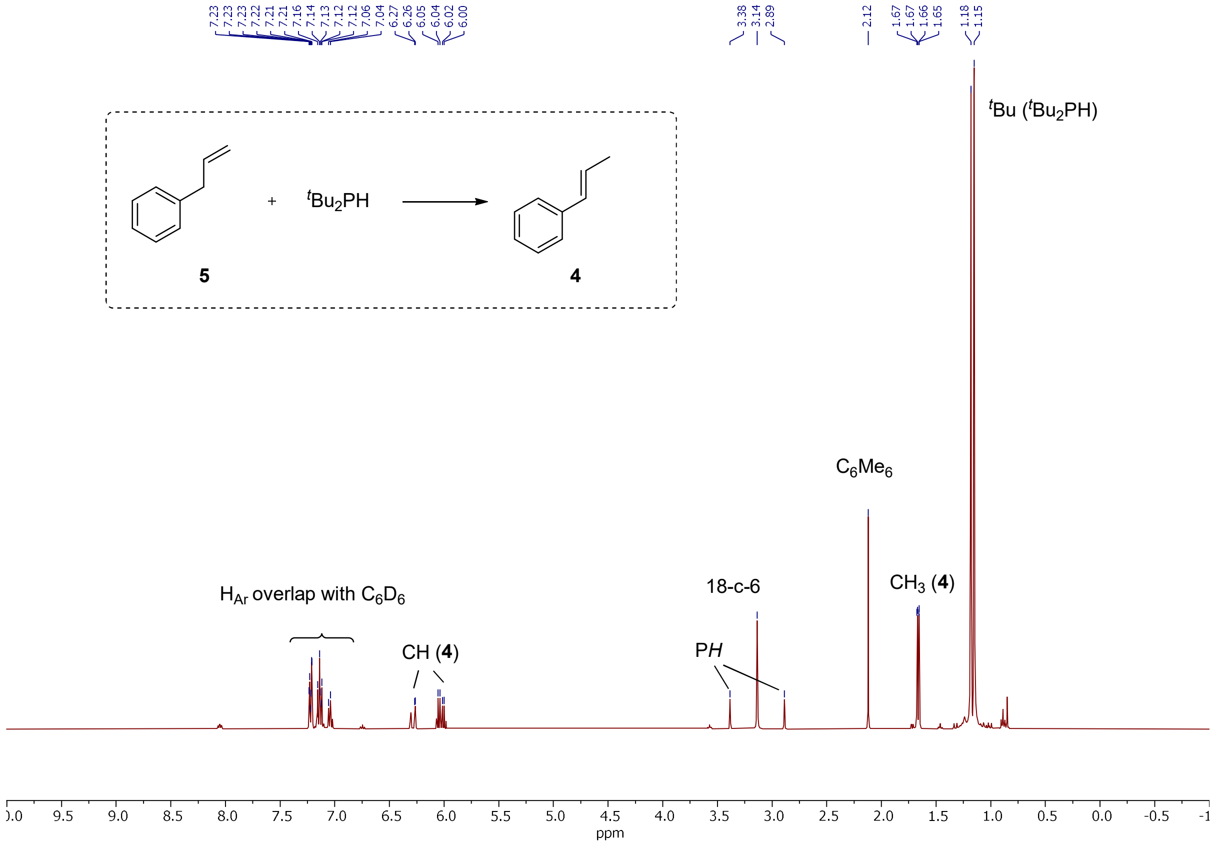

*Figure S82. ^{1}H NMR spectrum of the $\mathbf{1}^{Cs}$ (5 mol%) catalysed HP reaction of **5** with $^{t}Bu_2PH$ in benzene-d_6 at 300 K after 22 h at 90 °C. C_6Me_6 as internal standard. Full conversion, but isomerisation from **5** into **4** instead of HP.*

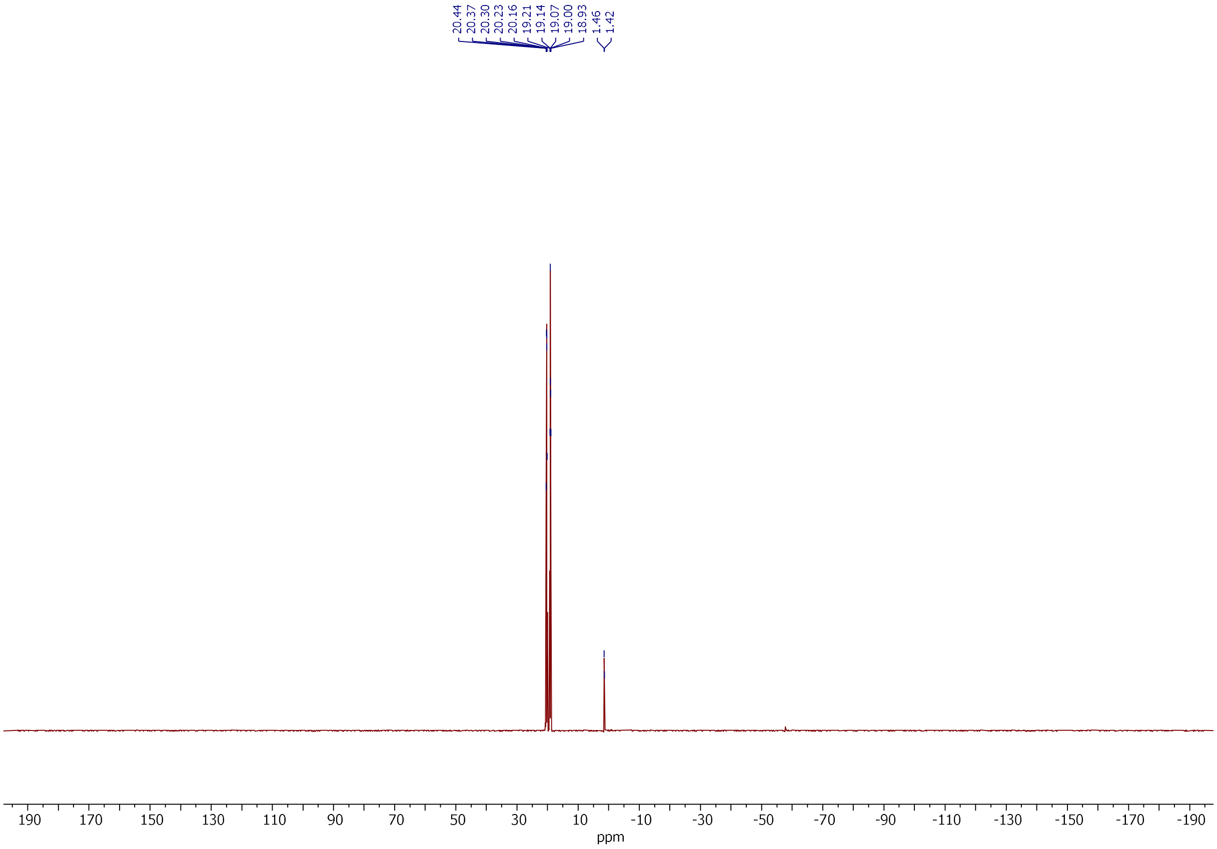

*Figure S83. ^{31}P NMR spectrum of the $\mathbf{1}^{Cs}$ (5 mol%) catalysed HP reaction of **5** with $^{t}Bu_2PH$ in benzene-d_6 at 300 K after 22 h at 90 °C. C_6Me_6 as internal standard. No conversion, mixture of $^{t}Bu_2PH$ (20 ppm) and $\mathbf{1}^{Cs}$ (1.4 ppm).*

$^{n}Bu_2PH$

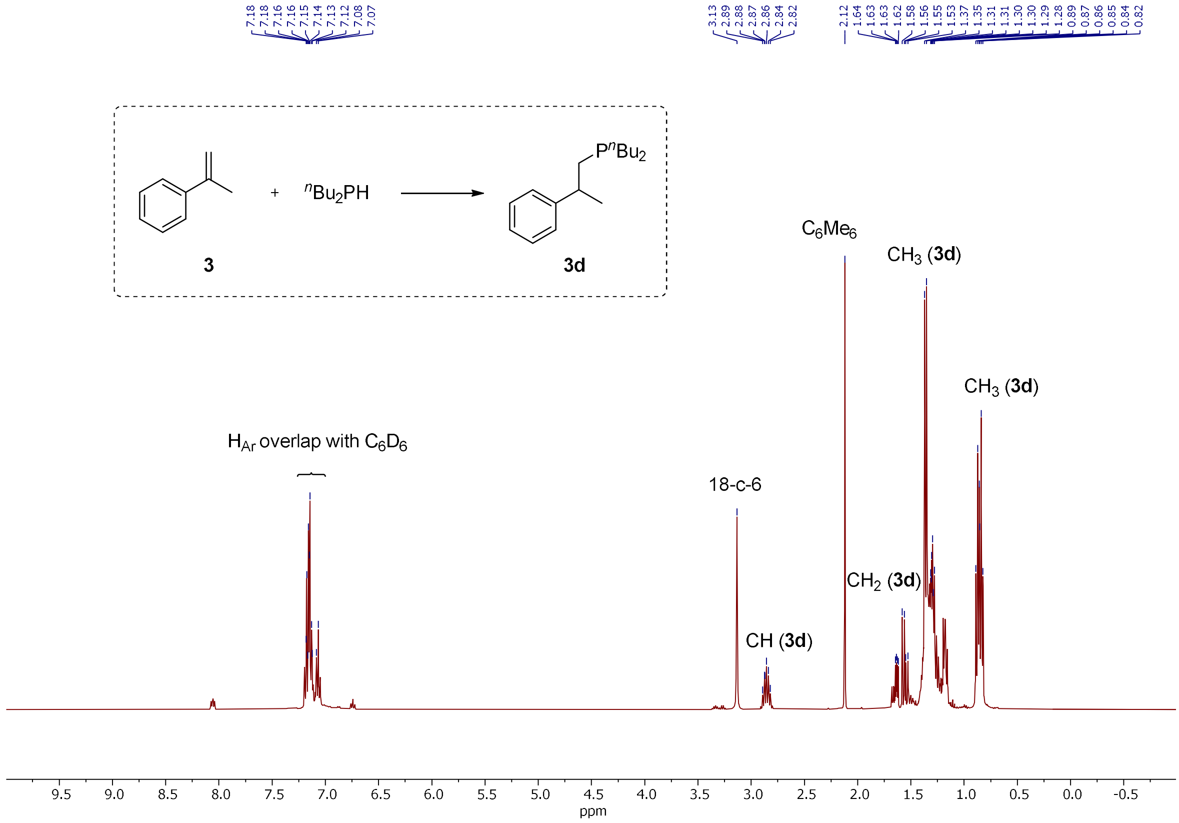

*Figure S84. ^{1}H NMR spectrum of the $\mathbf{1^{Cs}}$ (5 mol%) catalysed HP reaction of **3** with $^{n}Bu_2PH$ in benzene-d_6 at 300 K after 4 h at 90 °C. C_6Me_6 as internal standard. 88 % conversion.*

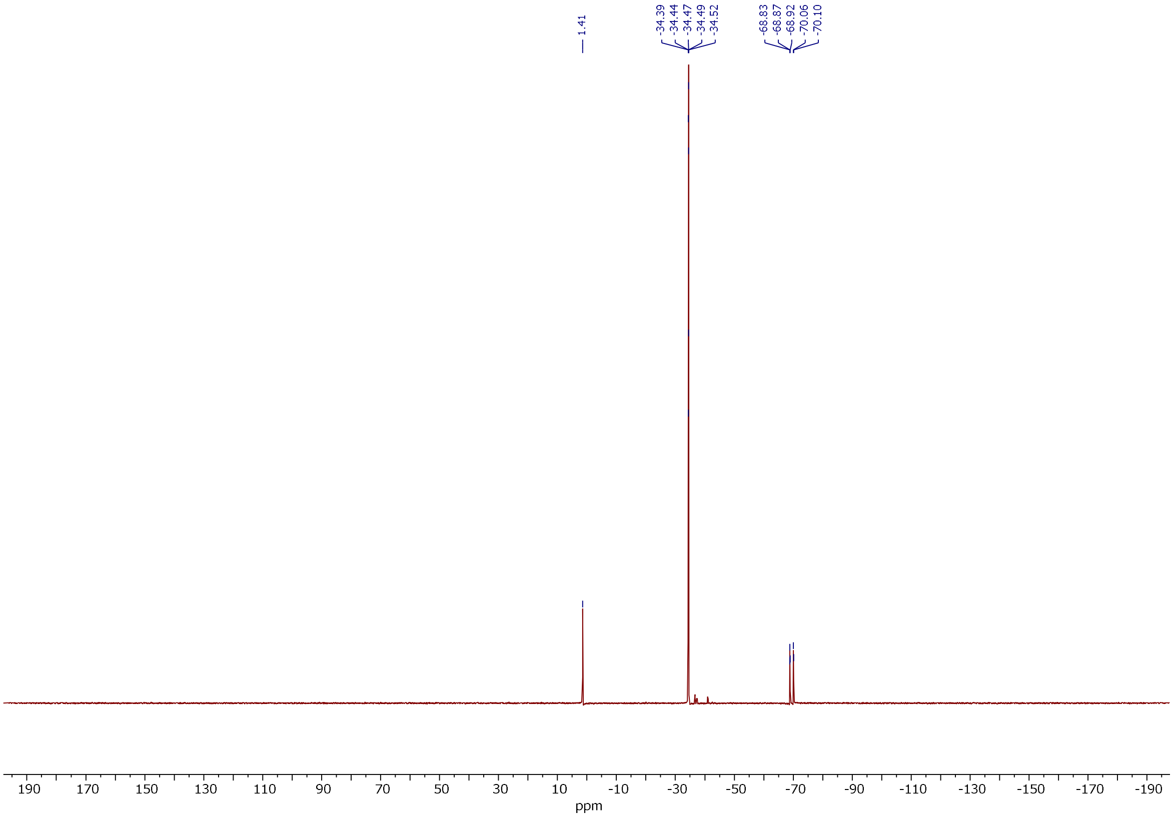

*Figure S85. ^{31}P NMR spectrum of the $\mathbf{1^{Cs}}$ (5 mol%) catalysed HP reaction of **3** with $^{n}Bu_2PH$ in benzene-d_6 at 300 K after 4 h at 90 °C. 88 % conversion. mixture of **3d** (-34.5 ppm), $^{n}Bu_2PH$ (-70 ppm) and $\mathbf{1^{Cs}}$ (1.4 ppm).*

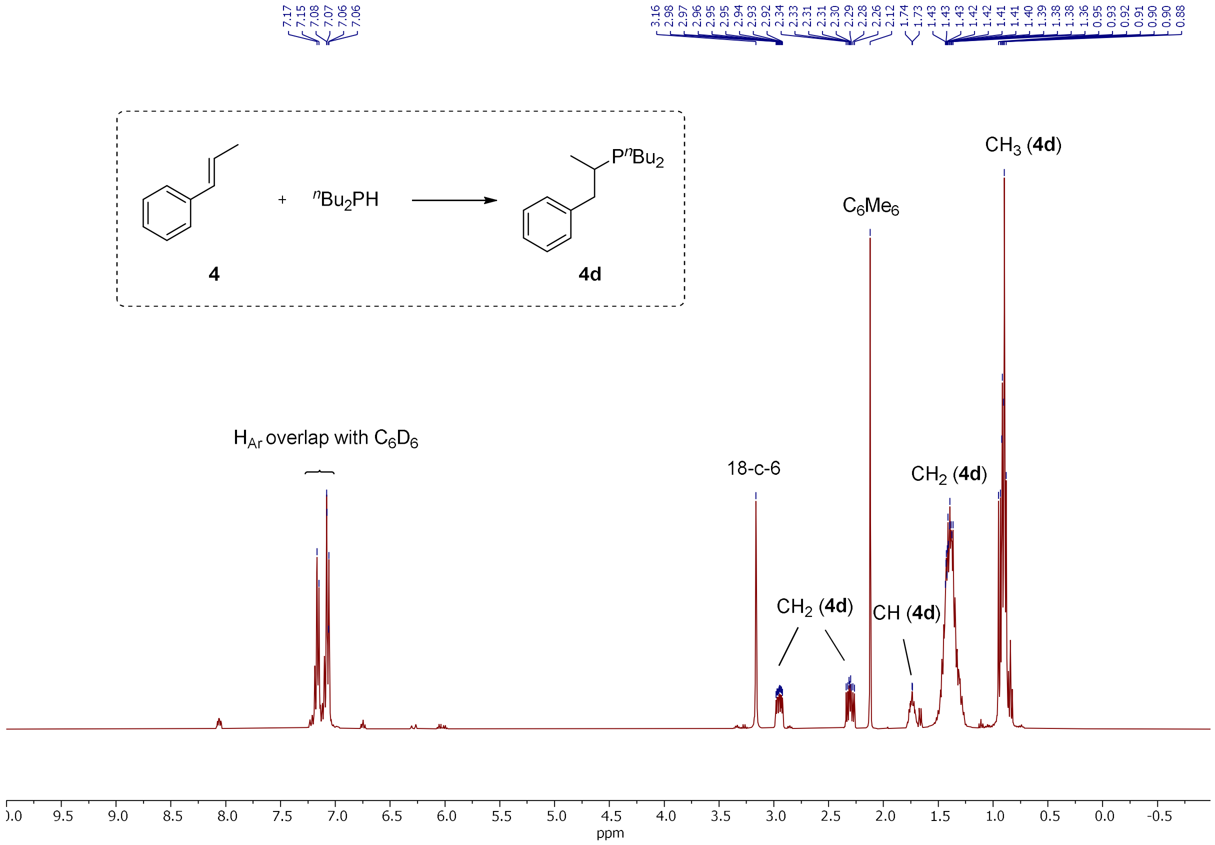

*Figure S86. ^{1}H NMR spectrum of the $\mathbf{1^{Cs}}$ (5 mol%) catalysed HP reaction of **4** with $^{n}Bu_2PH$ in benzene-d_6 at 300 K after 4 h at 90 °C. C_6Me_6 as internal standard. 92 % conversion.*

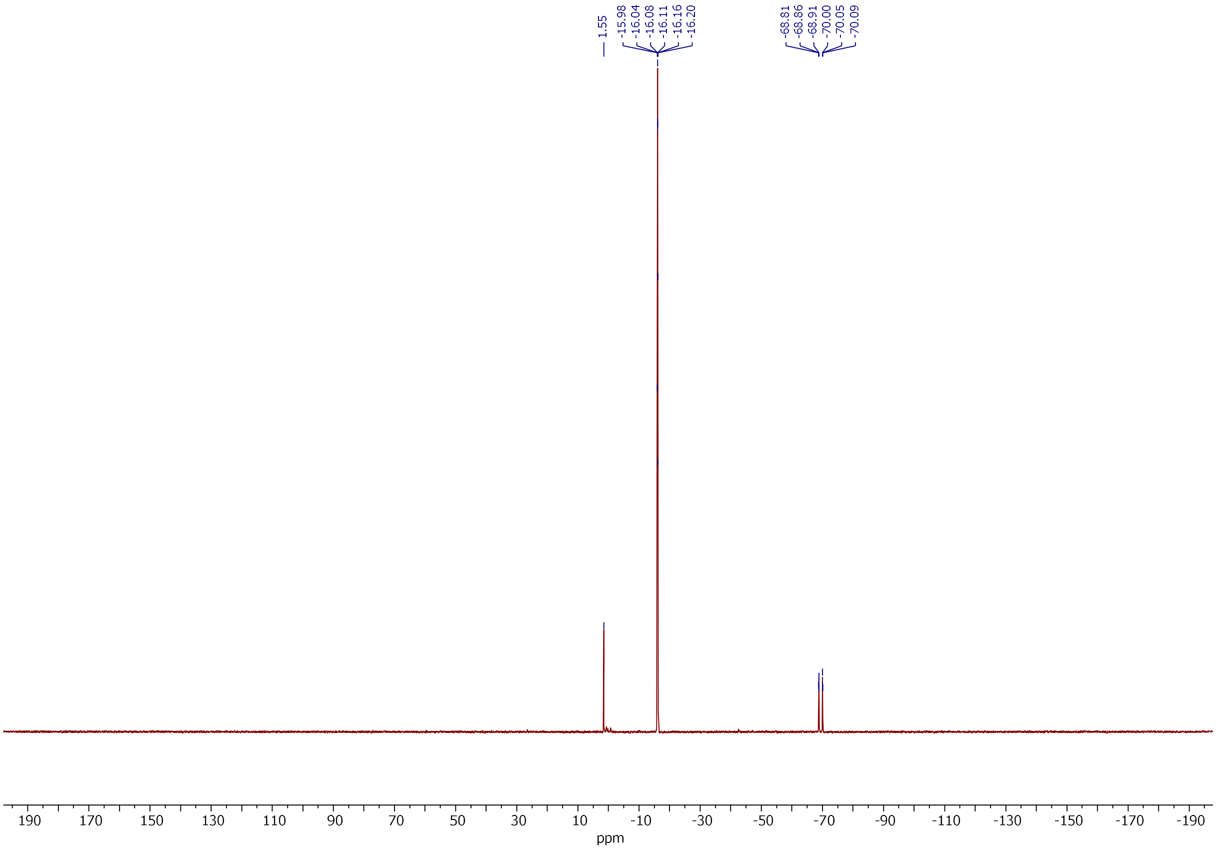

*Figure S87. ^{31}P NMR spectrum of the $\mathbf{1^{Cs}}$ (5 mol%) catalysed HP reaction of **4** with $^{n}Bu_2PH$ in benzene-d_6 at 300 K after 4 h at 90 °C. 92 % conversion. mixture of **4d** (-16 ppm), $^{n}Bu_2PH$ (-70 ppm) and $\mathbf{1^{Cs}}$ (1.5 ppm).*

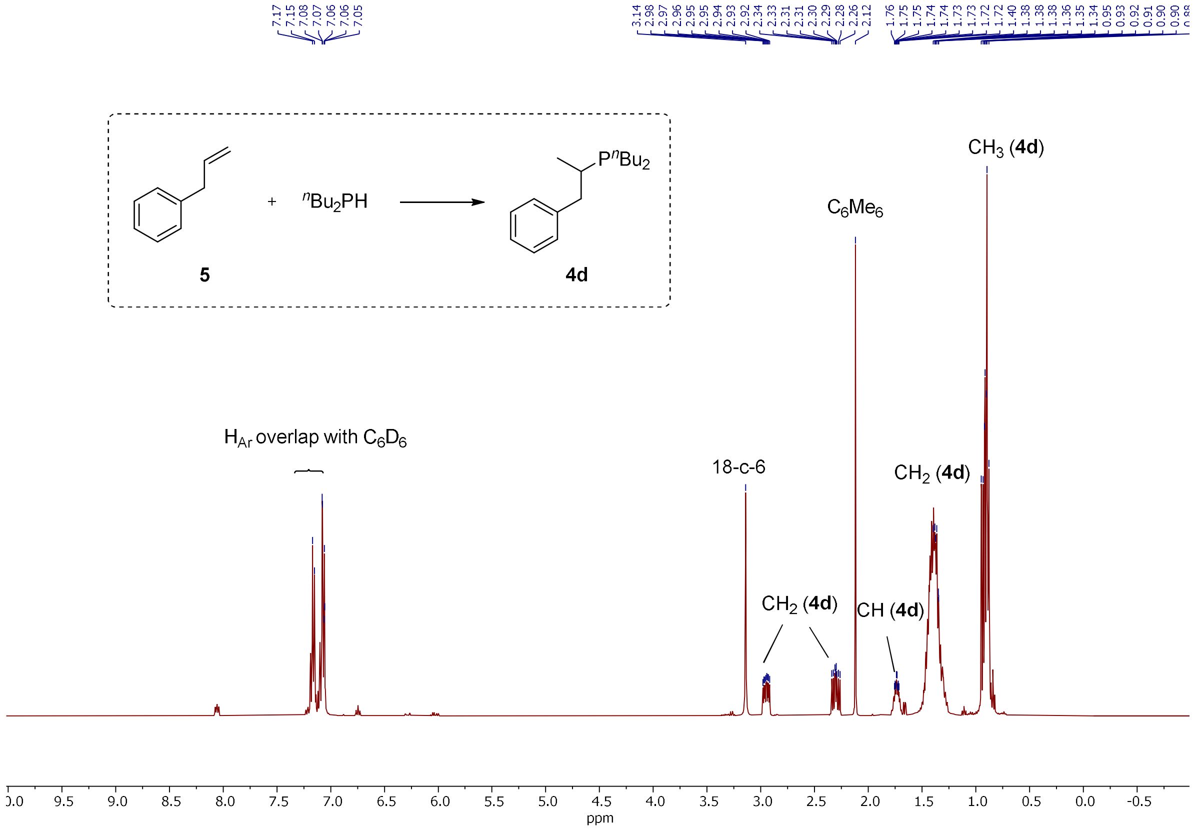

Figure S88. ^{1}H NMR spectrum of the $\mathbf{1^{Cs}}$ (5 mol%) catalysed HP reaction of **5** with $^{n}Bu_2PH$ in benzene-d_6 at 300 K after 4 h at 90 °C. C_6Me_6 as internal standard, 96 % conversion to **4d** after initial isomerization of **5** to **4** and subsequent HP.

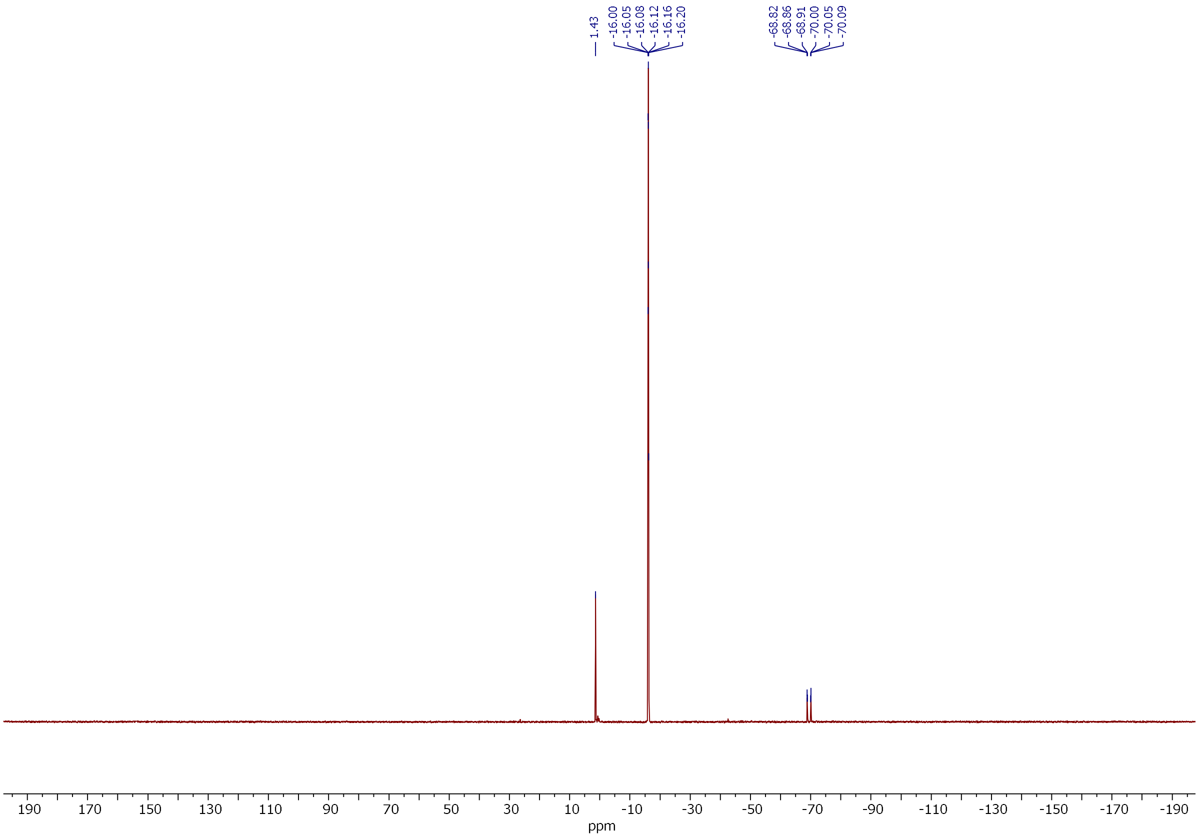

Figure S89. ^{31}P NMR spectrum of the $\mathbf{1^{Cs}}$ (5 mol%) catalysed HP reaction of **5** with $^{n}Bu_2PH$ in benzene-d_6 at 300 K after 4 h at 90 °C. 96 % conversion to **4d** after initial isomerization of **5** to **4** and subsequent HP. mixture of **4d** (-16 ppm), $^{n}Bu_2PH$ (-70 ppm) and $\mathbf{1^{Cs}}$ (1.4 ppm).

Section S3.3 – NMR Spectra of the catalytic hydrophosphination reactions of alkynes 8-12

Ph_2PH

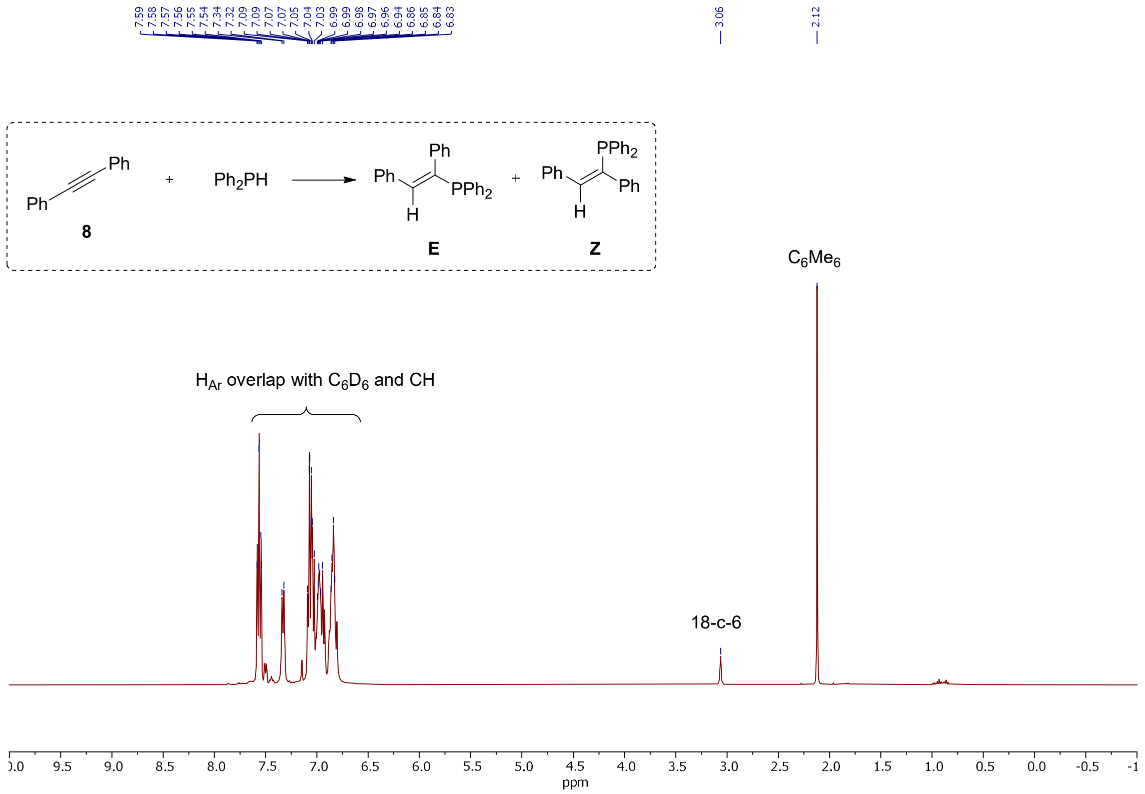

Figure S90. ¹H NMR spectrum of the $\mathbf{1^{Cs}}$ *(1 mol%) catalysed HP reaction of* **8** *with* Ph_2PH *in benzene-*d_6 *at 300 K after 2 h at RT.* C_6Me_6 *as internal standard, 99 % conversion.*

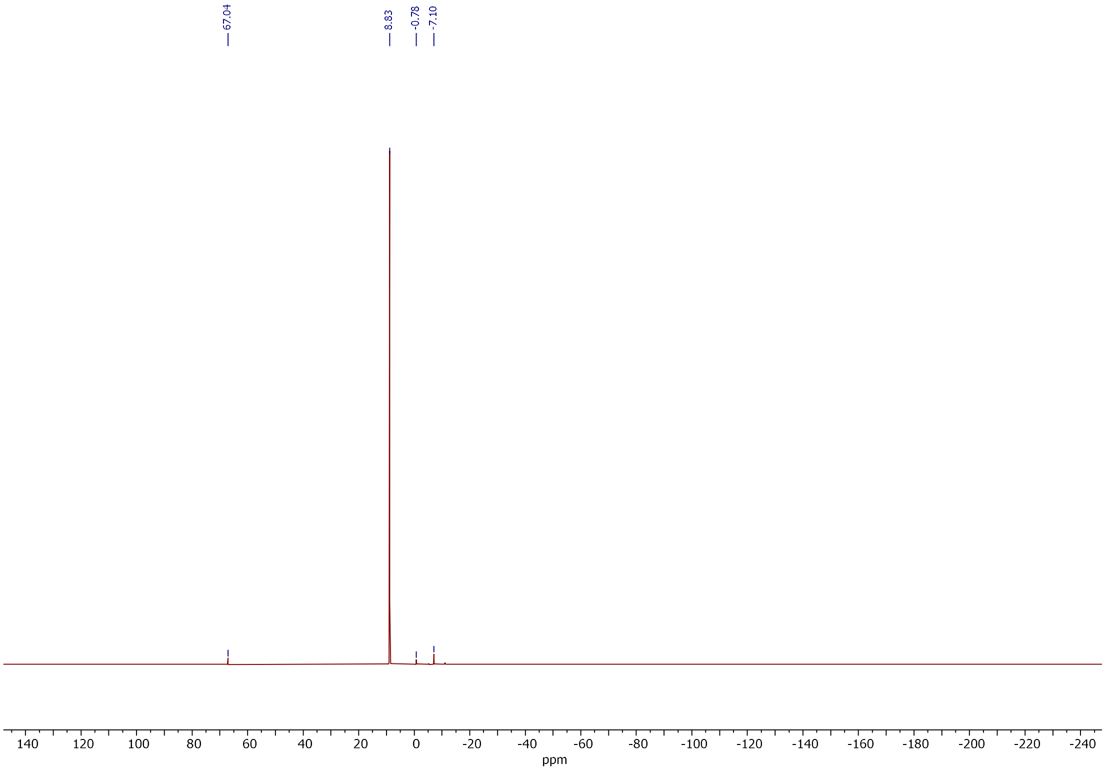

Figure S91. $^{31}P\{^1H\}$ *NMR spectrum of the* $\mathbf{1^{Cs}}$ *(1 mol%) catalysed HP reaction of* **8** *with* Ph_2PH *in benzene-*d_6 *at 300 K after 2 h at RT. 99 % conversion E-isomer (8.8 ppm) and minor unknown impurities (67, 0.8 and -7.1 ppm).*

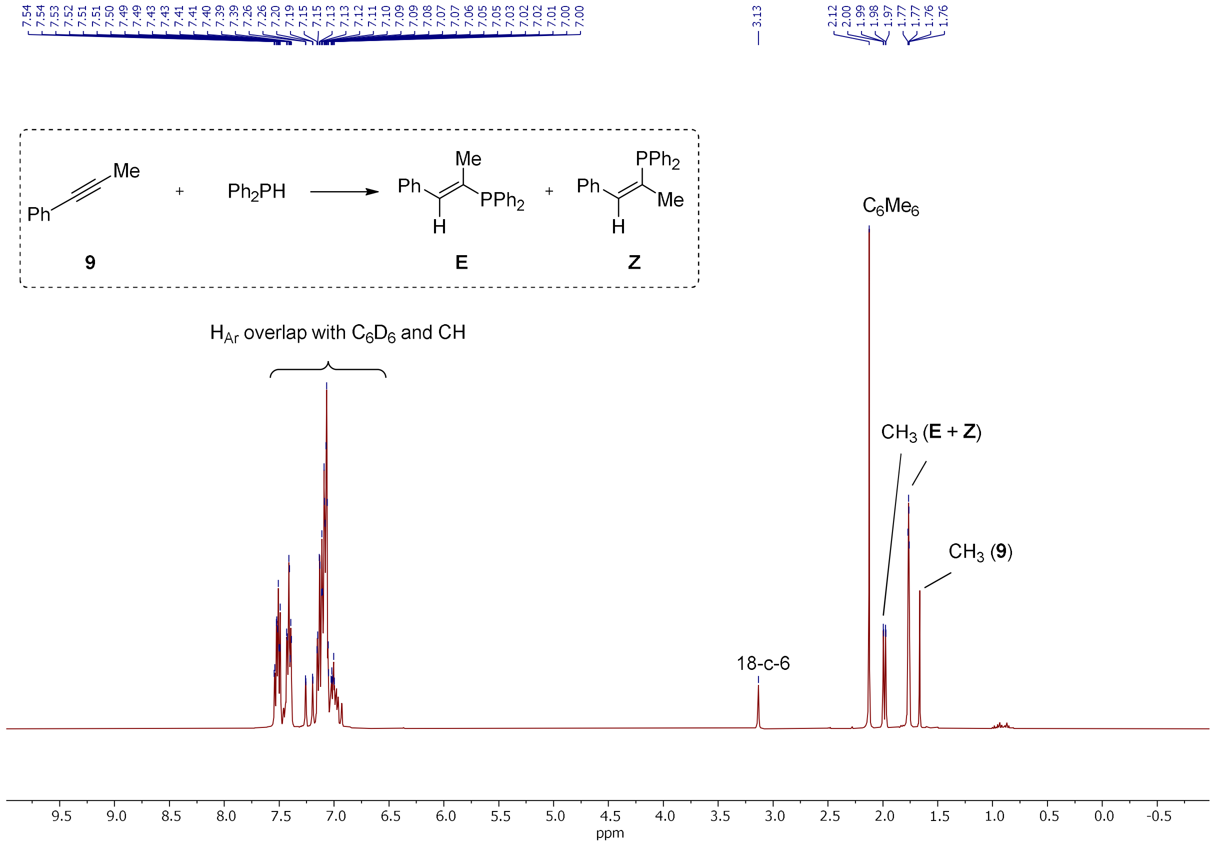

*Figure S92. ¹H NMR spectrum of the **1^Cs** (1 mol%) catalysed HP reaction of **9** with Ph_2PH in benzene-d_6 at 300 K after 4 h at RT. C_6Me_6 as internal standard, 99 % conversion.*

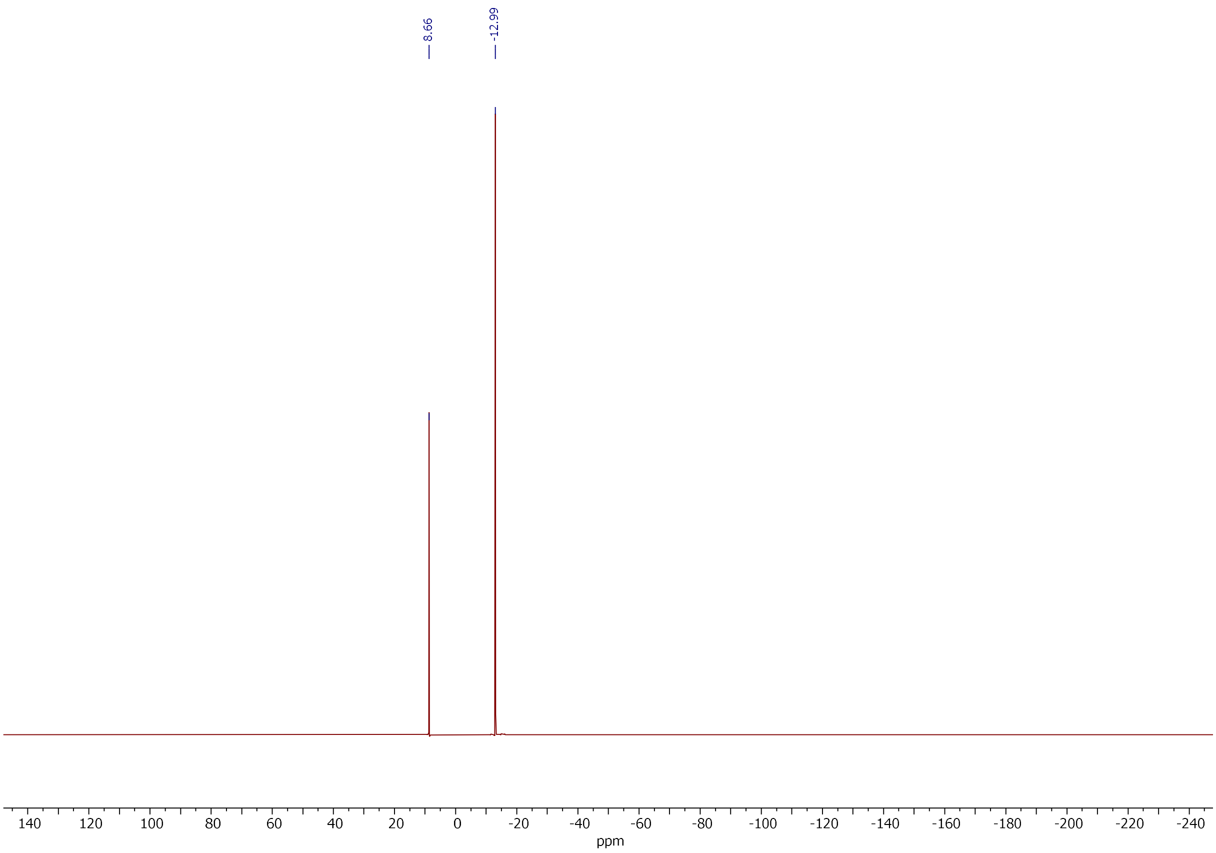

*Figure S93. ³¹P{¹H} NMR spectrum of the **1^Cs** (1 mol%) catalysed HP reaction of **9** with Ph_2PH in benzene-d_6 at 300 K after 4 h at RT. 99 % conversion E-isomer (-13 ppm), Z-isomer (8.7 ppm).*

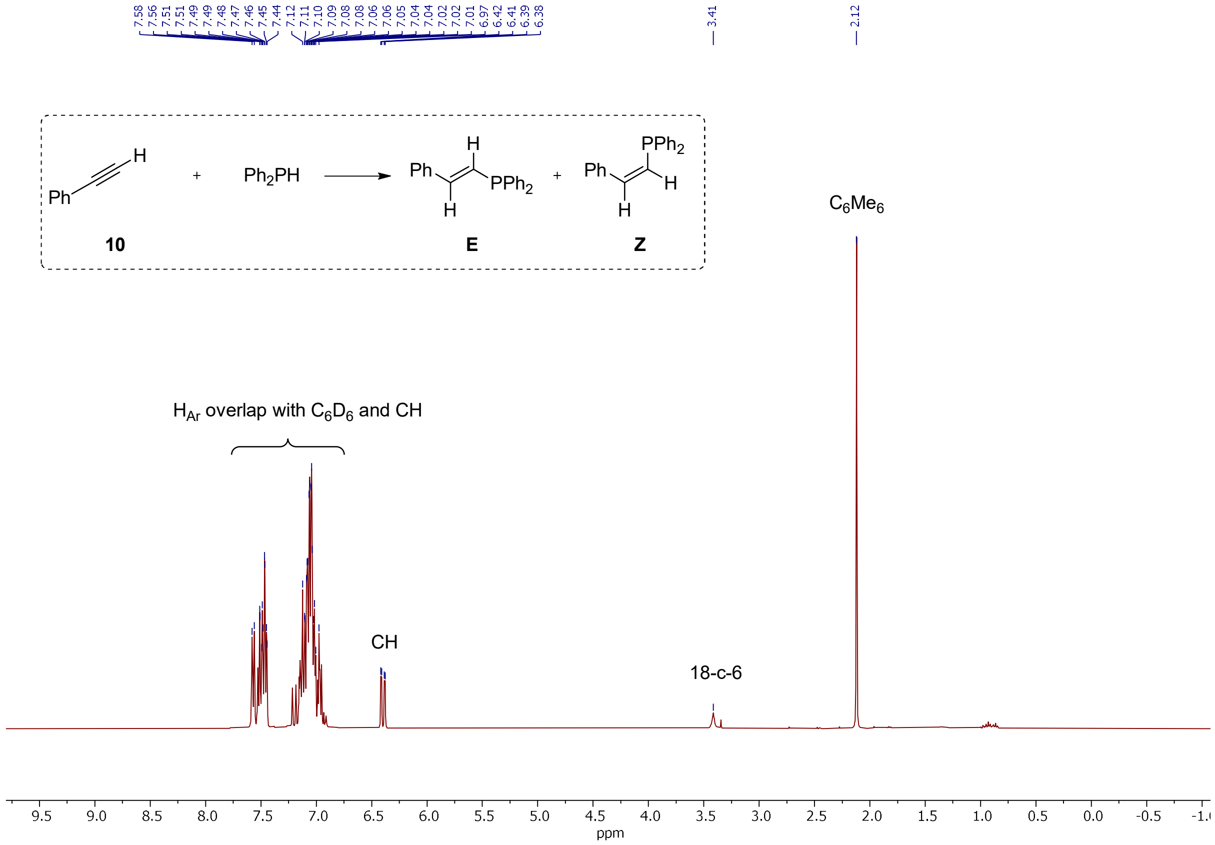

*Figure S94. ^{1}H NMR spectrum of the **1^{Cs}** (1 mol%) catalysed HP reaction of **10** with Ph_2PH in benzene-d_6 at 300 K after 45 min at RT. C_6Me_6 as internal standard, 99 % conversion.*

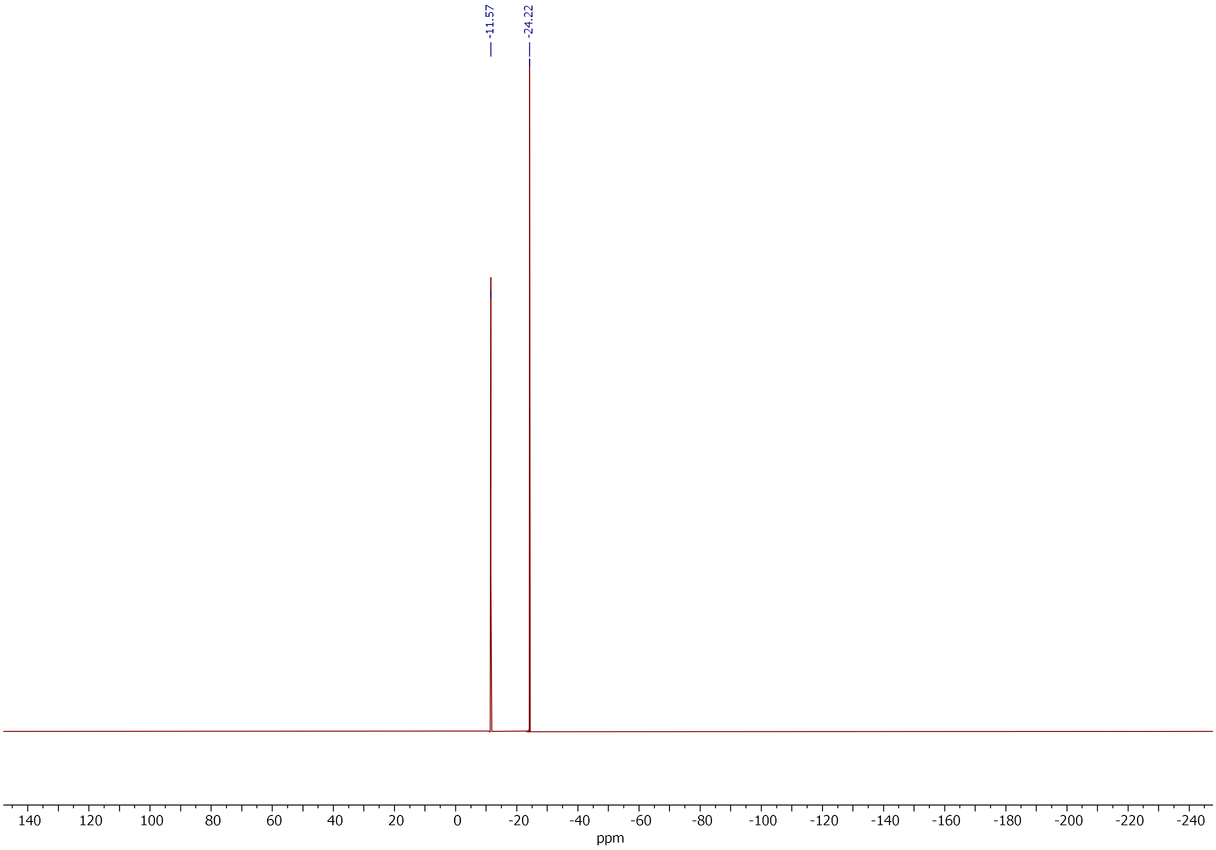

*Figure S95. $^{31}P\{^{1}H\}$ NMR spectrum of the **1^{Cs}** (1 mol%) catalysed HP reaction of **10** with Ph_2PH in benzene-d_6 at 300 K after 45 min at RT. 99 % conversion E-isomer (-24.2 ppm), Z-isomer (-11.6 ppm).*

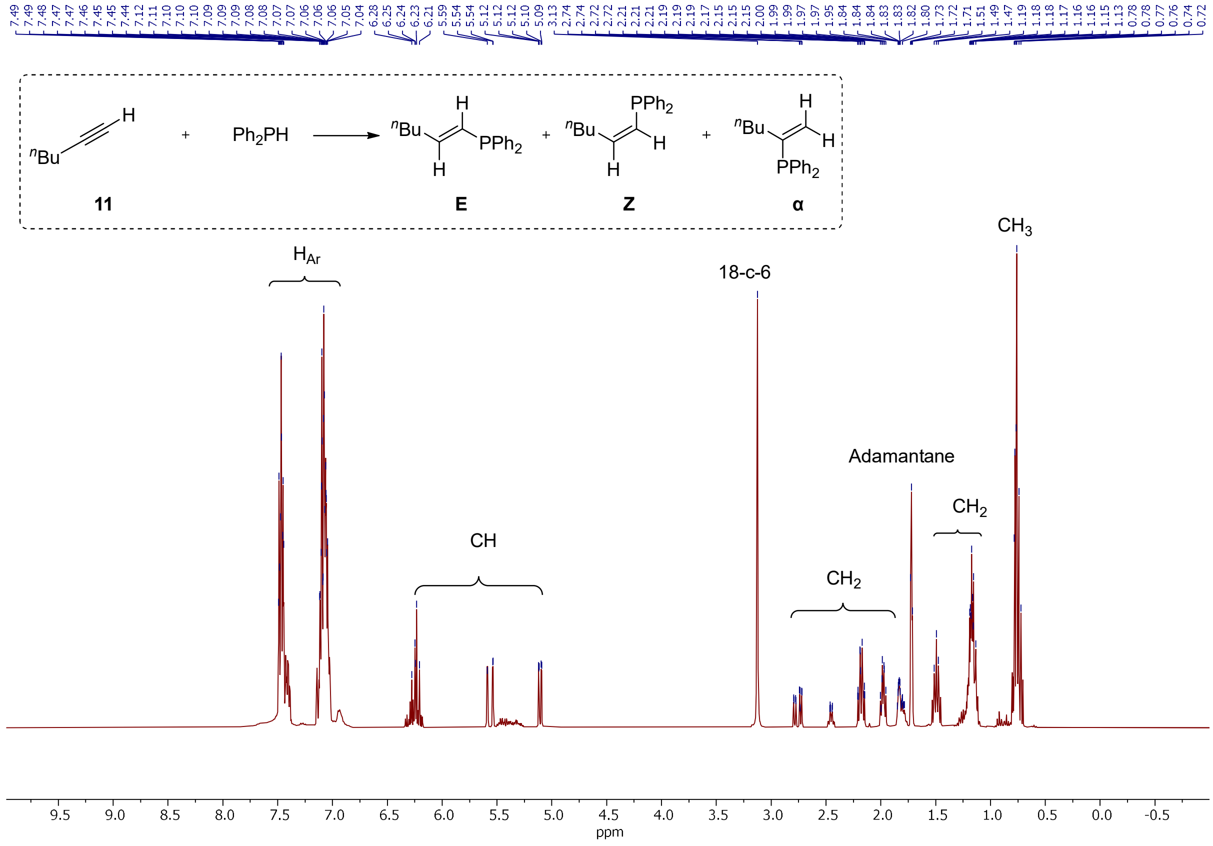

*Figure S96. ^{1}H NMR spectrum of the **1**^{Cs} (5 mol%) catalysed HP reaction of **11** with Ph_2PH in benzene-d_6 at 300 K after 4 h at 90 °C. Adamantane as internal standard, 73 % conversion.*

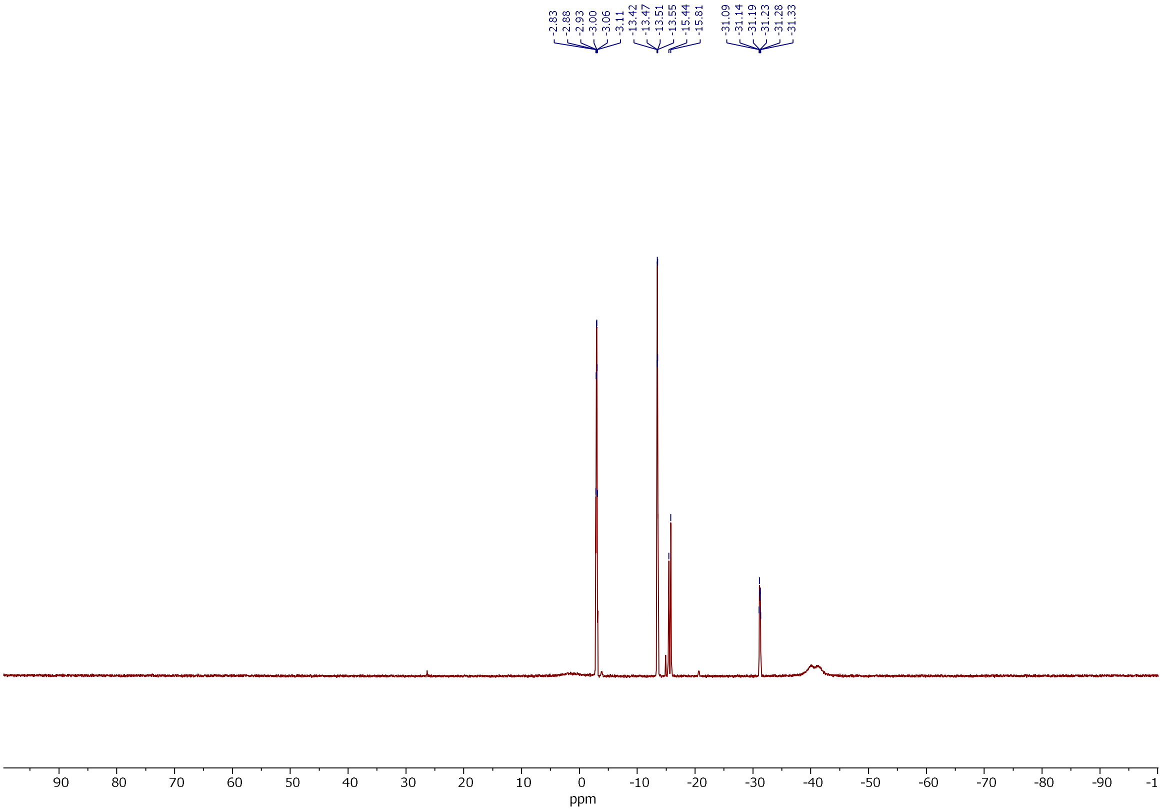

*Figure S97. ^{31}P NMR spectrum of the **1**^{Cs} (5 mol%) catalysed HP reaction of **11** with Ph_2PH in benzene-d_6 at 300 K after 4 h at 90 °C. 73 % conversion E-isomer (-31.2 ppm), Z-isomer (-13.5 ppm), α-isomer (-3 ppm), Ph_2PH (-40 ppm) and unknown side products (-15 ppm).*

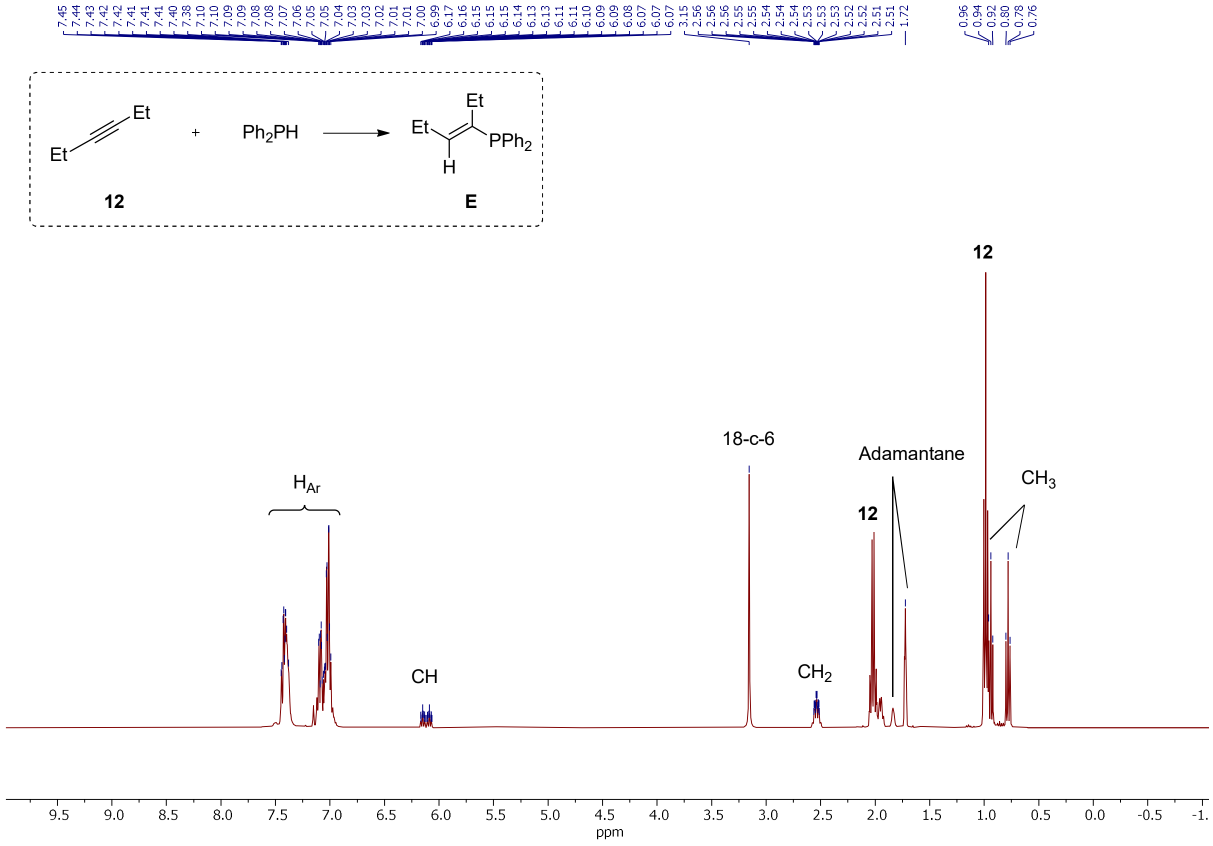

Figure S98. ^{1}H *NMR spectrum of the* $\mathbf{1^{Cs}}$ *(5 mol%) catalysed HP reaction of* ***12*** *with* Ph_2PH *in benzene-*d_6 *at 300 K after 22 h at 90 °C. Adamantane as internal standard, 25 % conversion.*

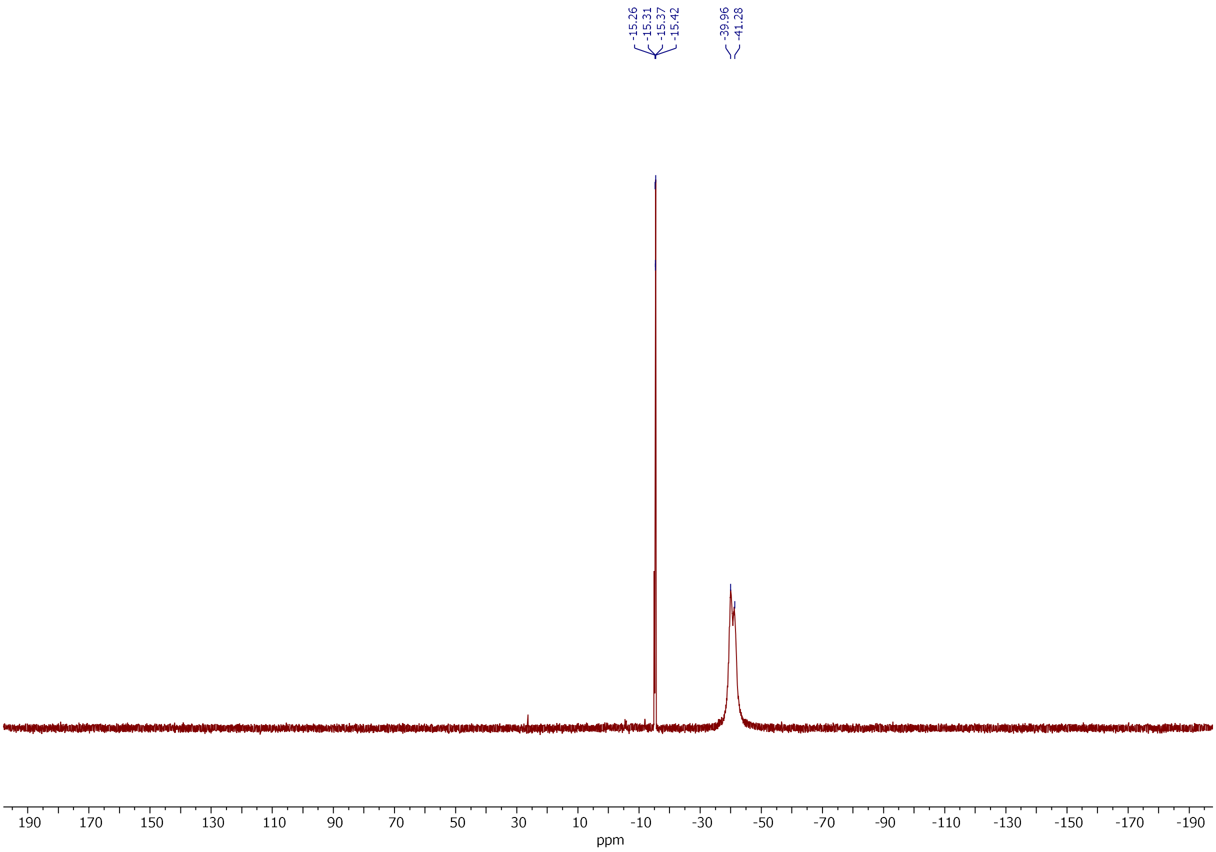

Figure S99. ^{31}P *NMR spectrum of the* $\mathbf{1^{Cs}}$ *(5 mol%) catalysed HP reaction of* ***12*** *with* Ph_2PH *in benzene-*d_6 *at 300 K after 22 h at 90 °C. 25 % conversion E-isomer (-15.4 ppm),* Ph_2PH *(-40 ppm.*

tBuPhPH

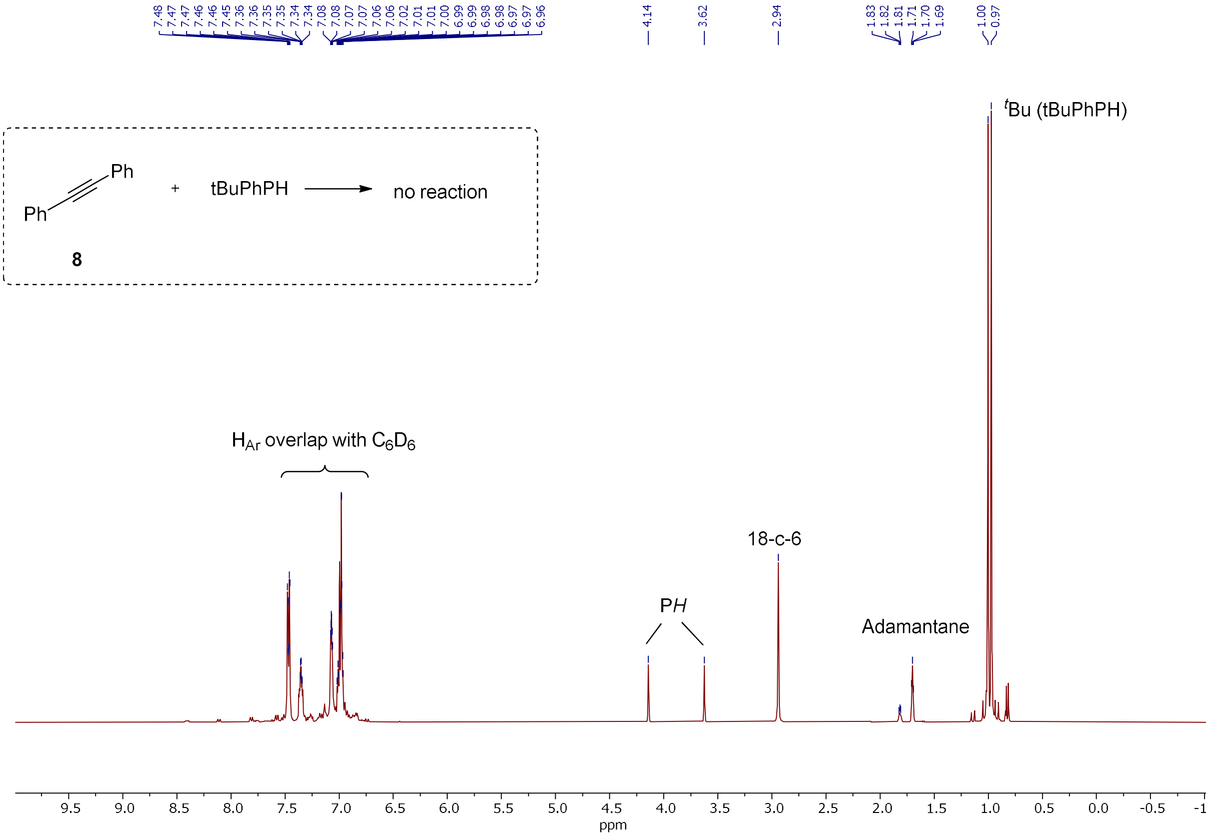

Figure S100. ^{1}*H NMR spectrum of the* $\mathbf{1^{Cs}}$ *(5 mol%) catalysed HP reaction of* ***8*** *with* ^{t}*BuPhPH in benzene-d*$_6$ *at 300 K after 18 h at 90 °C. Adamantane as internal standard, no conversion.*

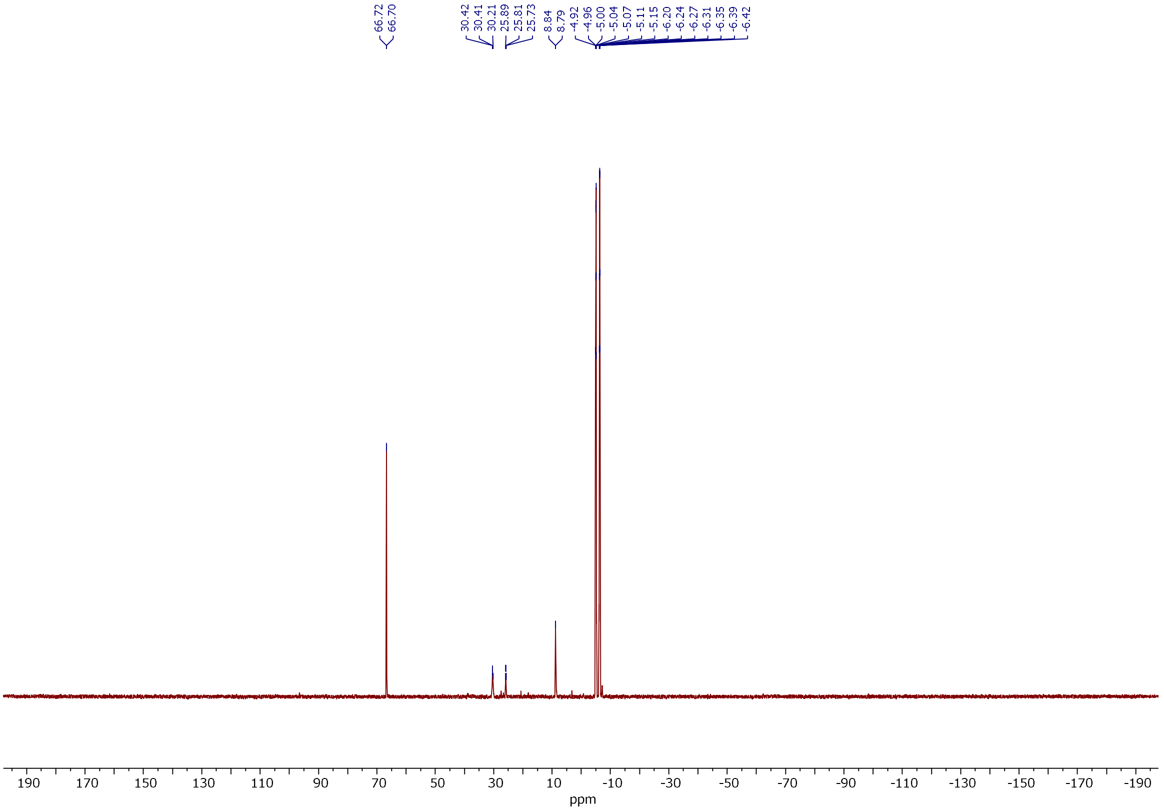

Figure S101. ^{31}*P NMR spectrum of the* $\mathbf{1^{Cs}}$ *(5 mol%) catalysed HP reaction of* ***8*** *with* ^{t}*BuPhPH in benzene-d*$_6$ *at 300 K after 18 h at 90 °C. No conversion, only minor unknown side products.*

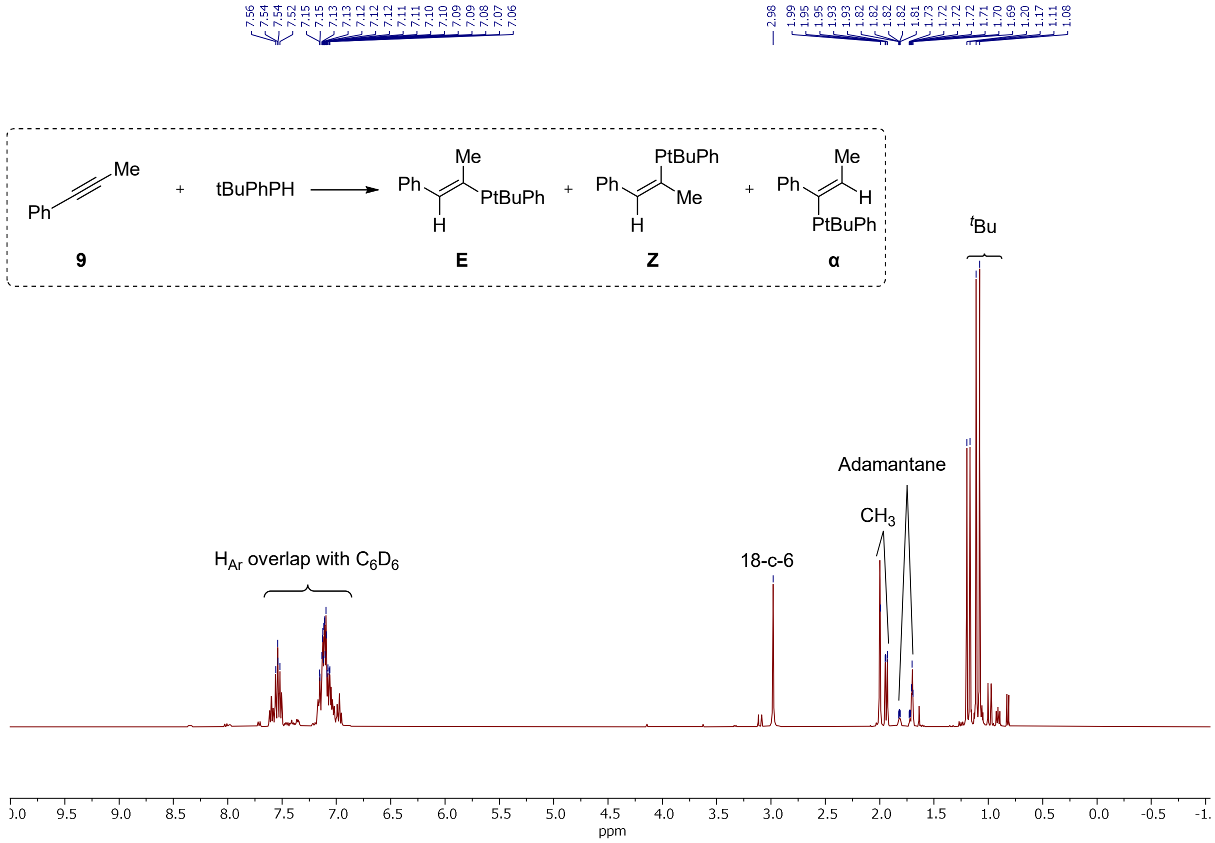

*Figure S102. ^{1}H NMR spectrum of the **1^{Cs}** (5 mol%) catalysed HP reaction of **9** with ^{t}BuPhPH in benzene-d$_6$ at 300 K after 2 h at 90 °C. Adamantane as internal standard, 93 % conversion.*

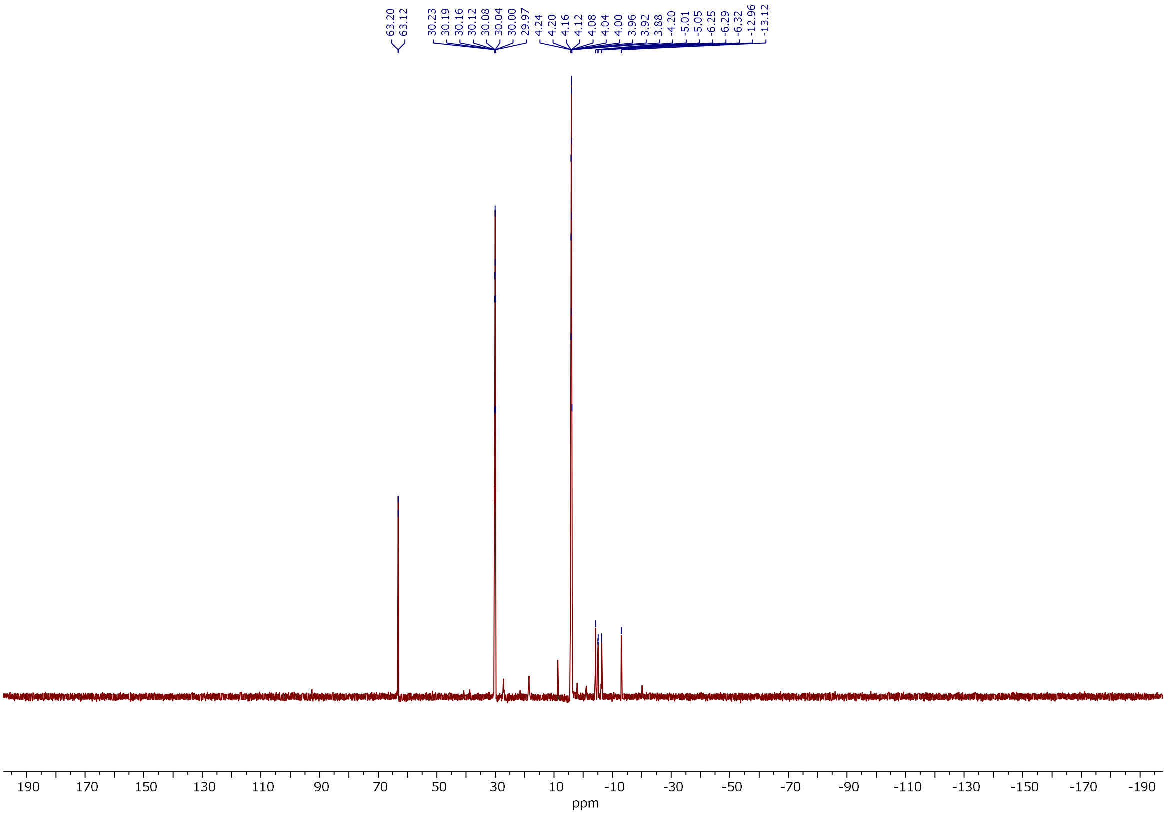

*Figure S103. ^{31}P NMR spectrum of the **1^{Cs}** (5 mol%) catalysed HP reaction of **9** with ^{t}BuPhPH in benzene-d$_6$ at 300 K after 2 h at 90 °C. 93 % conversion, mixture of isomers (63, 30 and 4 ppm), ^{t}BuPhPH (-6.3 ppm) and some very minor unknown side products.*

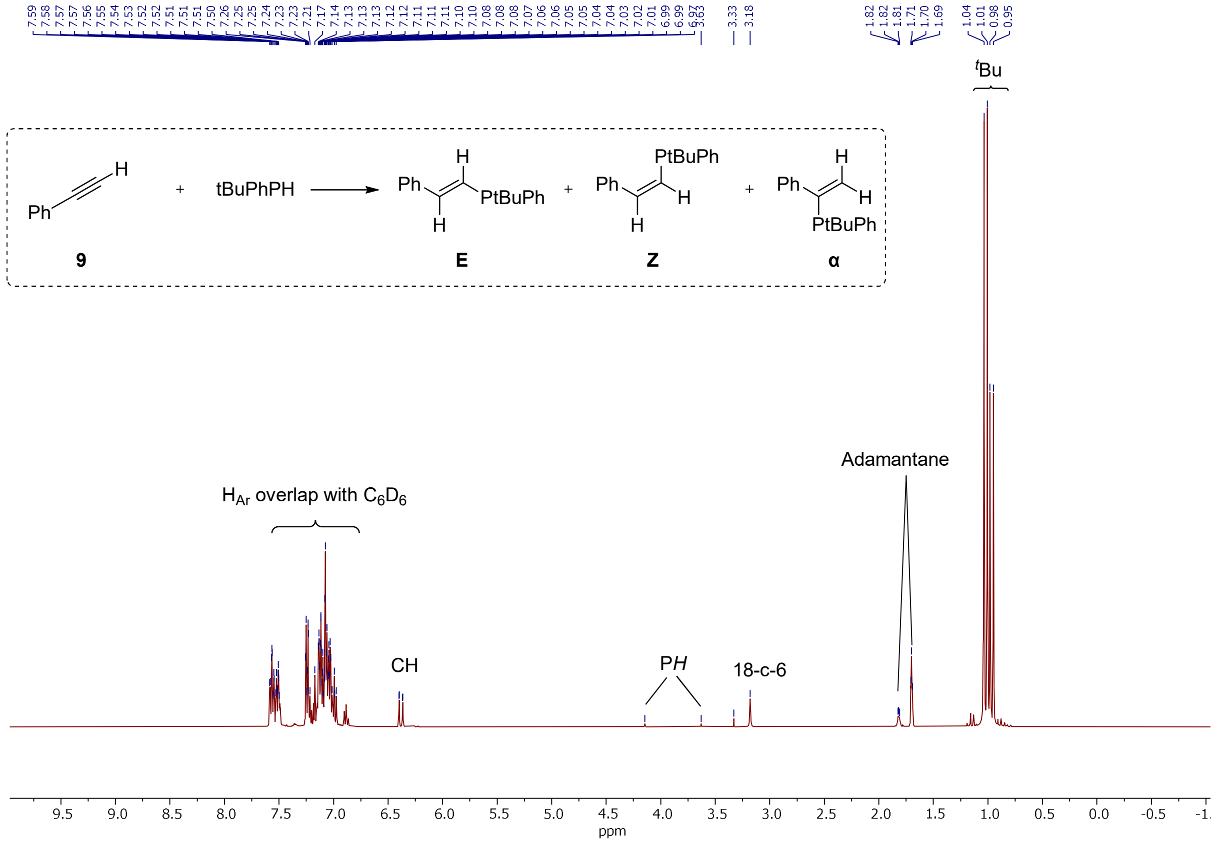

*Figure S104. ^{1}H NMR spectrum of the **1^{Cs}** (5 mol%) catalysed HP reaction of **10** with ^{t}BuPhPH in benzene-d_6 at 300 K after 2 h at 90 °C. Adamantane as internal standard, 96 % conversion.*

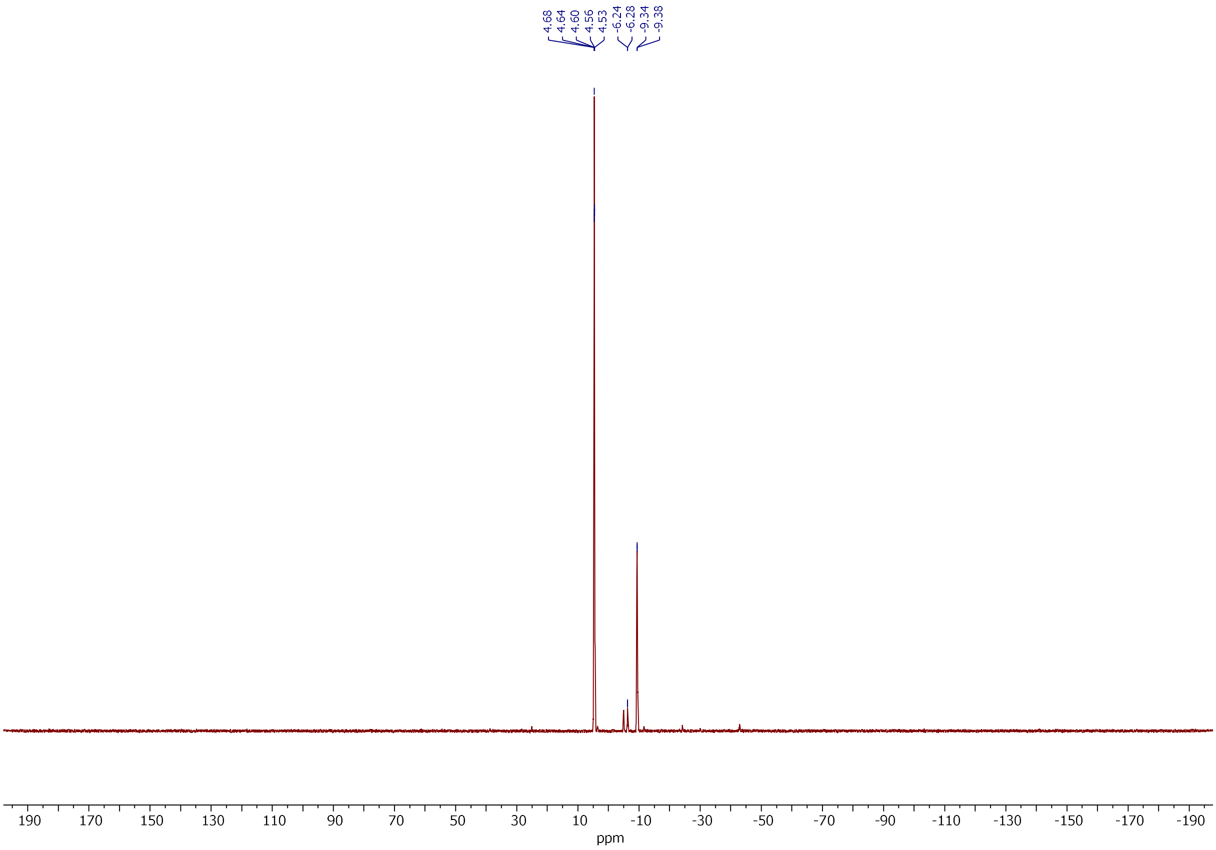

*Figure S105. ^{31}P NMR spectrum of the **1^{Cs}** (5 mol%) catalysed HP reaction of **10** with ^{t}BuPhPH in benzene-d_6 at 300 K after 2 h at 90 °C. 96 % conversion, mixture of isomers (4.6 and -9.4 ppm) and ^{t}BuPhPH (-6.3 ppm).*

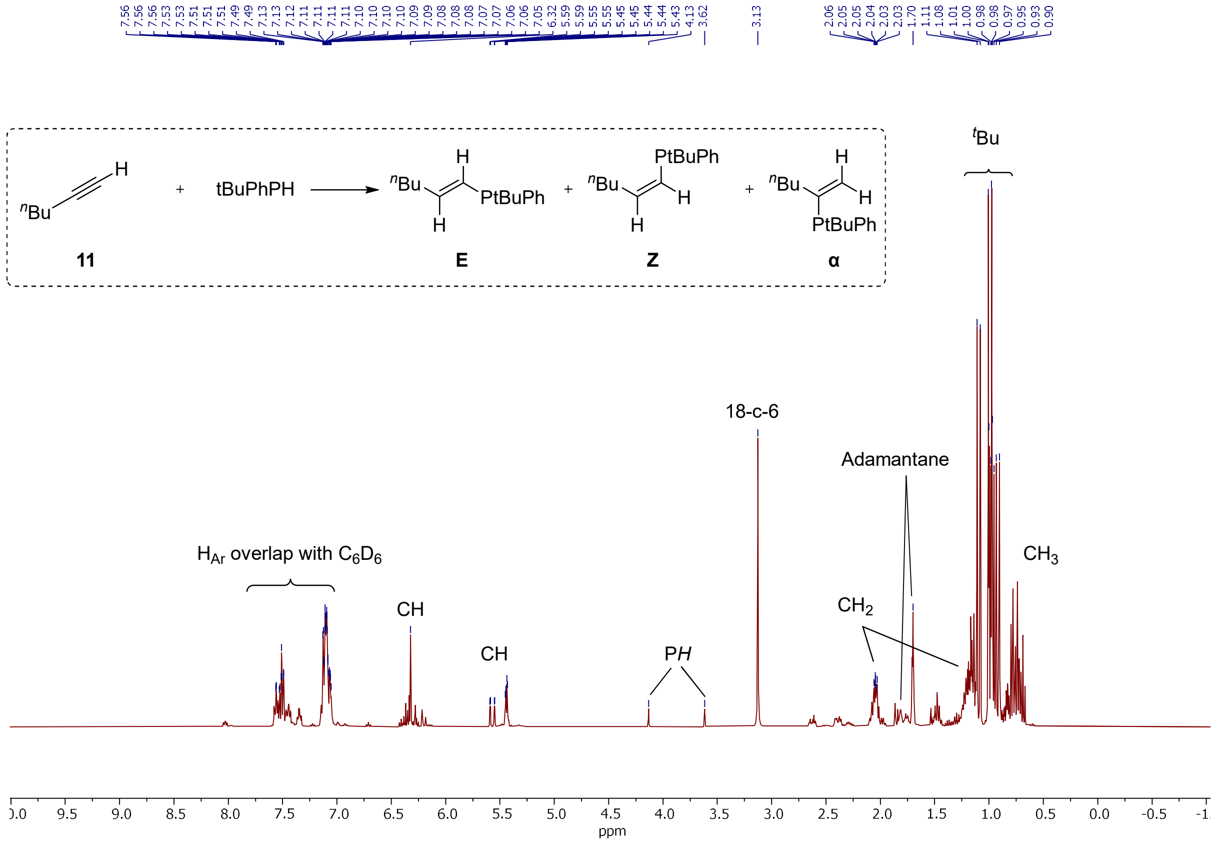

*Figure S106. ^{1}H NMR spectrum of the **1^{Cs}** (5 mol%) catalysed HP reaction of **11** with ^{t}BuPhPH in benzene-d$_6$ at 300 K after 18 h at 90 °C. Adamantane as internal standard, 87 % conversion.*

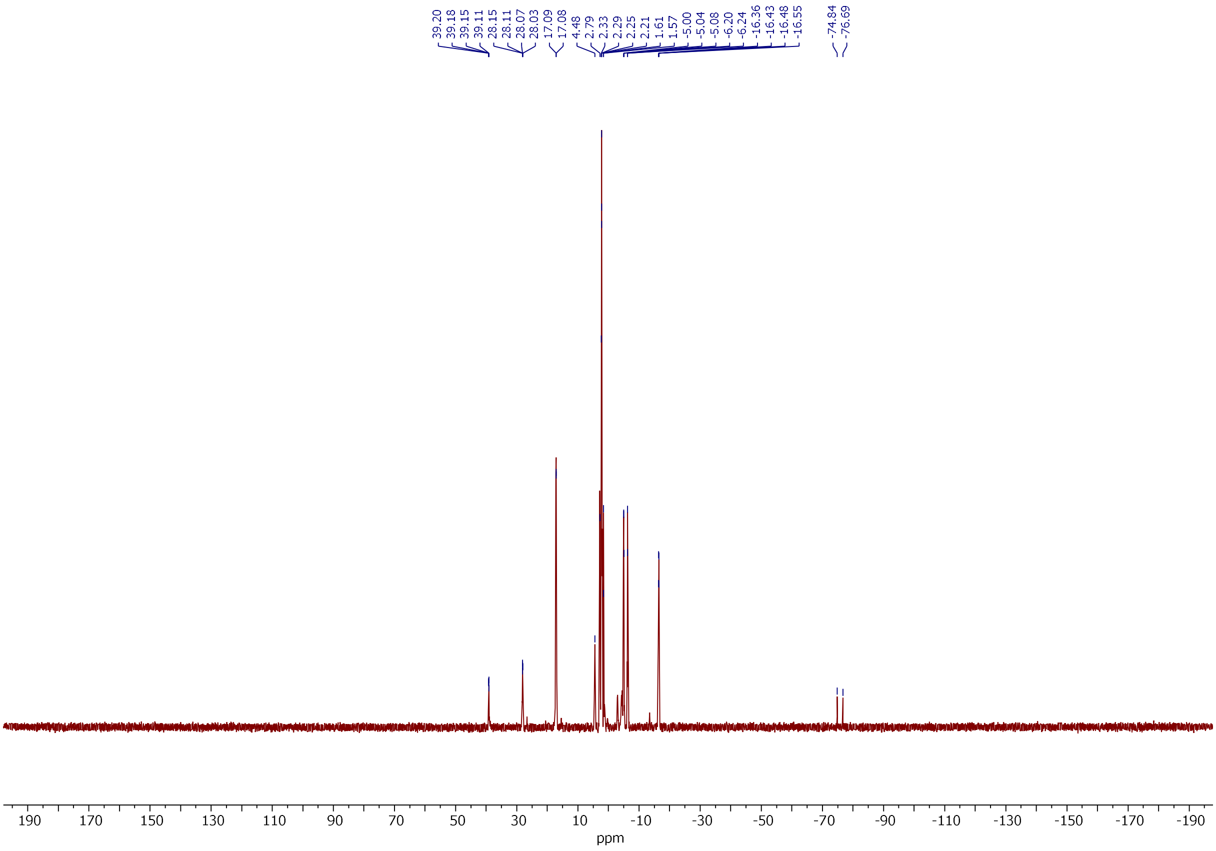

*Figure S107. ^{31}P NMR spectrum of the **1^{Cs}** (5 mol%) catalysed HP reaction of **11** with ^{t}BuPhPH in benzene-d$_6$ at 300 K after 18 h at 90 °C. 87 % conversion, mixture of isomers, possible side products and ^{t}BuPhPH (-6.3 ppm).*

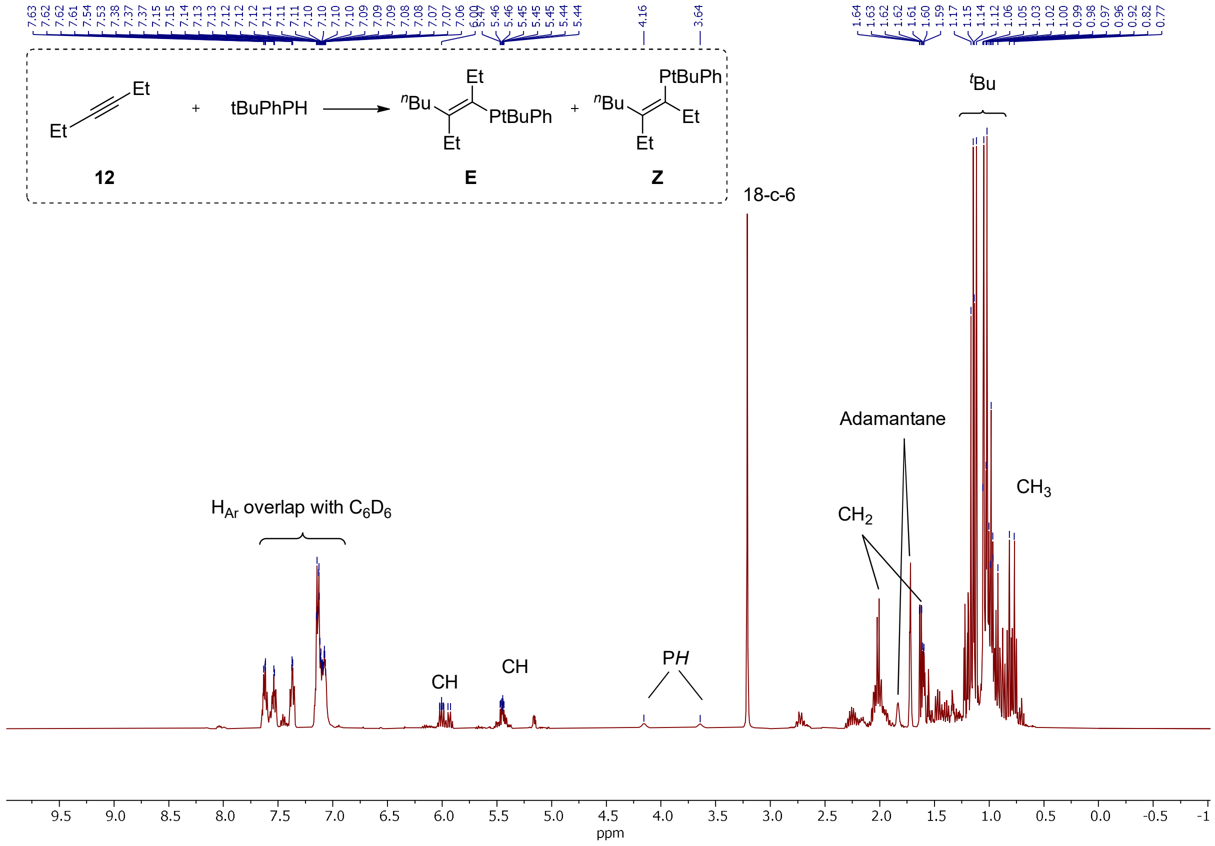

*Figure S108. ^{1}H NMR spectrum of the **1^{Cs}** (5 mol%) catalysed HP reaction of **12** with ^{t}BuPhPH in benzene-d$_6$ at 300 K after 18 h at 90 °C. Adamantane as internal standard, 90 % conversion.*

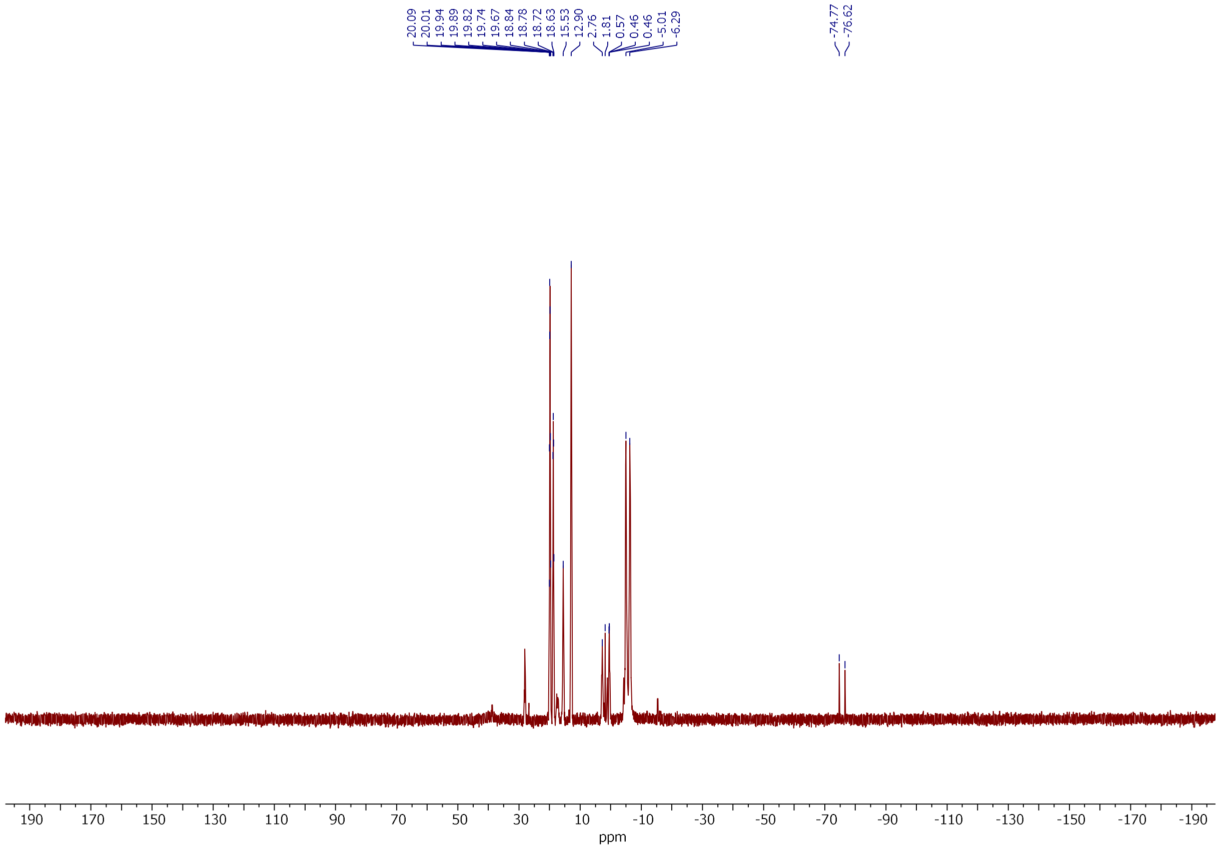

*Figure S109. ^{31}P NMR spectrum of the **1^{Cs}** (5 mol%) catalysed HP reaction of **12** with ^{t}BuPhPH in benzene-d$_6$ at 300 K after 18 h at 90 °C. 80 % conversion, mixture of isomers, possible side products and ^{t}BuPhPH (-6.3 ppm).*

tBu$_2$PH

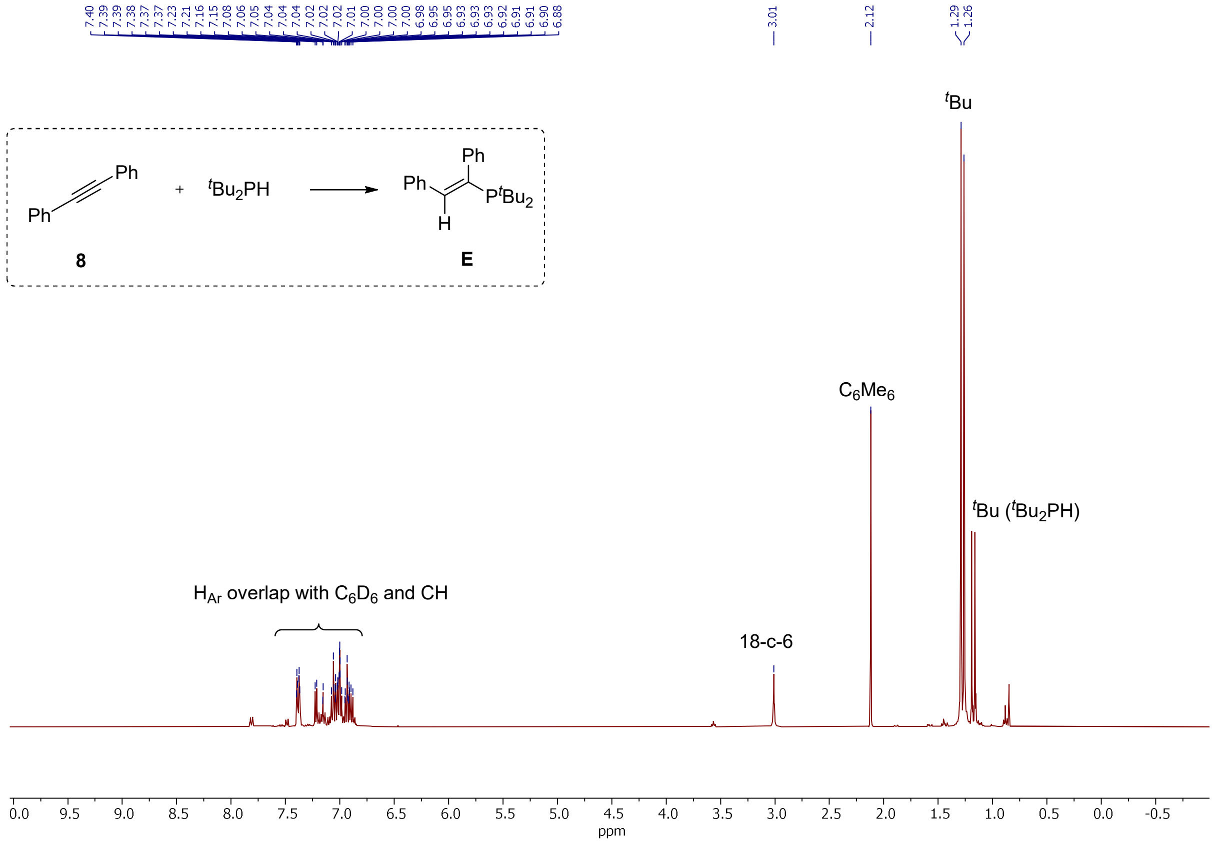

*Figure S110. ^{1}H NMR spectrum of the **1^{Cs}** (5 mol%) catalysed HP reaction of **8** with ^{t}Bu$_2$PH in benzene-d$_6$ at 300 K after 4 h at 90 °C. C$_6$Me$_6$ as internal standard, 99 % conversion.*

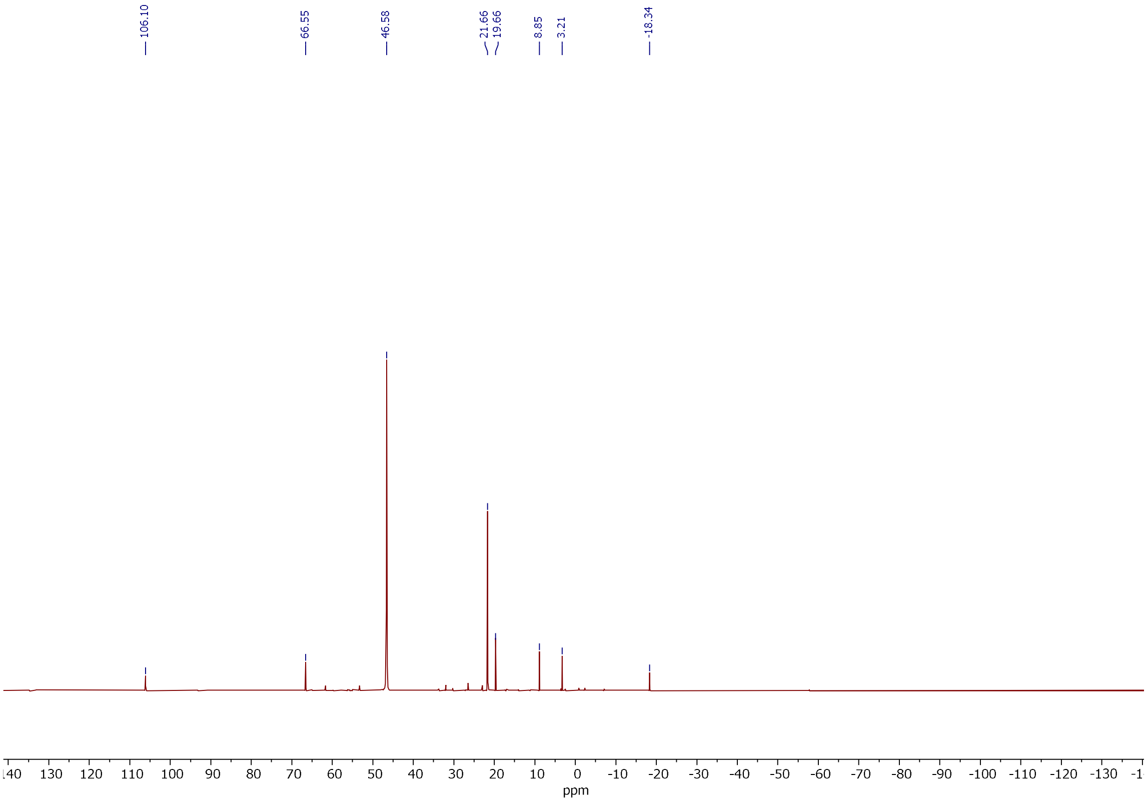

*Figure S111. ^{31}P{^{1}H} NMR spectrum of the **1^{Cs}** (5 mol%) catalysed HP reaction of **8** with ^{t}Bu$_2$PH in benzene-d$_6$ at 300 K after 18 h at 90 °C. 99 % conversion, product (46.6 ppm), ^{t}Bu$_2$PH (21.6 ppm) and minor unknown side products.*

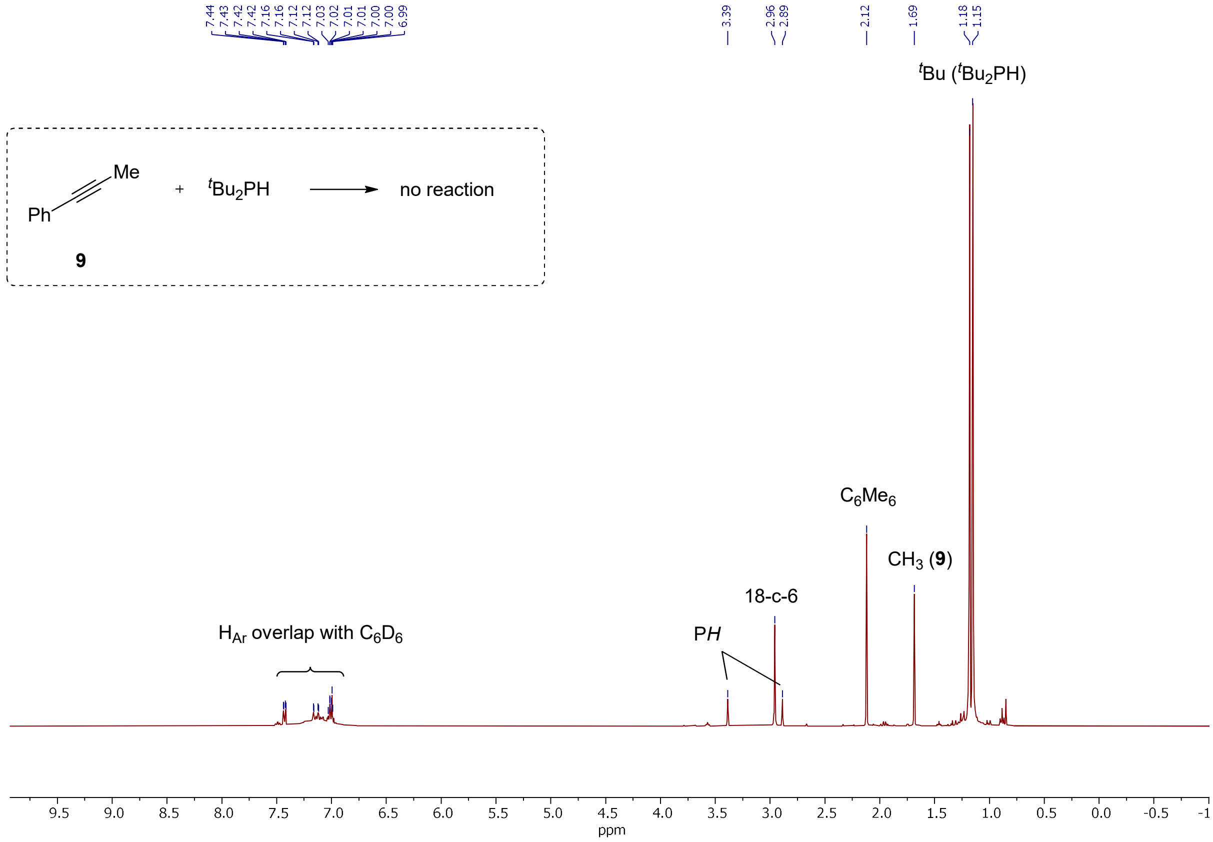

*Figure S112. ^{1}H NMR spectrum of the **1**^{Cs} (5 mol%) catalysed HP reaction of **9** with ^{t}Bu$_2$PH in benzene-d$_6$ at 300 K after 19 h at 90 °C. C_6Me_6 as internal standard, no conversion, mixture of substrates.*

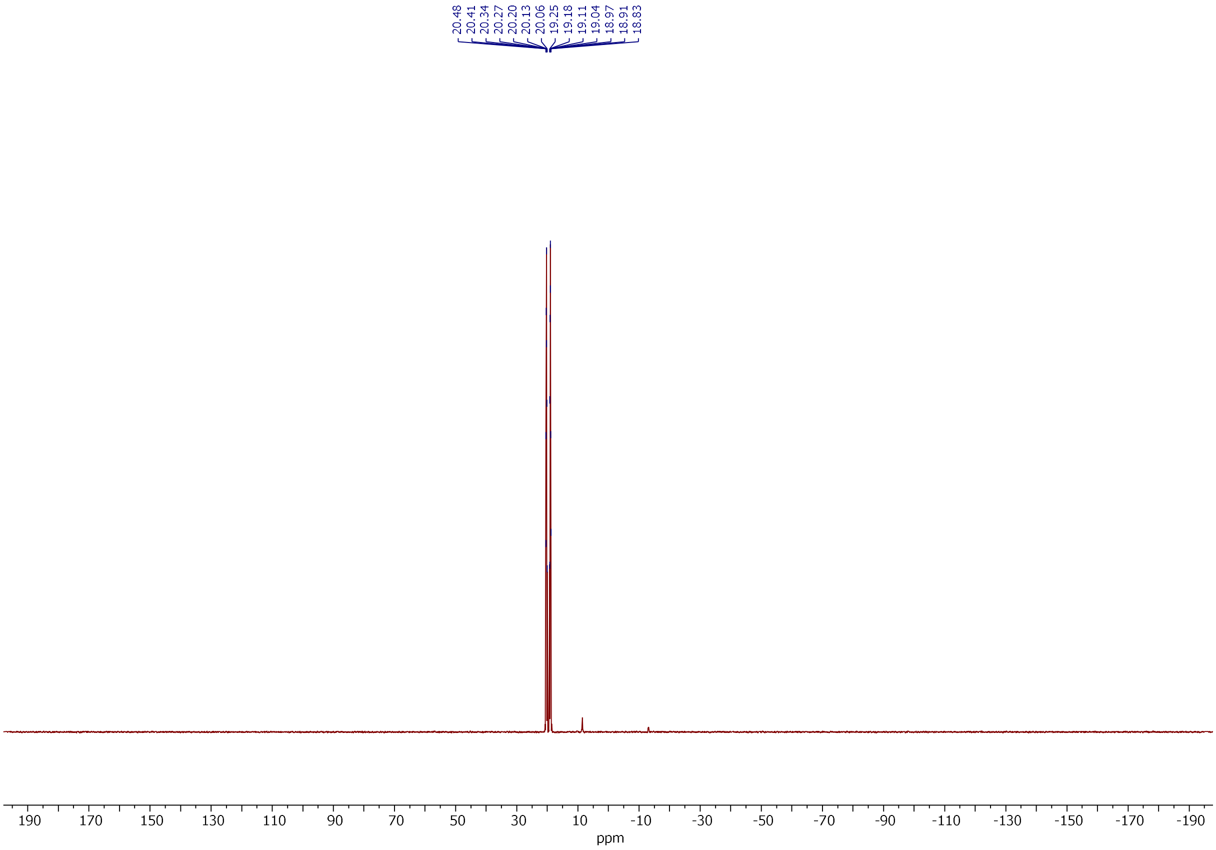

*Figure S113. ^{31}P NMR spectrum of the **1**^{Cs} (5 mol%) catalysed HP reaction of **9** with ^{t}Bu$_2$PH in benzene-d$_6$ at 300 K after 19 h at 90 °C. No conversion ^{t}Bu$_2$PH (19.6 ppm).*

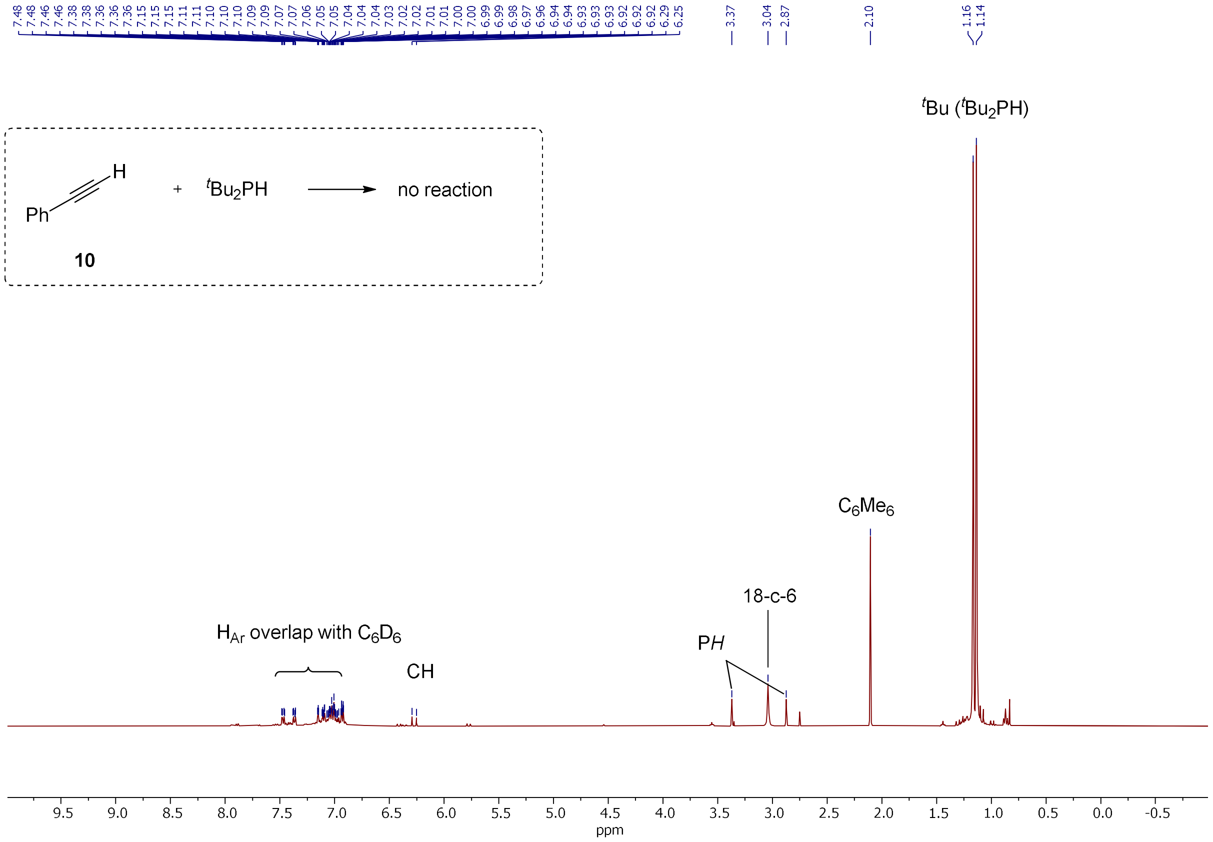

*Figure S114. ^{1}H NMR spectrum of the $\mathbf{1}^{Cs}$ (5 mol%) catalysed HP reaction of **10** with $^{t}Bu_2PH$ in benzene-d_6 at 300 K after 19 h at 90 °C. C_6Me_6 as internal standard, no conversion, mixture of substrates.*

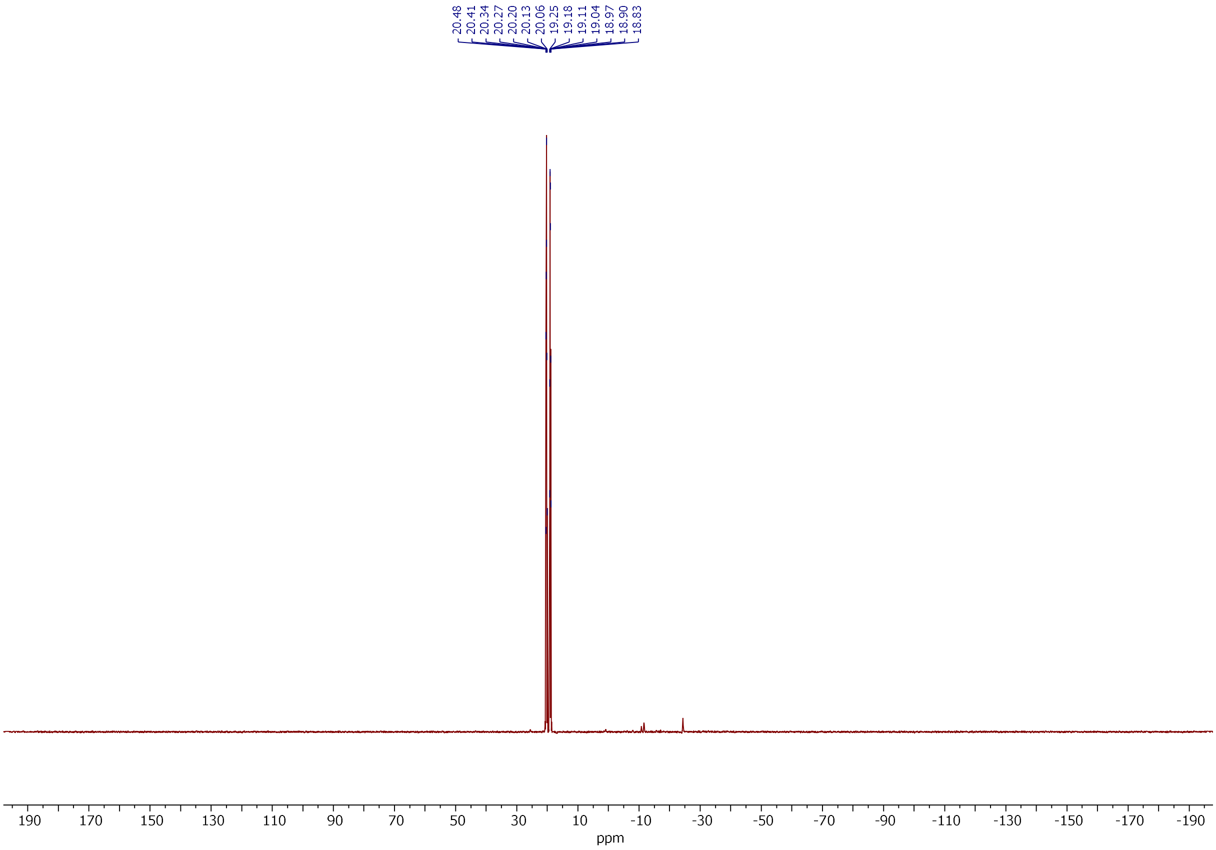

*Figure S115. ^{31}P NMR spectrum of the $\mathbf{1}^{Cs}$ (5 mol%) catalysed HP reaction of **10** with $^{t}Bu_2PH$ in benzene-d_6 at 300 K after 19 h at 90 °C. No conversion $^{t}Bu_2PH$ (19.6 ppm).*

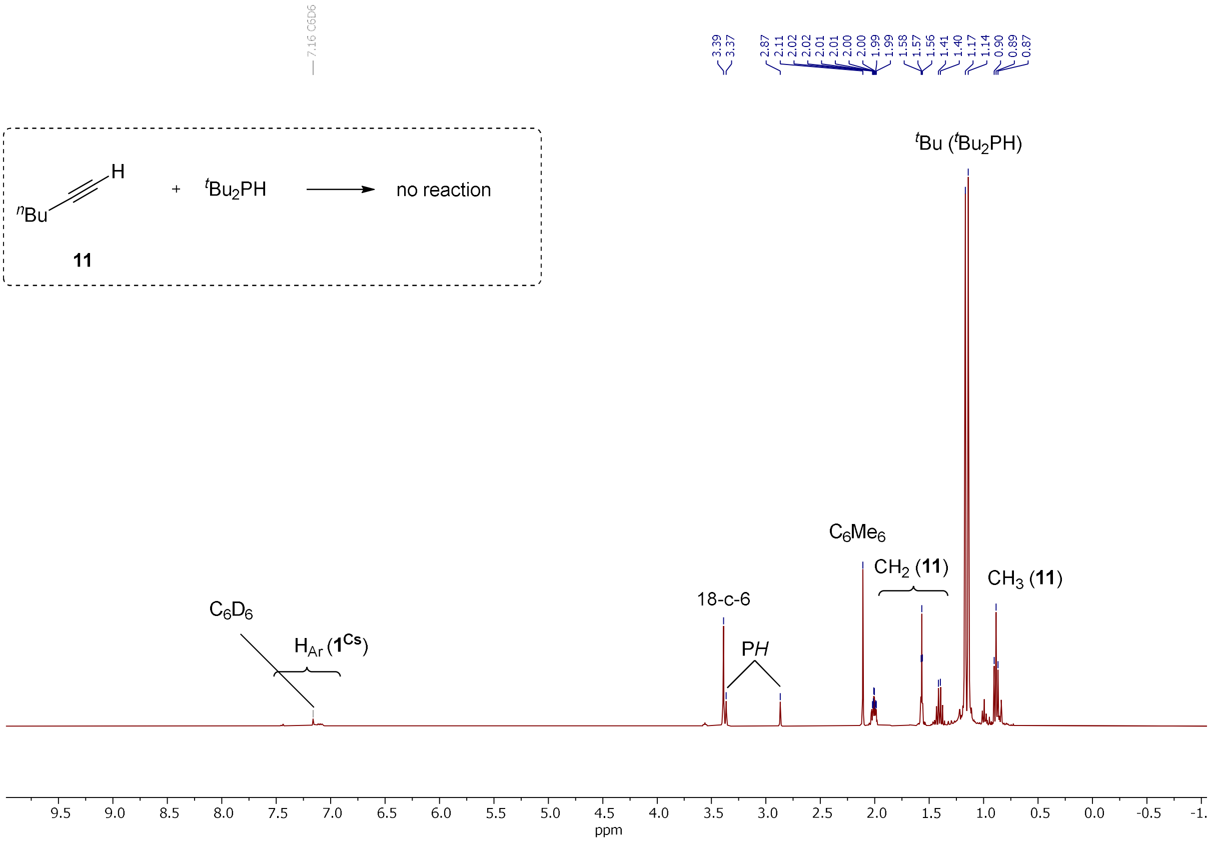

*Figure S116. ^1H NMR spectrum of the $\mathbf{1^{Cs}}$ (5 mol%) catalysed HP reaction of **11** with $^tBu_2PH$ in benzene-d_6 at 300 K after 19 h at 90 °C. C_6Me_6 as internal standard, no conversion, mixture of substrates.*

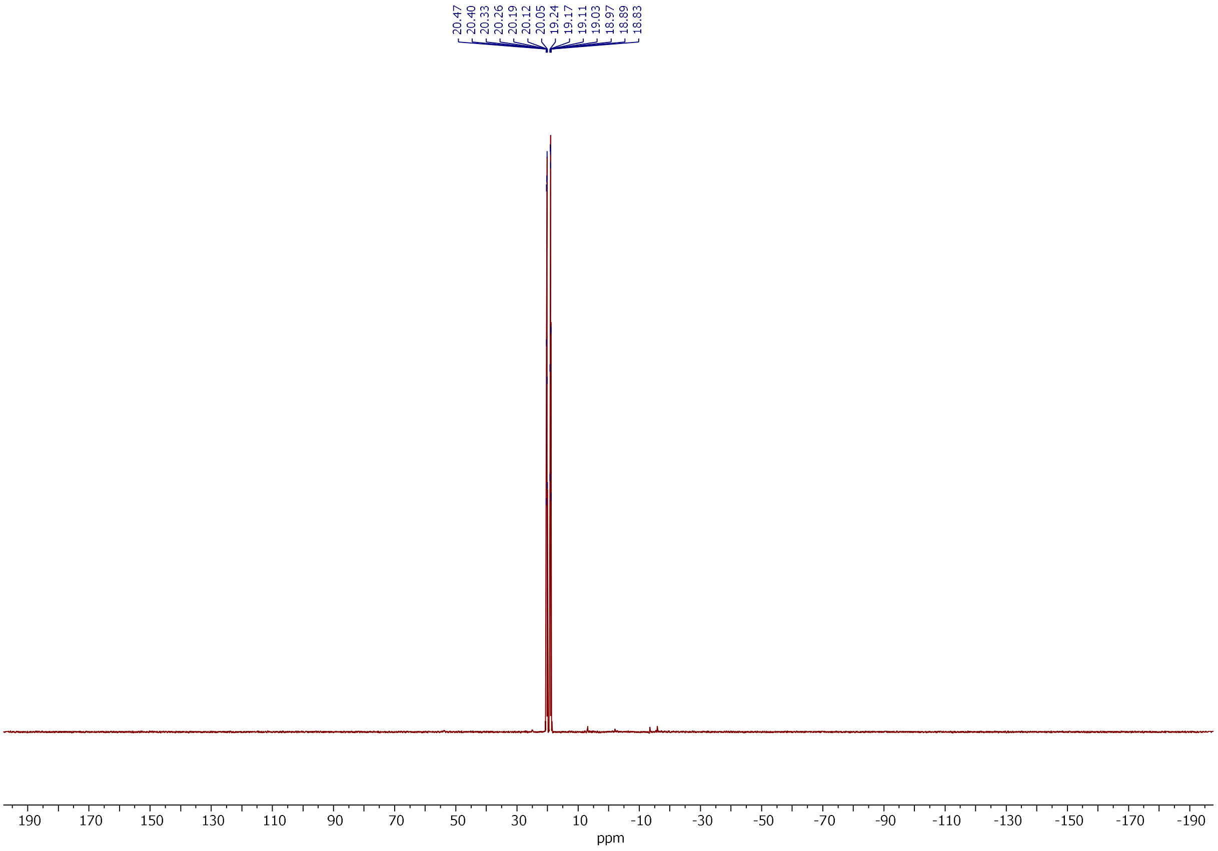

*Figure S117. ^{31}P NMR spectrum of the $\mathbf{1^{Cs}}$ (5 mol%) catalysed HP reaction of **11** with $^tBu_2PH$ in benzene-d_6 at 300 K after 19 h at 90 °C. No conversion $^tBu_2PH$ (19.6 ppm).*

nBu_2PH

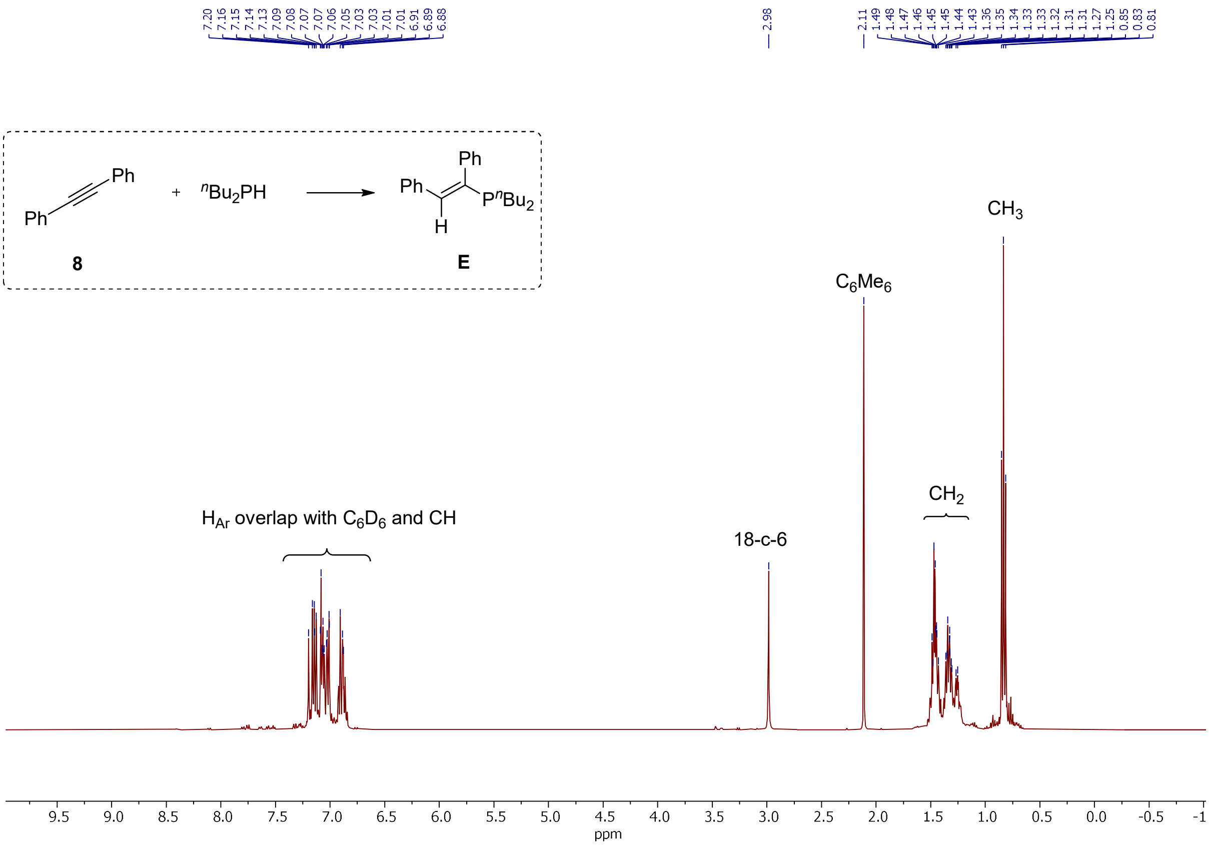

Figure S118. ^1H *NMR spectrum of the* $\mathbf{1^{Cs}}$ *(5 mol%) catalysed HP reaction of* **8** *with* $^nBu_2PH$ *in benzene-*d_6 *at 300 K after 4 h at 90 °C.* C_6Me_6 *as internal standard, 99 % conversion.*

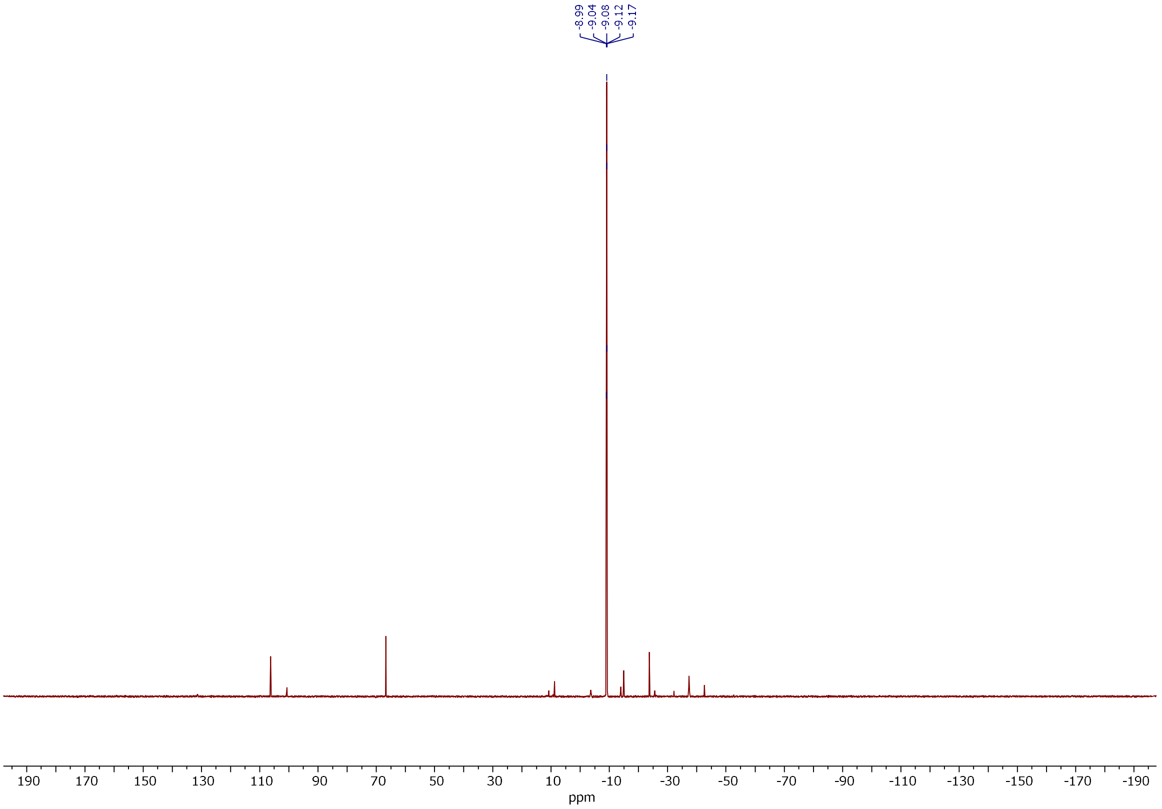

Figure S119. $^{31}P\{^1H\}$ *NMR spectrum of the* $\mathbf{1^{Cs}}$ *(5 mol%) catalysed HP reaction of* **8** *with* $^tBu_2PH$ *in benzene-*d_6 *at 300 K after 4 h at 90 °C. 99 % conversion, product (-9 ppm) and minor unknown side products.*

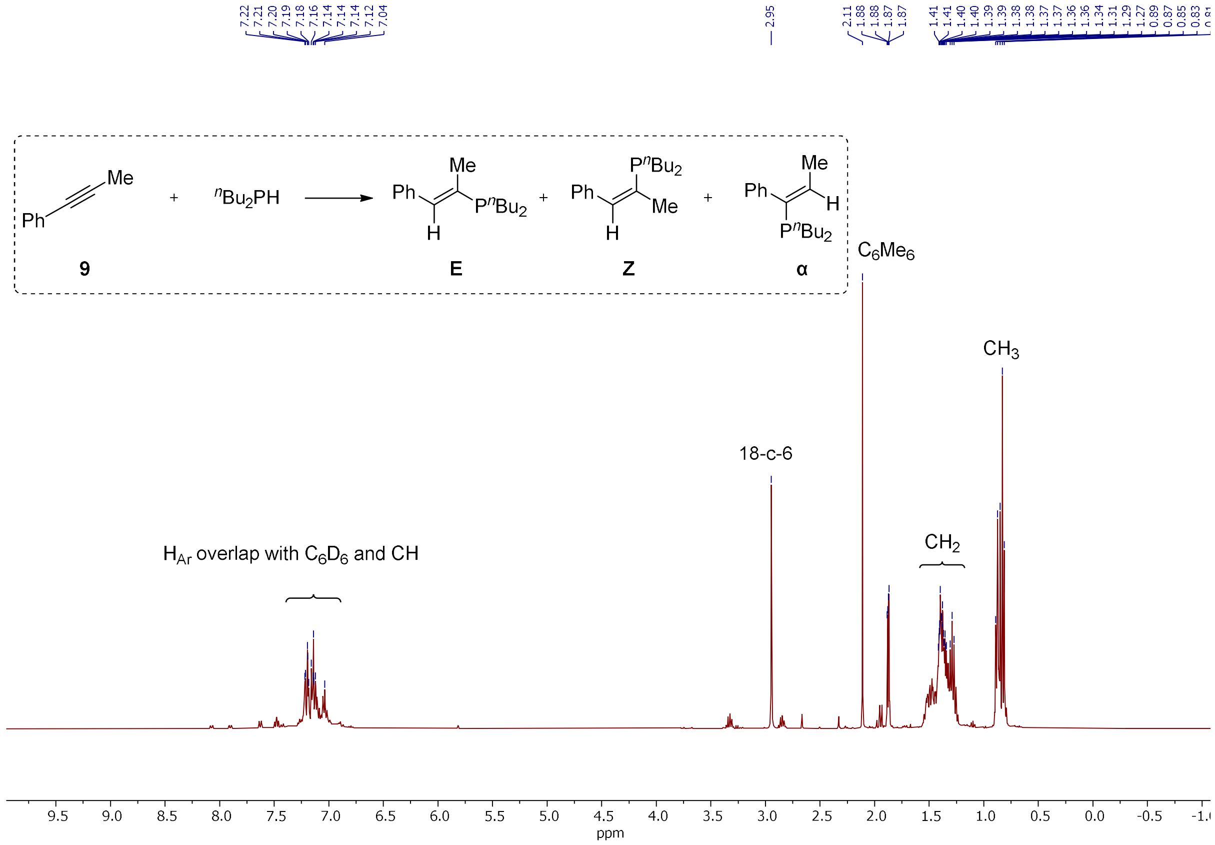

*Figure S120. ^{1}H NMR spectrum of the $\mathbf{1^{Cs}}$ (5 mol%) catalysed HP reaction of **9** with ^{n}Bu$_2$PH in benzene-d$_6$ at 300 K after 19 h at 90 °C. C$_6$Me$_6$ as internal standard, 50 % conversion. Mixture of substrates and products.*

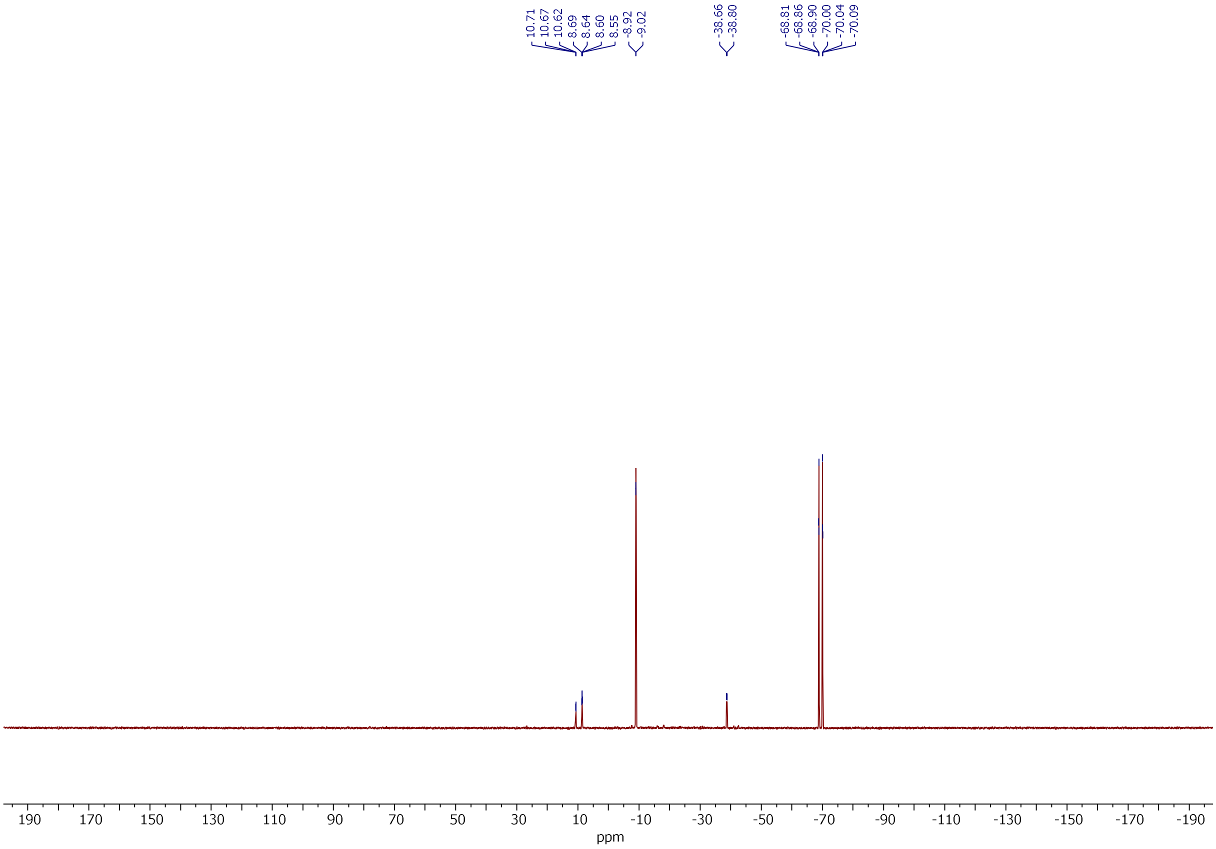

*Figure S121. ^{31}P NMR spectrum of the $\mathbf{1^{Cs}}$ (5 mol%) catalysed HP reaction of **9** with ^{n}Bu$_2$PH in benzene-d$_6$ at 300 K after 19 h at 90 °C. C$_6$Me$_6$ as internal standard, 50 % conversion. Mixture of ^{n}Bu$_2$PH (-70 ppm) and products.*

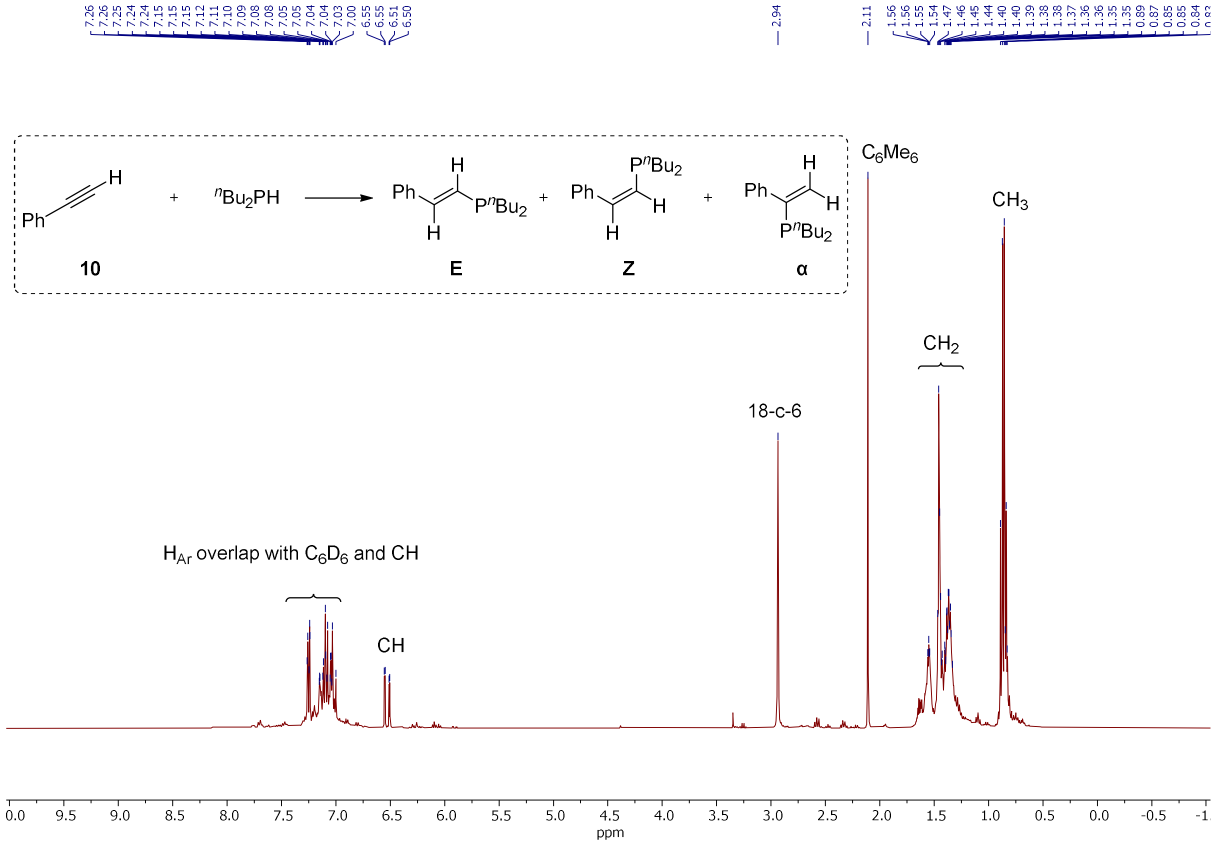

*Figure S122. ^{1}H NMR spectrum of the **1^{Cs}** (5 mol%) catalysed HP reaction of **10** with $^{n}Bu_2PH$ in benzene-d_6 at 300 K after 19 h at 90 °C. C_6Me_6 as internal standard, 96 % conversion. Mixture of products.*

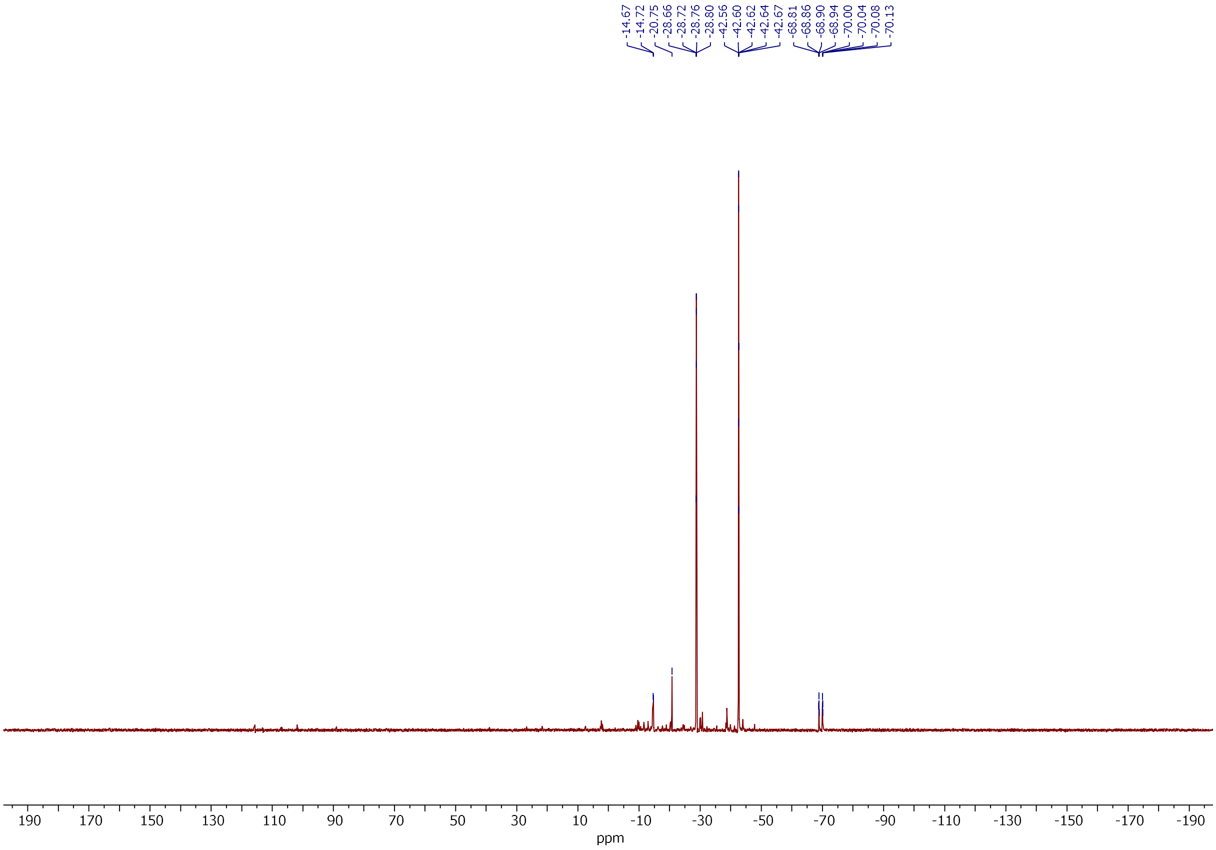

*Figure S123. ^{31}P NMR spectrum of the **1^{Cs}** (5 mol%) catalysed HP reaction of **10** with $^{n}Bu_2PH$ in benzene-d_6 at 300 K after 19 h at 90 °C. 96 % conversion. Mixture of $^{n}Bu_2PH$ (-70 ppm) and products.*

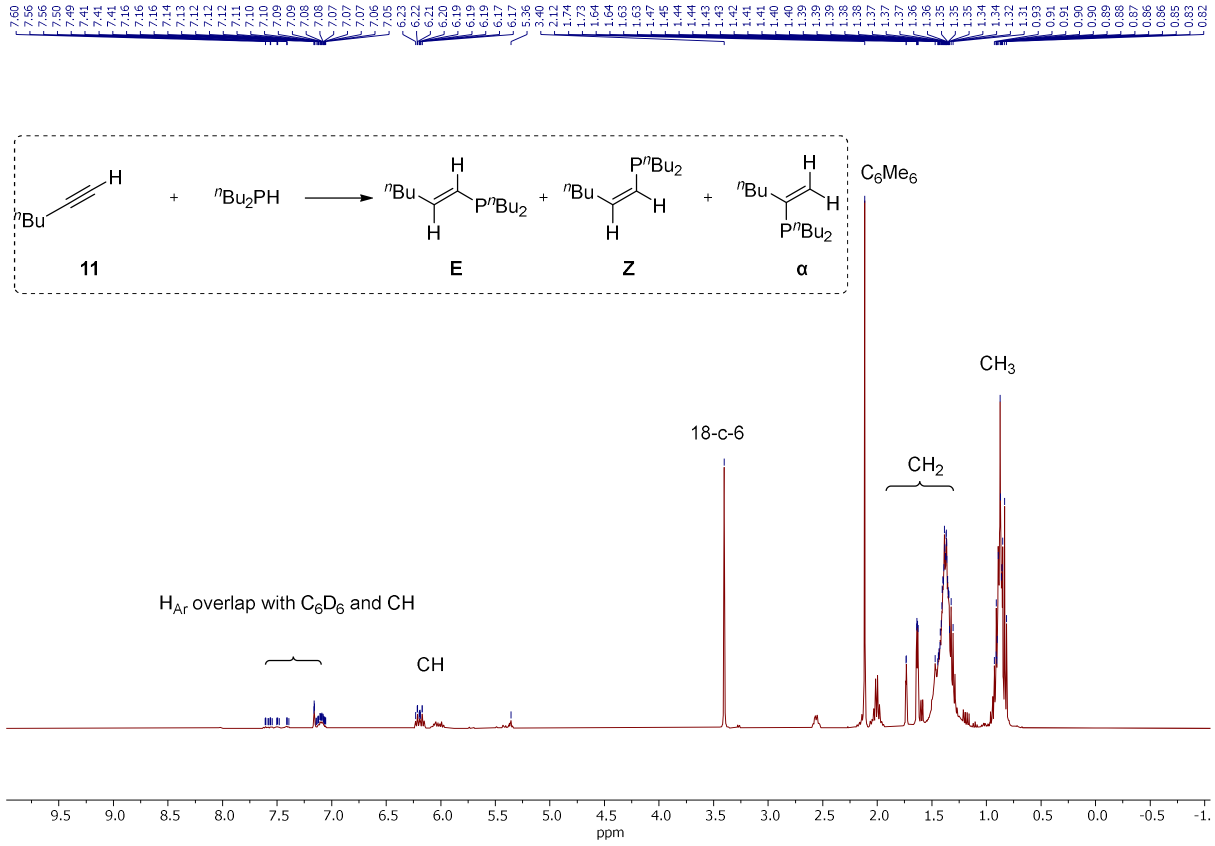

*Figure S124. ^{1}H NMR spectrum of the $\mathbf{1}^{\mathbf{Cs}}$ (5 mol%) catalysed HP reaction of **11** with ^{n}Bu$_2$PH in benzene-d$_6$ at 300 K after 4 h at 90 °C. C$_6$Me$_6$ as internal standard, 98 % conversion. Mixture of products.*

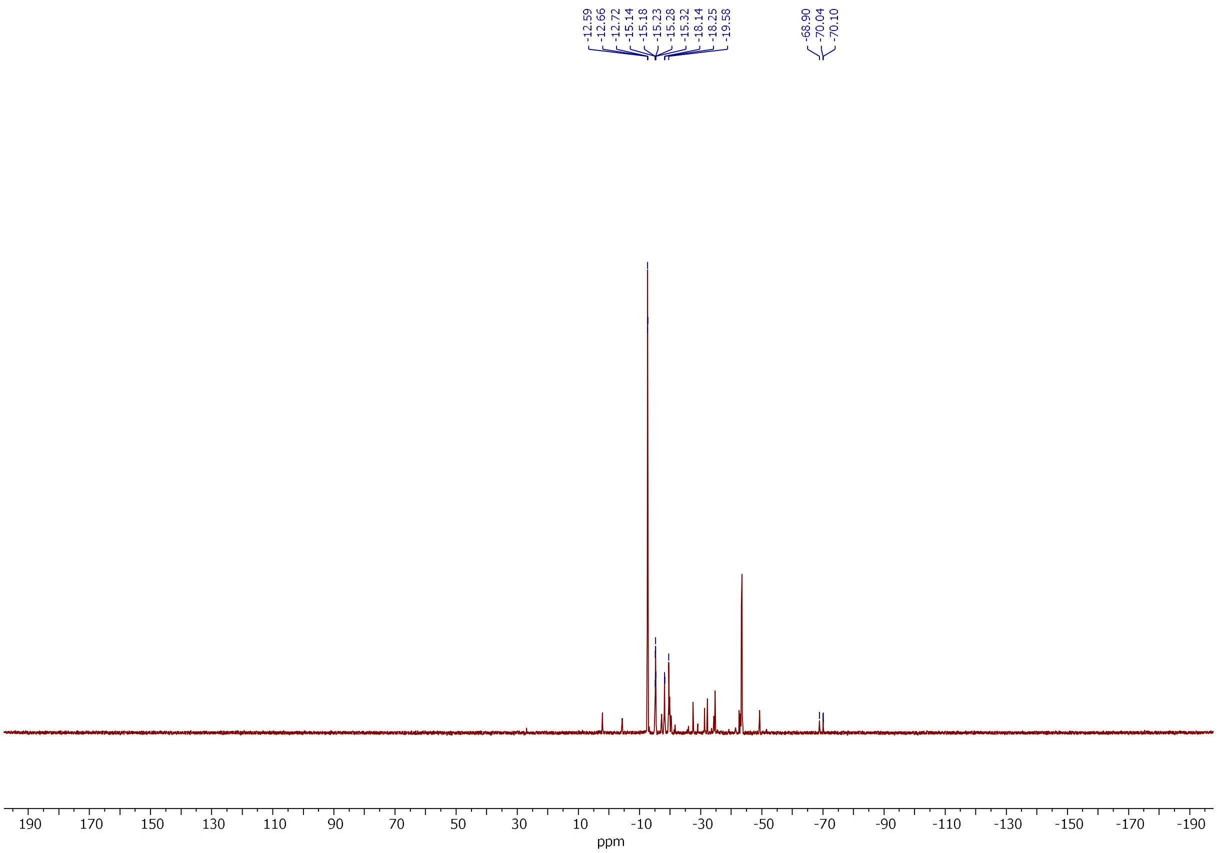

*Figure S125. ^{31}P NMR spectrum of the $\mathbf{1}^{\mathbf{Cs}}$ (5 mol%) catalysed HP reaction of **11** with ^{n}Bu$_2$PH in benzene-d$_6$ at 300 K after 4 h at 90 °C. 96 % conversion. Mixture of ^{n}Bu$_2$PH (-70 ppm) products.*

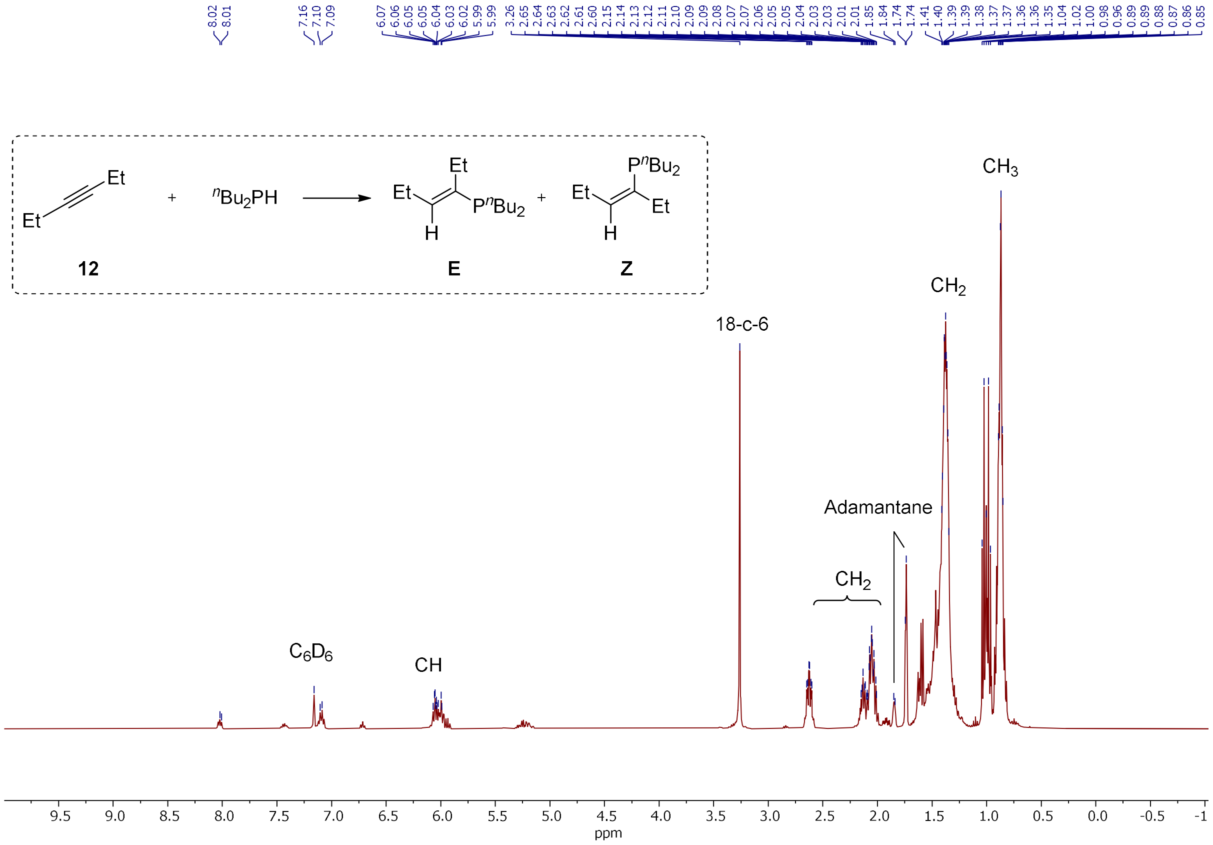

*Figure S126. ^{1}H NMR spectrum of the **1^{Cs}** (5 mol%) catalysed HP reaction of **12** with ^{n}Bu$_2$PH in benzene-d$_6$ at 300 K after 23 h at 90 °C. C$_6$Me$_6$ as internal standard, 85 % conversion. Mixture of products.*

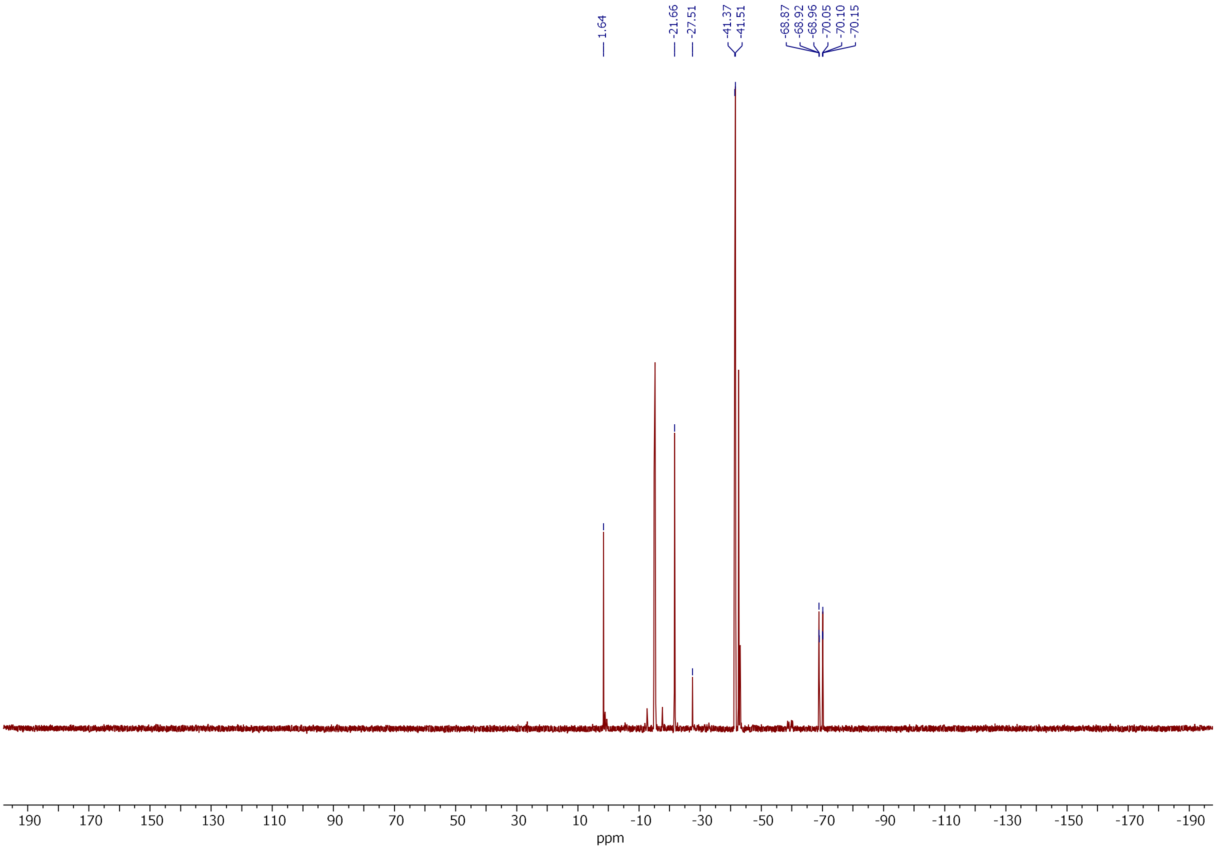

*Figure S127. ^{31}P NMR spectrum of the **1^{Cs}** (5 mol%) catalysed HP reaction of **12** with ^{n}Bu$_2$PH in benzene-d$_6$ at 300 K after 23 h at 90 °C. 85 % conversion. Mixture of ^{n}Bu$_2$PH (-70 ppm) products.*

Section S3.4 – NMR Spectra of the catalytic hydrophosphination reactions of additional substrates 13-17

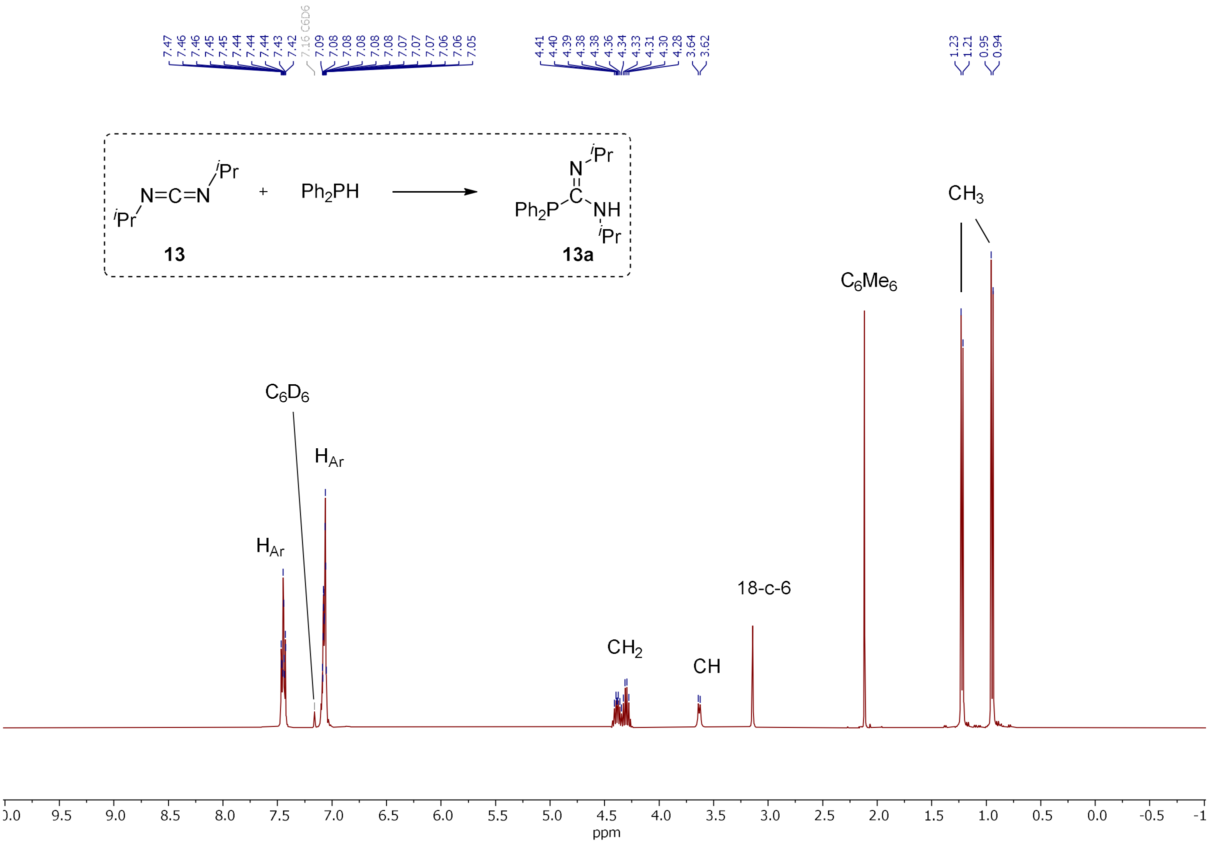

*Figure S128. ^{1}H NMR spectrum of the $\mathbf{1^{Cs}}$ catalysed HP reaction of **13** with Ph_2PH in benzene-d_6 at 300 K after 30 min at RT.*

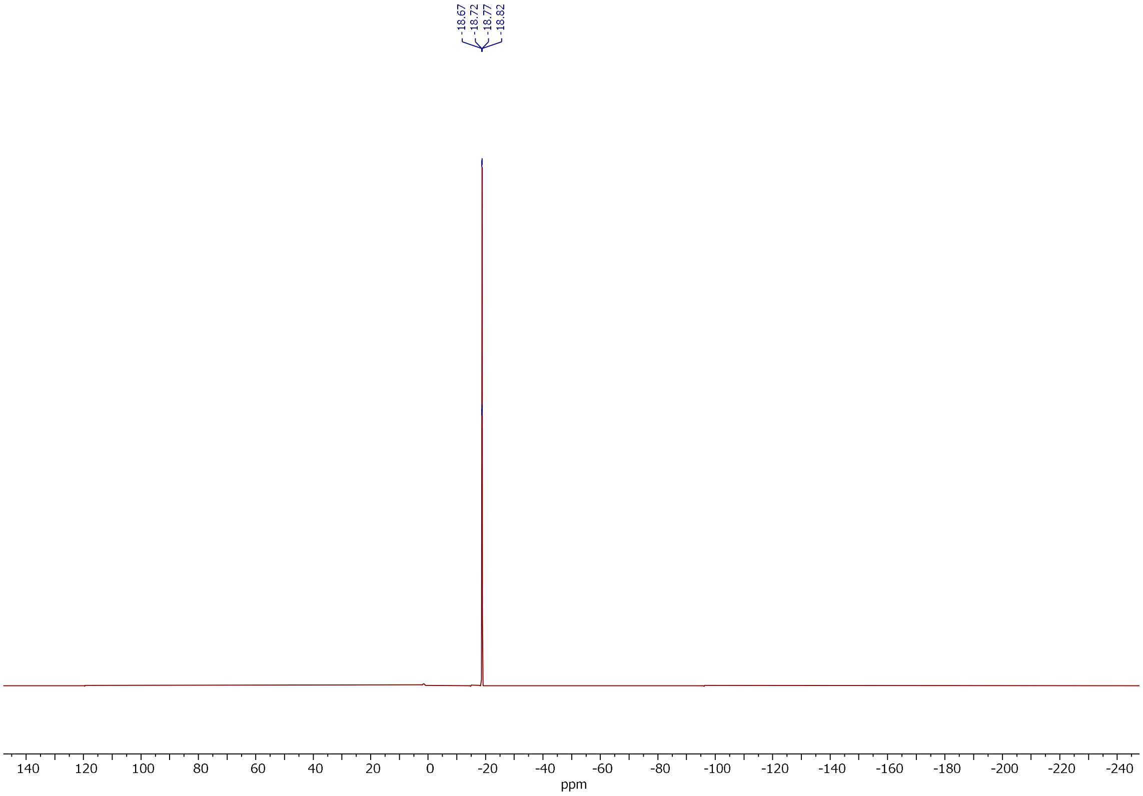

*Figure S129. ^{31}P NMR spectrum of the $\mathbf{1^{Cs}}$ catalysed HP reaction of **13** with Ph_2PH in benzene-d_6 at 300 K after 30 min at RT.*

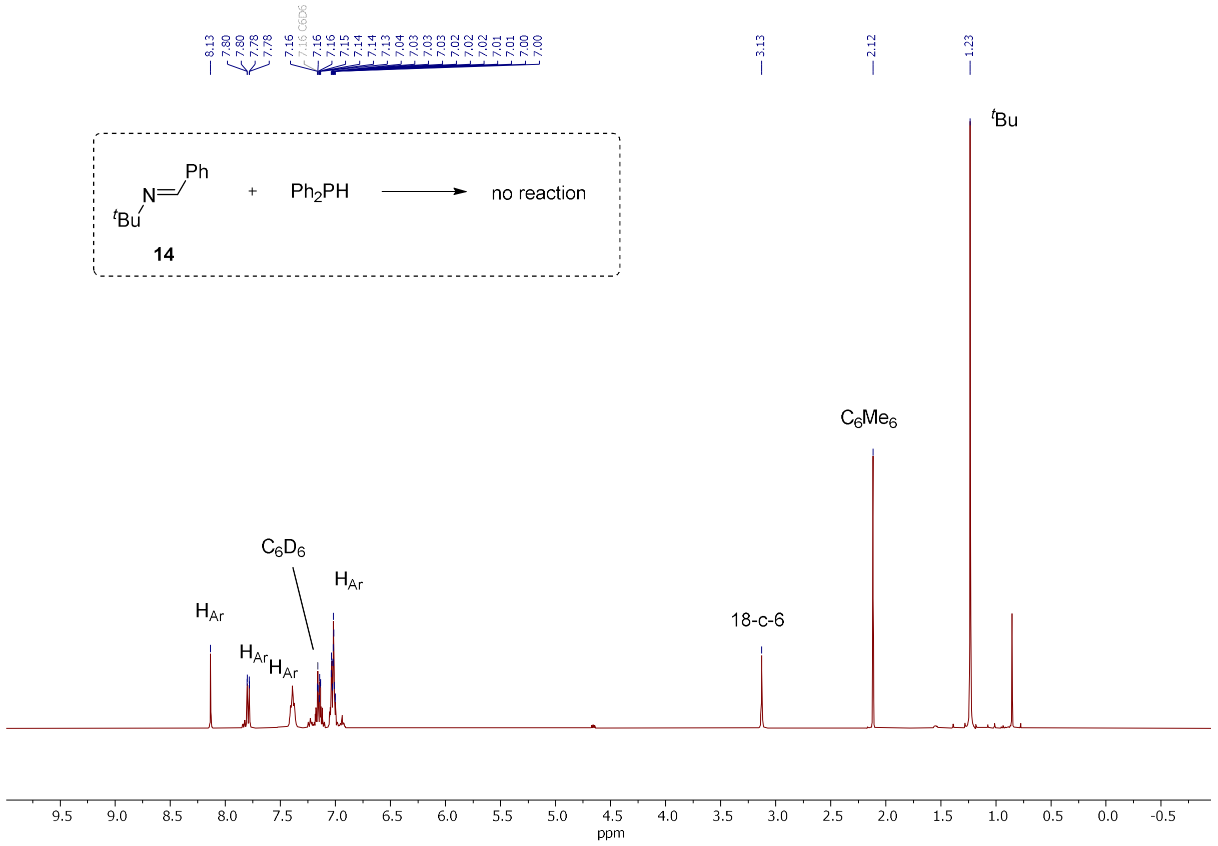

*Figure S130. ^{1}H NMR spectrum of the $\mathbf{1^{Cs}}$ catalysed HP reaction of **14** with Ph_2PH in benzene-d_6 at 300 K after 2 h at 75 °C. No conversion observed.*

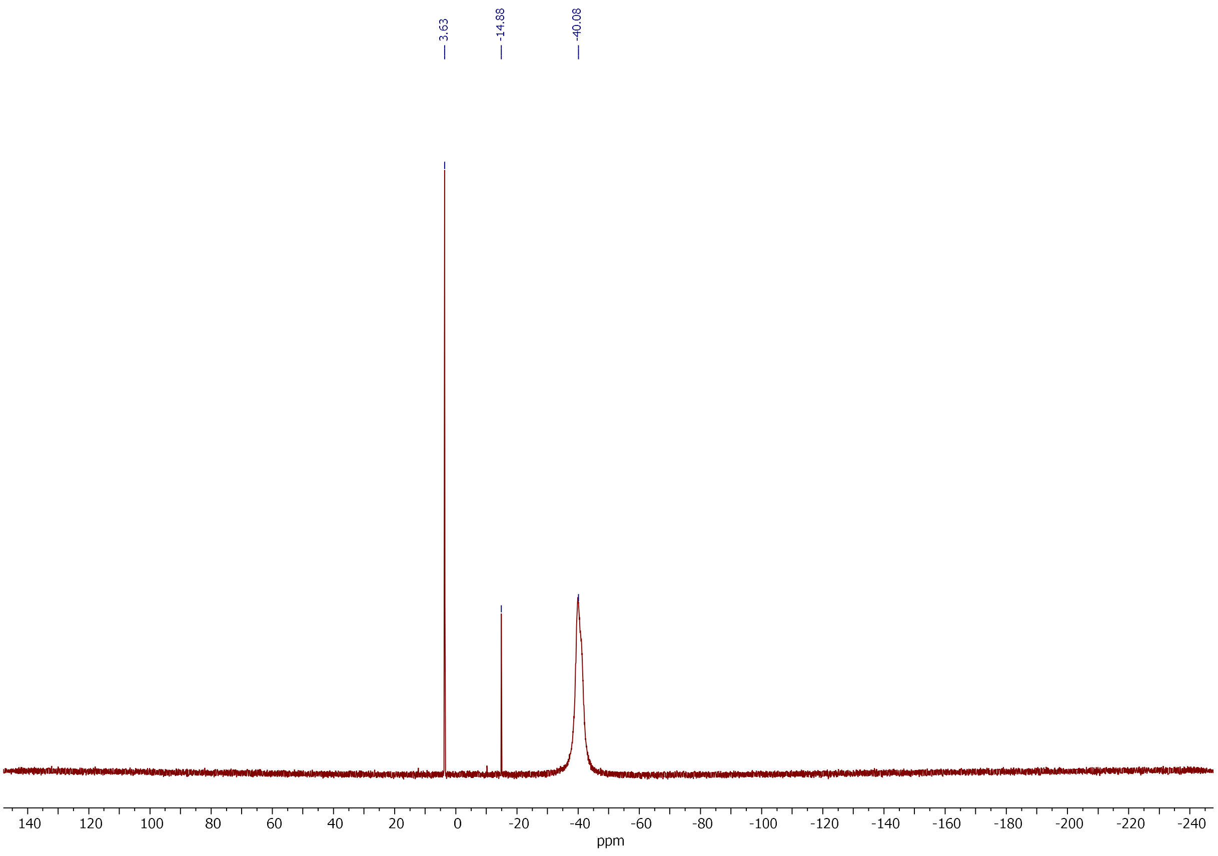

*Figure S131. ^{31}P NMR spectrum of the $\mathbf{1^{Cs}}$ catalysed HP reaction of **14** with Ph_2PH in benzene-d_6 at 300 K after 2 h at 75 °C. No conversion observed. Minor signals at 3.6 and -14.9 ppm belong to unknown side products.*

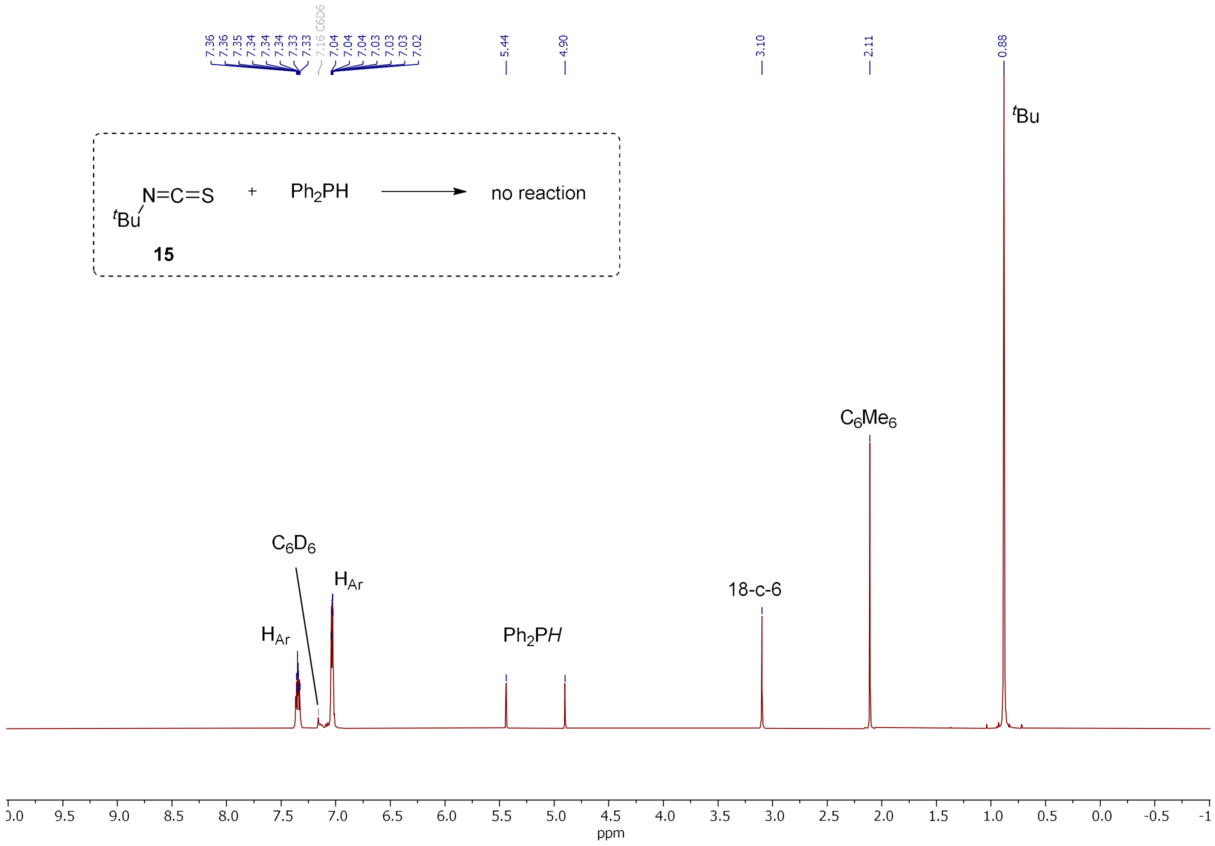

*Figure S132. ^{1}H NMR spectrum of the **1^{Cs}** catalysed HP reaction of **15** with Ph_2PH in benzene-d_6 at 300 K after 30 min at RT. No conversion observed, catalyst decomposes which is indicated by decolouration of the reaction mixture.*

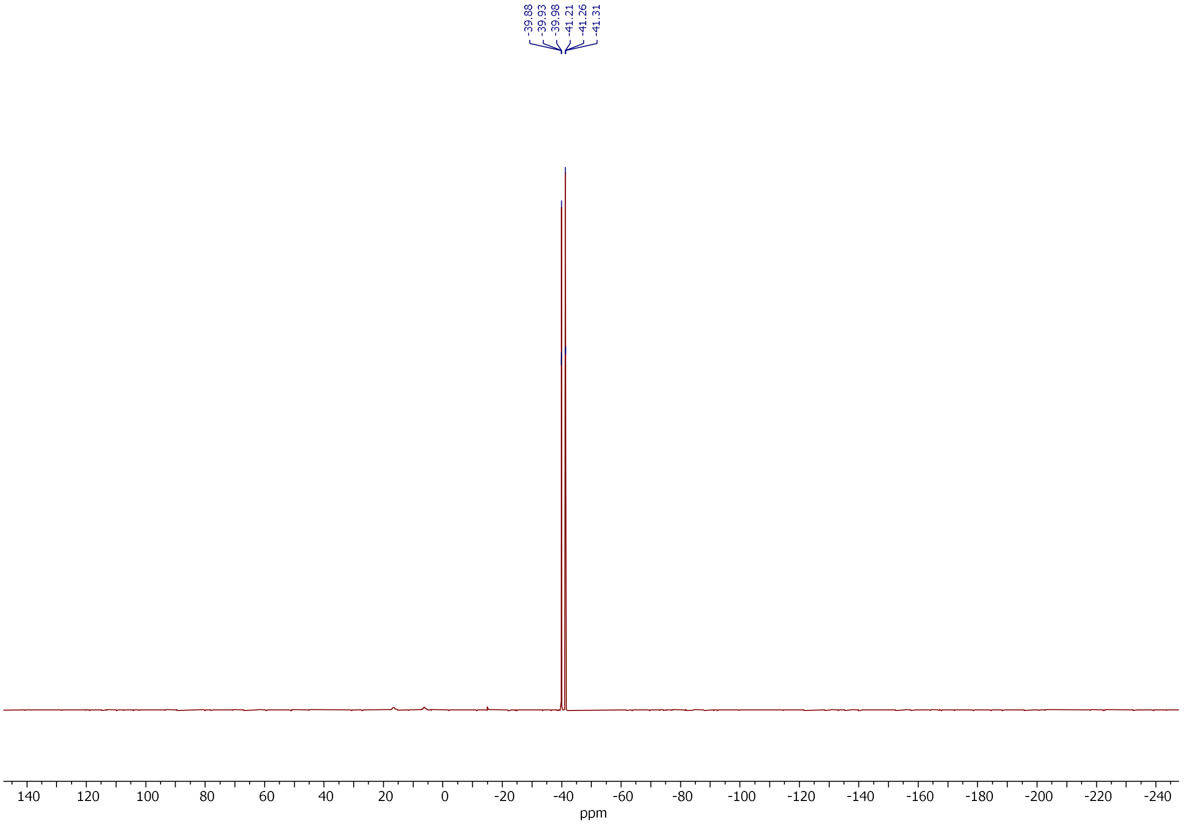

*Figure S133. ^{31}P NMR spectrum of the **1^{Cs}** catalysed HP reaction of **15** with Ph_2PH in benzene-d_6 at 300 K after 30 min at RT. No conversion observed, only Ph2PH visible. Catalyst decomposes which is indicated by decolouration of the reaction mixture.*

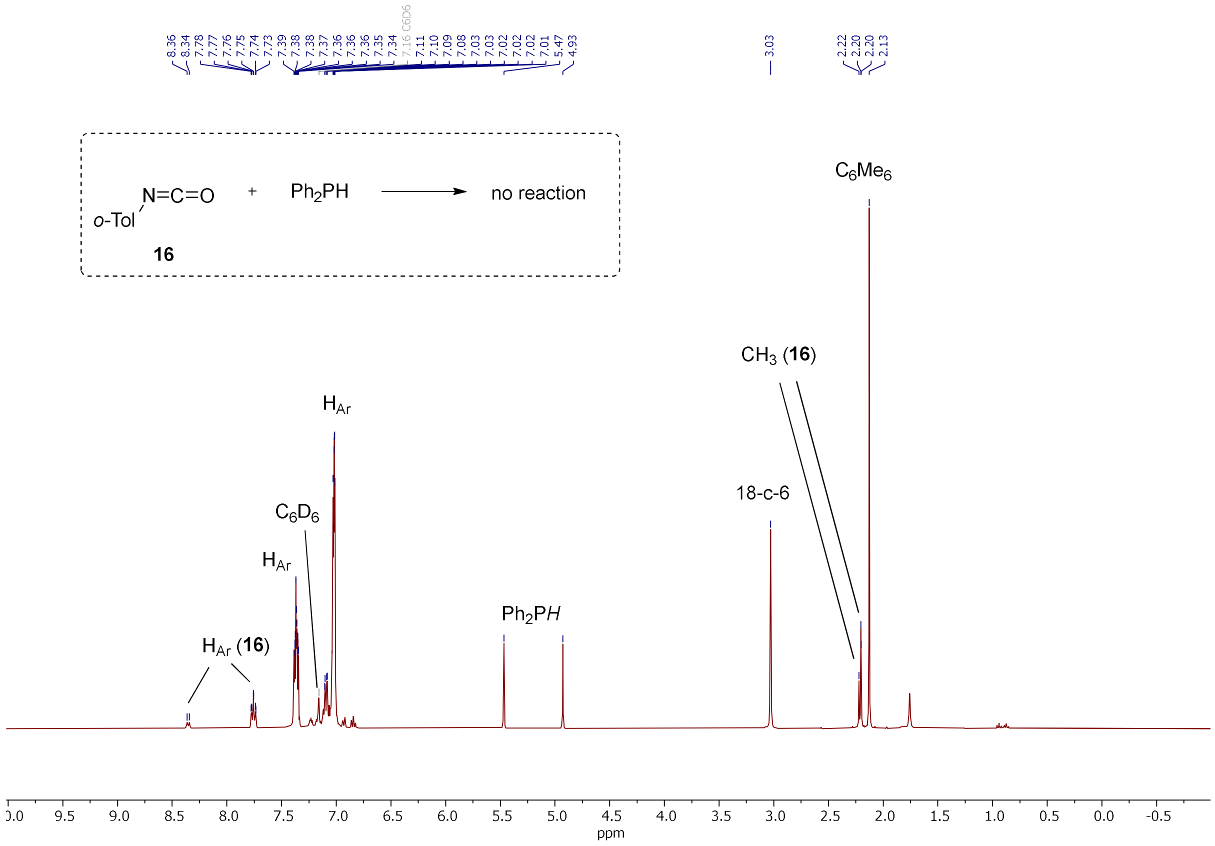

*Figure S134. ^{1}H NMR spectrum of the **1**^{Cs} catalysed HP reaction of **16** with Ph_2PH in benzene-d_6 at 300 K after 20 min at RT. No product observed, formation of a colourless solid and disappearance of the substrate signals suggest cyclotrimerisation reaction.*

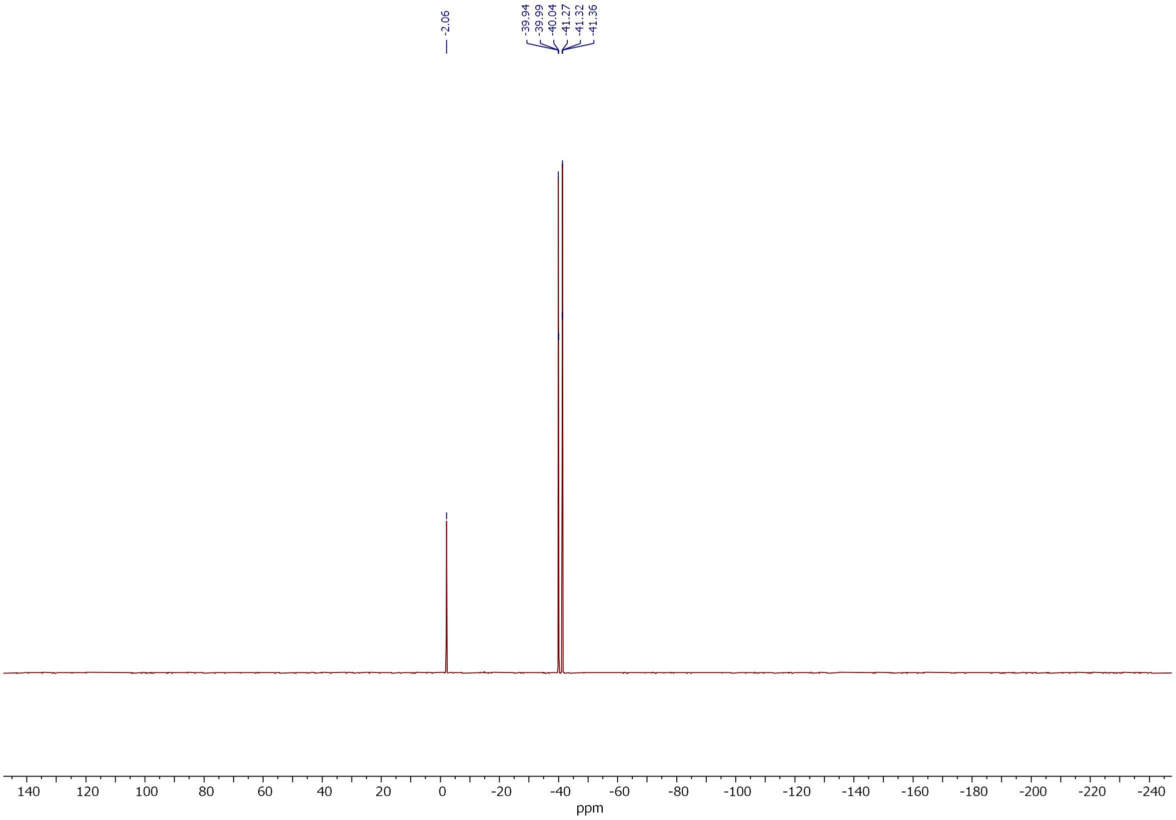

*Figure S135. ^{31}P NMR spectrum of the **1**^{Cs} catalysed HP reaction of **16** with Ph_2PH in benzene-d_6 at 300 K after 20 min at RT. No product observed, only Ph_2PH visible in the 31P spectrum. Minor signal at -2 ppm may belongs to decomposed catalyst.*

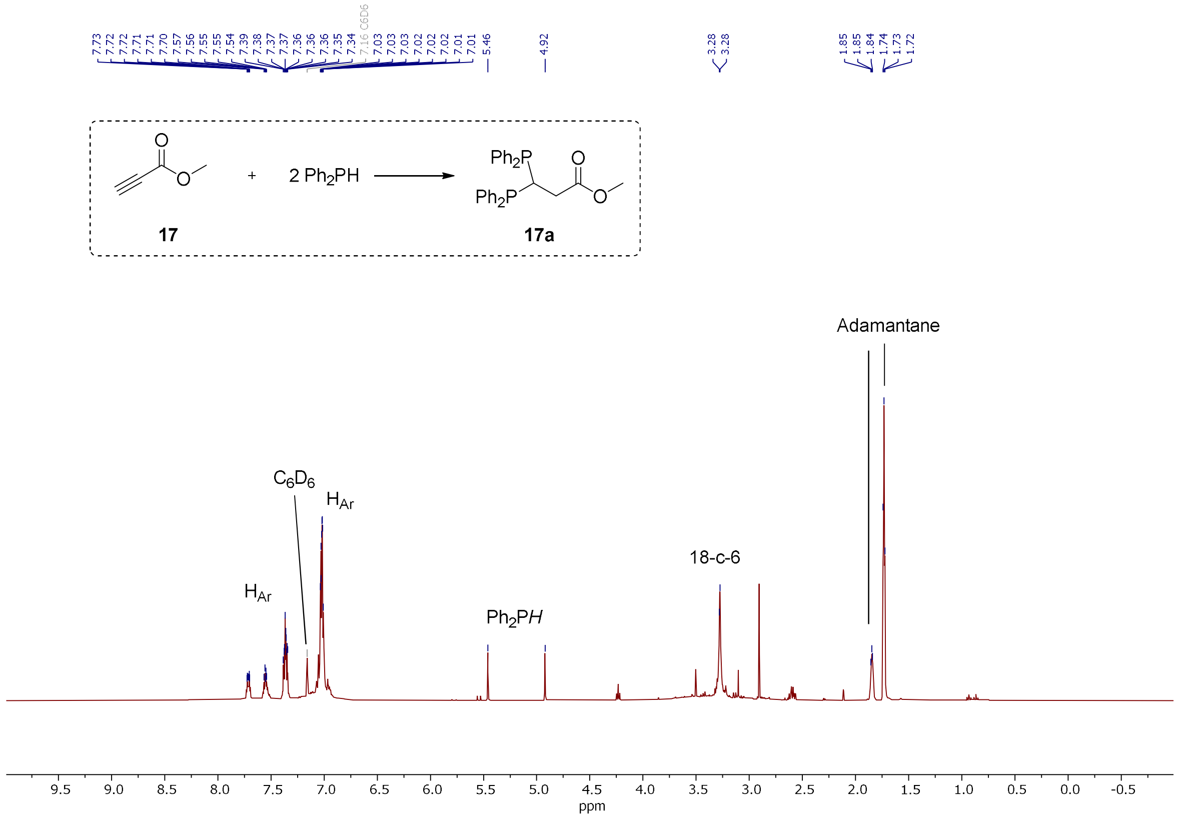

Figure S136. *^{1}H NMR spectrum of the* ***1^{Cs}*** *catalysed double HP reaction of* ***17*** *with Ph_2PH in benzene-d_6 at 300 K after 1 h at RT with direct addition of the substrate to the catalyst solution. Minor product observed, disappearance of the substrate signals and formation of broad signals suggest polymerisation of* ***17****.*

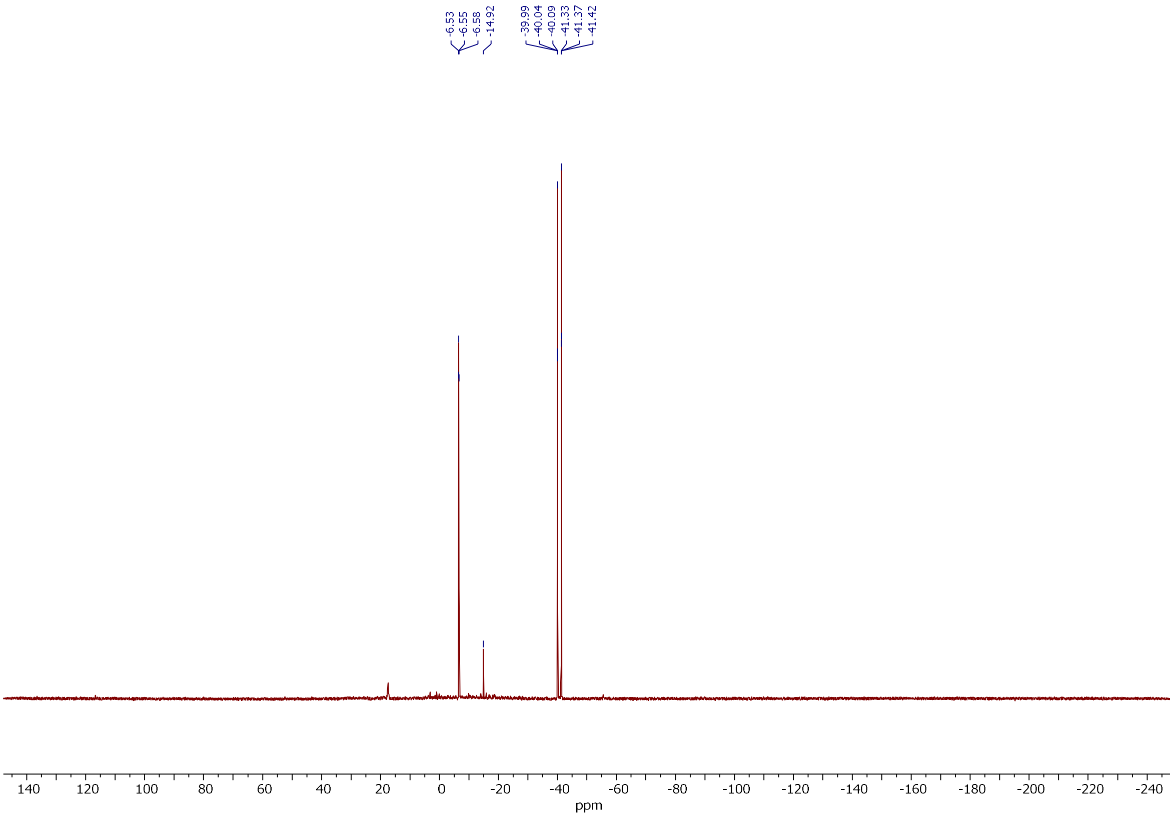

Figure S137. *^{31}P NMR spectrum of the* ***1^{Cs}*** *catalysed double HP reaction of* ***17*** *with Ph_2PH in benzene-d_6 at 300 K after 1 h at RT with direct addition of the substrate to the catalyst solution. Minor product observed (6.5 ppm), mostly Ph_2PH (-40 ppm) and minor mono hydrophosphinated product (-14 ppm).*

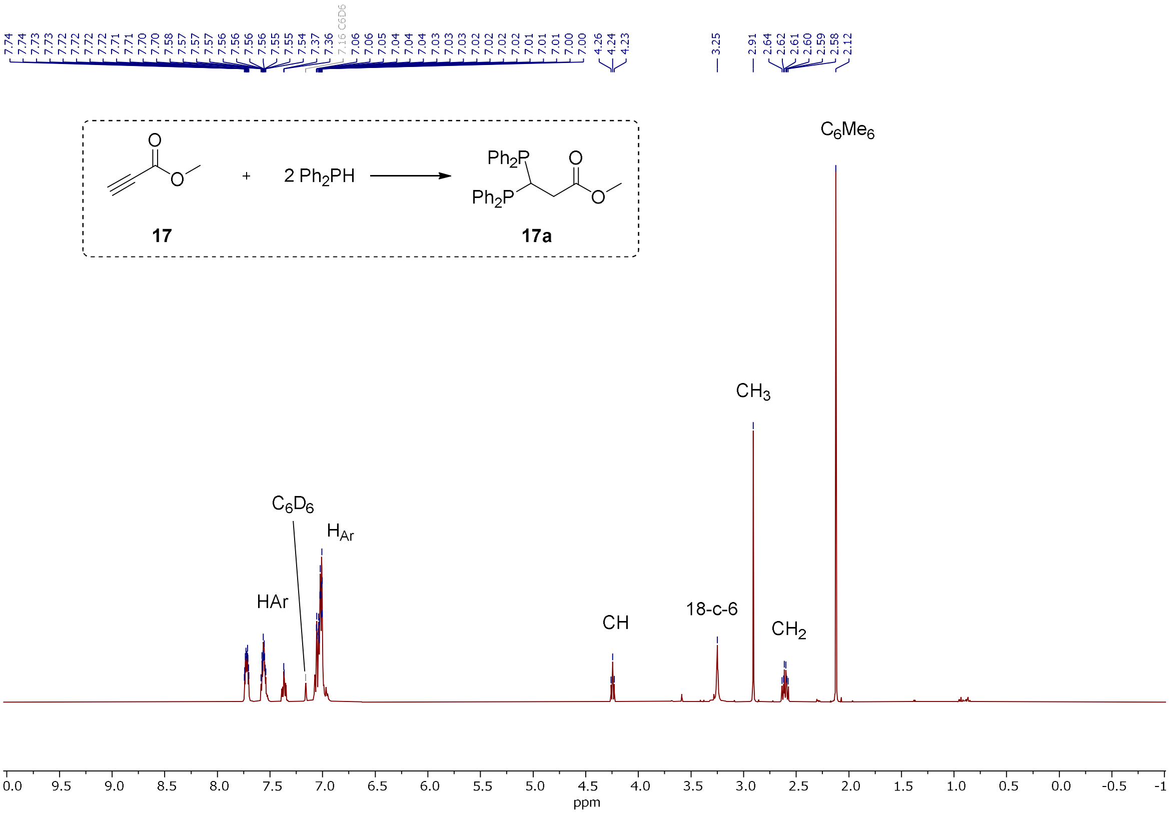

*Figure S138. ^{1}H NMR spectrum of the $\mathbf{1^{Cs}}$ catalysed double HP reaction of **17** with Ph_2PH in benzene-d_6 at 300 K after 20 min at RT after dropwise addition of the substrate diluted in benzene-d_6 to the catalyst solution over a period of 5 min.*

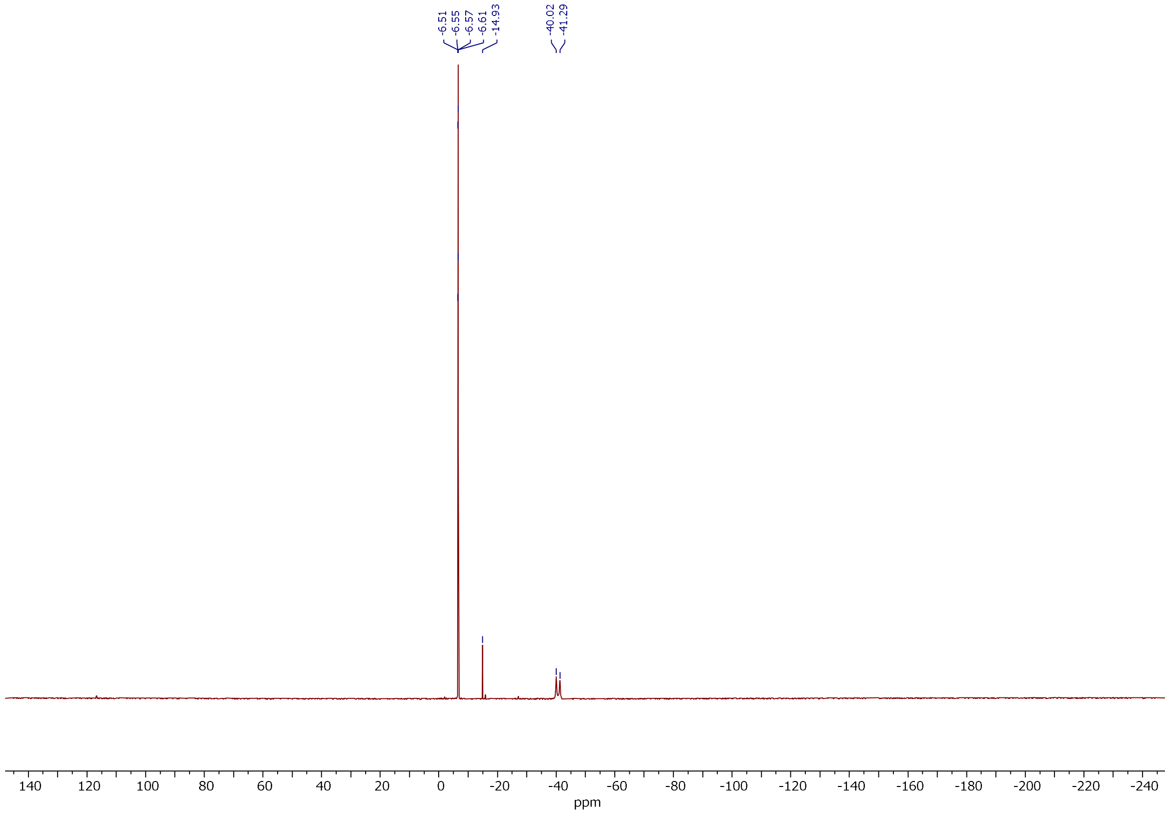

*Figure S139. ^{1}H NMR spectrum of the $\mathbf{1^{Cs}}$ catalysed double HP reaction of **17** with Ph_2PH in benzene-d_6 at 300 K after 20 min at RT after dropwise addition of the substrate diluted in benzene-d_6 to the catalyst solution over a period of 5 min. Major peak for the product observed (6.5 ppm), minor Ph_2PH (-40 ppm) and minor mono hydrophosphinated product (-14 ppm).*

Section S3.5 – NMR Spectra of the catalytic hydrophosphination reactions of Ph_2P(vinyl) 18

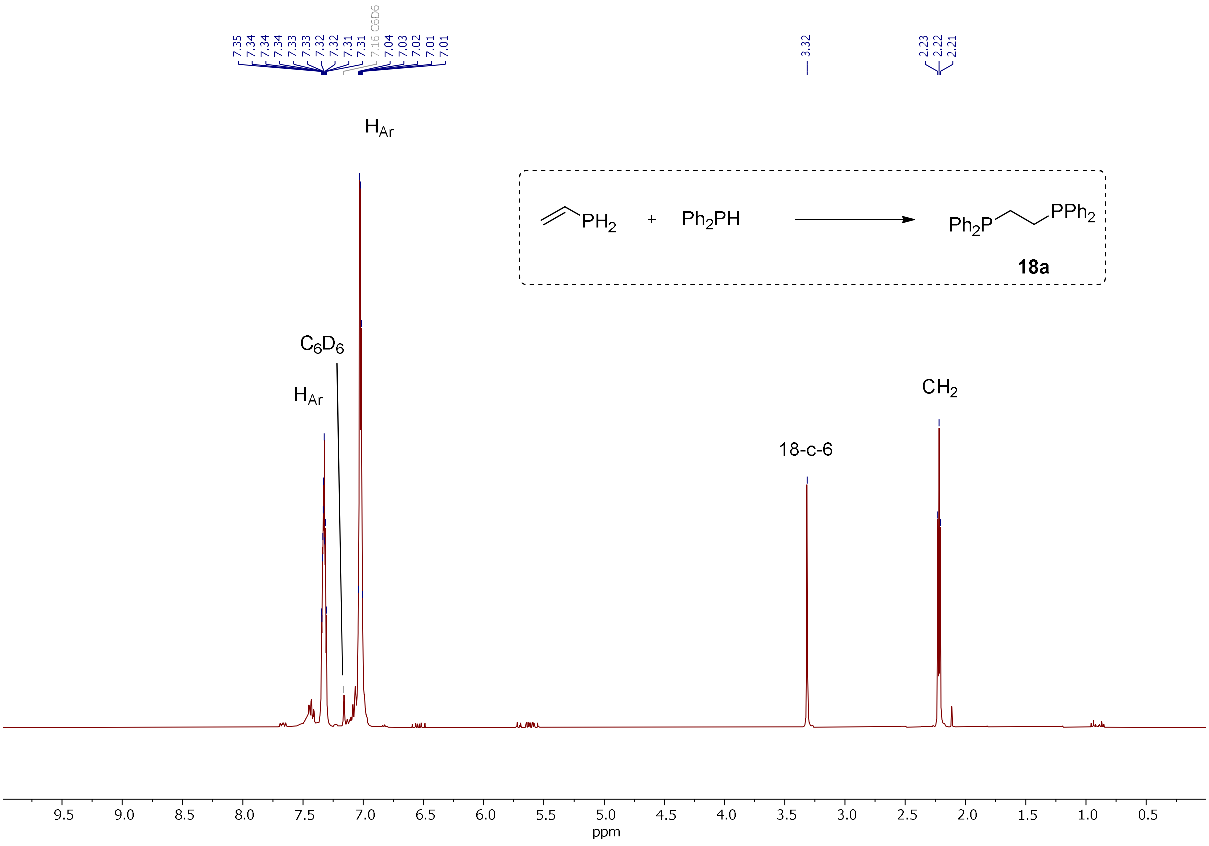

*Figure S140. ¹H NMR spectrum of the HP reaction of **18** with Ph_2PH in benzene-d_6 at 300 K after 12 min at RT.*

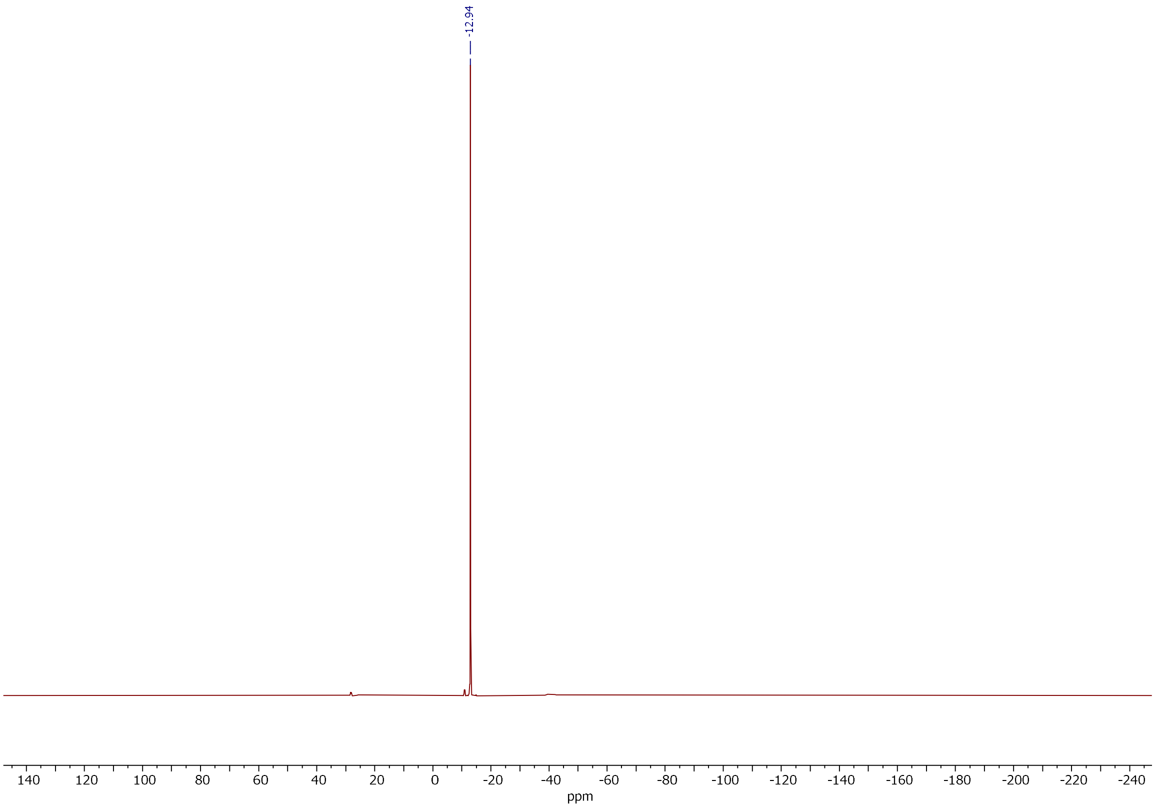

*Figure S141. ³¹P NMR spectrum of the HP reaction of **18** with Ph_2PH in benzene-d_6 at 300 K after 12 min at RT.*

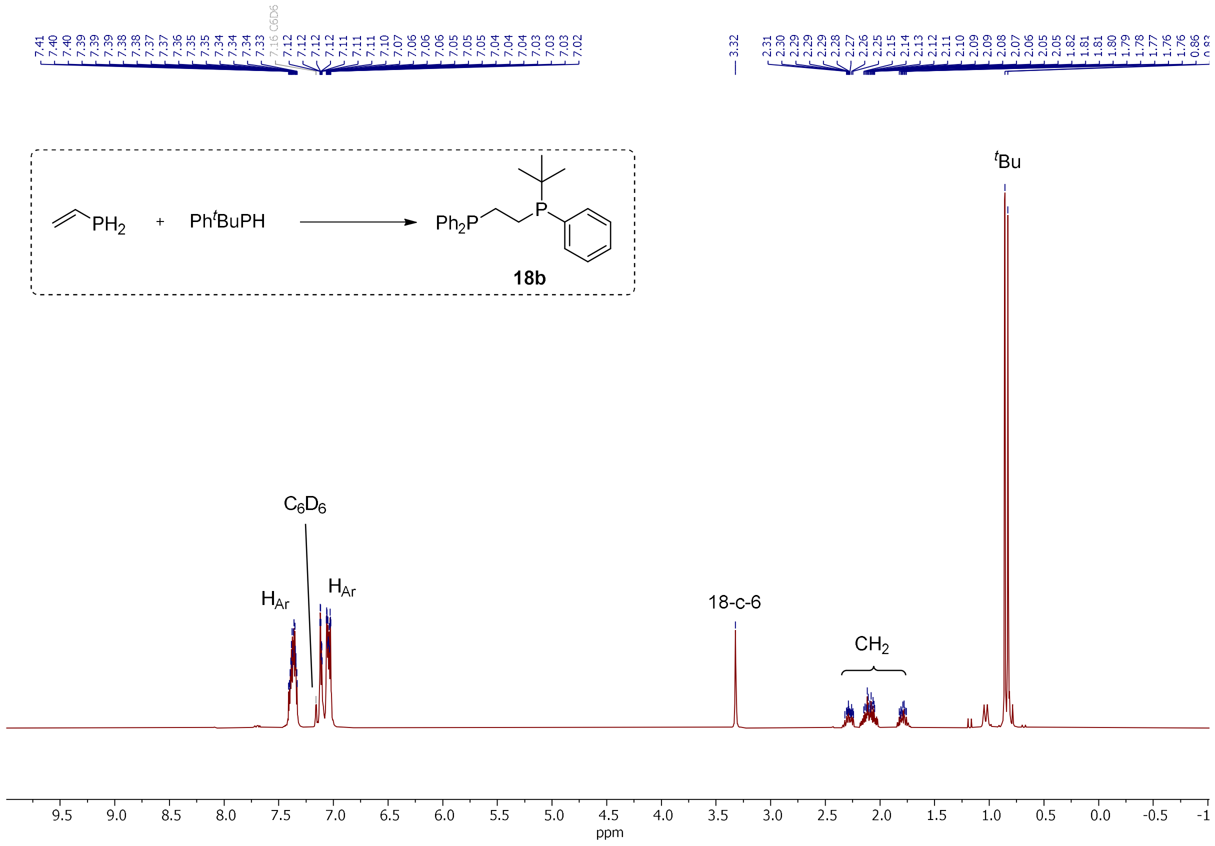

*Figure S142. ^{1}H NMR spectrum of the HP reaction of **18** with Ph^{t}BuPH in benzene-d_6 at 300 K after 28 min at RT.*

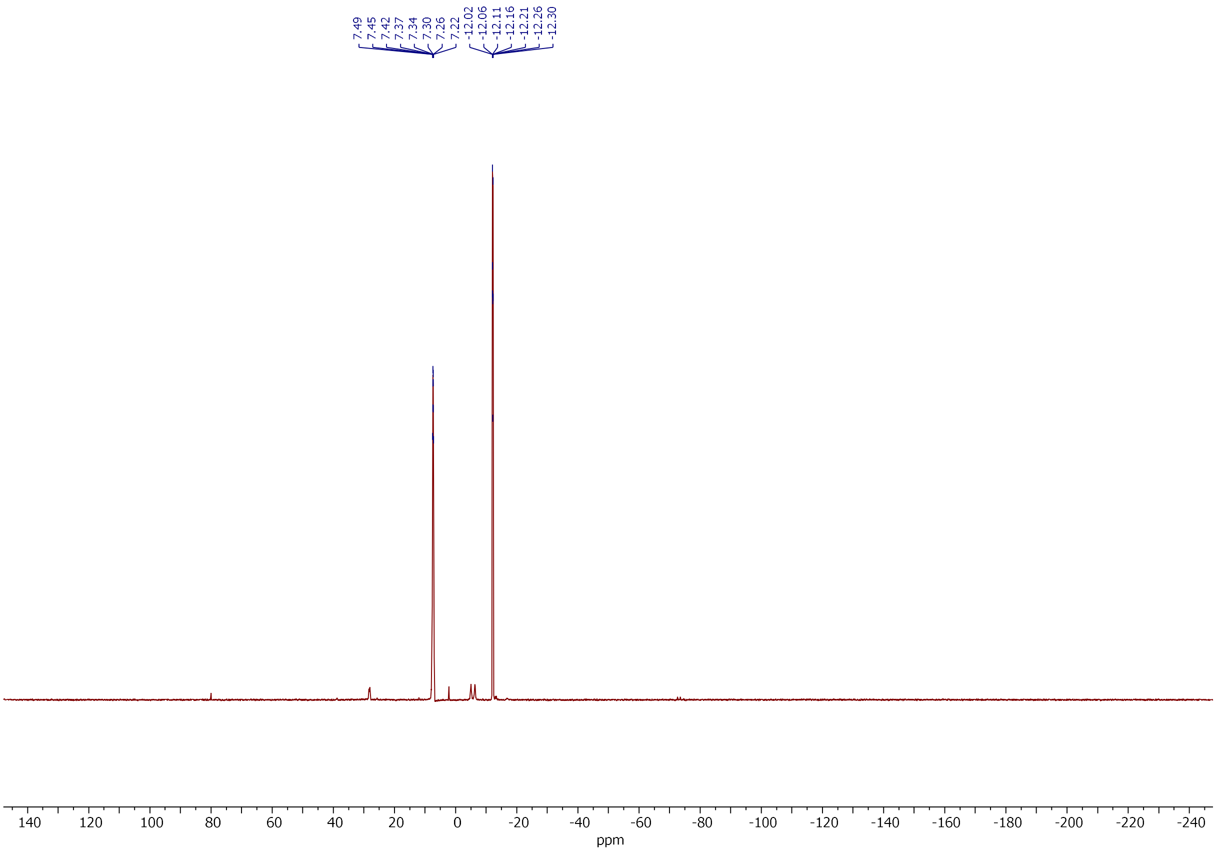

*Figure S143. ^{31}P NMR spectrum of the HP reaction of **18** with Ph^{t}BuPH in benzene-d_6 at 300 K after 28 min at RT.*

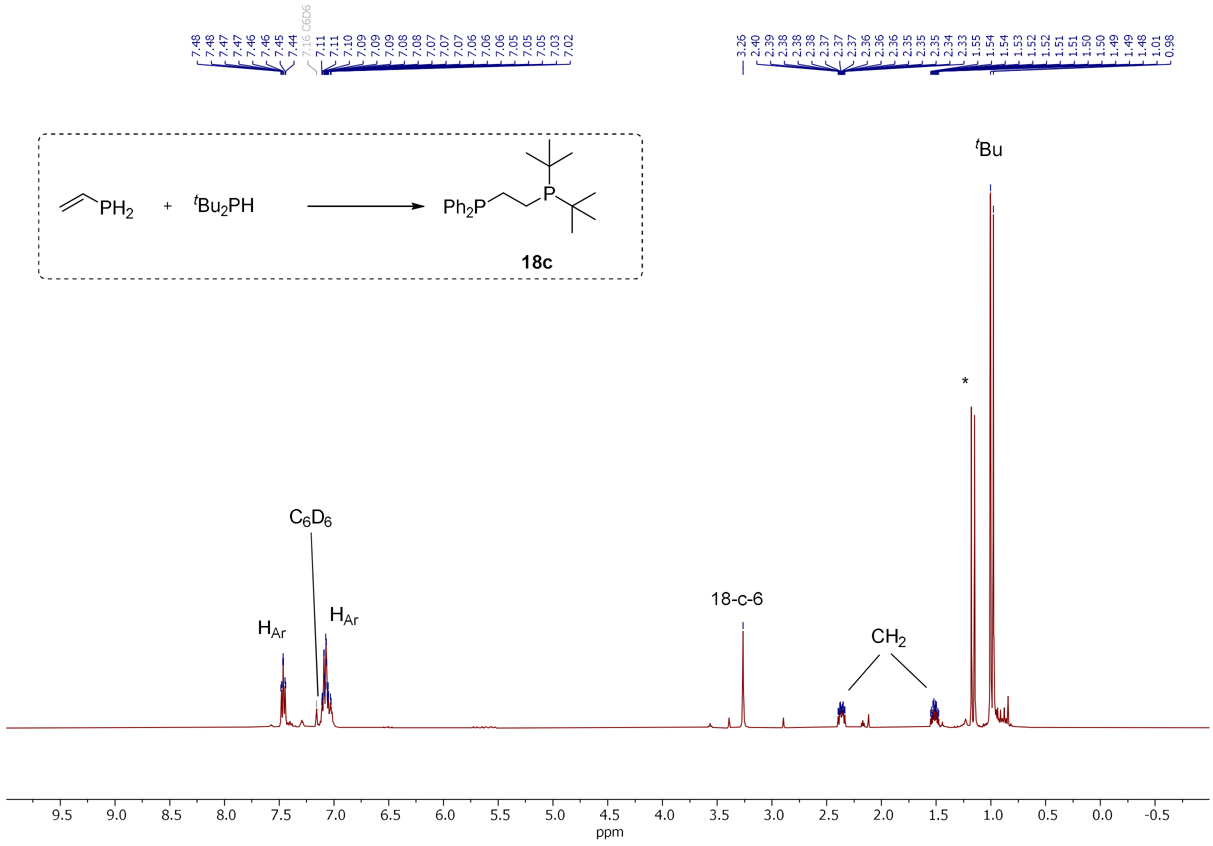

*Figure S144. ^{1}H NMR spectrum of the HP reaction of **18** with $^{t}Bu_2PH$ in benzene-d_6 at 300 K after 4 h at RT. * belongs to excess $^{t}Bu_2PH$.*

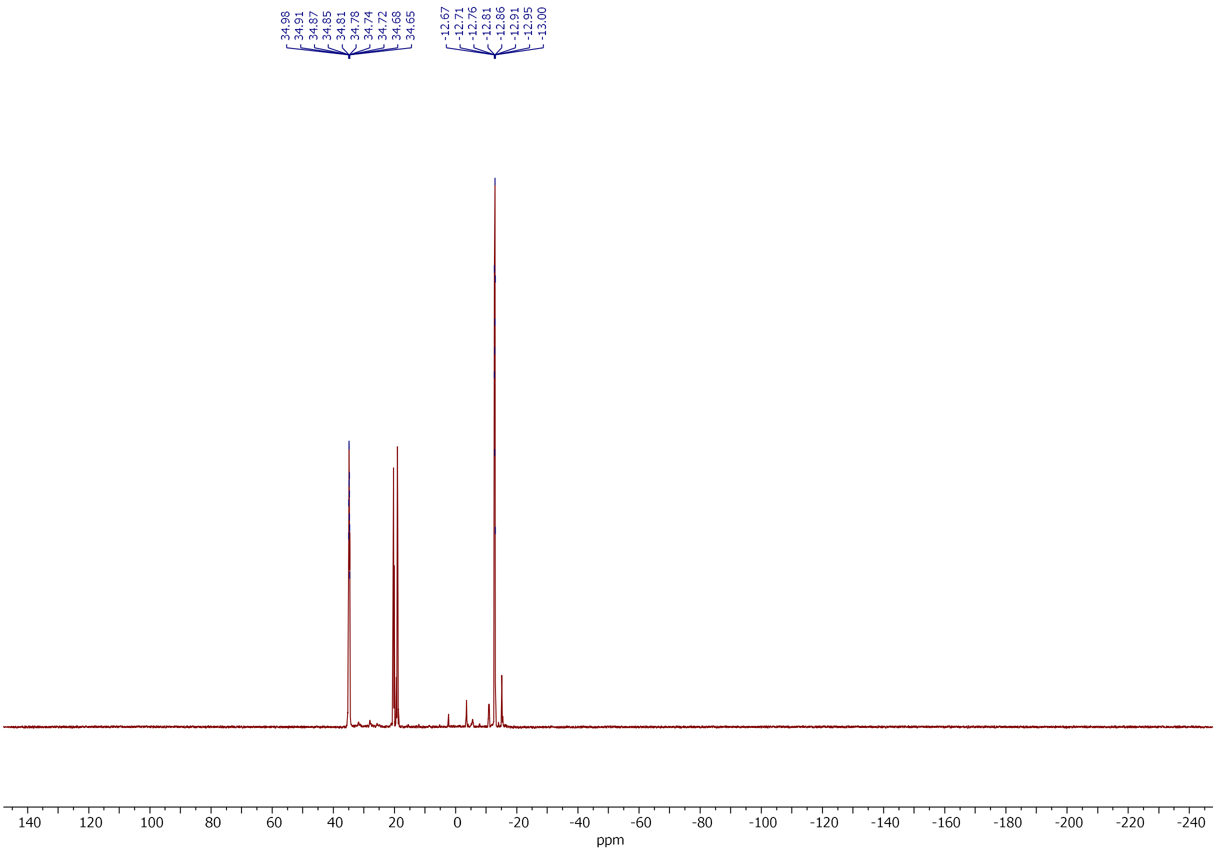

*Figure S145. ^{31}P NMR spectrum of the HP reaction of **18** with $^{t}Bu_2PH$ in benzene-d_6 at 300 K after 4 h at RT. Signal at 20 ppm belongs to excess $^{t}Bu_2PH$.*

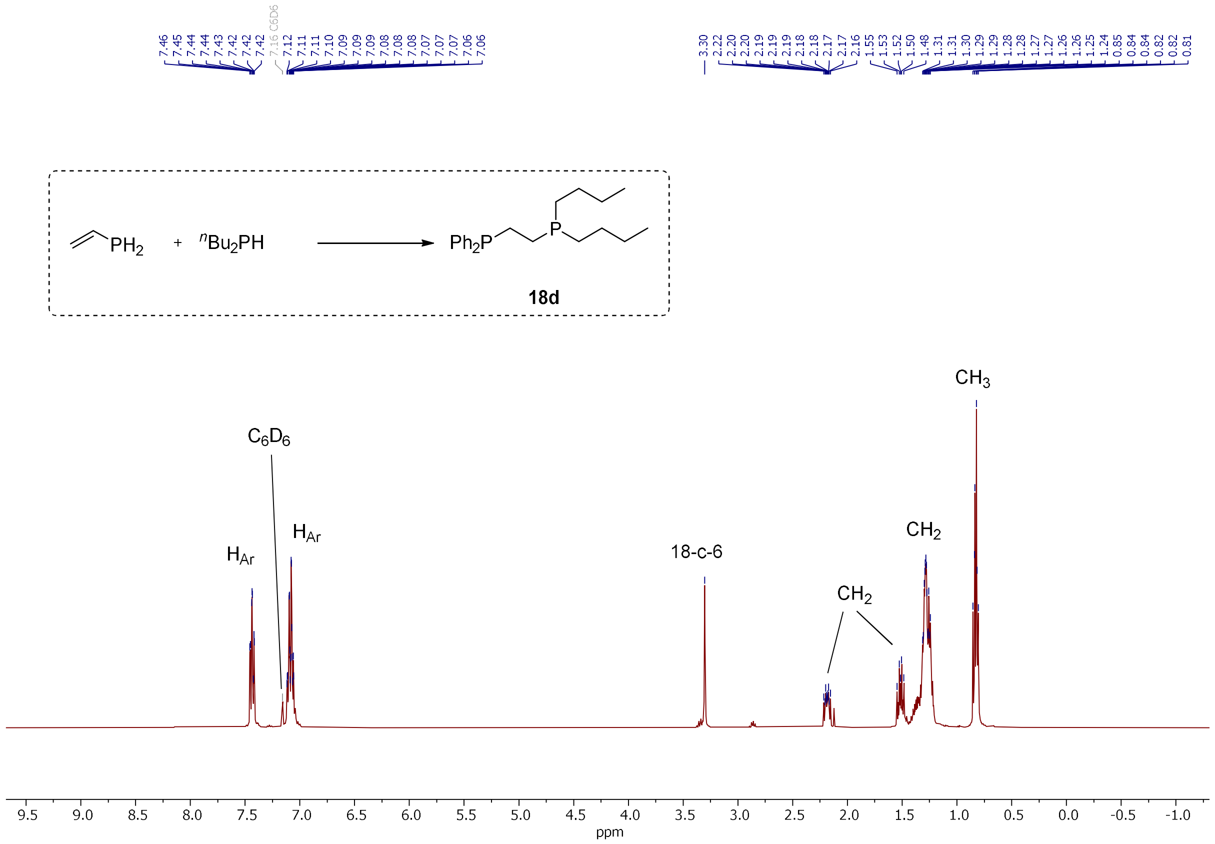

Figure S146. ^{1}*H NMR spectrum of the HP reaction of* ***18*** *with* ^{n}*Bu*$_2$*PH in benzene-d*$_6$ *at 300 K after 28 min at RT.*

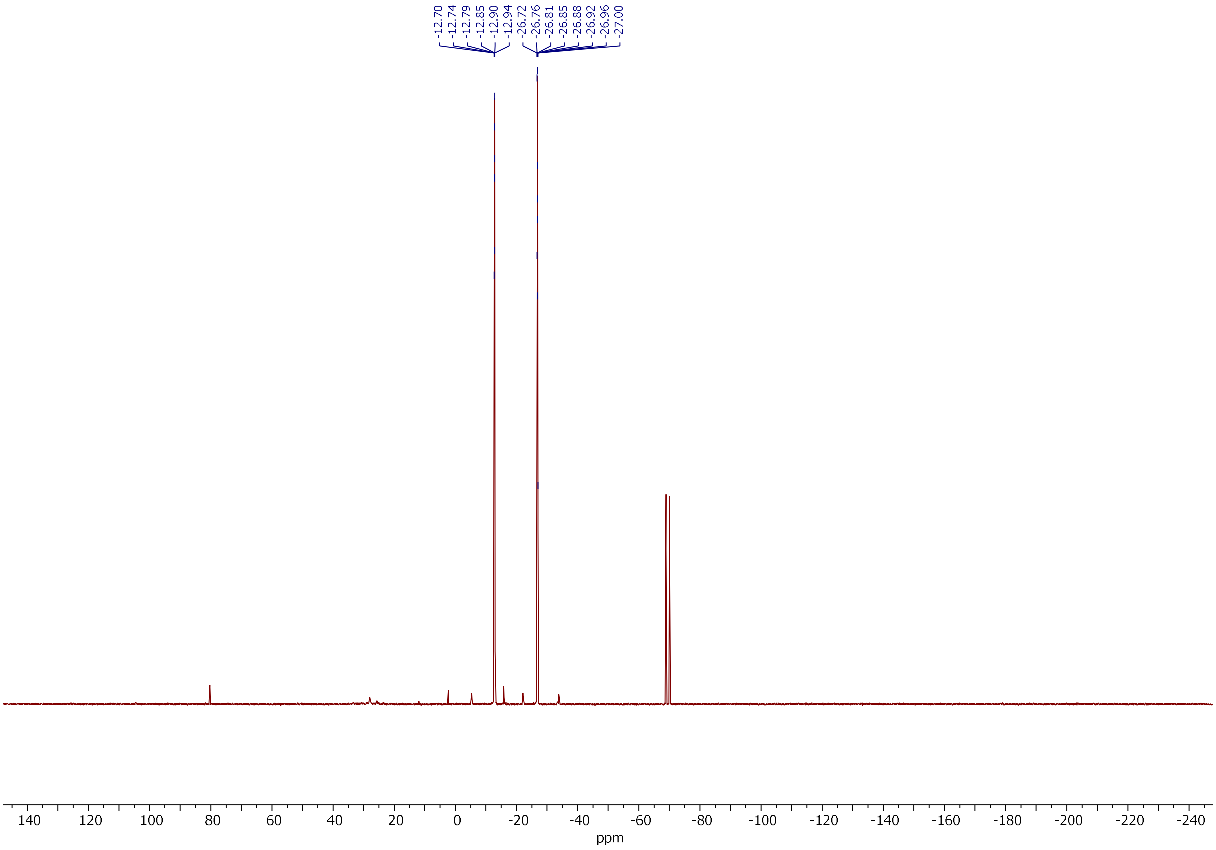

Figure S147. ^{31}*P NMR spectrum of the HP reaction of* ***18*** *with* ^{n}*Bu*$_2$*PH in benzene-d*$_6$ *at 300 K after 28 min. signal at -70 ppm belongs to excess* ^{n}*Bu*$_2$*PH at RT.*

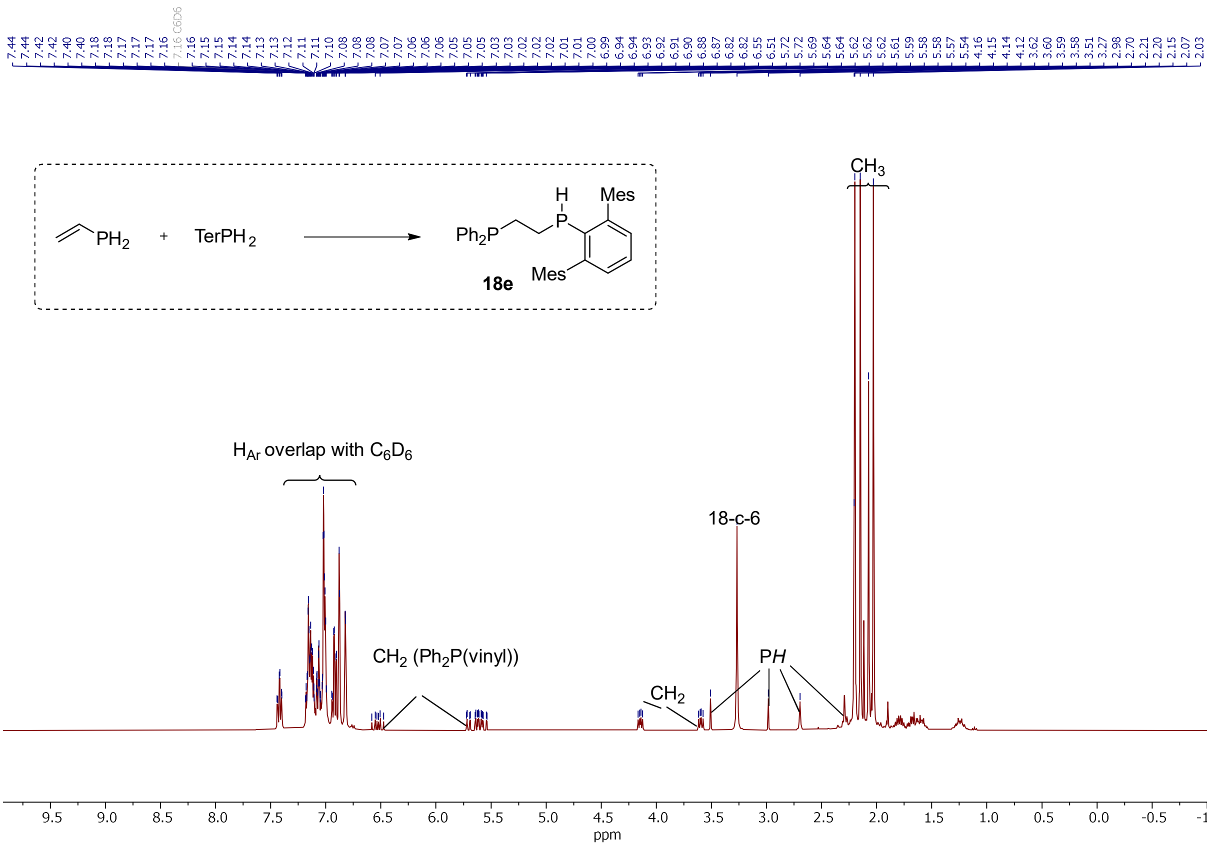

Figure S148. *^{1}H NMR spectrum of the HP reaction of* ***18*** *with $TerPH_2$ in benzene-d_6 at 300 K after 3 days at 80 °C. Mixture of products and starting materials, conversion 75 %.*

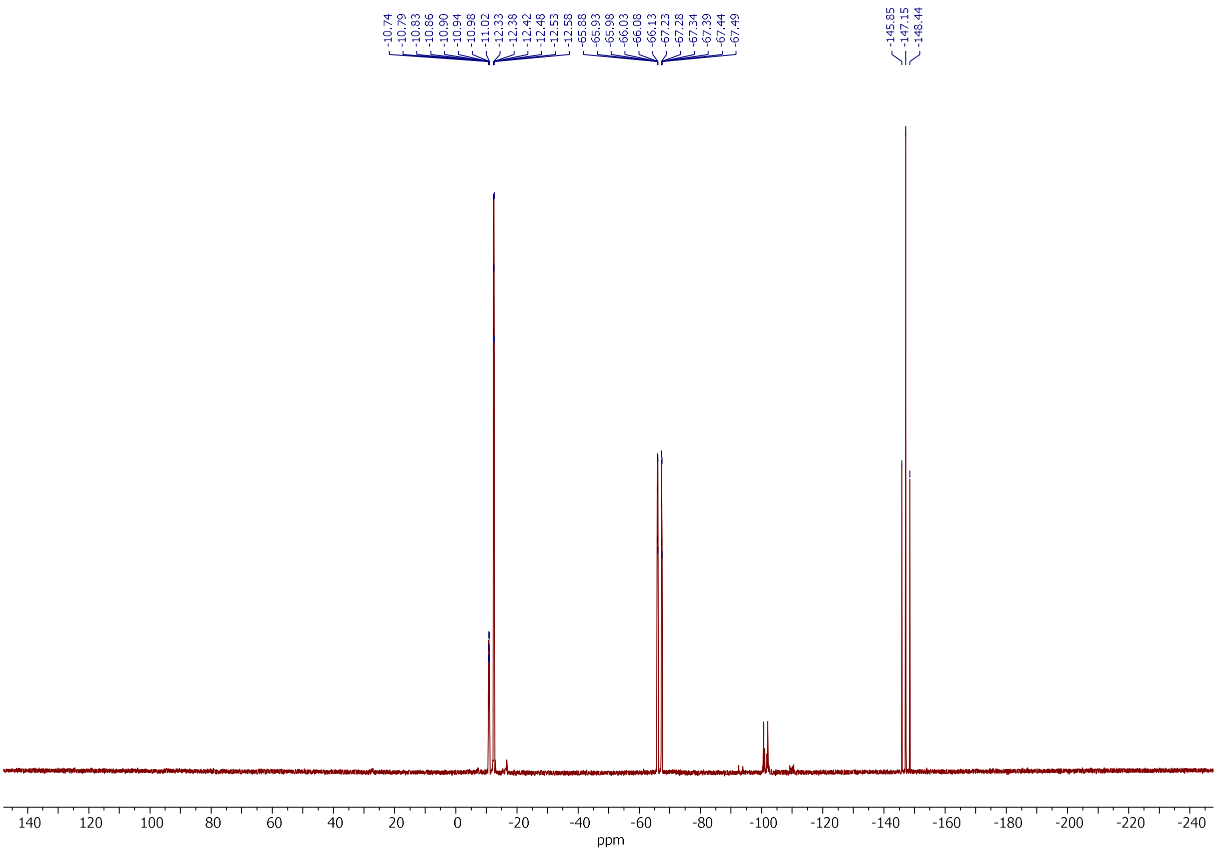

Figure S149. *^{31}P NMR spectrum of the HP reaction of* ***18*** *with $TerPH_2$ in benzene-d_6 at 300 K after 3 days at 80 °C. Mixture of products and starting materials, conversion 75 %.*

Section S3.6 – Variable Temperature (VT) NMR Spectra of the reactions of 1^AM with 2

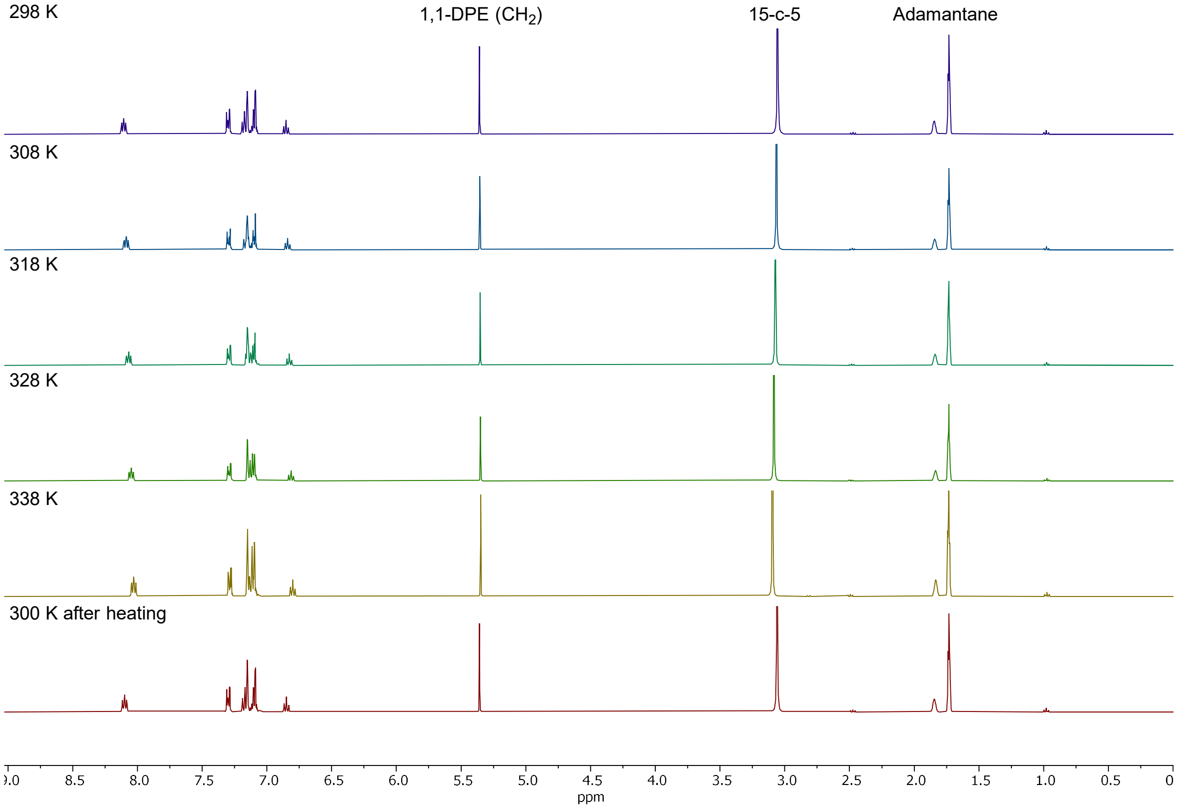

*Figure S150. Stacked ^{1}H VT NMR spectra of the stochiometric reaction of **1**^{Na} with **2** with adamantane as internal standard in benzene-d$_6$.*

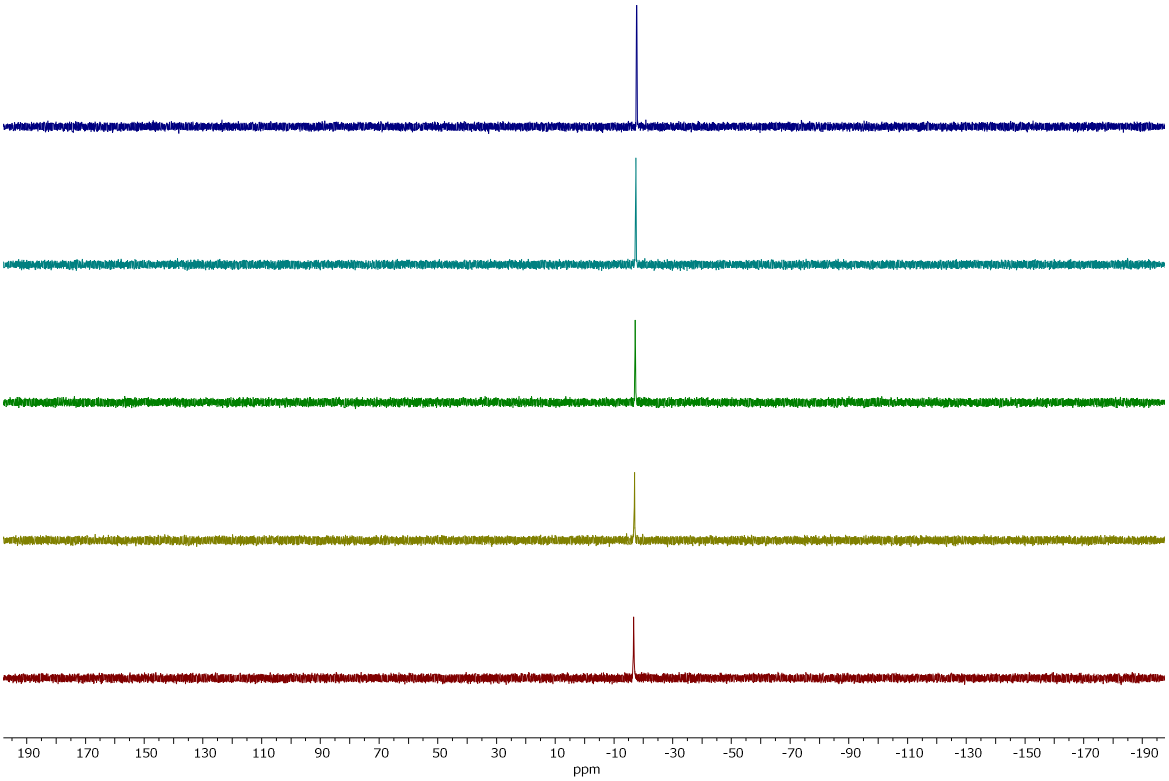

*Figure S151. Stacked ^{31}P{^{1}H} VT NMR spectra of the stochiometric reaction of **1**^{Na} with **2** with adamantane as internal standard in benzene-d$_6$.*

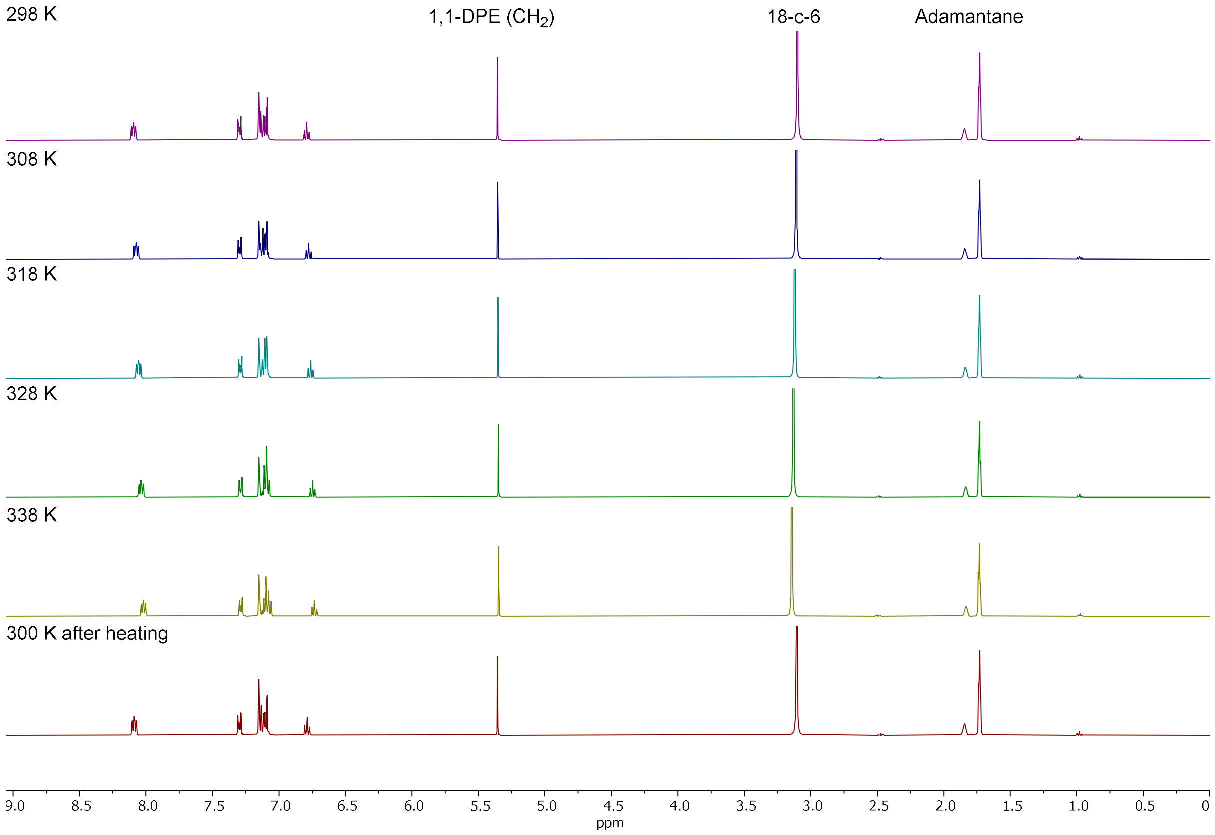

*Figure S152. Stacked ^{1}H VT NMR spectra of the stochiometric reaction of **1**^{K} with **2** with adamantane as internal standard in benzene-d$_6$.*

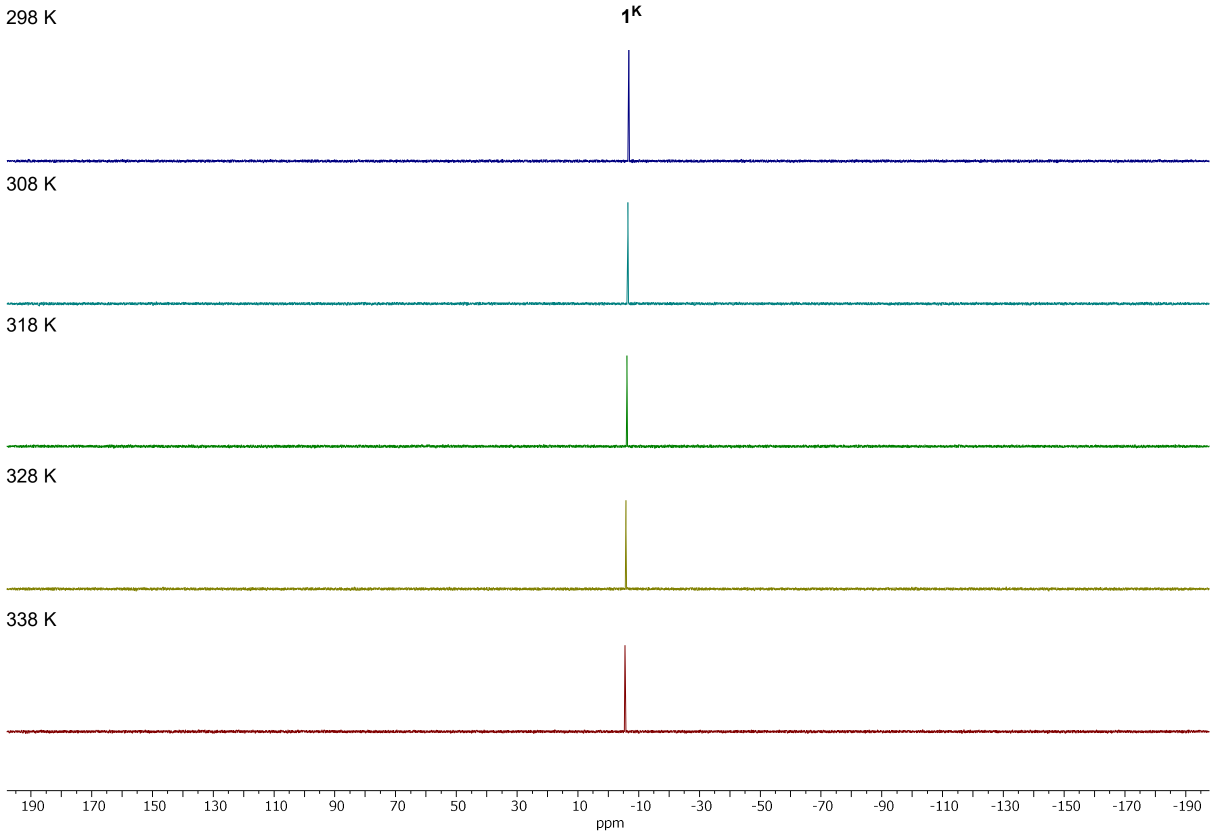

*Figure S153. Stacked $^{31}P\{^{1}H\}$ VT NMR spectra of the stochiometric reaction of **1**^{K} with **2** with adamantane as internal standard in benzene-d$_6$.*

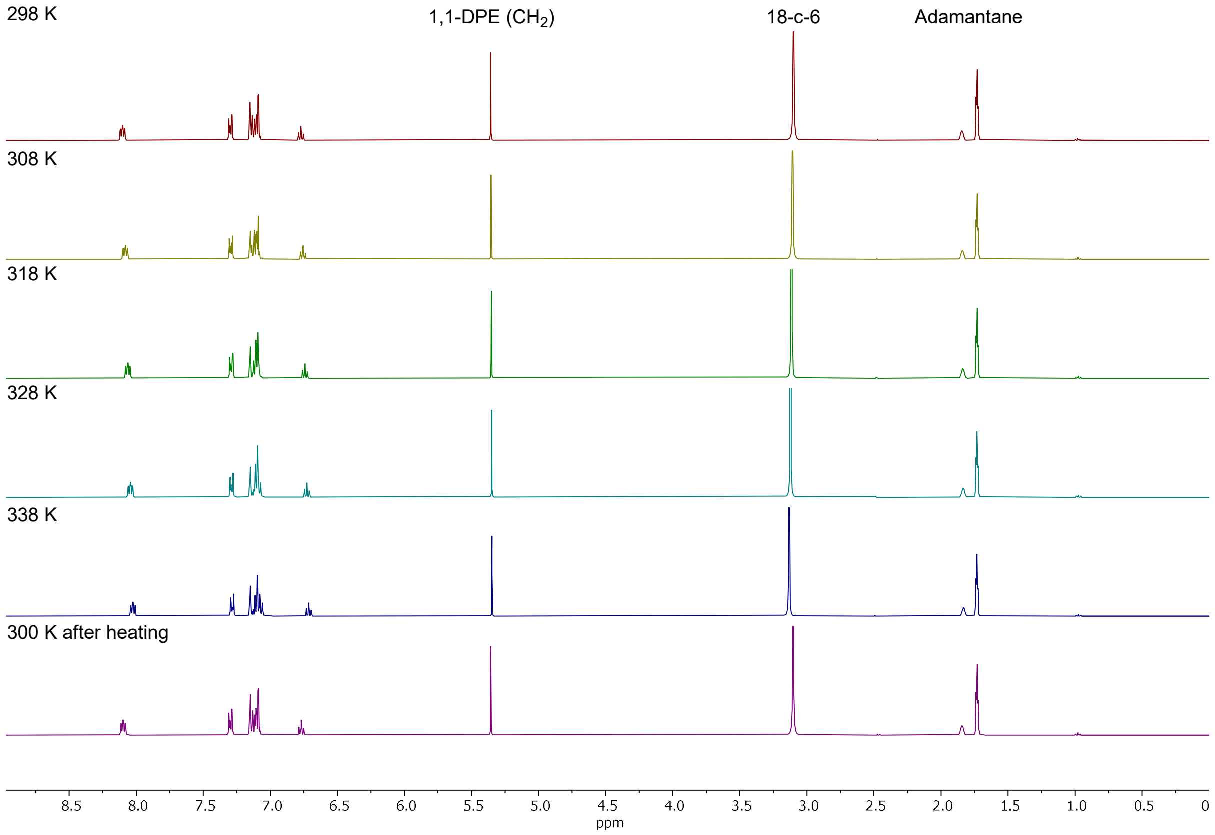

*Figure S154. Stacked ^{1}H VT NMR spectra of the stochiometric reaction of $\mathbf{1^{Rb}}$ with **2** with adamantane as internal standard in benzene-d_6.*

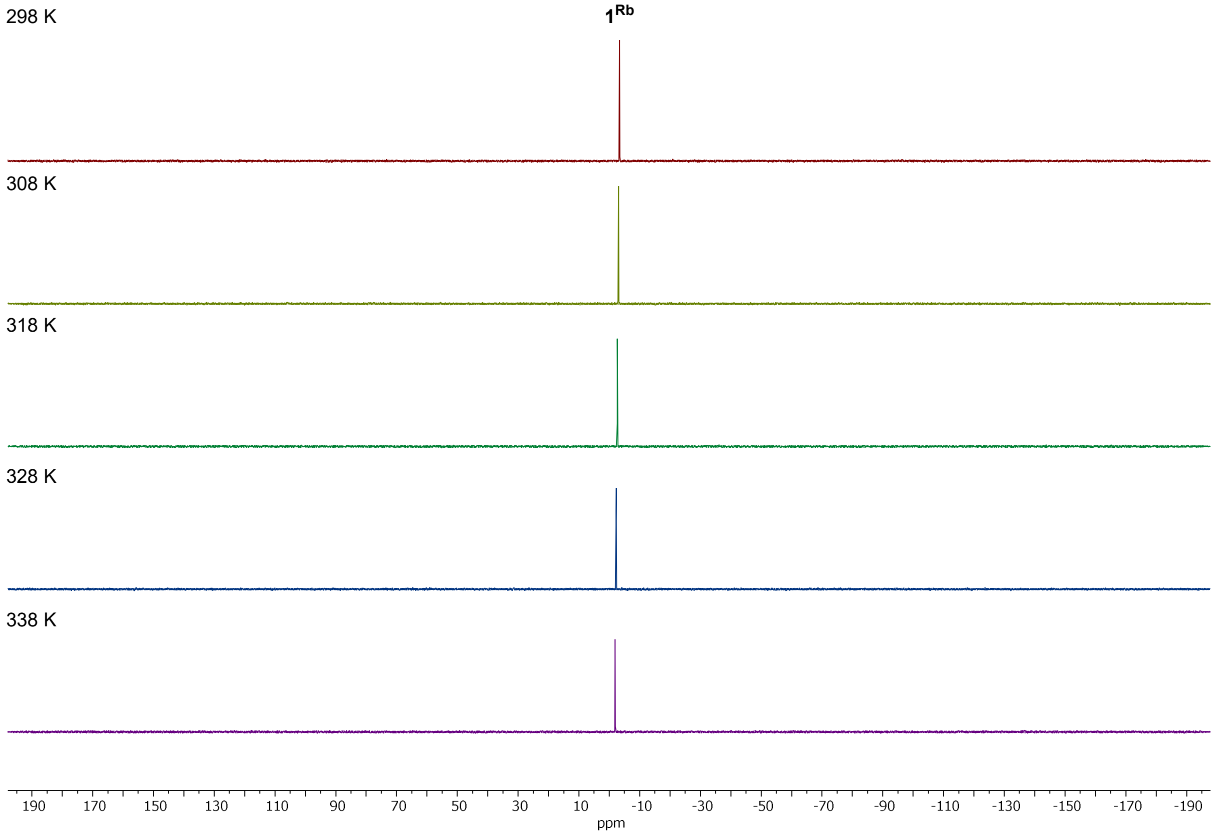

*Figure S155. Stacked $^{31}P\{^{1}H\}$ VT NMR spectra of the stochiometric reaction of $\mathbf{1^{K}}$ with **2** with adamantane as internal standard in benzene-d_6.*

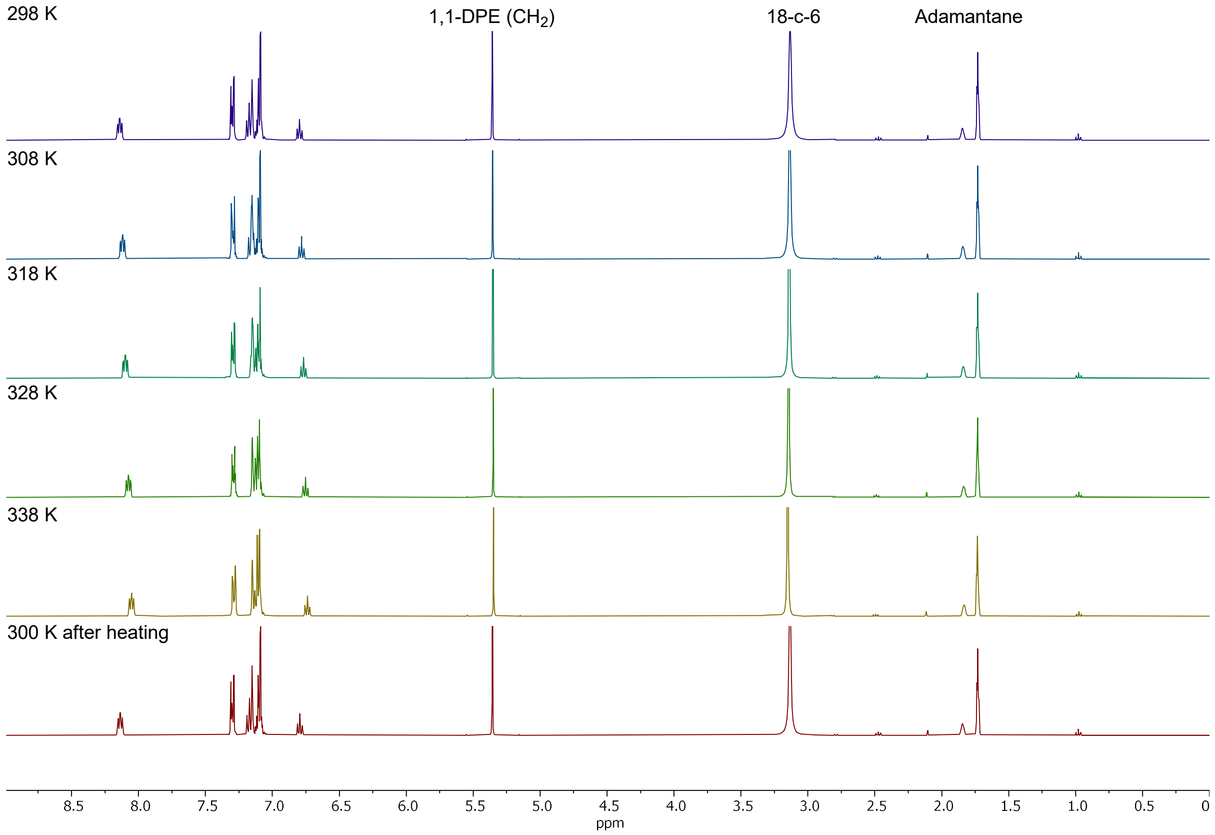

*Figure S156. Stacked ^{1}H VT NMR spectra of the stochiometric reaction of $\mathbf{1}^{Cs}$ with **2** with adamantane as internal standard in benzene-d_6.*

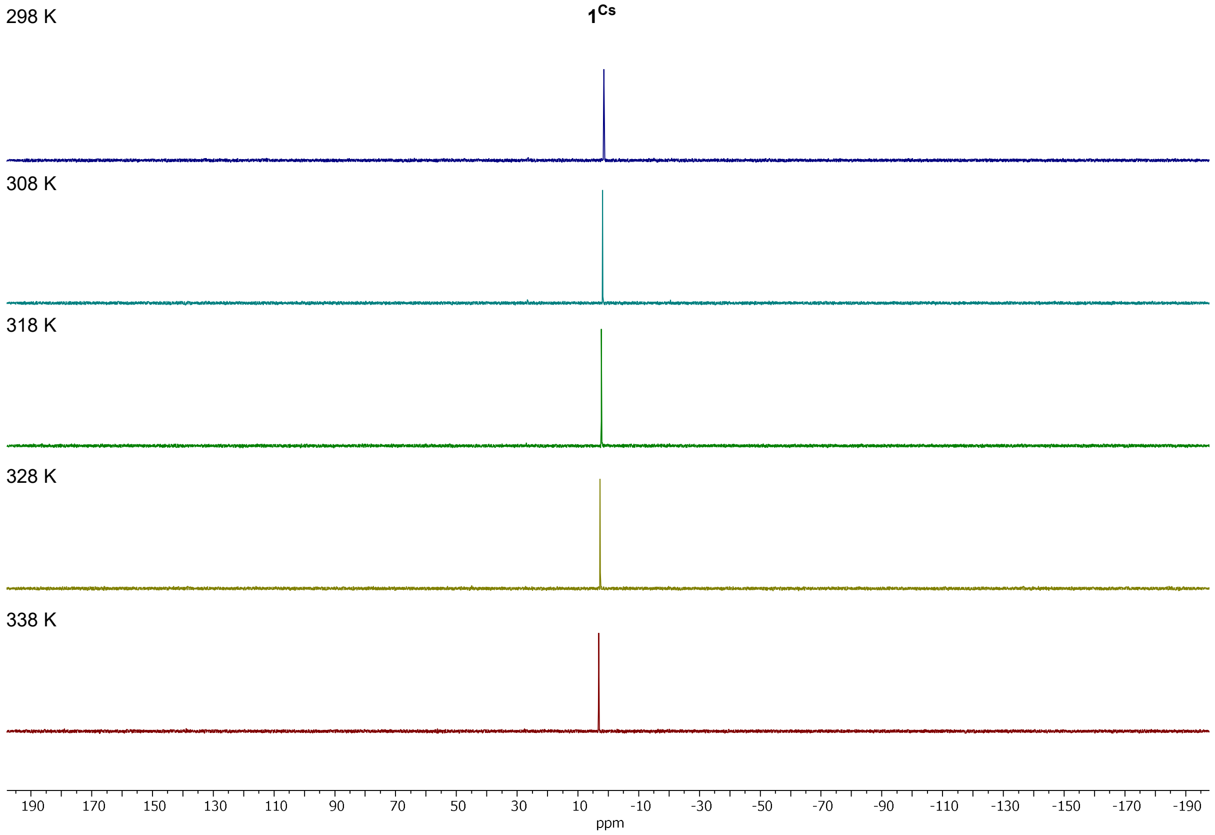

*Figure S157. Stacked $^{31}P\{^{1}H\}$ VT NMR spectra of the stochiometric reaction of $\mathbf{1}^{Cs}$ with **2** with adamantane as internal standard in benzene-d_6.*

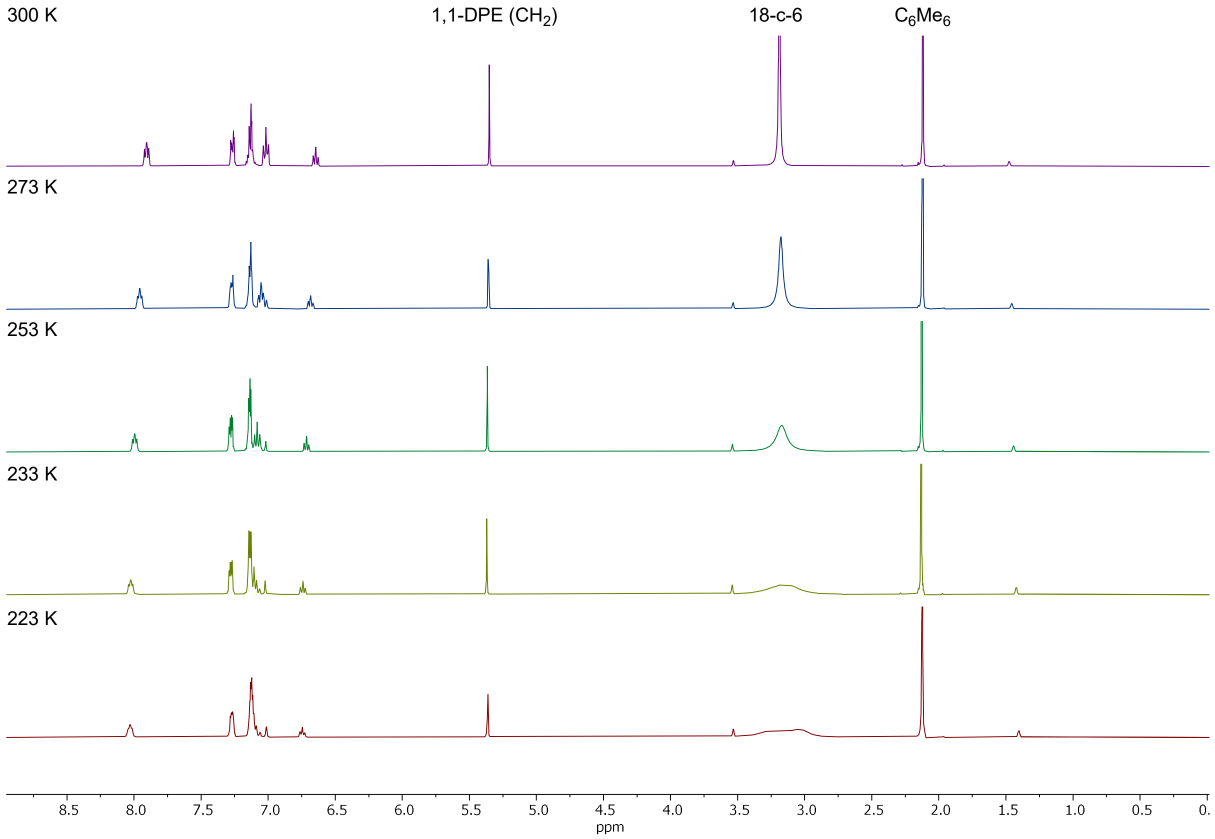

*Figure S158. Stacked ^{1}H VT NMR spectra of the stochiometric reaction of $\mathbf{1^{Cs}}$ with **2** with C_6Me_6 as internal standard in toluene-d_8.*

Section S4 – Crystallographic details

Crystallographic Details: Crystallographic data for all compounds were measured with a Rigaku Synergy-i instrument with monochromated Cu−Kα (λ = 1.54184 Å) radiation. The measured data was processed with the CrysAlisPro3 software package. The structures were solved in OLEX2 1.5[9] by dual-space direct methods with SHELXT[10], followed by full-matrix least-squares refinement using SHELXL.[11] All non-hydrogen atoms were refined anisotropically. The contribution of the hydrogen atoms, in their calculated positions, was included in the refinement using a riding model. A full listing of atomic coordinates, bond lengths, angles and displacement parameters for all the structures has been deposited at the Cambridge Crystallographic Data Centre (CCDC 2474763 (**2b**), 2474764 (**2c-O**), 2474765 (**2c-O·0.5**C_6H_6), 2474766 (**18b**), 2474767 (**18e**))

Table S3. Crystal data and structure refinement data.

Compound	**2b**	**2c-O**	**2c-O·0.5**C_6H_6	**18b**	**18e**
CCDC#	2474763	2474764	2474765	2474766	2474767
Empirical formula	$C_{24}H_{27}P$	$C_{22}H_{31}OP$	$C_{25}H_{34}OP$	$C_{24}H_{28}P_2$	$C_{38}H_{40}P_2$
Formula weight	346.42	342.44	381.49	378.4	558.64
Temperature/K	149.99(10)	100(1)	102(2)	149.98(10)	101(2)
Crystal system	monoclinic	monoclinic	monoclinic	trigonal	monoclinic
Space group	$P2_1/c$	$P2_1/n$	$P2_1/n$	R-3	$P2_1/c$
a/Å	12.0409(2)	6.0723(2)	6.31810(10)	45.1878(7)	37.0739(5)
b/Å	15.3278(4)	17.7702(5)	17.3140(3)	45.1878(7)	10.97010(14)
c/Å	11.1650(4)	20.1582(6)	19.9947(3)	5.73443(13)	15.9967(2)
α/°	90	90	90	90	90
β/°	100.386(2)	94.974(3)	95.612(2)	90	100.9041(12)
γ/°	90	90	90	120	90
Volume/Å^3	2026.86(9)	2167.00(11)	2176.77(6)	10140.6(4)	6388.45(15)
Z	4	4	4	18	8
ρ_{calc}g/cm^3	1.135	1.05	1.164	1.115	1.162
μ/mm^{-1}	1.195	1.141	1.186	1.764	1.403
F(000)	744	744	828	3636	2384
Crystal size/mm^3	0.236 × 0.067 × 0.047	0.285 × 0.285 × 0.06	0.243 × 0.102 × 0.049	0.235 × 0.05 × 0.046	0.386 × 0.367 × 0.213
Radiation	Cu Kα (λ = 1.54184)	Cu Kα (λ = 1.54184)	Cu Kα (λ = 1.54184)	Cu Kα (λ = 1.54184)	Cu Kα (λ = 1.54184)
2Θ range for data collection/°	7.464 to 144.264	6.642 to 132.686	6.768 to 146.552	6.776 to 146.08	4.854 to 147.144
Reflections collected	20367	11554	14570	17135	42492
Independent reflections	3937 [R_{int} = 0.0547, R_{sigma} = 0.0409]	3744 [R_{int} = 0.0382, R_{sigma} = 0.0428]	4340 [R_{int} = 0.0342, R_{sigma} = 0.0378]	4465 [R_{int} = 0.0279, R_{sigma} = 0.0284]	12674 [R_{int} = 0.0490, R_{sigma} = 0.0322]
Data/restraints/parameters	3937/9/229	3744/0/223	4340/0/250	4465/0/238	12674/0/742
Goodness-of-fit on F^2	1.066	1.047	1.058	1.051	1.112
Final R indexes [I>=2σ (I)]	R_1 = 0.0704, wR_2 = 0.1730	R_1 = 0.0560, wR_2 = 0.1371	R_1 = 0.0398, wR_2 = 0.1074	R_1 = 0.0424, wR_2 = 0.1057	R_1 = 0.0752, wR_2 = 0.1979
Final R indexes [all data]	R_1 = 0.0852, wR_2 = 0.1839	R_1 = 0.0674, wR_2 = 0.1435	R_1 = 0.0453, wR_2 = 0.1109	R_1 = 0.0505, wR_2 = 0.1096	R_1 = 0.0789, wR_2 = 0.2000
Largest diff. peak/hole / e Å^{-3}	0.76/-0.20	0.45/-0.36	0.49/-0.32	0.44/-0.29	0.61/-0.48

References

[1] F. Krämer, M. H. Crabbe, A. R. Kennedy, C. E. Weetman, I. Fernández, R. E. Mulvey, *Crown Ether Supported Alkali Metal Phosphides: Synthesis, Structures and Bonding, Chem. Eur. J.* **2025**, e02127.

[2] Z. Rohlík, P. Holzhauser, J. Kotek, J. Rudovský, I. Němec, P. Hermann, I. Lukeš, *Synthesis and coordination properties of palladium(II) and platinum(II) complexes with phosphonated triphenylphosphine derivatives, J. Organomet. Chem.* **2006**, *691*, 2409-2423.

[3] A. Naiini, Y. Han, M. Akinc, J. Verkade, *Synthesis and pyrolysis studies of [(tert-Bu) 2GaPR2] x, Inorg. Chem.* **1993**, *32*, 5394-5395.

[4] J. Bresien, C. Hering, A. Schulz, A. Villinger, *Dimers and Trimers of Diphosphenes: A Wealth of Cyclo-Phosphanes, Chem. Eur. J.* **2014**, *20*, 12607-12615.

[5] K. Issleib, W. Seidel, *Darstellung und chemisches Verhalten aliphatischer und cycloaliphatischer Diphosphine, R2P�PR2, Chem. Ber.* **1959**, *92*, 2681-2694.

[6] W. Voskuil, J. F. Arens, *Chemistry of acetylenic ethers LXII: Tertiary phosphines with an acetylene-phosphorus bond, Recl. Trav. Chim. Pays-Bas* **1962**, *81*, 993-1008.

[7] L. Orzechowski, G. Jansen, S. Harder, *Methandiide Complexes (R2CM2) of the Heavier Alkali Metals (M=Potassium, Rubidium, Cesium): Reaching the Limit?, Angew. Chem. Int. Ed.* **2009**, *48*, 3825-3829.

[8] J. B. Lambert, L. Lin, S. Keinan, *The C5SiMe7+ cation: pyramidal, bicyclic, or cyclohexadienyl?, Org. Biomol. Chem.* **2003**, *1*, 2559-2565.

[9] O. V. Dolomanov, L. J. Bourhis, R. J. Gildea, J. A. K. Howard, H. Puschmann, *OLEX2: a complete structure solution, refinement and analysis program, J. Appl. Crystallogr.* **2009**, *42*, 339-341.

[10] G. Sheldrick, *SHELXT - Integrated space-group and crystal-structure determination, Acta Crystallogr. Section A* **2015**, *71*, 3-8.

[11] G. Sheldrick, *Crystal structure refinement with SHELXL, Acta Crystallogr. Section C* **2015**, *71*, 3-8.

[12] F. Neese, *An improvement of the resolution of the identity approximation for the formation of the Coulomb matrix, J. Comput. Chem.* **2003**, *24*, 1740-1747.

[13] D. Bykov, P. Taras, I. Róbert, K. Simone, B. Ute, V. Edward, F. and Neese, *Efficient implementation of the analytic second derivatives of Hartree–Fock and hybrid DFT energies: a detailed analysis of different approximations, Molecular Physics* **2015**, *113*, 1961-1977.

[14] M. Garcia-Ratés, F. Neese, *Efficient implementation of the analytical second derivatives of hartree–fock and hybrid DFT energies within the framework of the conductor-like polarizable continuum model, J. Comput. Chem.* **2019**, *40*, 1816-1828.

[15] M. Garcia-Ratés, F. Neese, *Effect of the Solute Cavity on the Solvation Energy and its Derivatives within the Framework of the Gaussian Charge Scheme, J. Comput. Chem.* **2020**, *41*, 922-939.

[16] F. Neese, *Software update: The ORCA program system—Version 5.0, WIREs Comput. Mol. Sci.* **2022**, *12*, e1606.

[17] F. Neese, *The SHARK integral generation and digestion system, J. Comput. Chem.* **2023**, *44*, 381-396.

[18] F. Neese, *Software Update: The ORCA Program System—Version 6.0, WIREs Comput. Mol. Sci.* **2025**, *15*, e70019.

[19] J. P. Perdew, *Density-functional approximation for the correlation energy of the inhomogeneous electron gas, Phys. Rev. B* **1986**, *33*, 8822-8824.

[20] A. D. Becke, *Density-functional exchange-energy approximation with correct asymptotic behavior, Phys. Rev. A* **1988**, *38*, 3098-3100.

[21] F. Weigend, *Accurate Coulomb-fitting basis sets for H to Rn, Phys Chem Chem Phys* **2006**, *8*, 1057-1065.

[22] S. Grimme, J. Antony, S. Ehrlich, H. Krieg, *A consistent and accurate ab initio parametrization of density functional dispersion correction (DFT-D) for the 94 elements H-Pu, J. Chem. Phys.* **2010**, *132*, 154104.

[23] S. Grimme, S. Ehrlich, L. Goerigk, *Effect of the damping function in dispersion corrected density functional theory, J. Comput. Chem.* **2011**, *32*, 1456-1465.

[24] A. V. Marenich, C. J. Cramer, D. G. Truhlar, *Universal Solvation Model Based on Solute Electron Density and on a Continuum Model of the Solvent Defined by the Bulk Dielectric Constant and Atomic Surface Tensions, J. Phys. Chem. B* **2009**, *113*, 6378-6396.

[25] C. Gonzalez, H. B. Schlegel, *Reaction path following in mass-weighted internal coordinates, J. Phys. Chem.* **1990**, *94*, 5523-5527.

[26] Y. Zhao, D. G. Truhlar, *The M06 suite of density functionals for main group thermochemistry, thermochemical kinetics, noncovalent interactions, excited states, and transition elements: two new functionals and systematic testing of four M06-class functionals and 12 other functionals, Theor. Chem. Acc.* **2008**, *120*, 215-241.

[27] F. Weigend, R. Ahlrichs, *Balanced basis sets of split valence, triple zeta valence and quadruple zeta valence quality for H to Rn: Design and assessment of accuracy, Phys. Chem. Chem. Phys.* **2005**, *7*, 3297-3305.
